# Hypomethylation of Intragenic LINE-1 Represses Transcription in Cancer Cells through AGO2

**DOI:** 10.1371/journal.pone.0017934

**Published:** 2011-03-15

**Authors:** Chatchawit Aporntewan, Chureerat Phokaew, Jittima Piriyapongsa, Chumpol Ngamphiw, Chupong Ittiwut, Sissades Tongsima, Apiwat Mutirangura

**Affiliations:** 1 Department of Mathematics, Faculty of Science, Chulalongkorn University, Bangkok, Thailand; 2 Inter-Department Program of BioMedical Sciences, Faculty of Graduate School, Chulalongkorn University, Bangkok, Thailand; 3 National Center for Genetic Engineering and Biotechnology, Genome Institute, Thailand Science Park, Pathumtani, Thailand; 4 Department of Anatomy, Faculty of Medicine, Center of Excellence in Molecular Genetics of Cancer and Human Diseases, Chulalongkorn University, Bangkok, Thailand; Bellvitge Biomedical Research Institute (IDIBELL), Spain

## Abstract

In human cancers, the methylation of long interspersed nuclear element -1 (LINE-1 or L1) retrotransposons is reduced. This occurs within the context of genome wide hypomethylation, and although it is common, its role is poorly understood. L1s are widely distributed both inside and outside of genes, intragenic and intergenic, respectively. Interestingly, the insertion of active full-length L1 sequences into host gene introns disrupts gene expression. Here, we evaluated if intragenic L1 hypomethylation influences their host gene expression in cancer. First, we extracted data from L1base (http://l1base.molgen.mpg.de), a database containing putatively active L1 insertions, and compared intragenic and intergenic L1 characters. We found that intragenic L1 sequences have been conserved across evolutionary time with respect to transcriptional activity and CpG dinucleotide sites for mammalian DNA methylation. Then, we compared regulated mRNA levels of cells from two different experiments available from Gene Expression Omnibus (GEO), a database repository of high throughput gene expression data, (http://www.ncbi.nlm.nih.gov/geo) by chi-square. The odds ratio of down-regulated genes between demethylated normal bronchial epithelium and lung cancer was high (p<1E^−27^; OR = 3.14; 95% CI = 2.54–3.88), suggesting cancer genome wide hypomethylation down-regulating gene expression. Comprehensive analysis between L1 locations and gene expression showed that expression of genes containing L1s had a significantly higher likelihood to be repressed in cancer and hypomethylated normal cells. In contrast, many mRNAs derived from genes containing L1s are elevated in Argonaute 2 (AGO2 or EIF2C2)-depleted cells. Hypomethylated L1s increase L1 mRNA levels. Finally, we found that AGO2 targets intronic L1 pre-mRNA complexes and represses cancer genes. These findings represent one of the mechanisms of cancer genome wide hypomethylation altering gene expression. Hypomethylated intragenic L1s are a nuclear siRNA mediated *cis*-regulatory element that can repress genes. This epigenetic regulation of retrotransposons likely influences many aspects of genomic biology.

## Introduction

In cancer, DNA methylation in the rest of the genome, particularly at a long interspersed nuclear element-1 (LINE-1 or L1) retrotransposon, is generally depleted and this event occurs within the context of genome wide or global hypomethylation [1], [2], [3]. Global hypomethylation may play several roles in multistep carcinogenesis. Most commonly recognized effect is to facilitate chromosomal instability [4], probably mediated by hypomethylated genome associated replication independent DNA double strand break error prone repair [5], [6]. Recently, there is a report that hypomethylation of a L1 activates alternate promoter of MET oncogene [7]. However, the role of global L1 methylation, on genome wide gene expression, is less frequently studied and thus not well-characterized.

DNA methylation is a fundamental molecular characteristic of the human genome, and alteration of this epigenetic regulation is associated with cancers [2]. The effects of promoter methylation on chromatin configuration and gene transcription have been well-documented [8]. In contrast, the mechanisms, how DNA methylation within a gene (gene body methylation) controls gene expression, are less known. Gene body methylation changes may possess several consequences. Unique methylated sequences in introns are frequently found in highly expressed genes [9]. In contrast, the formation of heterochromatin by dense intragenic DNA methylation limits the efficiency of RNA polymerase [10]. Nevertheless, these evidences implied that methylation of intragenic repetitive sequences, including L1s, may also be important for maintaining the normal function of linked genomic loci.

L1s are also widely distributed in the genome [11]. L1 insertion has several potential functional consequences [12]. It is notable that L1s are not evenly distributed [11] and many are excluded from genomic regions containing housekeeping genes [13]. A genetically engineered in vitro study demonstrated that the insertion of active L1 sequences into host gene introns disrupts gene expression [14]. Throughout evolution, retrotransposition events produced >500,000 copies of L1 in the human genome [15]. However, not all L1s are full length and active; most are truncated. There are 80–100 retrotransposition-competent L1s in the human genome, but only six of these are thought to underlie all historic retrotransposition events [16]. More than 10,000 L1s are longer than 4.5 kb and contain a 5′ UTR, two open reading frames and a 3′ UTR that features a polyadenylation signal [17]. More than 2,000 of these L1s are intragenic, and they reside within more than 1,000 genes.

Recently, we evaluated the methylation patterns of the 5′ UTRs of L1 sequences [1], [18] and found that methylation levels vary at each locus and in different cell types in wild-type cells. In human cancers, the methylation of L1 retrotransposons is reduced variably [1], [18], [19]. The loss of genome wide L1 methylation in cancerous cells occurs as a generalized process. However, L1 methylation is influenced by its location in the genome [18]. For example, L1s in different introns of the same genes are generally modified in a similar way [18]. L1 hypomethylation is correlated with certain cellular phenotypes. In cancer, L1 hypomethylation is directly associated with multistep carcinogenesis and aggressive cancers with poor prognoses [1], [20], [21], [22], [23], [24]. Moreover, in normal cells methylation of L1 may be altered in association with certain cellular phenotypes such as high cancer risk, tissue differentiation and dietary [25], [26], [27], [28], [29], [30], [31], [32], [33]. Interestingly, intersperse repetitive sequence (IRS) hypomethylation patterns are different in cells with different phenotypes. For example, Alu hypomethylation is commonly found in aging cells, but L1 hypomethylation is not [34]. These lines of evidence lead us to hypothesize that thousands of genes may be regulated by intragenic L1 hypomethylation in cancerous cells.

Here, we extracted data from L1base (http://l1base.molgen.mpg.de) [17], a database containing putatively active L1 insertions, to compare intragenic and intergenic L1 characters. Then, we compared mRNA levels from hypomethylated normal cells and cancer expression array libraries, available from Gene Expression Omnibus (GEO), a database repository of high throughput gene expression data, (http://www.ncbi.nlm.nih.gov/geo) [35], [36]. Finally, comprehensive analyses between L1 locations and genome wide gene expression were performed to evaluate gene regulatory mechanisms of intragenic L1s in cancer.

## Results

### Intragenic and intergenic L1 sequences show distinct structural features

**Figure 1:**
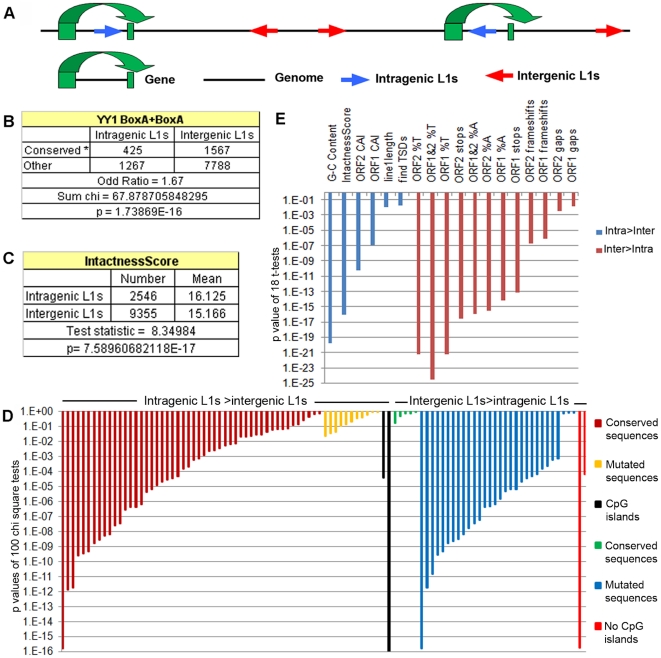
Intragenic L1s are conserved. A) L1s were divided into two classes, intragenic and intergenic L1s which are represented by blue and red arrows, respectively. B) The differences in structural characteristics between L1 groups were analyzed using the chi-square test, for categorical features and C) homoscedastic *t*-test for non-categorical features. D) 100 p-values of chi-square tests of three classes of L1 sequence characters, the conserved sequences, the mutated sequences and the presence of CpG islands, that are found overrepresented or underrepresented in intragenic L1s were displayed, intragenic L1s>intergenic L1s and intergenic L1s>intragenic L1s, respectively. E) 18 p-values of t-tests of L1 characters that are overrepresented and underrepresented in intragenic L1s were shown in blue, intra>inter, and red color, inter>intra, respectively.

Sequence variants of each long L1 are classified according to evolutionary period and retrotransposition activity [17]. Here, we analyzed if L1s are distinguishable depending on locations if the sequences are within genes, intragenic, or in between genes, intergenic (Fig. 1A). Various characteristics, described in L1base [17] including chromosomal location, subfamily, sequence and CpG islands, of 9,355 intergenic L1s and 2,546 intragenic L1s found in 1,454 genes were evaluated by 218 chi-square tests and 18 t-tests (Supporting Table S1 and S2). Examples of a chi-square test and a t-test were demonstrated (Fig. 1B and 1C). Statistical analysis revealed that there are numerous structural characteristics of intragenic L1s that are distinct from intergenic L1s (Fig. 1 and Supporting Table S2). 100 chi-square tests analyzed variations of the sequences that determine L1 transcriptional and retrotranspositional activity and the presence of CpG islands and 57 of the tests were significantly different at p values <0.001 (Fig. 1D and Supporting Table S2). These tests showed the prevalence's of conserved sequences of intragenic L1s were always higher than intergenic L1s whereas all mutated sequences were more common in intergenic L1s (Fig. 1D and Supporting Table S2). In addition, more frequent CpG islands were observed in intragenic L1s (Fig. 1D and Supporting Table S2). This finding was supported by comparing means of 18 features by t-test (Fig. 1C, 1E and Supporting Table S2). Intergenic L1s contain more A and T nucleotides, frameshifts, gaps and stop codons. In contrast, intragenic L1s contain higher G-C contents and intactness score (Fig. 1E and Supporting Table S2). In conclusion, intragenic L1 sequences have been conserved across evolutionary time with respect to transcriptional activity and CpG dinucleotide sites for mammalian DNA methylation. These findings implied physiological functions of intragenic L1 methylation.

### Intragenic L1s repress genes in cancer cells

**Figure 2:**
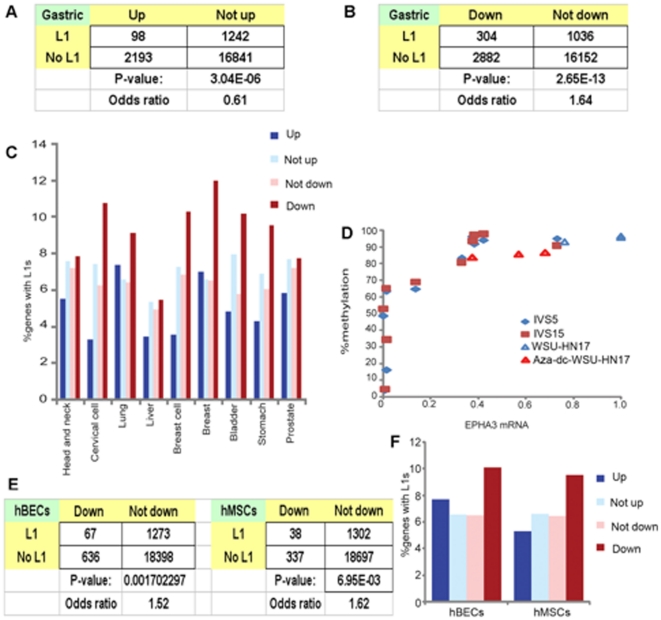
Intragenic L1s repress gene expression in cancer in the same pattern as demethylated normal cells. A) and B) are chi-square 2×2 tables, p values and odds ratios, comparing proportions of gastric cancer genes possessing L1s between up- (“Up”) or down- (“Down”) regulated and not up- or not down-regulated groups, respectively. C) Percentages of L1-containing mRNAs that are up- or down-regulated in various cancer types. D) EPHA3 mRNA and L1-EPHA3-IVS5 and IVS15 methylation levels in WSU-HN cells and Aza-dC-treated WSU-HN17s. E) Two chi-square 2×2 tables, p values and odds ratios, comparing proportions of Aza-dC-treated hBECs or hMSCs genes possessing L1s between down-regulated (“Down”) and not down-regulated groups, respectively. F) % of mRNAs from genes containing L1s in Aza-dC-treated hBECs and hMSCs. “Up” and “Down” indicate increased and decreased expression, respectively. GSE records, GSM samples and type of *t*-test and 2×2 tables of chi-square tests are provided in Supporting Table S3.

L1s are hypomethylated in many cancers [1]. To investigate whether intragenic L1s control host genes in L1 hypomethylated cancer cells, we compared gene expression in cancer between genes possessing intragenic L1s and the rest. Genes possessing L1s were determined by L1base [17] and expression microarray data is publicly available data from the GEO [35], [36]. Each gene was classified by 2 student t-tests, up- and down-regulations. If the mean of cancer group was statistically higher or lower than normal group, the gene was classified as up- or down-regulated, respectively. If t-test was not statistically significant, the gene was classified as not up- or not down-regulated, respectively. The distribution of genes possessing L1s which showed the increased expression in cancer was compared with the rest of gene set by chi-square tests (Fig. 2A). The same analysis was performed for genes with decreased expression in cancer (Fig. 2B). Figure 2A and 2B demonstrated examples of chi-square tests of gene expression in gastric cancer. Genes possessing intragenic L1s were found less likely to be up-regulated (odds ratio (OR) = 0.61, p = 3.04E−06) (Fig. 2A). Moreover, expression of genes containing L1s were more commonly decreased (OR = 1.64, p = 2.66E−13) (Fig. 2B). Intragenic L1s may control hundreds of genes. Among 1,340 genes containing L1s, 1,242 genes were not up-regulated and 304 genes were down-regulated (Fig. 2A and 2B). Analysis of a number of expression arrays showed similar results. We tested head and neck squamous cell carcinoma, cervical cancer cells, lung adrenocarcinoma cells, liver cancer, breast cancer cells, ductal and lobular breast cancer, bladder carcinoma situ, microsatellite instable gastric cancer, metastasis prostate cancer. We found that genes down-regulated in cervical cancer cells, lung adrenocarcinoma cells, breast cancer cells, ductal and lobular breast cancer, bladder carcinoma situ, microsatellite instable gastric cancer are more likely to contain L1s. Moreover, genes with higher expression levels in head and neck squamous cell carcinoma, cervical cancer cells, liver cancer, breast cancer cells, bladder carcinoma situ, microsatellite instable gastric cancer, metastasis prostate cancer are less likely to possess L1s (Supporting Table S3 and Fig. 2C). Therefore, intragenic L1s may repress host genes in these cancers. Although in vitro insertion of active L1 sequences into host gene introns disrupted gene expression [14], most mRNA levels from genes containing L1 were not absent (Supporting Fig. S1). Moreover, a comparison of genes in cancer, as described in supporting table S3.1–S3.9, showed that the probability of genes containing L1 to be commonly down-regulated in independent experiments is higher than genes without L1(p-value  = 7.99E-08, mean L1 = 1.6642, mean no L1 = 1.4861) (Supporting Fig. S2). These analyses supported biological significance of intragenic L1s in gene regulation in cancer.

To demonstrate the relation pattern between L1 methylation levels and gene expression, we measured intragenic L1 methylation levels and host gene's mRNA level. Previously, we evaluated methylation levels of 17 intragenic L1 loci and found that the L1 methylation levels of L1-EPHA3-IVS5 and L1-EPHA3-IVS15 are strongly correlated in cancer cells, suggesting locus specific mechanism [18]. Measurement of intragenic L1-EPHA3 methylation and EPHA3 mRNA levels in head and neck squamous cell cancer (HNSCC) cell lines (WSU-HNs) revealed that lower levels of intragenic L1-EPHA3-IVS5 and L1-EPHA3-IVS15 methylation correlated with lower EPHA3 mRNA levels (Pearson r = 0.7961 and 0.7638, respectively; Fig. 2D). EPHA3 mRNA in WSU-HN17 cells was also significantly lower when L1-EPHA3 was hypomethylated (paired *t*-test; p<0.001; Fig. 2D). Therefore, the level of mRNA can be directly correlated with intragenic L1 methylation.

### Loss of methylation in normal cell represses genes that harbor L1s

**Figure 3:**
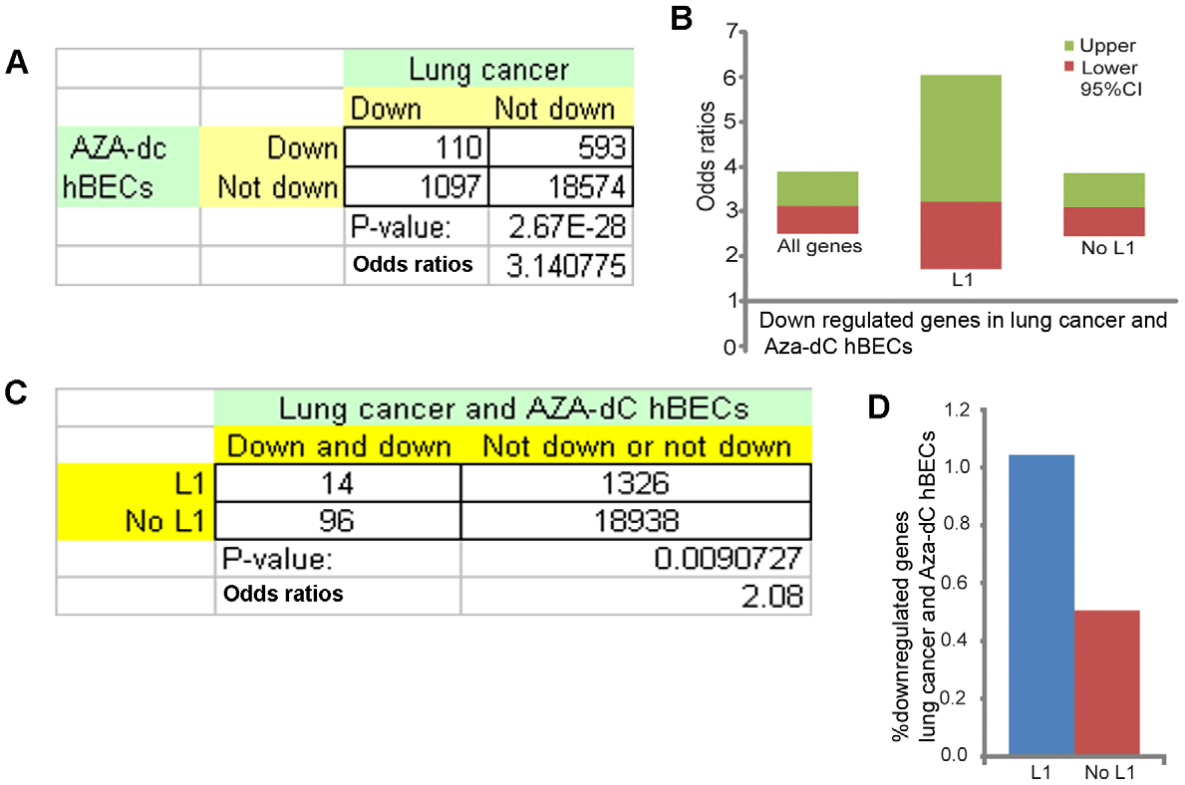
Demethylated genome and hypomethylated intragenic L1s repress gene expression in cancer. A) 2×2 tables, p values and odds ratios and B) odds ratio for mRNAs that are under-expressed (“Down”) in lung cancer and Aza-dC-treated hBEC cells compared to non-Aza-dC-treated hBEC cells for (A and B) all genes, (B) genes with L1s (L1) and genes without L1s (No L1). B) The middle, top and bottom lines of each two-color box are the odds ratio and upper and lower 95% confidence interval (CI), respectively. C) 2×2 tables, p values and odds ratios and D) percentages of mRNAs that are under-expressed (“Down”) in both Aza-dC-treated cells and cancer cells compared with genes that are not downregulated in Aza-dC-treated cells or cancer, for genes with and without L1s (L1 and No L1). The corresponding 2×2 contingency tables for A) and B), and C) and D) are provided in Supporting Table S4, and S5, respectively.

An analysis of gene expression in human bronchial epithelial cells (hBECs) and human mesenchymal stem cells (hMSCs) after genome wide demethylation by 5-aza-2′-deoxycytidine (Aza-dC) treatment demonstrated a greater prevalence of intragenic L1s in down-regulated genes (OR = 1.52, p = 0.0017 and OR = 1.62, p = 0.0069, respectively) (Fig. 2E, 2F and Supporting Table S3), interestingly, a similar pattern as found in cancer (Fig. 2C). We further explored if genome wide hypomethylation regulated genes in cancer. We performed a chi-square test to determine the significance of overlap between down-regulated genes in demethylated hBECs and in lung cancer. Genes which were down-regulated in Aza-dC treatment on hBECs were found to preferentially have lower mRNA levels in the cancerous cells of the lung (p = 2.67E−28; OR = 3.14; 95% CI = 2.54−3.88; Figure 3A and 3B and Supporting Table S4). This supports the hypothesis that hypomethylation down regulates genes in cancer.

Interestingly, hypomethylation down regulates both groups of genes, with L1 (p<2.59E−04; OR = 3.24; 95% CI = 1.73−6.05; Supporting Table S4) and without L1 (p<2.34E−24; OR = 3.09; 95% CI = 2.46−3.87; Supporting Table S4). Therefore, it is possible that in addition to L1 there are other DNA methylated gene body elements that regulated gene expression. This hypothesis is supported by a recent report that, in gene body, unique methylated sequences are more prevalence in highly expressed genes [9]. To further differentiate the role of L1s, we compared between genes with and without L1s. We found that the event of down-regulation of genes in both Aza-dC treated hBECs and lung cancer is more prevalent in genes containing L1s than in genes without L1. (OR = 2.08, p = 0.009; Fig. 3C and 3D and Supporting Table S5). Therefore, hypomethylation decreases the expression of many genes in cancer, and intragenic L1s act as a methylation-mediated *cis*-regulatory element.

Many genes frequently downregulated in cancer display hypermethylated promoters [8]. However, we found no connection between promoter hypermethylation in cancer and the presence of intragenic L1s. Genes with hypermethylated promoter have been shown to be up-regulated when cells were demethylated. The expression of genes with L1 was not frequently increased when cancer cells were demethylated (Supporting Table S6.1 and S6.2).

### L1 hypomethylation increases L1 RNA levels

**Figure 4:**
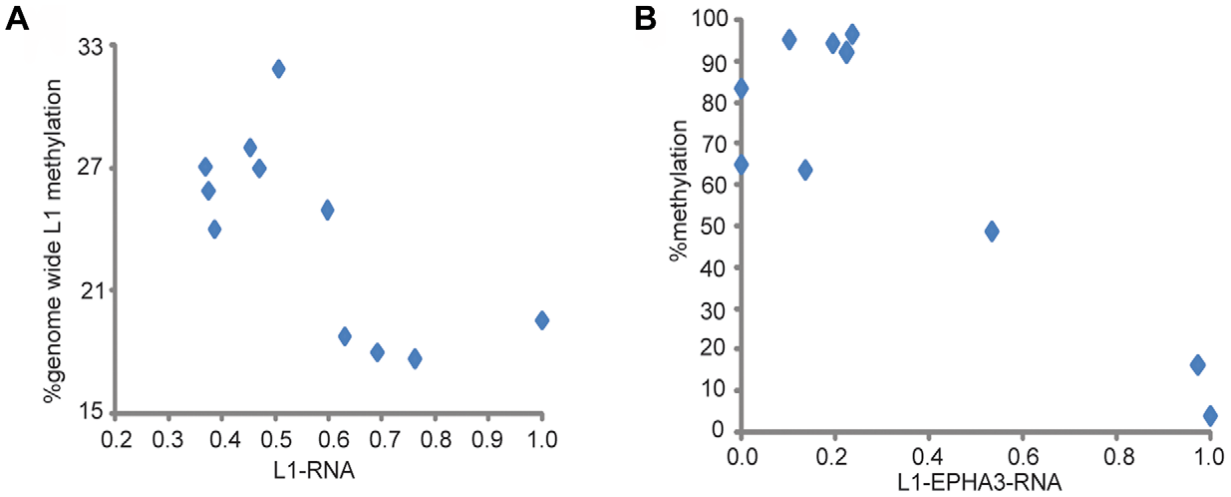
L1 hypomethylation increases L1 RNA. A) Genome wide L1 methylation and L1 RNA levels in WSU-HN cells. B) L1-EPHA3 methylation and L1-EPHA3 RNA levels.

Measurement of methylation and RNA levels showed an inverse correlation between genome wide L1 methylation and L1 RNA (Pearson r = −0.6955; Fig. 4A). This finding supports the hypothesis that L1 hypomethylation increases L1 RNA transcription [37]. Intronic genes have been proposed to form aberrant RNA complexes with host genes and consequently inactivate host gene transcription [38]. L1s are retrotransposable elements that may still possess transcriptional activity at a significant number of loci [29]. Moreover, some L1s are transcribed beyond their polyA addition sites and consequently produce chimeric RNAs that include both L1 and unique intronic sequences [39]. We screened for and found L1-EPHA3 RNA from intron 15 of the EPHA3 gene and observed a significant inverse association between L1-EPHA3 RNA and L1-EPHA3 methylation (Pearson r = −0.8686; Fig. 4B). Therefore, L1 hypomethylation leads to increased L1 transcription and consequently produces more intronic L1 RNA.

### Intragenic LINE-1 elements repress transcription in cancer cells through AGO2

Retrotransposon RNAs or transcripts forming dsRNA structures trigger RISC assembly [40]. After binding small interfering RNAs (siRNA), RISC recognizes and degrades complementary RNA molecules [41]. L1s possess up to three internal promoters, at the 5′ and 3′ ends and in the 5′ antisense direction [42], [43], [44]. The sense and antisense promoters in the 5′ UTR produce bidirectional transcripts that are subsequently processed into siRNAs to prevent retrotransposition [40]. Therefore, we hypothesized that L1 RNA and intronic pre-mRNA of genes containing L1s, because of complementarity of both sequences, form dsRNA which may be targeted by RISC, consequently depleting the amount of mRNA derived from genes containing L1s. The RISC complex is composed of Dicer, Argonaute and siRNA. A similar complex with AGO2 acts to silence gene transcription in the nucleus [45], [46].

**Figure 5:**
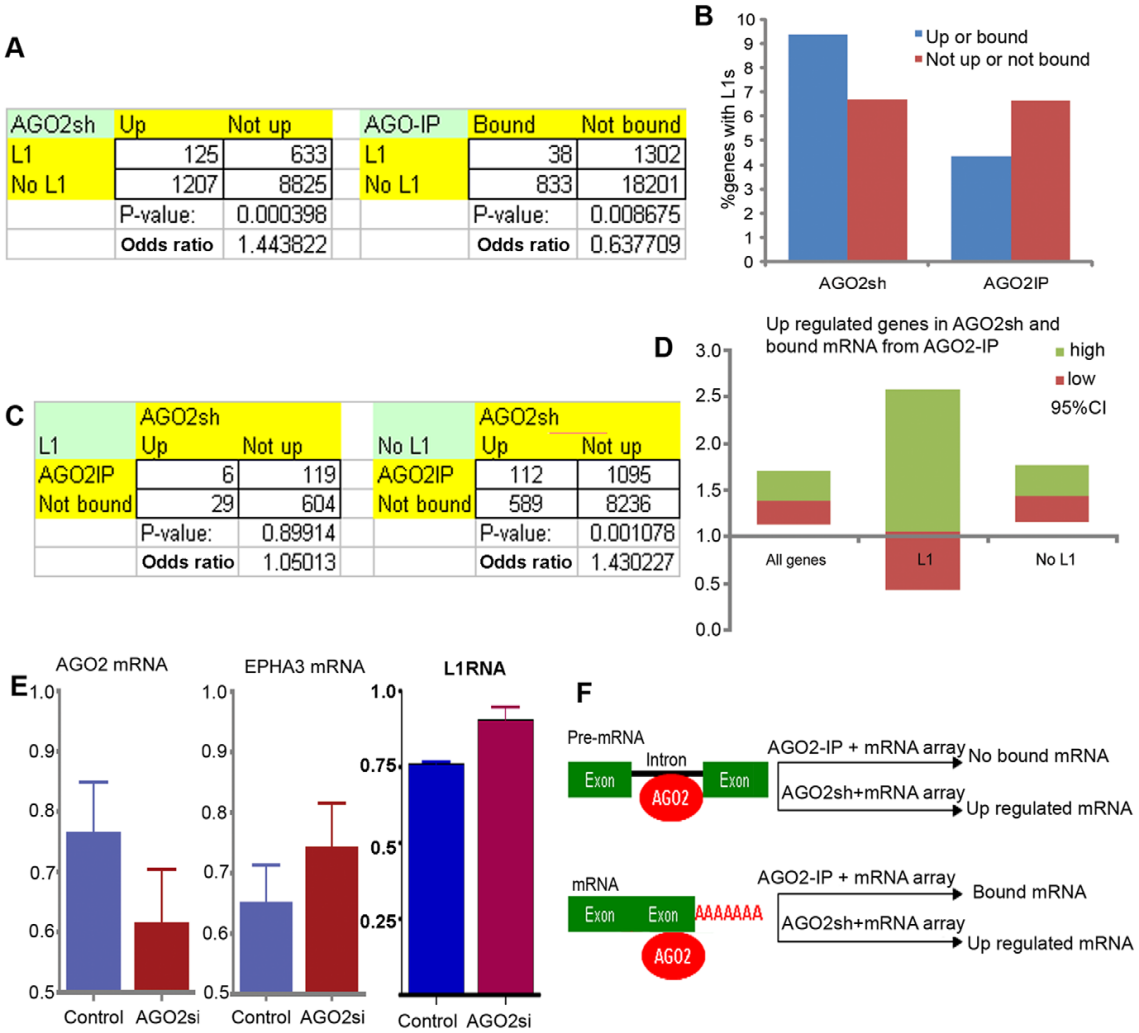
AGO2 and L1 RNA mediate intragenic L1 down-regulated gene expression in gene containing L1s. A) 2×2 tables, p values and odds ratios and B) percentages of L1 containing-genes that exhibit increased mRNA levels in AGO2sh-treated cells (“Up”) or are bound by AGO2 in AGO2IP (“Bound”), respectively. C) 2×2 tables, p values and odds ratios and D) odds ratios (95% CI) comparing HEK293T mRNA between up-regulated genes and not up-regulated genes upon AGO2sh and bound by AGO2 proteins for all genes (D), genes with L1s (L1) and genes without L1s (No L1), (C and D). D) The middle, top and bottom lines of each two-color box are the odds ratios and upper and lower 95% CIs, respectively. E) Levels of AGO2, EPHA3 mRNA and L1RNA in WSU-HN17 AGO2si cells. Data represent means ± SEM. GSE records, GSM samples, type of *t*-test for A) and B) are given in Supporting Table S3. 2×2 contingency tables for C) and D) are given in Supporting Table S4. F) Two scenarios of AGO2-target binding reflecting in different mRNA array results when using AGO2-IP and AGO2sh as probes. Negative result was expected from mRNA expression microarray using AGO2-IP RNA as probes, (“AGO2-IP + mRNA array”), when introns of pre-mRNA were targeted. However, positive result was expected from mRNA expression microarray using mRNA from AGO2sh as probes, (“AGO2sh + mRNA array”), regardless AGO2 targets pre-mRNA or mRNA.

If intragenic L1 RNA reduces host gene mRNA via AGO2, AGO2 protein deprivation will result to increase mRNA levels of genes hosting L1s. Analysis of mRNA microarray of AGO2 down-regulated cells, AGO2sh [47], demonstrated that the limited expression of AGO2 in a human embryonic kidney cell line (HEK293T) resulted in an expression pattern of gene containing L1s that was opposite from that observed during L1 hypomethylation; namely, they were more likely to be up-regulated (OR = 1.44, p = 0.0004; Fig. 5A and 5B and Supporting Table S3). The shRNAs of DICER1, AGO1, AGO3 and AGO4 did not upregulate genes with L1 (Supporting Table S6.3−S6.7). This suggested that AGO2 preferentially limits the concentration of mRNAs derived from genes containing L1s. We further evaluated an mRNA microarray experiment hybridized by AGO2 precipitated RNA [48] and found that AGO2 may not directly degrade mRNAs that are derived from genes containing L1s. Although RISC binds and degrades mRNA, mRNAs derived from genes containing L1s were less likely to be bound by AGO2 (OR = 0.64, p = 0.009; Fig. 5A and 5B and Supporting Table S3), which was initially surprising given the results of the AGO2sh experiments (Fig. 5A and 5B and Supporting Table S3).

When comparing AGO2-bound mRNAs to AGO2sh-upregulated genes, we found that for mRNAs derived from genes without L1, AGO2 significantly bound to mRNAs that were enriched when cells were treated with AGO2sh (OR = 1.43, p = 0.001; Fig. 5C and 5D and Supporting Table S4). This confirmed that AGO2 targets and degrades these mRNAs. In contrast, the mRNAs of genes containing L1s do not bind significantly to AGO2 even if they are up-regulated by AGO2sh (OR = 1.05, p = 0.90; Fig. 5C and 5D and Supporting Table S4). The down-regulation of AGO2 also increased EPHA3 mRNA and L1RNA levels in WSU-HN17 cells (p<0.01; Fig. 5E). Significant alteration of L1 methylation was not observed by AGO2sh (p = 0.942). Therefore, even though AGO2 preferentially down-regulates genes containing L1s, it does not do so by binding to their mRNAs. Because most intragenic L1s are located in introns, pre-mRNAs, particularly intronic sequences, are the preferable AGO2 targets (Fig. 5F).

**Figure 6:**
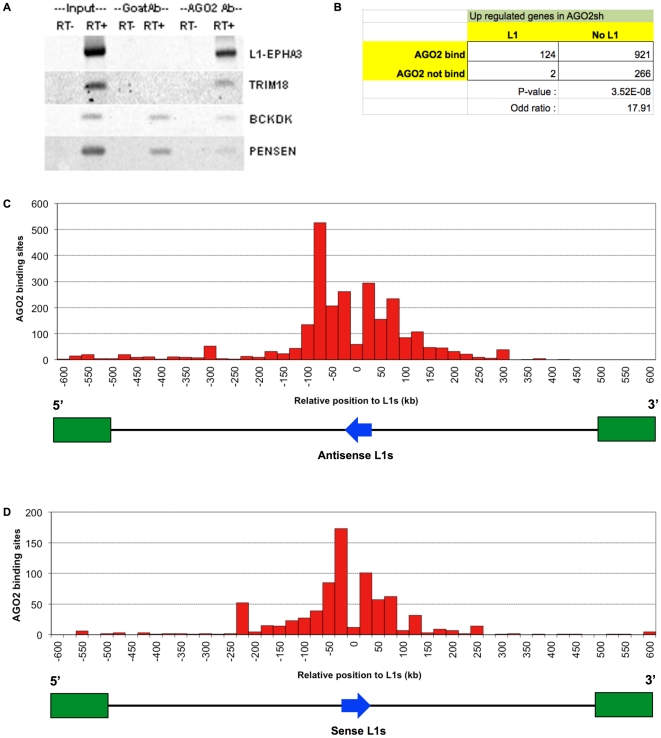
AGO2 binds intragenic L1 RNA and targets AGO2 regulated genes. A) RNA immunoprecipitation/RT-PCR. TRIM38 is a miRNA binding site in intron of TRIM38. BCKDK and PSENEN are introns of the genes, lacking of miRNA binding site. RT- and RT+ are samples without and with reverse transcriptase treatment, respectively. B) 2×2 contingency table, p-values and odds ratios of chi-square test and C) grouped frequency distribution (histogram) of AGO2 binding sites corresponding with the location of antisense L1 with a 25-kb interval. and D) histogram of AGO2 binding sites corresponding with the location of sense L1 with a 25-kb interval.

**Figure 7:**
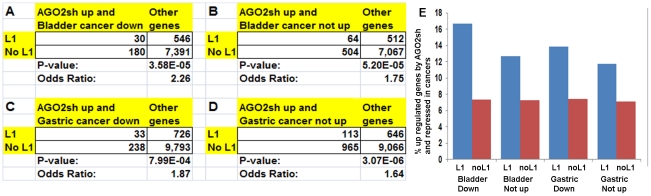
AGO2 repress expression of gene containing L1 in cancer. The Chi-square test shows that the up-regulated genes in AGO2si and the down-regulated (or not up) genes in A and B) bladder carcinoma and C and D) gastric carcinoma are associated with L1. The test and control are of the same as in the Supporting Table S3.12, S3.7 and S3.8. AGO2 represses expression of gene containing L1 in cancer. The chi-square test shows that the up-regulated genes in AGO2si and the down-regulated (or not up) genes in A) and B) bladder carcinoma and C) and D) gastric carcinoma are associated with L1. The test and control datasets are the same as present in the Supporting Table S3.12, S3.7 and S3.8. E) The percentage of genes that was both up-regulated by AGO2sh and repressed in cancer cells.

Here we provided an example of AGO2 binding with L1-associated pre-mRNA. RNA immunoprecipitation and RT-PCR confirmed that AGO2 binds to L1-EPHA3 RNA (Fig. 6A). We used the information of genes containing L1 from L1base to identify the up-regulated genes in AGO2 depleted HEK293T cell lines, which have L1 in the gene body. Using this set of up-regulated genes, Figure 6B shows the 2x2 contingency table displaying a chi-square test of association between the presence of L1 and AGO2 binding sites. AGO2 binding sites were found in all L1-containing genes that were up-regulated in AGO2sh cells (124 out of 126 genes, OR = 17.91, p = 3.52E−08). Focusing on the up-regulated genes, we also observed the distribution of AGO2 binding sites on these genes in which L1 can be found in their intronic regions. Numbers of AGO2 binding sites were counted if they are found in the vicinity of L1 assuming that L1 is located between any exons. Particularly, we created a histogram by counting the number of AGO2 binding sites located within 600 kb upstream and downstream of L1 using the 25-kb interval size (Fig. 6C and 6D). Figure 6C and Figure 6D demonstrate the frequency distribution of AGO2 binding sites with respect to antisense L1 and sense L1, respectively. Interestingly, hundreds of AGO2 binding sites were found at hypothetical locations presenting double strand RNA between pre-mRNA and 5′ or 3′ L1 transduction sequence (Fig. 6C and 6D). These were sequences nearby L1 at 5′ direction from L1 toward gene transcriptional start sites. Therefore, AGO2 preferentially regulates genes containing L1s by targeting intragenic L1 RNAs with sequence complementary to pre-mRNAs. Finally, the event of up-regulation of genes in AGO2 depleted cells and down- or not up-regulated in cancer was found more prevalent in genes containing L1s than in genes without L1 (Fig. 7A–7E). Therefore, intragenic L1s act as a AGO2-mediated *cis*-regulatory element in cancer.

Finally, we evaluated the correlation between expression changes and the presence of intergenic L1s. We compared genes with sense and antisense intergenic L1 within 1 or 2 kb from 5′ and 3′ of the genes (Supporting Table S7). Unlike genes containing L1s, there are a limited number of genes with nearby intergenic L1s. Interestingly, L1 within 1 kb from the end of the genes prevented genes from up regulation (OR = 0.23, p = 0.03; Supporting Table S7). In contrast, there was no regulatory evidence of L1 at 5′ end of genes (Supporting Table S7). This data supported a possibility that L1 transcript in opposite direction can repress the nearby genes.

## Discussion

Our comprehensive analysis of genome and expression array databases is a simple and useful approach to explore disease- or biological process-related mechanisms that alter genome wide gene expression patterns. Here, we showed that genes hosting intragenic L1s are more likely to be repressed in cancer and that the level of repression depends on the degree of L1 hypomethylation. The degree of L1 hypomethylation varies at each locus of each tumor and may change throughout the multistage carcinogenesis process. In general, more advanced stages of cancer are associated with a greater degree of hypomethylation [1], [20], [21], [22], [23], [24]. Therefore, L1s may promote cancer progression in part due to increasing degrees of gene repression and numbers of repressed genes.

It is important to note that the conclusion of our comprehensive analysis between L1 locations and gene expression should not be altered by L1 insertion dimorphisms (LIDs) of analyzed samples. L1s are still active retrotransposons and new insertion can be identified as LIDs [50], [51], [52]. Nevertheless, majority if LIDs are truncated and localized intergenics [50], [51], [52]. Therefore, the number of intragenic long LINE-1 dimorphisms are low. Moreover, L1Base reported L1 locations based on *Homo sapiens* Genome: Statistics — Build 36 version 1. This suggests that most L1s in L1Base are not newly inserted L1s and very few may represent common LIDs. Consequently, the degree that LIDs influence the complementary analysis was very low.

Many studies have reported the methylation of tumor suppressor gene promoters in cancer cells; this epigenetic regulation has become a potential candidate for biomarker and therapeutic target development. To our knowledge, this is the first study to demonstrate that global hypomethylation down-regulates genes in cancer. Moreover, there may be several hypomethylation-mediated cis-suppressor elements, including intragenic L1s. Genome wide hypomethylation is common to many cancer types [1]. Therefore, the hypomethylation sites and repressed genes described here represent a vast number of molecular targets and diagnostic markers.

Nevertheless, not all intragenic L1s can repress gene expression in cancer, and L1s may regulate genes through several distinct mechanisms. Even though L1 sequence analysis showed that intragenic L1s have been conserved throughout human evolution, their sequences and distributions do vary considerably. Moreover, the methylation levels of some L1 loci are independent of genome wide L1 hypomethylation in cancer [18]. Notably, Rangnawa and colleagues [39] reported varying L1 RNA levels in normal cells that generally feature a limited range of intronic L1 methylation [18], suggesting that other factors also influence L1 expression. Therefore, it is complicated but important to further explore L1 and genome characteristics that may determine their repression properties in cancer cells.

Here, we identified one mechanism by which L1s can repress genes in cancer. First, L1 hypomethylation increases L1 RNA levels. Then, the AGO2 protein regulates genes possessing L1. Finally, mRNA processing of genes harboring hypomethylated L1s is disrupted. AGO2 was reported to commonly target L1 RNA [40] and was proposed to prevent retrotransposition events [53]. However, the role of the L1-RNA-AGO2 complexes derived from the majority of retrotranspositionally incompetent elements was unknown. Moreover, there are several mechanisms by which RISC can regulate gene expression [54]. This study also proposed a new role for nuclear RISC complexes.

This study proved that intragenic L1 hypomethylation represses genes via a post-transcriptionally mechanism, based on siRNA and AGO2. However, it is possible that there are other mechanisms that should be further explored such as the intereference with the elongating RNA Pol2 transcribing their host genes or formation of chromatin complex in relation with L1 methylation level.

In conclusion, intragenic L1 produces L1 RNA upon hypomethylation in cancer tissues, and the host gene is consequently down-regulated when AGO2 is present. Further studies should reveal additional factors that influence this process both in *cis* and in *trans*, such as L1 sequence variations, boundary sequences, methylation, chromatin configuration, location, transcription factors that are correlated with the degree of L1 methylation, molecules involved in the AGO2-intronic L1 RNA processing mechanism and factors that guide or prevent AGO2 recognition. In addition to their modifications in cancer, L1 and other IRS methylation are altered by many biological processes including the disease-related ones [1], [18], [25], [26], [29], [30], [34], [55]. Therefore, it will be interesting to explore whether changes in L1 and other IRS methylation also regulate genes under these conditions.

## Materials and Methods

### Statistical analysis of L1 characters

L1s reported in L1base [17] were categorized according to their genomic locations as “intragenic” or “intergenic” based on NCBI Reference Sequence (RefSeq) annotation. The differences in structural characteristics between L1 groups were analyzed using the chi-square test and homoscedastic *t*-test for categorical and non-categorical functionally important features, respectively. For categorical features, the frequency of intragenic L1 features was counted both according to the number of genes and the number of L1 sequences that contained the tested features.

### Classification of mRNA

mRNAs from GEO [35], [36] were classified as up- or down-regulated and not up- or not down-regulated depending on the statistical significance determined by student's t-test. The libraries were GSE6631[56], GSE9750[57], GSE5816[58], GSE14811[59], GSE1299[60], GSE5764[61], GSE3167[62], GSE13911[63], GSE6919[64], GSE9764[65], GSE4246[47], GSE14537[48] and GSE14054[49]. A Student's *t*-test was performed on all probes. Some probes represented more than one gene (homologous probes). A gene was counted as differentially expressed (up- or down-regulated) by expression level of at least one unique probe. If a gene contained only homologous probes, there must be at least two homologous probes representing the same gene. Up- or down-regulated genes were counted when representing probes were significantly different between test and control groups at p<0.01. P<0.05 was used when the number of tests or controls was two or mRNA was prepared by immunoprecipitation.

### Expression of genes possessing internal L1s

To evaluate if intragenic L1s can influence host gene expression, up- or down-regulated genes and genes without significantly increased- or decreased- expression were divided into two categories whether containing intragenic L1s or not. Genes hosting L1s were listed in Supporting Table S1. The numbers of genes in each subset were compared using a chi-square test (Table 1).

**Table 1:** 2×2 table of chi-square test to evaluate if intragenic L1s can influence host gene expression.

	Up- or down-regulated genes	Not up- or not down-regulated genes
Genes containing L1s	Number of regulated genes containing L1s	Number of not regulated genes containing L1s
Genes without intragenic L1s	Number of regulated genes without intragenic L1s	Number of not regulated genes without intragenic L1s

### Connection Up- or Down- Regulation Expression Analysis of Microarrays (CU-DREAM)

CU-DREAM is a method for analyzing databases of two expression arrays with different conditions to determine if the two conditions share genome wide gene regulation pathway. First, mRNAs from two different expression microarrays were classified as up- or down-regulated and not up- or not down-regulated depending on the statistical significance determined by student's t-test. From data of two arrays, each mRNA presented in both experiments were classified into 4 groups: 1) regulated in both array experiments, 2) not regulated only in the first experiment, 3) not regulated only in the second experiment, and 4) not regulated in both array experiments. The numbers of genes in each subset were compared using a chi-square test (Table 2). Non random distribution of these four groups indicated the connection of the two variables if the two experiments promoted or inhibited the same mechanism(s) that altered genome wide gene expression.

**Table 2:** CU-DREAM 2×2 table of chi-square test.

	Up- or down-regulated genes of experiment A	Not up- or not down-regulated genes of experiment A
Up- or down-regulated genes of experiment B	Number of genes in the 1^st^ group	Number of genes in the 2^nd^ group
Not up- or not down-regulated genes of experiment B	Number of genes in the 3^rd^ group	Number of genes in the 4^th^ group

### Cell preparation

Eleven HNSCC cell lines (WSU-HNs), including WSU-HN 4, 6, 8, 12, 13, 17, 19, 22, 26, 30 and 31, were provided by Dr. Silvio Gutkind (NIH, USA). Cells were cultured in Dulbecco's modified Eagle's medium (DMEM; Gibco BRL, Life Technologies, Pairly, UK) supplemented with 10% heat-inactivated fetal bovine serum (FBS; Sigma, St. Louis, MO, USA). Cells were incubated at 37°C in 5% CO_2_. To inhibit DNA methyltransferase (DNMT) activity, WSU-HN cells were supplemented with 4 µM 5-aza-2-deoxycytidine (Cat.No. A3656 Sigma-Aldrich) every 24 hours for up to 16 days for genomic demethylation. WSU-HN17 cells were transiently transfected with siRNA against eIF2C2 mRNA (AGO2 siRNA), which was designed by and purchased from Santa Cruz Biotechnology, Inc. (eIF2C2 siRNA (h): sc-44409). A non-silencing siRNA with no homology to any known mammalian genes (AllStars negative control siRNA, QIAGEN, Basel, Switzerland) was transiently transfected as a negative control siRNA for each experiment.

### L1 methylation analysis

The methods of genome wide L1 and specific loci L1 methylation measurements were extensively validated [1], [18], [34]. Briefly, genomic DNA was denatured in 0.22 M NaOH at 37°C for 10 min. A 30 µl aliquot of 10 mM hydroquinone and 520 µl 3M sodium bisulfite were added and the DNA was further incubated for 16–20 hrs at 50°C. The DNA was purified and incubated in 0.33 M NaOH at 25°C for 5 min, ethanol-precipitated, then washed with 70% ethanol and re-suspended in 20 µl TE buffer. A 2 µl sample of bisulfited DNA was subjected to 35 cycles of PCR with two primers as reported [1], [18] at an annealing temperature of 53°C. The amplicons were digested in 30 µl reaction volumes with 2U of *TaqI* or 8U of *TasI* in 1x*TaqI* buffer (MBI Fermentas) at 65°C overnight and then electrophoresed in 8% non-denaturing polyacrylamide gels. The intensities of DNA fragments were measured with a PhosphorImager and analyzed using ImageQuant software (Molecular Dynamics). The methylated amplicons (*Taq*I positive) yielded 80 bp DNA fragments while unmethylated amplicons (*Tas*I positive) yielded 97 bp fragments. The L1 methylation level was calculated as a percentage (the intensity of methylated L1 digested by *TaqI* divided by the sum of the unmethylated L1 digested by *TasI*-and the *TaqI*-positive amplicons). The same set of DNAs was applied as a positive control in each set of COBRA experiments.

### Reverse transcription (RT) PCR

Total RNA was extracted from cell lines using the Trizol reagent (Life Technologies, Inc.) according to the manufacturer's instructions. The RNA was treated with RNase-free DNaseI(Fermentas) to remove contaminating genomic DNA and with RiboLock™ Ribonuclease Inhibitor (Fermentas) to prevent degradation. To synthesize cDNA, 5 µg DNA-free RNA was dissolved in 12 µl of DEPC-treated water containing 0.5 µg oligo(dT)18 primer (Fermentas). The RNA was incubated for 5 min at 70°C and chilled on ice for 5 min. Each sample was then incubated with 200U RevertAid™ M-MuLV Reverse Transcriptase (Fermentas), 20 U Ribolock™ Ribonuclease inhibitor (Fermentas) and 20 mM dNTPs for 1 hr at 42°C, followed by 10 min at 70°C and subsequent chilling on ice. cDNA was amplified using the exon primers listed in the Appendix. RNA that had not been reverse transcribed was included as negative control to evaluate the amount of LINE-1 DNA contamination. Oligo(dT)18, random Hexamer and L1-EPHA3-IVS15-P1, ACAAATACCATATCCTTCAAGACAAATCG, were used for the RT step. The PCR oligonucleotides used were: L1-RNA, CAGGAAGGGGAATATCACACTC and TGCGCTGCACCCACTAACTC; and 5′L1-RNA, GGCCAGTGTGTGTGCGCACCG and CCAGGTGTGGGATATAGTCTCGTGG; AGO2, CACAAGTTGGTTCTGCGCTA and TGAAACTTGCACTTCGCATC; GAPDH, TTCGCTCTCTGCTCCTCCTGTTC and CTGGTGACCAGGCGCCCAA; L1-EPHA3 RNA, CTAACCTGCACAATGTGCACATGTACCC and L1-EPHA3-IVS15F. For RNA immunoprecipitation experiment control primers were, TRIM38, GCAAAAACCACAATTACTTTTGCAC and AAGAGAGAAAATTGGTAATCAGCTTG; negative control, PSENEN, GGCACCCCAGCCGGAGGA and CGGGTCGTCCCAAGGGTCTG; and BCKDK, CCCACCATGATGCTCTACGCTGG and CCTTGATGCGGTGAGCAATCCTC. Real-time RT-PCR was performed for 40 cycles with an annealing temperature of 60°C. Real-time RT-PCR was performed in a Light Cycler machine (Roche Molecular Biochemicals, Indianapolis, IN, USA) using QuantiTect SYBR Green I (Qiagen, Hilden, Germany) according to the manufacturer's instructions. To quantitate gene expression, each PCR product was cloned into the pGEM-T easy vector (Promega, Santhan, UK) and used as controls. All mRNA, L1 RNA and L1-EPHA3 RNA levels were normalized with GAPDH, total RNA and band intensity, respectively. The highest RT-PCR levels observed in each experiment were adjusted to 1.

### RNA immunoprecipitation

AGO2 antibody (sc-32659) and goat IgG (sc-2028) (Santa Cruz) were used to immunoprecipitate RNA as described (http://www.epigenome-noe.net/researchtools/protocol.php?protid=28) [66]. Cells were grown in a 75 cm^2^ flask at 80% confluence, washed with PBS and trypsinized. Approximately, 1×10^8^ cells were added to a 15 ml conical tube, pelleted, and resuspended in 10 ml 1% formaldehyde in PBS. This crosslinking reaction was performed for 30 minutes at room temperature and stopped by the addition of glycine at a final concentration of 125 mM. The pellet was washed twice with ice-cold PBS containing 1× protease inhibitor cocktail. The cell pellet was resuspended in 200 µl Buffer A (5 mM PIPES (pH 8.0), 85 mM KCl, 0.5% NP40, 1× Roche protease inhibitors cocktail, SUPERase•in (50 U/ml)) and placed on ice for 10 minutes. The crude nuclei fraction was pelleted by microcentrifugation at 5000 rpm for 5 minutes at 4°C. The pellet was washed once in Buffer A without NP-40, then resuspended in 500 µl Buffer B (1% SDS, 10 mM EDTA, 50 mM Tris-HCl pH (8.1), 1× Roche protease inhibitors cocktail, SUPERase in (50 U/ml)) and incubated on ice for 10 minutes. Lysates were sonicated three times at 4°C using a Branson Sonifier at constant power, output = 70%, and continuous sonication for 20 seconds. After sonication, insoluble elements were cleared by microcentrifugation at 14,000 rpm for 10 minutes at 4°C. The sonicate was diluted 10-fold with IP Buffer to a final volume of 1 ml per immunoprecipitation reaction. A 1% aliquot was preserved as an input sample and frozen at −80°C until the reverse crosslinking step. For the precipitation step, 5 µg of primary antibody or a normal IgG control was added to each tube. Immune complexes were allowed to form by slow mixing on a rotating platform at 4°C overnight. To collect immune complexes, 50 µl Protein A/G Agarose-PLUS (Santa Cruz) was added to each tube and slow mixing rotation was continued for 2 hours. Immune complexes were pulled down by gentle centrifugation at 1000 rpm for 2 minutes at 4°C. Each immune complex was washed five times (1 ml wash, 5 minutes each). After each wash (low salt wash, high salt wash, LiCl wash and 2 washes with TE pH 8.0), complexes were pelleted by gentle centrifugation (1000 rpm, 1 minute) and the wash buffer was aspirated using a clean pipette tip. Immune complexes were eluted by the addition of 250 µl Elution Buffer and collected by centrifugation (8000 rpm, 2 minutes). NaCl was added to a final concentration of 200 mM (including the input samples) and placed at 65°C for at least 2 hours to reverse crosslinking. Samples were subjected to Trizol LS reagent extraction and resuspended in 20 µl DEPC-treated water. DNA was removed from the samples by treatment with RNase-free DNaseI (Fermentas Inc.). TRIM38 was predicted to be positive. BCKDK and PSENEN were predicted as negative controls. TRIM38 was up-regulated in AGO2si experiment and possess a intronic miRNA binding site [67]. BCKDK and PSENEN do not contain miRNA binding site [67].

### Localization of AGO2 target pre mRNA

The chromosomal locations of AGO2 binding sites were retrieved from the CLIPZ database [68], which releases RNA-binding protein (RBP) binding site data generated by cross-linking and immunoprecipitation (CLIP) mapping technique. Only AGO2 binding sites longer than 18 base pairs were included in our study. We mapped the locations of AGO2 binding sites to human genome reference sequence hg18 (build 36.3) and then identified NCBI RefSeq target genes of AGO2. The list of genes that contain AGO2 binding sites and also contain L1 were obtained from intersecting the set of genes that has at least one AGO2 target site with the set of L1-associated genes inferred from L1base. The positions of AGO2 binding sites in relative to L1 sequences were also calculated. To determine if AGO2 works in concert with L1 in the regulation of gene expression, the microarray data of AGO2 knock down HEK293T-derived cell lines and control group [47] were used for the analysis. These expression data were obtained from the experiment GSE4246 deposited in the Gene Expression Omnibus (GEO) database [35], [36]. Paired t-test with the p-value 0.05 cutoff was used to differentiate the up-regulated genes from unchanged as well as down-regulated genes. The association between the presence of L1 and AGO2 binding site was analyzed using chi-square test.

## Supporting Information

Figure S1 S1.1 shows the log-scale expression level of experiment GSE3167 bladder carcinoma situ vs normal bladder epithelium. The test and control are the same as shown in the supporting table S3.7. S1.2 shows the log-scale expression level of experiment GSE13911 microsatellite instable gastric cancer vs normal stomach epithelium. The test and control are the same as shown in the supporting table S3.8.
(PDF)

Figure S2 the distributions of genes commonly down-regulated in the independent experiments compared between genes containing L1 and genes without L1 including the list of L1-containing genesfound to be down-regulated in at least one experiment.
(PDF)

Table S1 Genes containing L1 sequences.
(PDF)

Table S2 Analysis of L1 characteristics.
(PDF)

Table S3 GSE records, GSM samples, type of t-test, and 2×2 contingency tables of chi-square tests corresponding to the expression analysis of genes possessing internal L1s.
(PDF)

Table S4 The 2×2 contingency tables corresponding to CU-DREAM chi-square tests.
(PDF)

Table S5 The 2×2 contingency tables corresponding to genes possessing internal L1s which were down-regulated in both cancer and demethylated normal cells.
(PDF)

Table S6 List of GSE records and GSM samples, type of t-test, and 2×2 contingency tables of chi-square tests for the analysis of up-regulation in genes possessing internal L1s. The data were displayed for expression in demethylated lung cancer cells (Table S6.1 and S6.2), DICER1sh (Table S6.3 and S6.4), AGO1sh (Table S6.5), AGO3sh (Table S6.6), and AGO4sh (Table S6.7).
(PDF)

Table S7 Odds ratios, p values and 2×2 contingency tables of chi-square tests comparing between expression of genes possessing nearby intergenic L1s and the rest of genes in bladder carcinoma situ. The GSE and GSM records were listed in supporting table S3.7.
(PDF)
