# Supplementary material for: Hypomethylation of Intragenic LINE-1 Represses Transcription in Cancer Cells through AGO2
Source: PLoS One. 2011 Mar 15;6(3):e17934. doi: 10.1371/journal.pone.0017934 (PMC3057998; doi:10.1371/journal.pone.0017934)
Supplement: Figure S1 — S1.1 shows the log-scale expression level of experiment GSE3167 bladder carcinoma situ vs normal bladder epithelium. The test and control are the same as shown in the supporting table S3.7. S1.2 shows the log-scale expression level of experiment GSE13911 microsatellite instable gastric cancer vs normal stomach epithelium. The test and control are the same as shown in the supporting table S3.8. (PDF) [file pone.0017934.s001.pdf]

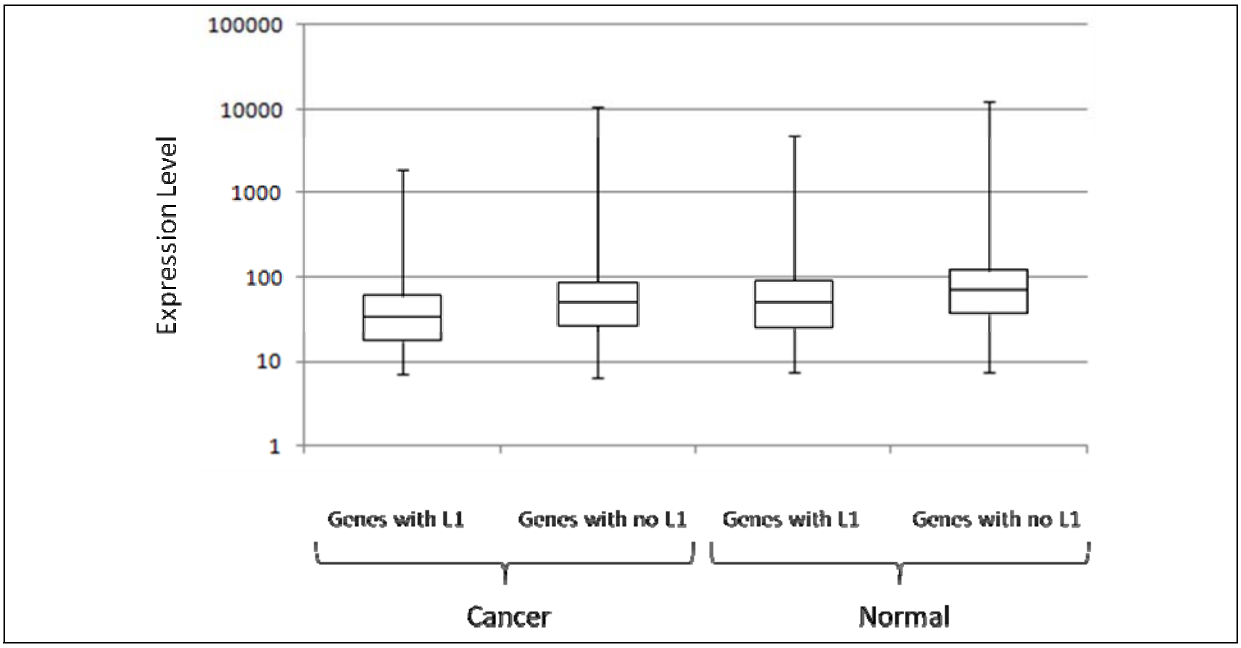

Fig S1.1 shows the log-scale expression level of experiment GSE3167 bladder carcinoma situ vs normal bladder epithelium. The test and control are of the same as in the supplementary Table 3.7.

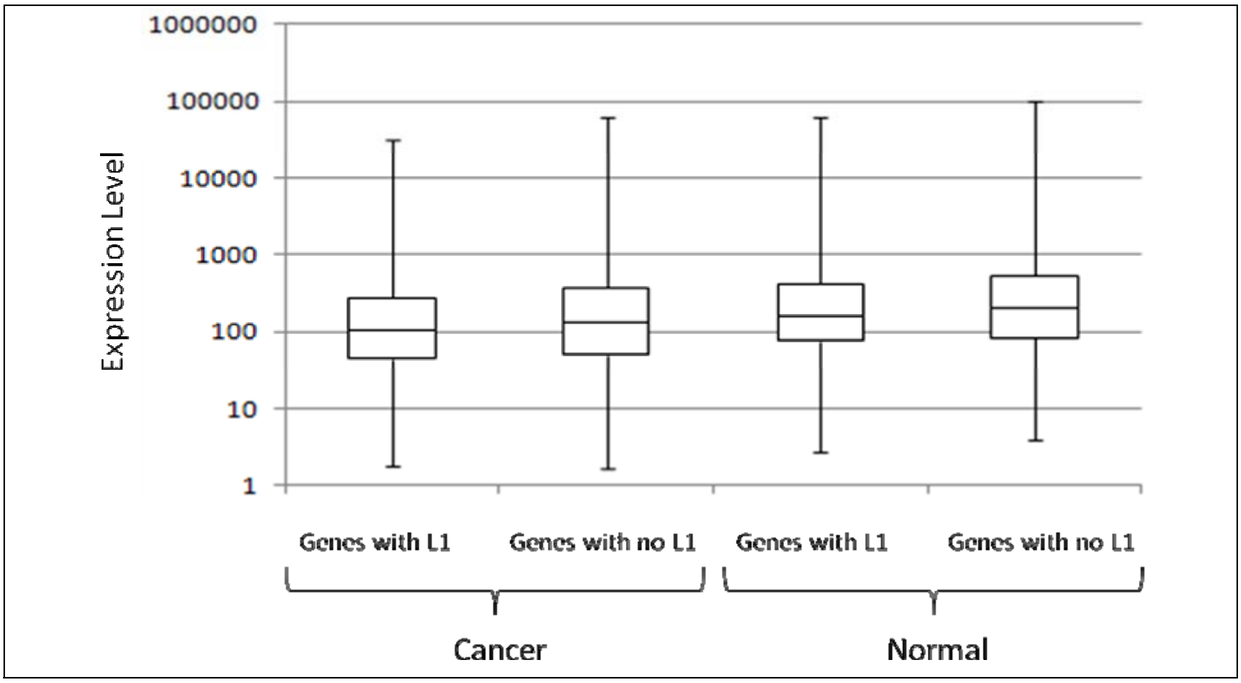

Fig S1.2 shows the log-scale expression level of experiment GSE13911 microsatellite instable gastric cancer vs normal stomach epithelium. The test and control are of the same as in the supplementary Table 3.8.
