# Supplementary material for: Hypomethylation of Intragenic LINE-1 Represses Transcription in Cancer Cells through AGO2
Source: PLoS One. 2011 Mar 15;6(3):e17934. doi: 10.1371/journal.pone.0017934 (PMC3057998; doi:10.1371/journal.pone.0017934)
Supplement: Figure S2 — the distributions of genes commonly down-regulated in the independent experiments compared between genes containing L1 and genes without L1 including the list of L1-containing genesfound to be down-regulated in at least one experiment. (PDF) [file pone.0017934.s002.pdf]

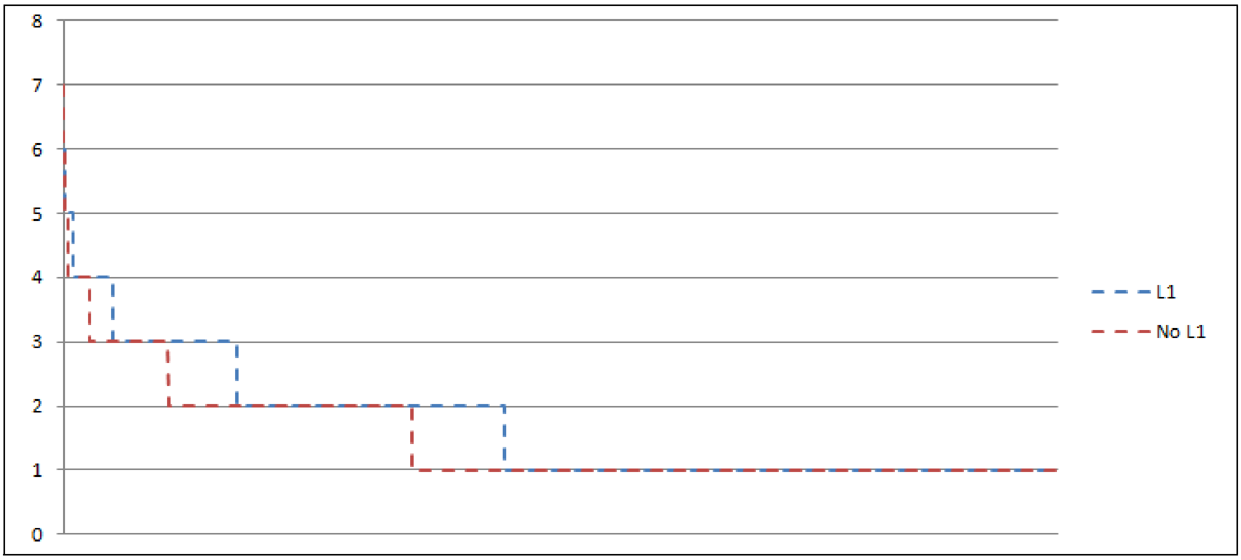

Supporting fig S2. Common genes: The figure shows a comparison of common down-regulated genes between genes with L1 and noL1. We collected the genes from nine experiments (Supporting Table S3.1 to S3.9). There are 816 down-regulated genes with L1 and 8,789 with no L1 respectively. The genes are placed on the x-axis. The y-axis indicates the number of experiments that the genes were found in common. Note that two plots are scaled to the same width. The unpaired t-test yields a p-value of 7.99E-08 (mean L1 = 1.6642, mean no L1 = 1.4861).
