# Supplementary material for: Hypomethylation of Intragenic LINE-1 Represses Transcription in Cancer Cells through AGO2
Source: PLoS One. 2011 Mar 15;6(3):e17934. doi: 10.1371/journal.pone.0017934 (PMC3057998; doi:10.1371/journal.pone.0017934)
Supplement: Table S1 — Genes containing L1 sequences. (PDF) [file pone.0017934.s003.pdf]

Table 1.1 Genes containing L1 sequences (1454 genes).

NCBI gene ID	NCBI gene name	mRNA accession
22	ABCB7	NM_004299
40	ACCN1	NM_001094 NM_183377
105	ADARB2	NM_018702
132	ADK	NM_001123 NM_006721
225	ABCD2	NM_005164
238	ALK	NM_004304
245	ALOX12P2	NR_002710
288	ANK3	NM_001149 NM_020987
313	AOAH	NM_001637
320	APBA1	NM_001163
329	BIRC2	NM_001166
351	APP	NM_000484 NM_201413 NM_201414
367	AR	NM_000044 NM_001011645
411	ARSB	NM_000046 NM_198709
472	ATM	NM_000051 NM_138292
540	ATP7B	NM_000053 NM_001005918
545	ATR	NM_001184
546	ATRX	NM_000489 NM_138270 NM_138271
549	AUH	NM_001698
577	BAI3	NM_001704
627	BDNF	NM_001709 NM_170731 NM_170732 NM_170733 NM_170734 NM_170735
644	BLVRA	NM_000712
658	BMPR1B	NM_001203
665	BNIP3L	NM_004331
667	DST	NM_001723 NM_015548 NM_020388 NM_183380
729	C6	NM_000065
735	C9	NM_001737
753	C18orf1	NM_001003674 NM_001003675 NM_004338 NM_181481 NM_181482 NM_181483
814	CAMK4	NM_001744
817	CAMK2D	NM_001221 NM_172115 NM_172127 NM_172128
840	CASP7	NM_001227 NM_033338 NM_033339 NM_033340
952	CD38	NM_001775
953	ENTPD1	NM_001776
987	LRBA	NM_006726
1006	CDH8	NM_001796
1010	CDH12	NM_004061
1012	CDH13	NM_001257
1015	CDH17	NM_004063
1016	CDH18	NM_004934
1047	CLGN	NM_004362
1062	CENPE	NM_001813
1121	CHM	NM_000390
1129	CHRM2	NM_000739 NM_001006626 NM_001006627 NM_001006628 NM_001006629 [illegible]
1130	LYST	NM_000081 NM_001005736
1131	CHRM3	NM_000740
1136	CHRNA3	NM_000743
1139	CHRNA7	NM_000746
1161	ERCC8	NM_000082 NM_001007233 NM_001007234

1176	AP3S1	NM_001002924 NM_001284
1193	CLIC2	NM_001289
1272	CNTN1	NM_001843 NM_175038
1286	COL4A4	NM_000092
1287	COL4A5	NM_000495 NM_033380 NM_033381
1288	COL4A6	NM_001847 NM_033641
1295	COL8A1	NM_001850 NM_020351
1301	COL11A1	NM_001854 NM_080629 NM_080630
1310	COL19A1	NM_001858
1362	CPD	NM_001304
1378	CR1	NM_000573 NM_000651
1432	MAPK14	NM_001315 NM_139012 NM_139013 NM_139014
1496	CTNNA2	NM_004389
1501	CTNND2	NM_001332
1550	CYP2A7P1	-
1557	CYP2C19	NM_000769
1558	CYP2C8	NM_000770
1559	CYP2C9	NM_000771
1562	CYP2C18	NM_000772
1574	CYP3A	-
1577	CYP3A5	NM_000777
1600	DAB1	NM_021080
1607	DGKB	NM_004080 NM_145695
1612	DAPK1	NM_004938
1617	DAZ1	NM_004081
1620	DBC1	NM_014618
1629	DBT	NM_001918
1630	DCC	NM_005215
1633	DCK	NM_000788
1644	DDC	NM_000790
1666	DECR1	NM_001359
1730	DIAPH2	NM_006729 NM_007309
1740	DLG2	NM_001364
1756	DMD	NM_000109 NM_004006 NM_004007 NM_004009 NM_004010 NM_004011 NM_004012 [illegible]
1770	DNAH9	NM_001372 NM_004662
1780	DYNC1I1	NM_004411
1794	DOCK2	NM_004946
1795	DOCK3	NM_004947
1806	DPYD	NM_000110
1826	DSCAM	NM_001389 NM_206887
1828	DSG1	NM_001942
1837	DTNA	NM_001390 NM_001391 NM_001392 NM_032975 NM_032978 NM_032979 NM_032980 [illegible]
1896	EDA	NM_001005609 NM_001005610 NM_001005611 NM_001005612 NM_001005613 [illegible]
1910	EDNRB	NM_000115 NM_003991
1950	EGF	NM_001963
2042	EPHA3	NM_005233 NM_182644
2047	EPHB1	NM_004441
2060	EPS15	NM_001981
2066	ERBB4	NM_005235
2070	EYA4	NM_004100 NM_172103 NM_172105
2099	ESR1	NM_000125
2121	EVC	NM_153717

2162	F13A1	NM_000129
2176	FANCC	NM_000136
2182	ACSL4	NM_004458 NM_022977
2201	FBN2	NM_001999
2218	FKTN	NM_006731
2222	FDFT1	NM_004462
2257	FGF12	NM_004113 NM_021032
2259	FGF14	NM_004115 NM_175929
2262	GPC5	NM_004466
2272	FHIT	NM_002012
2312	FLG	NM_002016
2330	FMO5	NM_001461
2334	AFF2	NM_002025
2444	FRK	NM_002031
2509	FTHP1	-
2515	ADAM2	NM_001464
2530	FUT8	NM_004480 NM_178154 NM_178155 NM_178156 NM_178157
2556	GABRA3	NM_000808
2560	GABRB1	NM_000812
2585	GALK2	NM_001001556 NM_002044
2643	GCH1	NM_000161 NM_001024024 NM_001024070 NM_001024071
2690	GHR	NM_000163
2719	GPC3	NM_004484
2742	GLRA2	NM_002063
2743	GLRB	NM_000824
2762	GMDS	NM_001500
2768	GNA12	NM_007353
2805	GOT1	NM_002079
2863	GPR39	NM_001508
2890	GRIA1	NM_000827
2892	GRIA3	NM_000828 NM_007325 NM_181894
2893	GRIA4	NM_000829
2894	GRID1	NM_017551
2895	GRID2	NM_001510
2898	GRIK2	NM_021956 NM_175768
2900	GRIK4	NM_014619
2915	GRM5	NM_000842
2917	GRM7	NM_000844 NM_181874 NM_181875
2918	GRM8	NM_000845
2932	GSK3B	NM_002093
2977	GUCY1A2	NM_000855
3064	HTT	NM_002111
3075	CFH	NM_000186 NM_001014975
3096	HIVEP1	NM_002114
3176	HNMT	NM_001024074 NM_001024075 NM_006895
3358	HTR2C	NM_000868
3360	HTR4	NM_000870 NM_001040169 NM_001040171 NM_001040172 NM_001040173 [illegible]
3376	IARS	NM_002161 NM_013417
3382	ICA1	NM_004968 NM_022307
3426	CFI	NM_000204
3482	IGF2R	NM_000876
3535	IGL@	-

3574	IL7	NM_000880
3600	IL15	NM_000585 NM_172174
3617	IMPG1	NM_001563
3673	ITGA2	NM_002203
3680	ITGA9	NM_002207
3684	ITGAM	NM_000632
3688	ITGB1	NM_002211 NM_033666 NM_033667 NM_033668 NM_033669 NM_133376
3694	ITGB6	NM_000888
3709	ITPR2	NM_002223
3717	JAK2	NM_004972
3756	KCNH1	NM_002238 NM_172362
3760	KCNJ3	NM_002239
3776	KCNK2	NM_001017424 NM_001017425 NM_014217
3781	KCNN2	NM_021614 NM_170775
3784	KCNQ1	NM_000218 NM_181797 NM_181798
3790	KCNS3	NM_002252
3908	LAMA2	NM_000426
3953	LEPR	NM_001003679 NM_001003680 NM_002303
3973	LHCGR	NM_000233
3998	LMAN1	NM_005570
4008	LMO7	NM_005358
4017	LOXL2	NM_002318
4018	LPA	NM_005577
4026	LPP	NM_005578
4036	LRP2	NM_004525
4045	LSAMP	NM_002338
4052	LTBP1	NM_000627 NM_206943
4063	LY9	NM_001033667 NM_002348
4065	LY75	NM_002349
4126	MANBA	NM_005908
4128	MAOA	NM_000240
4133	MAP2	NM_001039538 NM_002374 NM_031845 NM_031847
4139	MARK1	NM_018650
4158	MC2R	NM_000529
4163	MCC	NM_002387
4179	CD46	NM_002389 NM_153826 NM_172350 NM_172351 NM_172352 NM_172353 NM_172354 [illegible]
4199	ME1	NM_002395
4208	MEF2C	NM_002397
4223	MEOX2	NM_005924
4233	MET	NM_000245
4253	CTAGE5	NM_005930 NM_203354 NM_203355 NM_203356 NM_203357
4255	MGMT	NM_002412
4281	MID1	NM_000381 NM_033290 NM_033291
4285	MIPEP	NM_005932
4306	NR3C2	NM_000901
4311	MME	NM_000902 NM_007287 NM_007288 NM_007289
4325	MMP16	NM_005941 NM_022564
4331	MNAT1	NM_002431
4332	MNDA	NM_002432
4361	MRE11A	NM_005590 NM_005591
4437	MSH3	NM_002439
4438	MSH4	NM_002440

4507	MTAP	NM_002451
4593	MUSK	NM_005592
4642	MYO1D	NM_015194
4644	MYO5A	NM_000259
4646	MYO6	NM_004999
4649	MYO9A	NM_006901
4659	PPP1R12A	NM_002480
4684	NCAM1	NM_000615 NM_181351
4703	NEB	NM_004543
4704	NDUFA9	NM_005002
4724	NDUFS4	NM_002495
4734	NEDD4	NM_006154 NM_198400
4745	NELL1	NM_006157
4750	NEK1	NM_012224
4753	NELL2	NM_006159
4756	NEO1	NM_002499
4772	NFATC1	NM_006162 NM_172387 NM_172388 NM_172389 NM_172390
4810	NHS	NM_198270
4848	CNOT2	NM_014515
4867	NPHP1	NM_000272 NM_207181
4897	NRCAM	NM_001037132 NM_001037133 NM_005010
4921	DDR2	NM_001014796 NM_006182
4940	OAS3	NM_006187
4978	OPCML	NM_001012393 NM_002545
4983	OPHN1	NM_002547
4988	OPRM1	NM_000914 NM_001008503 NM_001008504 NM_001008505
5033	P4HA1	NM_000917 NM_001017962
5053	PAH	NM_000277
5058	PAK1	NM_002576
5067	CNTN3	NM_020872
5101	PCDH9	NM_020403 NM_203487
5126	PCSK2	NM_002594
5128	PCTK2	NM_002595
5136	PDE1A	NM_001003683 NM_005019
5137	PDE1C	NM_005020
5139	PDE3A	NM_000921
5140	PDE3B	NM_000922
5142	PDE4B	NM_001037339 NM_001037340 NM_001037341 NM_002600
5144	PDE4D	NM_006203
5151	PDE8A	NM_002605 NM_173454 NM_173455 NM_173456 NM_173457
5156	PDGFRA	NM_006206
5194	PEX13	NM_002618
5205	ATP8B1	NM_005603
5212	VIT	NM_053276
5218	PFTK1	NM_012395
5241	PGR	NM_000926
5243	ABCB1	NM_000927
5251	PHEX	NM_000444
5255	PHKA1	NM_002637
5257	PHKB	NM_000293 NM_001031835
5288	PIK3C2G	NM_004570
5314	PKHD1	NM_138694 NM_170724

5332	PLCB4	NM_000933 NM_182797
5334	PLCL1	NM_006226
5337	PLD1	NM_002662
5340	PLG	NM_000301
5412	UBL3	NM_007106
5446	PON3	NM_000940
5475	PPEF1	NM_006240 NM_152224 NM_152226
5521	PPP2R2B	NM_004576 NM_181674 NM_181675 NM_181676 NM_181677 NM_181678
5523	PPP2R3A	NM_002718 NM_181897
5550	PREP	NM_002726
5558	PRIM2	NM_000947
5567	PRKACB	NM_002731 NM_182948 NM_207578
5570	PKIB	NM_032471 NM_181794 NM_181795
5577	PRKAR2B	NM_002736
5578	PRKCA	NM_002737
5586	PKN2	NM_006256
5587	PRKD1	NM_002742
5588	PRKCQ	NM_006257
5592	PRKG1	NM_006258
5593	PRKG2	NM_006259
5602	MAPK10	NM_002753 NM_138980 NM_138981 NM_138982
5611	DNAJC3	NM_006260
5618	PRLR	NM_000949
5627	PROS1	NM_000313
5638	PRRG1	NM_000950
5649	RELN	NM_005045 NM_173054
5682	PSMA1	NM_002786 NM_148976
5728	PTEN	NM_000314
5738	PTGFRN	NM_020440
5747	PTK2	NM_005607 NM_153831
5783	PTPN13	NM_006264 NM_080683 NM_080684 NM_080685
5789	PTPRD	NM_002839 NM_130391 NM_130392 NM_130393
5796	PTPRK	NM_002844
5797	PTPRM	NM_002845
5801	PTPRR	NM_002849 NM_130846
5825	ABCD3	NM_002858
5834	PYGB	NM_002862
5858	PZP	NM_002864
5890	RAD51L1	NM_002877 NM_133509 NM_133510
5906	RAP1A	NM_001010935 NM_002884
5910	RAP1GDS1	NM_021159
5924	RASGRF2	NM_006909
5925	RB1	NM_000321
5927	JARID1A	NM_005056
5983	RFC3	NM_002915 NM_181558
5991	RFX3	NM_002919 NM_134428
6000	RGS7	NM_002924
6091	ROBO1	NM_002941 NM_133631
6092	ROBO2	NM_002942
6095	RORA	NM_002943 NM_134260 NM_134261 NM_134262
6098	ROS1	NM_002944
6119	RPA3	NM_002947

6196	RPS6KA2	NM_001006932 NM_021135
6252	RTN1	NM_021136 NM_206852 NM_206857
6262	RYR2	NM_001035
6263	RYR3	NM_001036
6342	SCP2	NM_001007098 NM_001007099 NM_001007100 NM_001007250 NM_002979
6344	SCTR	NM_002980
6399	TRAPPC2	NM_001011658 NM_014563
6444	SGCD	NM_000337 NM_172244
6451	SH3BGRL	NM_003022
6456	SH3GL2	NM_003026
6457	SH3GL3	NM_003027
6480	ST6GAL1	NM_003032 NM_173216 NM_173217
6505	SLC1A1	NM_004170
6546	SLC8A1	NM_021097
6549	SLC9A2	NM_003048
6565	SLC15A2	NM_021082
6579	SLCO1A2	NM_005075 NM_021094 NM_134431
6581	SLC22A3	NM_021977
6586	SLIT3	NM_003062
6641	SNTB1	NM_021021
6660	SOX5	NM_006940 NM_152989 NM_178010
6683	SPAST	NM_014946 NM_199436
6695	SPOCK1	NM_004598
6764	ST5	NM_005418 NM_139157 NM_213618
6769	STAC	NM_003149
6775	STAT4	NM_003151
6786	STIM1	NM_003156
6788	STK3	NM_006281
6845	VAMP7	NM_005638
6847	SYCP1	NM_003176
6854	SYN2	NM_003178 NM_133625
6857	SYT1	NM_005639
6870	TACR3	NM_001059
6885	MAP3K7	NM_003188 NM_145331 NM_145332 NM_145333
6905	TBCE	NM_003193
6935	ZEB1	NM_030751
6955	TRA@	-
7007	TECTA	NM_005422
7035	TFPI	NM_001032281 NM_006287
7068	THRB	NM_000461
7092	TLL1	NM_012464
7107	GPR137B	NM_003272
7111	TMOD1	NM_003275
7164	TPD52L1	NM_001003395 NM_001003396 NM_001003397 NM_003287
7223	TRPC4	NM_016179
7224	TRPC5	NM_012471
7225	TRPC6	NM_004621
7253	TSHR	NM_000369 NM_001018036
7260	TSSC1	NM_003310
7267	TTC3	NM_001001894 NM_003316
7299	TYR	NM_000372
7325	UBE2E2	NM_152653

7367	UGT2B17	NM_001077
7373	COL14A1	NM_021110
7399	USH2A	NM_007123 NM_206933
7402	UTRN	NM_007124
7403	UTX	NM_021140
7404	UTY	NM_007125 NM_182659 NM_182660
7405	UVRAG	NM_003369
7424	VEGFC	NM_005429
7518	XRCC4	NM_003401 NM_022406 NM_022550
7587	ZNF37A	NM_001007094 NM_003421
7620	ZNF69	NM_021915
7626	ZNF75	NM_007131
7757	ZNF208	NM_007153
7762	ZNF215	NM_013250
7768	ZNF225	NM_013362
7770	ZNF227	NM_182490
7813	EVI5	NM_005665
7827	NPHS2	NM_014625
7840	ALMS1	NM_015120
7851	MALL	NM_005434
7881	KCNAB1	NM_003471 NM_172159 NM_172160
7913	DEK	NM_003472
7957	EPM2A	NM_001018041 NM_005670
7991	TUSC3	NM_006765 NM_178234
8001	GLRA3	NM_006529
8038	ADAM12	NM_003474 NM_021641
8139	GAN	NM_022041
8287	USP9Y	NM_004654
8411	EEA1	NM_003566
8452	CUL3	NM_003590
8455	ATRN	NM_139321 NM_139322
8460	TPST1	NM_003596
8464	SUPT3H	NM_003599 NM_181356
8499	PPFIA2	NM_003625
8502	PKP4	NM_001005476 NM_003628
8516	ITGA8	NM_003638
8540	AGPS	NM_003659
8546	AP3B1	NM_003664
8573	CASK	NM_003688
8577	TMEFF1	NM_003692
8601	RGS20	NM_003702 NM_170587
8618	CADPS	NM_003716 NM_183393 NM_183394
8621	CDC2L5	NM_003718 NM_031267
8633	UNC5C	NM_003728
8654	PDE5A	NM_001083 NM_033430 NM_033437
8671	SLC4A4	NM_003759
8685	MARCO	NM_006770
8708	B3GALT1	NM_020981
8732	RNGTT	NM_003800
8745	ADAM23	NM_003812
8756	ADAM7	NM_003817
8801	SUCLG2	NM_003848

8805	TRIM24	NM_003852 NM_015905
8808	IL1RL2	NM_003854
8821	INPP4B	NM_003866
8832	CD84	NM_003874
8854	ALDH1A2	NM_003888 NM_170696 NM_170697
8874	ARHGEF7	NM_003899 NM_145735
8898	MTMR2	NM_016156 NM_201278 NM_201281
8924	HERC2	NM_004667
8925	HERC1	NM_003922
8935	SKAP2	NM_003930
8936	WASF1	NM_001024934 NM_001024935 NM_001024936 NM_003931
8942	KYNU	NM_001032998 NM_003937
9014	TAF1B	NM_005680
9037	SEMA5A	NM_003966
9061	PAPSS1	NM_005443
9079	LDB2	NM_001290
9081	PRY	NM_004676
9162	DGKI	NM_004717
9173	IL1RL1	NM_003856 NM_016232
9194	SLC16A7	NM_004731
9201	DCLK1	NM_004734
9213	XPR1	NM_004736
9223	MAGI1	NM_001033057 NM_004742 NM_015520
9228	DLGAP2	NM_004745
9229	DLGAP1	NM_001003809 NM_004746
9312	KCNB2	NM_004770
9348	NDST3	NM_004784
9358	ITGBL1	NM_004791
9364	RAB28	NM_001017979 NM_004249
9369	NRXN3	NM_004796 NM_138970
9372	ZFYVE9	NM_004799 NM_007323 NM_007324
9378	NRXN1	NM_004801 NM_138735
9425	CDYL	NM_004824 NM_170751 NM_170752
9457	FHL5	NM_020482
9462	RASAL2	NM_004841 NM_170692
9465	AKAP7	NM_004842 NM_016377 NM_138633
9472	AKAP6	NM_004274
9474	ATG5	NM_004849
9508	ADAMTS3	NM_014243
9522	SCAMP1	NM_004866 NM_052822
9562	MINPP1	NM_004897
9568	GABBR2	NM_005458
9576	SPAG6	NM_012443 NM_172242
9577	BRE	NM_004899 NM_199191 NM_199192 NM_199193 NM_199194
9586	CREB5	NM_001011666 NM_004904 NM_182898 NM_182899
9627	SNCAIP	NM_005460
9628	RGS6	NM_004296
9630	GNA14	NM_004297
9649	RALGPS1	NM_014636
9657	IQCB1	NM_001023570 NM_001023571
9659	PDE4DIP	NM_001002810 NM_001002811 NM_001002812 NM_014644 NM_022359
9666	DZIP3	NM_014648

9692	KIAA0391	NM_014672
9699	RIMS2	NM_014677
9702	CEP57	NM_014679
9705	ST18	NM_014682
9722	NOS1AP	NM_014697
9723	SEMA3E	NM_012431
9730	VPRBP	NM_014703
9732	DOCK4	NM_014705
9734	HDAC9	NM_014707 NM_058176 NM_058177 NM_178423 NM_178425
9743	RICS	NM_014715
9749	PHACTR2	NM_014721
9758	FRMPD4	NM_014728
9760	TOX	NM_014729
9765	ZFYVE16	NM_014733
9779	TBC1D5	NM_014744
9786	KIAA0586	NM_014749
9811	KIAA0427	NM_014772
9832	JAKMIP2	NM_014790
9843	HEPH	NM_014799 NM_138737
9844	ELMO1	NM_001039459 NM_014800 NM_130442
9863	MAGI2	NM_012301
9871	SEC24D	NM_014822
9873	FCHSD2	NM_014824
9892	SNAP91	NM_014841
9899	SV2B	NM_014848
9910	RABGAP1L	NM_001035230 NM_014857
9934	P2RY14	NM_014879
9938	ARHGAP25	NM_001007231 NM_014882
9942	XYLB	NM_005108
9958	USP15	NM_006313
9990	SLC12A6	NM_005135
10000	AKT3	NM_005465 NM_181690
10010	TANK	NM_004180 NM_133484
10056	FARSB	NM_005687
10060	ABCC9	NM_005691 NM_020297 NM_020298
10082	GPC6	NM_005708
10085	EDIL3	NM_005711
10090	UST	NM_005715
10098	TSPAN5	NM_005723
10144	FAM13A1	NM_001015045 NM_014883
10165	SLC25A13	NM_014251
10178	ODZ1	NM_014253
10207	INADL	NM_176877
10225	CD96	NM_005816 NM_198196
10231	RCAN2	NM_005822
10240	MRPS31	NM_005830
10242	KCNMB2	NM_005832 NM_181361
10243	GPHN	NM_001024218 NM_020806
10257	ABCC4	NM_005845
10274	STAG1	NM_005862
10283	SDCCAG10	NM_005869
10308	ZNF267	NM_003414

10314	LANCL1	NM_006055
10345	TRDN	NM_006073
10350	ABCA9	NM_080283
10352	WARS2	NM_015836 NM_201263
10367	CBARA1	NM_006077
10371	SEMA3A	NM_006080
10392	NOD1	NM_006092
10404	PGCP	NM_016134
10406	WFDC2	NM_006103 NM_080734 NM_080735 NM_080736
10418	SPON1	NM_006108
10451	VAV3	NM_006113
10463	SLC30A9	NM_006345
10464	PIBF1	NM_006346
10466	COG5	NM_006348 NM_181733
10495	ENOX2	NM_006375 NM_182314
10497	UNC13B	NM_006377
10513	APPBP2	NM_006380
10529	NEBL	NM_006393 NM_213569
10558	SPTLC1	NM_006415 NM_178324
10563	CXCL13	NM_006419
10564	ARFGEF2	NM_006420
10599	SLCO1B1	NM_006446
10643	IGF2BP3	NM_006547
10651	MTX2	NM_001006635 NM_006554
10667	FARS2	NM_006567
10718	NRG3	NM_001010848
10721	POLQ	NM_199420
10752	CHL1	NM_006614
10800	CYSLTR1	NM_006639
10846	PDE10A	NM_006661
10873	ME3	NM_001014811 NM_006680
10874	NMU	NM_006681
10877	CFHR4	NM_006684
10878	CFHR3	NM_021023
10886	NPFFR2	NM_004885 NM_053036
10888	GPR83	NM_016540
10941	UGT2A1	NM_006798
10973	ASCC3	NM_006828 NM_022091
11001	SLC27A2	NM_003645
11005	SPINK5	NM_006846
11027	LILRA2	NM_006866
11036	GTF2A1L	NM_006872 NM_172196
11055	ZPBP	NM_007009
11061	LECT1	NM_001011705 NM_007015
11064	CEP110	NM_007018
11107	PRDM5	NM_018699
11122	PTPRT	NM_007050 NM_133170
11124	FAF1	NM_007051 NM_131917
11128	POLR3A	NM_007055
11136	SLC7A9	NM_014270
11141	IL1RAPL1	NM_014271
11146	GLMN	NM_053274

11148	HHLA2	NM_007072
11169	WDHD1	NM_001008396 NM_007086
11174	ADAMTS6	NM_197941
11214	AKAP13	NM_006738 NM_007200 NM_144767
11227	GALNT5	NM_014568
11231	SEC63	NM_007214
11235	PDCD10	NM_007217 NM_145859 NM_145860
11275	KLHL2	NM_007246
11281	POU6F2	NM_007252
22796	COG2	NM_007357
22797	TFEC	NM_001018058 NM_012252
22829	NLGN4Y	NM_014893
22862	FNDC3A	NM_014923
22866	CNKSR2	NM_014927
22871	NLGN1	NM_014932
22882	ZHX2	NM_014943
22891	ZNF365	NM_014951 NM_199450 NM_199451 NM_199452
22901	ARSG	NM_014960
22920	KIFAP3	NM_014970
22955	SCMH1	NM_001031694 NM_012236
22986	SORCS3	NM_014978
22987	SV2C	NM_014979
22990	PCNX	NM_014982
22999	RIMS1	NM_014989
23007	PLCH1	NM_014996
23026	MYO16	NM_015011
23047	PDS5B	NM_015032
23049	SMG1	NM_015092
23057	NMNAT2	NM_015039 NM_170706
23071	TXNDC4	NM_015051
23077	MYCBP2	NM_015057
23081	JMJD2C	NM_015061
23090	ZNF423	NM_015069
23092	ARHGAP26	NM_015071
23105	FSTL4	NM_015082
23122	CLASP2	NM_015097
23132	RAD54L2	NM_015106
23136	EPB41L3	NM_012307
23161	SNX13	NM_015132
23167	EFR3A	NM_015137
23179	RGL1	NM_015149
23194	FBXL7	NM_012304
23215	BAT2D1	NM_015172
23229	ARHGEF9	NM_015185
23236	PLCB1	NM_015192 NM_182734
23239	PHLPP	NM_194449
23242	COBL	NM_015198
23245	ASTN2	NM_014010 NM_198186 NM_198187 NM_198188
23253	ANKRD12	NM_015208
23256	SCFD1	NM_016106 NM_182835
23261	CAMTA1	NM_015215
23273	KIAA0367	NM_015225

23275	POFUT2	NM_015227 NM_133634 NM_133635
23281	KIAA0774	NM_001033602 NM_015233
23284	LPHN3	NM_015236
23287	AGTPBP1	NM_015239
23301	EHBP1	NM_015252
23312	DMXL2	NM_015263
23345	SYNE1	NM_015293 NM_033071 NM_133650 NM_182961
23414	ZFPM2	NM_012082
23431	AP4E1	NM_007347
23530	NNT	NM_012343 NM_182977
23553	HYAL4	NM_012269
23586	DDX58	NM_014314
23601	CLEC5A	NM_013252
23604	DAPK2	NM_014326
23635	SSBP2	NM_012446
23639	LRRC6	NM_012472
23643	LY96	NM_015364
23783	ADPRTL4	-
24137	KIF4A	NM_012310
24145	PANX1	NM_015368
25769	SLC24A2	NM_020344
25771	TBC1D22A	NM_014346
25827	FBXL2	NM_012157
25834	MGAT4C	NM_013244
25849	DKFZP564O0823	NM_015393
25914	RTTN	NM_173630
25924	MYRIP	NM_015460
25925	ZNF521	NM_015461
25963	TMEM87A	NM_015497
26002	MOXD1	NM_001031699
26009	ZZZ3	NM_015534
26033	ATRNL1	NM_207303
26034	PIP3-E	NM_015553
26040	SETBP1	NM_015559
26047	CNTNAP2	NM_014141
26052	DNM3	NM_015569
26053	AUTS2	NM_015570
26054	SENP6	NM_015571
26059	ERC2	NM_015576
26090	ABHD12	NM_015600
26122	EPC2	NM_015630
26133	TRPC4AP	NM_015638 NM_199368
26137	ZBTB20	NM_015642
26154	ABCA12	NM_015657 NM_173076
26166	RGS22	NM_015668
26228	STAP1	NM_012108
26235	FBXL4	NM_012160
26280	IL1RAPL2	NM_017416
26290	GALNT8	NM_017417
26575	RGS17	NM_012419
26610	ELP4	NM_019040
26750	RPS6KC1	NM_012424

26958	COPG2	NM_012133
26960	NBEA	NM_015678
27067	STAU2	NM_014393
27068	PPA2	NM_001034191 NM_006903 NM_176866 NM_176867 NM_176869
27115	PDE7B	NM_018945
27123	DKK2	NM_014421
27130	INVS	NM_014425 NM_183245
27152	INTU	NM_015693
27159	CHIA	NM_021797 NM_201653
27185	DISC1	NM_001012957 NM_001012958 NM_001012959 NM_018662
27194	SEDLP4	-
27241	BBS9	NM_001033604 NM_001033605 NM_014451 NM_198428
27242	TNFRSF21	NM_014452
27252	KLHL20	NM_014458
27255	CNTN6	NM_014461
27258	LSM3	NM_014463
27283	TINAG	NM_014464
27291	C10orf28	NM_014472
27303	RBMS3	NM_001003792 NM_001003793 NM_014483
27306	PGDS	NM_014485
27328	PCDH11X	NM_014522 NM_032967 NM_032968 NM_032969
27332	ZNF638	NM_001014972 NM_014497
27333	GOLIM4	NM_014498
28957	MRPS28	NM_014018
28965	SLC27A6	NM_001017372 NM_014031
28998	MRPL13	NM_014078
29119	CTNNA3	NM_013266
29767	TMOD2	NM_014548
29906	ST8SIA5	NM_013305
29922	NME7	NM_013330 NM_197972
29953	TRHDE	NM_013381
29957	SLC25A24	NM_013386 NM_213651
29967	LRP12	NM_013437
29969	MDFIC	NM_199072
29970	SCHIP1	NM_014575
29994	BAZ2B	NM_013450
30010	NXPH1	NM_152745
49855	SCAPER	NM_020843
50485	SMARCAL1	NM_014140
50507	NOX4	NM_016931
50814	NSDHL	NM_015922
50859	SPOCK3	NM_001040159 NM_016950
50863	HNT	NM_016522
50940	PDE11A	NM_016953
51057	LOC51057	NM_015910
51071	DERA	NM_015954
51086	TNNI3K	NM_015978
51134	CCDC41	NM_016122
51164	DCTN4	NM_016221
51167	CYB5R4	NM_016230
51170	HSD17B11	NM_016245
51196	PLCE1	NM_016341

51265	CDKL3	NM_016508
51281	ANKMY1	NM_016552 NM_017844
51302	CYP39A1	NM_016593
51306	C5orf5	NM_016603
51319	RSRC1	NM_016625
51334	PRR16	NM_016644
51366	UBR5	NM_015902
51390	AIG1	NM_016108
51397	COMMD10	NM_016144
51430	C1orf9	NM_014283 NM_016227
51454	GULP1	NM_016315
51473	DCDC2	NM_016356
51501	C11orf73	NM_016401
51542	VPS54	NM_001005739 NM_016516
51594	NAG	NM_015909
51606	ATP6V1H	NM_015941 NM_213619 NM_213620
51705	EMCN	NM_016242
51761	ATP8A2	NM_016529
51776	ZAK	NM_016653 NM_133646
53344	CHIC1	NM_001039840
53353	LRP1B	NM_018557
53358	SHC3	NM_016848
53616	ADAM22	NM_004194 NM_016351 NM_021721 NM_021722 NM_021723
53904	MYO3A	NM_017433
53942	CNTN5	NM_014361 NM_175566
54212	SNTG1	NM_018967
54221	SNTG2	NM_018968
54431	DNAJC10	NM_018981
54462	KIAA1128	NM_018999
54503	ZDHHC13	NM_001001483 NM_019028
54504	CPVL	NM_019029 NM_031311
54514	DDX4	NM_024415
54520	CCDC93	NM_019044
54532	USP53	NM_019050
54558	SPATA6	NM_019073
54714	CNGB3	NM_019098
54765	TRIM44	NM_017583
54768	HYDIN	NM_017558
54808	DYM	NM_017653
54809	SAMD9	NM_017654
54823	C1orf26	NM_017673
54827	FAM55D	NM_017678
54828	BCAS3	NM_017679
54830	NUP62CL	NM_017681
54832	VPS13C	NM_001018088 NM_017684 NM_018080 NM_020821
54839	LRRC49	NM_017691
54842	FLJ20160	NM_017694
54875	CNTLN	NM_017738
54882	ANKHD1	NM_017747 NM_017978 NM_024668
54885	TBC1D8B	NM_017752 NM_198881
54886	RP11-35N6.1	NM_017753 NM_207299
54914	KIAA1797	NM_017794

54916	C14orf101	NM_017799
54954	FAM120C	NM_017848
54967	CXorf48	NM_001031705 NM_017863
54970	TTC12	NM_017868
55010	C12orf48	NM_017915
55013	CCDC109B	NM_017918
55016	MARCH1	NM_017923
55023	PHIP	NM_017934
55031	USP47	NM_017944
55034	MOCOS	NM_017947
55041	PLEKHB2	NM_001031706 NM_017958
55061	SUSD4	NM_001037175 NM_017982
55064	C9orf68	NM_001039395
55068	ENOX1	NM_017993
55075	UACA	NM_001008224 NM_018003
55076	TMEM45A	NM_018004
55100	WDR70	NM_018034
55103	RALGPS2	NM_018037 NM_152663
55120	FANCL	NM_018062
55125	CEP192	NM_032142
55129	TMEM16K	NM_018075
55130	ARMC4	NM_018076
55165	CEP55	NM_018131
55217	TMLHE	NM_018196
55236	UBA6	NM_018227
55248	TMEM206	NM_018252
55255	WDR41	NM_018268
55275	VPS53	NM_018289
55277	FGGY	NM_018291
55289	ACOXL	NM_018308
55296	TBC1D19	NM_018317
55297	CCDC91	NM_018318
55328	C10orf59	NM_001031709 NM_018363
55331	PHCA	NM_018367
55334	SLC39A9	NM_018375
55351	STK32B	NM_018401
55356	SLC22A15	NM_018420
55423	SIRPG	NM_001039508 NM_018556 NM_080816
55504	TNFRSF19	NM_018647 NM_148957
55553	SOX6	NM_017508 NM_033326
55567	DNAH3	NM_017539
55576	STAB2	NM_017564
55607	PPP1R9A	NM_017650
55610	CCDC132	NM_017667 NM_024553
55613	MTMR8	NM_017677
55617	TASP1	NM_017714
55686	MREG	NM_018000
55703	POLR3B	NM_018082
55711	MLSTD1	NM_018099
55744	C7orf44	NM_018224
55757	UGCGL2	NM_020121
55779	WDR52	NM_018338

55788	LMBRD1	NM_018368
55789	DEPDC1B	NM_018369
55799	CACNA2D3	NM_018398
55840	EAF2	NM_018456
55843	ARHGAP15	NM_018460
55869	HDAC8	NM_018486
55871	CBWD1	NM_018491
55906	KIAA1166	NM_018684
55914	ERBB2IP	NM_001006600 NM_018695
56001	NXF2	NM_017809 NM_022053
56062	KLHL4	NM_019117 NM_057162
56159	TEX11	NM_001003811 NM_031276
56163	RNF17	NM_031277
56164	STK31	NM_031414 NM_032944
56169	MLZE	NM_031415
56171	DNAH7	NM_018897
56341	PRMT8	NM_019854
56477	CCL28	NM_148672
56479	KCNQ5	NM_019842
56852	RAD18	NM_020165
56884	FSTL5	NM_020116
56895	AGPAT4	NM_020133
56899	ANKS1B	NM_020140 NM_152788 NM_181670
56934	CA10	NM_020178
56957	OTUD7B	NM_020205
56981	PRDM11	NM_020229
56987	BBX	NM_020235
56990	CDC42SE2	NM_001038702 NM_020240
57047	PLSCR2	NM_020359
57054	DAZ3	NM_020364
57055	DAZ2	NM_001005785 NM_001005786 NM_020363
57062	DDX24	NM_020414
57094	CPA6	NM_020361
57097	PARP11	NM_020367
57102	C12orf4	NM_020374
57103	C12orf5	NM_020375
57113	TRPC7	NM_020389
57135	DAZ4	NM_001005375 NM_020420
57161	PELI2	NM_021255
57187	THOC2	NM_020449
57188	ADAMTSL3	NM_207517
57282	SLC4A10	NM_022058
57337	SENP7	NM_020654
57406	ABHD6	NM_020676
57478	USP31	NM_020718
57484	RNF150	NM_020724
57511	COG6	NM_020751
57512	GPR158	NM_020752
57520	HECW2	NM_020760
57526	PCDH19	NM_020766
57531	HACE1	NM_020771
57536	KIAA1328	NM_020776

57537	SORCS2	NM_020777
57544	TXNDC16	NM_020784
57552	AADACL1	NM_020792
57554	LRRC7	NM_020794
57560	IFT80	NM_020800
57578	KIAA1409	NM_020818
57579	FAM135A	NM_020819
57589	KIAA1432	NM_020829
57619	SHROOM3	NM_020859
57620	STIM2	NM_020860
57623	ZFAT	NM_001029939 NM_020863
57626	KLHL1	NM_020866
57628	DPP10	NM_001004360 NM_020868
57639	CCDC146	NM_020879
57653	KIAA1529	NM_020893
57669	EPB41L5	NM_020909
57689	LRRC4C	NM_020929
57698	KIAA1598	NM_018330
57706	DENND1A	NM_020946 NM_024820
57728	WDR19	NM_025132
57821	C1orf114	NM_021179
58499	ZNF462	NM_021224
59277	NTN4	NM_021229
59350	RXFP1	NM_021634
60412	EXOC4	NM_001037126 NM_021807
60468	BACH2	NM_021813
60492	CCDC90B	NM_021825
60495	HPSE2	NM_021828
60682	SMAP1	NM_021940
63892	THADA	NM_022065 NM_198554
63917	GALNT11	NM_022087
63982	TMEM16C	NM_031418
64067	NPAS3	NM_022123 NM_173159
64084	CLSTN2	NM_022131
64087	MCCC2	NM_022132
64092	SAMSN1	NM_022136
64097	EPB41L4A	NM_022140
64116	SLC39A8	NM_022154
64168	NECAB1	NM_022351
64224	HERPUD2	NM_022373
64232	MS4A5	NM_023945
64326	RFWD2	NM_001001740 NM_022457
64327	LMBR1	NM_022458
64374	SIL1	NM_001037633 NM_022464
64393	ZMAT3	NM_022470 NM_152240
64478	CSMD1	NM_033225
64754	SMYD3	NM_022743
64762	FAM59A	NM_022751
64799	IQCH	NM_001031715 NM_022784
64839	FBXL17	NM_022824
64864	RFXDC2	NM_022841
64901	RANBP17	NM_022897

64902	AGXT2	NM_031900
64969	MRPS5	NM_031902
65084	TMEM135	NM_022918
65217	PCDH15	NM_033056
65975	STK33	NM_030906
66000	TMEM108	NM_023943
66037	BOLL	NM_033030 NM_197970
79071	ELOVL6	NM_024090
79172	CENPO	NM_024322
79175	ZNF343	NM_024325
79582	SPAG16	NM_001025436 NM_024532
79587	CARS2	NM_024537
79589	RNF128	NM_024539 NM_194463
79634	SCRN3	NM_024583
79657	RPAP3	NM_024604
79674	VEPH1	NM_024621
79698	ZMAT4	NM_024645
79710	MORC4	NM_024657
79722	ANKRD55	NM_001039935 NM_024669
79740	ZBBX	NM_024687
79741	C10orf68	NM_024688
79750	ZNF385D	NM_024697
79768	C15orf29	NM_024713
79772	MCTP1	NM_001002796 NM_024717
79781	IQCA	NM_024726
79783	C7orf10	NM_024728
79799	UGT2A3	NM_024743
79807	GSTCD	NM_001031720 NM_024751
79815	NPAL2	NM_024759
79820	CATSPERB	NM_024764
79823	C2orf34	NM_024766
79828	METTL8	NM_024770
79858	NEK11	NM_024800 NM_145910
79895	ATP8B4	NM_024837
79908	BTNL8	NM_024850
79925	SPEF2	NM_024867 NM_144722
79937	CNTNAP3	NM_033655
79953	C20orf39	NM_024893
79970	ZNF767	NM_024910
79974	C7orf58	NM_024913
79983	POF1B	NM_024921
80055	PGAP1	NM_024989
80070	ADAMTS20	NM_025003 NM_175851
80071	CCDC15	NM_025004
80144	FRAS1	NM_025074
80146	UXS1	NM_025076
80157	FLJ21511	NM_025087
80224	NUBPL	NM_025152
80258	EFHC2	NM_025184
80309	SPHKAP	NM_030623
80321	CEP70	NM_024491
80333	KCNIP4	NM_001035003 NM_001035004 NM_025221 NM_147181 NM_147182 NM_147183

80705	TSGA10	NM_025244 NM_182911
80821	DDHD1	NM_030637
80853	JHDM1D	NM_030647
80856	KIAA1715	NM_030650
81494	CFHR5	NM_030787
81533	ITFG1	NM_030790
81539	SLC38A1	NM_030674
81553	FAM49A	NM_030797
81567	TXNDC5	NM_022085 NM_030810
81578	COL21A1	NM_030820
81608	FIP1L1	NM_030917
81615	TMEM163	NM_030923
81792	ADAMTS12	NM_030955
81846	SBF2	NM_030962
81849	ST6GALNAC5	NM_030965
81931	ZNF93	NM_031218
83259	PCDH11Y	NM_032971 NM_032972 NM_032973
83468	GLT8D2	NM_031302
83478	ARHGAP24	NM_001025616 NM_031305
83539	CHST9	NM_031422
83641	FAM107B	NM_031453
83659	TEKT1	NM_053285
83696	NIBP	NM_031466
83698	CALN1	NM_001017440 NM_031468
83699	SH3BGRL2	NM_031469
83700	JAM3	NM_032801
83734	ATG10	NM_031482
83851	SYT16	NM_031914
83872	HMCN1	NM_031935
83894	TTC29	NM_031956
83938	C10orf11	NM_032024
83943	IMMP2L	NM_032549
83989	C5orf21	NM_032042
84034	EMILIN2	NM_032048
84056	KATNAL1	NM_001014380 NM_032116
84059	GPR98	NM_032119
84062	DTNBP1	NM_032122 NM_183040 NM_183041
84068	SLC10A7	NM_001029998 NM_001030316 NM_032128
84083	ZRANB3	NM_032143
84102	SLC41A2	NM_032148
84146	ZNF644	NM_016620 NM_032186 NM_201269
84187	TMEM164	NM_032227
84216	TMEM117	NM_032256
84239	ATP13A4	NM_032279
84280	BTBD10	NM_032320
84303	CHCHD6	NM_032343
84498	FAM120B	NM_032448
84530	KIAA1853	NM_194286
84539	MCHR2	NM_001040179 NM_032503
84570	COL25A1	NM_032518 NM_198721
84620	ST6GAL2	NM_032528
84679	SLC9A7	NM_032591

84708	LNX1	NM_032622
84766	EFCAB4B	NM_032680
84791	C1orf97	NM_032705
84871	AGBL4	NM_032785
84873	GPR128	NM_032787
84900	RNFT2	NM_032814
84946	LTV1	NM_032860
84953	MICALCL	NM_032867
84955	NUDCD1	NM_032869
84969	TOX2	NM_032883
84978	FRMD5	NM_032892
85413	SLC22A16	NM_033125
85417	CCNB3	NM_033031 NM_033670
85458	DIXDC1	NM_001037954 NM_033425
89846	FGD3	NM_033086
89874	SLC25A21	NM_030631
89876	C3orf15	NM_033364
89978	ATPBD4	NM_080650
90025	UBE2CBP	NM_198920
90293	KLHL13	NM_033495
91050	CCDC149	NM_173463
91133	L3MBTL4	NM_173464
91147	TMEM67	NM_153704
91431	LOC91431	NM_138698
91526	ANKRD44	NM_153697
91687	CENPL	NM_033319
91752	ZNF804A	NM_194250
92346	C1orf105	NM_139240
92369	SPSB4	NM_080862
92454	PRR8	NM_053043
92565	FANK1	NM_145235
92675	DTD1	NM_080820
92949	ADAMTSL1	NM_052866 NM_139238 NM_139264
93035	PKHD1L1	NM_177531
93273	LEMD1	NM_001001552
93492	TPTE2	NM_130785 NM_199254
93627	MGC16169	NM_033115
93664	CADPS2	NM_001009571 NM_017954
96459	FNIP1	NM_001008738 NM_133372
112609	C6orf117	NM_138409
113201	CASC4	NM_138423 NM_177974
114134	SLC2A13	NM_052885
114299	PALM2	NM_001037293 NM_053016
114327	EFHC1	NM_018100
114569	MAL2	NM_052886
114784	CSMD2	NM_052896
114786	XKR4	NM_052898
114788	CSMD3	NM_052900 NM_198123 NM_198124
114792	KLHL32	NM_052904
114805	GALNT13	NM_052917
114815	SORCS1	NM_001013031 NM_052918
114836	SLAMF6	NM_052931

114880	OSBPL6	NM_032523 NM_145739
114908	TMEM123	NM_052932
115111	SLC26A7	NM_052832 NM_134266
115123	MARCH3	NM_178450
115286	SLC25A26	NM_173471
115350	FCRL1	NM_052938
115825	WDFY2	NM_052950
115827	RAB3C	NM_138453
116150	NUS1	NM_138459
116328	C8orf34	NM_052958
116496	FAM129A	NM_052966
116931	MED12L	NM_053002
117154	DACH2	NM_053281
117177	RAB3IP	NM_001024647 NM_022456 NM_175623 NM_175624 NM_175625
117245	HRASLS5	NM_054108
117531	TMC1	NM_138691
117583	PARD3B	NM_057177 NM_152526 NM_205863
118429	ANTXR2	NM_058172
118491	TTC18	NM_145170
118611	C10orf90	NM_001004298
120103	SLC36A4	NM_152313
120400	FAM55A	NM_152315
120406	FAM55B	NM_182495
120935	CCDC38	NM_182496
121256	TMEM132D	NM_133448
121601	TMEM16D	NM_178826
122046	C13orf26	NM_152325
123355	LRRC28	NM_144598
123591	C15orf27	NM_152335
124149	FLJ43980	NM_001004299
125228	C18orf19	NM_152352
126204	NLRP13	NM_176810
126859	C1orf125	NM_144696 NM_182766
128153	SPATA17	NM_138796
128553	TSHZ2	NM_173485
128646	SIRPD	NM_178460
128954	GAB4	NM_001037814
129563	DIS3L2	NM_152383
129642	MBOAT2	NM_138799
129684	CNTNAP5	NM_130773 NM_138996
130132	RFTN2	NM_144629
130271	PLEKHH2	NM_172069
130399	ACVR1C	NM_145259
130540	ALS2CR12	NM_139163
130574	LYPD6	NM_194317
130940	CCDC148	NM_138803
131034	CPNE4	NM_130808
131096	KCNH8	NM_144633
131544	DKFZp667G2110	NM_153605
131566	DCBLD2	NM_080927
132320	SCLT1	NM_144643
132612	ADAD1	NM_139243

132671	SPATA18	NM_145263
132884	EVC2	NM_147127
132949	AASDH	NM_181806
133015	C4orf28	NM_145048
133121	ENPP6	NM_153343
133482	SLCO6A1	NM_173488
133558	FLJ40243	NM_173489
133690	CAPSL	NM_144647
134359	C5orf37	NM_152408
135138	PACRG	NM_152410
135152	B3GAT2	NM_080742
136332	LRGUK	NM_144648
137492	VPS37A	NM_152415
137868	SGCZ	NM_139167
138046	RALYL	NM_173848
138412	LOC138412	-
138639	PTPDC1	NM_152422 NM_177995
139221	MUM1L1	NM_152423
139322	APOOL	NM_198450
139324	HDX	NM_144657
139411	PTCHD1	NM_173495
139466	LOC139466	-
140469	MYO3B	NM_138995
140609	NEK7	NM_133494
140733	MACROD2	NM_001033086 NM_001033087 NM_080676
143279	HECTD2	NM_173497 NM_182765
143425	SYT9	NM_175733
143884	CWF19L2	NM_152434
144402	CPNE8	NM_153634
144577	FLJ32549	NM_152440
145173	B3GALTL	NM_194318
145282	MIPOL1	NM_138731
145389	SLC38A6	NM_153811
145407	C14orf37	NM_001001872
145508	C14orf145	NM_152446
145581	LRFN5	NM_152447
145773	FAM81A	NM_152450
145957	NRG4	NM_138573
146057	TTBK2	NM_173500
146845	WDR16	NM_001037306 NM_145054
148418	SAMD13	NM_001010971
148534	TMEM56	NM_152487
148641	SLC35F3	NM_173508
148823	C1orf150	NM_145278
148867	SLC30A7	NM_133496
149233	IL23R	NM_144701
149297	FAM78B	NM_001017961
149628	PYHIN1	NM_152501 NM_198928 NM_198929 NM_198930
150159	NHEDC1	NM_139173
150465	TTL	NM_153712
150472	CBWD2	NM_172003
150596	FLJ32955	NM_153041

151126	ZNF385B	NM_152520
151246	SGOL2	NM_152524
151254	ALS2CR11	NM_152525
151393	FAM82A	NM_144713
151531	UPP2	NM_173355
151647	FAM19A4	NM_001005527 NM_182522
151790	WDR49	NM_178824
152002	C3orf21	NM_152531
152028	FNDC6	NM_144717
152185	CCDC52	NM_144718
152189	CMTM8	NM_178868
152330	CNTN4	NM_175607 NM_175612 NM_175613
152404	IGSF11	NM_001015887 NM_152538
152579	SCFD2	NM_152540
152940	FLJ25371	NM_152543
153218	SPINK5L3	NM_001040129
154215	NKAIN2	NM_001040214
154661	RUNDC3B	NM_138290
154664	ABCA13	NM_152701
154743	FLJ31818	NM_152556
157376	C8orf78	NM_182525
157680	VPS13B	NM_015243 NM_017890 NM_152564 NM_181661
157807	RLBP1L1	NM_173519
158038	LINGO2	NM_152570
158158	RASEF	NM_152573
158297	FAM154A	NM_153707
158326	FREM1	NM_144966
158584	FAAH2	NM_174912
160140	C11orf65	NM_152587
160335	TMTC2	NM_152588
160492	IFLTD1	NM_152590
160518	MGC24039	NM_144973
160728	SLC5A8	NM_145913
160777	CCDC60	NM_178499
161357	MDGA2	NM_182830
161725	OTUD7A	NM_130901
162282	ANKFN1	NM_153228
162517	FBXO39	NM_153230
163081	ZNF567	NM_152603
163131	ZNF780B	NM_001005851
163486	DENND1B	NM_144977
163589	TDRD5	NM_173533
166336	PRICKLE2	NM_198859
166378	SPATA5	NM_145207
167359	MGC42105	NM_153361
168090	C6orf118	NM_144980
168667	BMPER	NM_133468
168975	CNBD1	NM_173538
169044	COL22A1	NM_152888
170691	ADAMTS17	NM_139057
170712	COX7B2	NM_130902
171019	ADAMTS19	NM_133638

196074	METT5D1	NM_152636
196296	DCDC5	NM_198462
196446	C12orf28	NM_182530
196527	TMEM16F	NM_001025356
196792	FAM24B	NM_152644
196951	C15orf33	NM_152647
200150	PLD5	NM_152666
200162	SPAG17	NM_206996
200420	ALMS1P	NM_145300
200844	C3orf67	NM_198463
202333	CMYA5	NM_153610
202374	STK32A	NM_145001
202559	KHDRBS2	NM_152688
203102	ADAM32	NM_145004
203238	C9orf93	NM_173550
203427	SLC25A43	NM_145305
203447	NRK	NM_198465
204219	LASS3	NM_178842
204801	NLRP11	NM_145007
204962	SLC44A5	NM_152697
206938	C9orf94	NM_001040272
219578	ZNF804B	NM_181646
219771	CCNY	NM_145012 NM_181698
220032	GDPD4	NM_182833
220107	DLEU7	NM_198989
220115	LOC220115	NR_002793
220416	RP11-139H14.4	NM_001024609
220869	CBWD5	NM_001024916
221016	CCDC7	NM_001026383 NM_145023
221061	C10orf38	NM_001010924
221074	SLC39A12	NM_152725
221078	NSUN6	NM_182543
221143	N6AMT2	NM_174928
221264	C6orf199	NM_145025
221294	NT5DC1	NM_152729
221302	ZUFSP	NM_145062
221322	C6orf170	NM_152730
221458	KIF6	NM_145027
221895	JAZF1	NM_175061
222234	MGC35361	NM_147194
222235	FBXL13	NM_145032
223075	CCDC129	NM_194300
246119	TTTY10	NR_001542
246126	CYorf15A	NM_001005852
246269	LACE1	NM_145315
253260	RICTOR	NM_152756
253430	IPMK	NM_152230
253559	CADM2	NM_153184
253582	C6orf191	NM_001010876
253769	WDR27	NM_182552
253782	LASS6	NM_203463
253827	MSRB3	NM_001031679 NM_198080

254065	BRWD3	NM_153252
254251	LCORL	NM_153686
254827	NAALADL2	NM_207015
255119	C4orf22	NM_152770
255631	COL24A1	NM_152890
255928	SYT14	NM_153262
256435	ST6GALNAC3	NM_152996
256643	CXorf23	NM_198279
256691	MAMDC2	NM_153267
256764	WDR72	NM_182758
257019	FRMD3	NM_174938
257044	C1orf101	NM_173807
257068	PLCXD2	NM_153268
257194	NEGR1	NM_173808
259239	WFDC11	NM_147197
260425	MAGI3	NM_020965 NM_152900
266695	DKFZp686A1627	NR_002801
266722	HS6ST3	NM_153456
280664	WFDC10B	NM_172006 NM_172131
282809	WDR51B	NM_172240
283208	P4HA3	NM_182904
283209	PGM2L1	NM_173582
283316	CD163L1	NM_174941
283417	DPY19L2	NM_173812
283455	KSR2	NM_173598
283461	C12orf40	NM_001031748
283755	LOC283755	NM_001024682
283777	FLJ39743	NM_182562
284521	OR2L13	NM_175911
284525	SLC9A11	NM_178527
285154	C2orf58	NM_173652
285195	SLC9A9	NM_173653
285216	LOC285216	-
285282	RABL3	NM_173825
285331	CCDC66	NM_001012506
285335	SLC9A10	NM_183061
285362	SUMF1	NM_182760
285386	TPRG1	NM_198485
285555	C4orf37	NM_174952
285596	FAM153A	NM_173663
285600	C5orf36	NM_173665
285754	FLJ37396	NM_001039527
286046	XKR6	NM_173683
286053	NSMCE2	NM_173685
286183	NKAIN3	NM_173688
286205	C9orf126	NM_173690
286239	LOC286239	-
286451	YIPF6	NM_173834
286464	CXorf59	NM_173695
286554	BCORL2	NR_002923
317761	C14orf39	NM_174978
327658	HDHD1BP	-

337876	CHSY3	NM_175856
338645	LUZP2	NM_001009909
338811	FAM19A2	NM_178539
338821	LST-3TM12	NM_001009562
339416	ANKRD45	NM_198493
339479	FAM5C	NM_199051
339500	ZNF678	NM_178549
339883	C3orf35	NM_178339 NM_178342
340267	COL28A1	NM_001037763
340419	RSPO2	NM_178565
340441	A26A1	NM_001002920 NM_001005365
340481	ZDHHC21	NM_178566
340533	KIAA2022	NM_001008537
340595	ZCCHC16	NM_001004308
340811	AKR1CL1	NM_001007536
341019	DCDC1	NM_181807
341640	FREM2	NM_207361
343450	KCNT2	NM_198503
344387	CDKL4	NM_001009565
344758	GPR149	NM_001038705
345557	PLCXD3	NM_001005473
345757	TMEM157	NM_198507
347365	ITIH5L	NM_198510
347404	LANCL3	NM_198511
347613	PARP4P	-
347732	CATSPER3	NM_178019
348158	ACSM2B	NM_182617
348808	LOC348808	NR_002811
348825	TPRXL	NR_002223
348980	HCN1	NM_021072
349152	DPY19L2P2	NM_182634
349565	NMNAT3	NM_178177
353299	RGSL1	NM_181572
359791	LOC359791	-
360021	PPP1R12BP	-
374467	C12orf63	NM_198520
374470	C12orf42	NM_198521
374618	TEX9	NM_198524
374654	KIF7	NM_198525
374864	C18orf34	NM_198995
374868	ATP9B	NM_198531
374992	SEC63D1	NM_198550
375484	C5orf25	NM_198567
375519	GJB7	NM_198568
375612	LHFPL3	NM_199000
378955	RBMY2JP	-
386617	KCTD8	NM_198353
386695	OFDYP11	-
387601	SLC22A25	NM_199352
387694	SH2D4B	NM_207372
387700	SLC16A12	NM_213606
388646	GBP7	NM_207398

388649	C1orf146	NM_001012425
388650	FAM69A	NM_001006605
388815	C21orf34	NM_001005732 NM_001005733 NM_001005734
389170	LEKR1	NM_001004316
389400	GFRAL	NM_207410
389634	LOC389634	NM_001012988
389668	XKR9	NM_001011720
389840	MAP3K15	NM_001001671
392232	LOC392232	-
392636	TMEM195	NM_001004320
399694	SHC4	NM_203349
401013	FLJ34870	NM_207481
401145	MGC48628	NM_207491
401191	FLJ46010	NM_001001703
401252	LOC401252	NM_001013681
401337	FLJ45974	NM_001001707
401398	LOC401398	NM_001023566
401474	SAMD12	NM_207506
401541	CENPP	NM_001012267
401612	MCART6	NM_001012755
401967	NBPF17P	-
404672	GTF2H5	NM_207118
404734	ANKHD1-EIF4EBP3	NM_020690
404744	AAA1	NM_207283 NM_207284 NM_207285 NM_207286 NM_207287 NM_207288 NM_207289 [illegible]
407738	FAM19A1	NM_213609
414753	LOC414753	-
440867	FLJ16124	NM_001004345
441024	MTHFD2L	NM_001004346
441116	FLJ16171	NM_001004348
441644	LOC441644	-
441732	LOC441732	-
442117	GALNT17	NM_001034845
442862	PRY2	NM_001002758
445571	CBWD3	NM_201453
445815	PALM2-AKAP2	NM_007203 NM_147150
497258	BDNFOS	NR_002832
503497	MS4A13	NM_001012417
554236	DPY19L2P1	NR_002833
619279	ZNF704	NM_001033723
642406	LOC642406	-
642484	FLJ45743	NM_001039759
643199	LOC643199	-
643707	LOC643707	-
643789	LOC643789	-
644094	LOC644094	-
644335	LOC644335	-
644780	LOC644780	-
645272	LOC645272	-
645840	TR2IT1	NM_001039783
648283	LOC648283	-
649024	LOC649024	-
650666	LOC650666	-

653510	LOC653510	-
654463	FER1L6	NM_001039112

Table 1.2 Genes with sense L1s (336 genes).

NCBI gene ID	NCBI gene name	mRNA accession
40	ACCN1	NM_001094 NM_183377
225	ABCD2	NM_005164
238	ALK	NM_004304
288	ANK3	NM_001149 NM_020987
313	AOAH	NM_001637
411	ARSB	NM_000046 NM_198709
472	ATM	NM_000051 NM_138292
545	ATR	NM_001184
546	ATRX	NM_000489 NM_138270 NM_138271
549	AUH	NM_001698
665	BNIP3L	NM_004331
667	DST	NM_001723 NM_015548 NM_020388 NM_183380
814	CAMK4	NM_001744
817	CAMK2D	NM_001221 NM_172115 NM_172127 NM_172128
952	CD38	NM_001775
1012	CDH13	NM_001257
1015	CDH17	NM_004063
1161	ERCC8	NM_000082 NM_001007233 NM_001007234
1378	CR1	NM_000573 NM_000651
1432	MAPK14	NM_001315 NM_139012 NM_139013 NM_139014
1501	CTNND2	NM_001332
1558	CYP2C8	NM_000770
1562	CYP2C18	NM_000772
1577	CYP3A5	NM_000777
1666	DECR1	NM_001359
1794	DOCK2	NM_004946
2070	EYA4	NM_004100 NM_172103 NM_172105
2099	ESR1	NM_000125
2257	FGF12	NM_004113 NM_021032
2330	FMO5	NM_001461
2509	FTHP1	-
2515	ADAM2	NM_001464
2890	GRIA1	NM_000827
2892	GRIA3	NM_000828 NM_007325 NM_181894
2977	GUCY1A2	NM_000855
3096	HIVEP1	NM_002114
3176	HNMT	NM_001024074 NM_001024075 NM_006895
3482	IGF2R	NM_000876
3574	IL7	NM_000880
3717	JAK2	NM_004972
3781	KCNN2	NM_021614 NM_170775
4017	LOXL2	NM_002318
4052	LTBP1	NM_000627 NM_206943
4126	MANBA	NM_005908
4133	MAP2	NM_001039538 NM_002374 NM_031845 NM_031847
4208	MEF2C	NM_002397
4223	MEOX2	NM_005924
4306	NR3C2	NM_000901

4593	MUSK	NM_005592
4642	MYO1D	NM_015194
4644	MYO5A	NM_000259
4649	MYO9A	NM_006901
4703	NEB	NM_004543
4704	NDUFA9	NM_005002
4772	NFATC1	NM_006162 NM_172387 NM_172388 NM_172389 NM_172390
4897	NRCAM	NM_001037132 NM_001037133 NM_005010
4921	DDR2	NM_001014796 NM_006182
4988	OPRM1	NM_000914 NM_001008503 NM_001008504 NM_001008505
5126	PCSK2	NM_002594
5139	PDE3A	NM_000921
5156	PDGFRA	NM_006206
5194	PEX13	NM_002618
5243	ABCB1	NM_000927
5332	PLCB4	NM_000933 NM_182797
5334	PLCL1	NM_006226
5412	UBL3	NM_007106
5523	PPP2R3A	NM_002718 NM_181897
5638	PRRG1	NM_000950
5682	PSMA1	NM_002786 NM_148976
5738	PTGFRN	NM_020440
5789	PTPRD	NM_002839 NM_130391 NM_130392 NM_130393
5834	PYGB	NM_002862
5906	RAP1A	NM_001010935 NM_002884
5910	RAP1GDS1	NM_021159
5924	RASGRF2	NM_006909
5925	RB1	NM_000321
5983	RFC3	NM_002915 NM_181558
6000	RGS7	NM_002924
6119	RPA3	NM_002947
6263	RYR3	NM_001036
6342	SCP2	NM_001007098 NM_001007099 NM_001007100 NM_001007250 NM_002979
6505	SLC1A1	NM_004170
6546	SLC8A1	NM_021097
6549	SLC9A2	NM_003048
6581	SLC22A3	NM_021977
6660	SOX5	NM_006940 NM_152989 NM_178010
6695	SPOCK1	NM_004598
6786	STIM1	NM_003156
6857	SYT1	NM_005639
6955	TRA@	-
7223	TRPC4	NM_016179
7253	TSHR	NM_000369 NM_001018036
7267	TTC3	NM_001001894 NM_003316
7299	TYR	NM_000372
7373	COL14A1	NM_021110
7399	USH2A	NM_007123 NM_206933
7404	UTY	NM_007125 NM_182659 NM_182660
7587	ZNF37A	NM_001007094 NM_003421
7620	ZNF69	NM_021915
7626	ZNF75	NM_007131

7827	NPHS2	NM_014625
8038	ADAM12	NM_003474 NM_021641
8139	GAN	NM_022041
8455	ATRN	NM_139321 NM_139322
8502	PKP4	NM_001005476 NM_003628
8516	ITGA8	NM_003638
8577	TMEFF1	NM_003692
8601	RGS20	NM_003702 NM_170587
8618	CADPS	NM_003716 NM_183393 NM_183394
8671	SLC4A4	NM_003759
8685	MARCO	NM_006770
8708	B3GALT1	NM_020981
8745	ADAM23	NM_003812
8874	ARHGEF7	NM_003899 NM_145735
8898	MTMR2	NM_016156 NM_201278 NM_201281
8924	HERC2	NM_004667
8925	HERC1	NM_003922
8935	SKAP2	NM_003930
8942	KYNU	NM_001032998 NM_003937
9014	TAF1B	NM_005680
9173	IL1RL1	NM_003856 NM_016232
9194	SLC16A7	NM_004731
9201	DCLK1	NM_004734
9229	DLGAP1	NM_001003809 NM_004746
9508	ADAMTS3	NM_014243
9577	BRE	NM_004899 NM_199191 NM_199192 NM_199193 NM_199194
9649	RALGPS1	NM_014636
9666	DZIP3	NM_014648
9702	CEP57	NM_014679
9705	ST18	NM_014682
9723	SEMA3E	NM_012431
9743	RICS	NM_014715
9765	ZFYVE16	NM_014733
9786	KIAA0586	NM_014749
9811	KIAA0427	NM_014772
9832	JAKMIP2	NM_014790
9871	SEC24D	NM_014822
9892	SNAP91	NM_014841
9938	ARHGAP25	NM_001007231 NM_014882
9942	XYLB	NM_005108
9990	SLC12A6	NM_005135
10098	TSPAN5	NM_005723
10207	INADL	NM_176877
10231	RCAN2	NM_005822
10242	KCNMB2	NM_005832 NM_181361
10350	ABCA9	NM_080283
10463	SLC30A9	NM_006345
10513	APPBP2	NM_006380
10558	SPTLC1	NM_006415 NM_178324
10800	CYSLTR1	NM_006639
10973	ASCC3	NM_006828 NM_022091
11001	SLC27A2	NM_003645

11005	SPINK5	NM_006846
11061	LECT1	NM_001011705 NM_007015
11124	FAF1	NM_007051 NM_131917
11141	IL1RAPL1	NM_014271
11148	HHLA2	NM_007072
11231	SEC63	NM_007214
23049	SMG1	NM_015092
23132	RAD54L2	NM_015106
23167	EFR3A	NM_015137
23239	PHLPP	NM_194449
23242	COBL	NM_015198
23287	AGTPBP1	NM_015239
23431	AP4E1	NM_007347
23604	DAPK2	NM_014326
23643	LY96	NM_015364
25769	SLC24A2	NM_020344
25849	DKFZP564O0823	NM_015393
25963	TMEM87A	NM_015497
26053	AUTS2	NM_015570
26122	EPC2	NM_015630
26133	TRPC4AP	NM_015638 NM_199368
26154	ABCA12	NM_015657 NM_173076
26610	ELP4	NM_019040
26750	RPS6KC1	NM_012424
26958	COPG2	NM_012133
27115	PDE7B	NM_018945
27159	CHIA	NM_021797 NM_201653
27252	KLHL20	NM_014458
27255	CNTN6	NM_014461
27283	TINAG	NM_014464
27306	PGDS	NM_014485
29767	TMOD2	NM_014548
29922	NME7	NM_013330 NM_197972
29957	SLC25A24	NM_013386 NM_213651
50485	SMARCAL1	NM_014140
50863	HNT	NM_016522
51170	HSD17B11	NM_016245
51265	CDKL3	NM_016508
51606	ATP6V1H	NM_015941 NM_213619 NM_213620
51705	EMCN	NM_016242
54503	ZDHHC13	NM_001001483 NM_019028
54714	CNGB3	NM_019098
54842	FLJ20160	NM_017694
54882	ANKHD1	NM_017747 NM_017978 NM_024668
54916	C14orf101	NM_017799
54954	FAM120C	NM_017848
55016	MARCH1	NM_017923
55031	USP47	NM_017944
55076	TMEM45A	NM_018004
55103	RALGPS2	NM_018037 NM_152663
55165	CEP55	NM_018131
55275	VPS53	NM_018289

55289	ACOXL	NM_018308
55296	TBC1D19	NM_018317
55356	SLC22A15	NM_018420
55423	SIRPG	NM_001039508 NM_018556 NM_080816
55504	TNFRSF19	NM_018647 NM_148957
55567	DNAH3	NM_017539
55610	CCDC132	NM_017667 NM_024553
55711	MLSTD1	NM_018099
55744	C7orf44	NM_018224
56062	KLHL4	NM_019117 NM_057162
56895	AGPAT4	NM_020133
56957	OTUD7B	NM_020205
57094	CPA6	NM_020361
57103	C12orf5	NM_020375
57188	ADAMTSL3	NM_207517
57511	COG6	NM_020751
57520	HECW2	NM_020760
57552	AADACL1	NM_020792
57619	SHROOM3	NM_020859
57698	KIAA1598	NM_018330
57728	WDR19	NM_025132
63892	THADA	NM_022065 NM_198554
64067	NPAS3	NM_022123 NM_173159
64224	HERPUD2	NM_022373
64232	MS4A5	NM_023945
64374	SIL1	NM_001037633 NM_022464
79750	ZNF385D	NM_024697
79781	IQCA	NM_024726
79820	CATSPERB	NM_024764
79828	METTL8	NM_024770
79925	SPEF2	NM_024867 NM_144722
79970	ZNF767	NM_024910
79983	POF1B	NM_024921
80853	JHDM1D	NM_030647
81539	SLC38A1	NM_030674
81553	FAM49A	NM_030797
81615	TMEM163	NM_030923
83539	CHST9	NM_031422
83641	FAM107B	NM_031453
83659	TEKT1	NM_053285
83700	JAM3	NM_032801
84102	SLC41A2	NM_032148
84280	BTBD10	NM_032320
84303	CHCHD6	NM_032343
84498	FAM120B	NM_032448
84539	MCHR2	NM_001040179 NM_032503
84766	EFCAB4B	NM_032680
84969	TOX2	NM_032883
85458	DIXDC1	NM_001037954 NM_033425
89874	SLC25A21	NM_030631
89876	C3orf15	NM_033364
89978	ATPBD4	NM_080650

90293	KLHL13	NM_033495
92346	C1orf105	NM_139240
92565	FANK1	NM_145235
93273	LEMD1	NM_001001552
96459	FNIP1	NM_001008738 NM_133372
113201	CASC4	NM_138423 NM_177974
114569	MAL2	NM_052886
114786	XKR4	NM_052898
114815	SORCS1	NM_001013031 NM_052918
114880	OSBPL6	NM_032523 NM_145739
115123	MARCH3	NM_178450
116150	NUS1	NM_138459
116931	MED12L	NM_053002
120103	SLC36A4	NM_152313
120935	CCDC38	NM_182496
128646	SIRPD	NM_178460
130132	RFTN2	NM_144629
136332	LRGUK	NM_144648
139466	LOC139466	-
145957	NRG4	NM_138573
146845	WDR16	NM_001037306 NM_145054
149628	PYHIN1	NM_152501 NM_198928 NM_198929 NM_198930
151254	ALS2CR11	NM_152525
152189	CMTM8	NM_178868
158158	RASEF	NM_152573
158297	FAM154A	NM_153707
158326	FREM1	NM_144966
160518	MGC24039	NM_144973
162282	ANKFN1	NM_153228
162517	FBXO39	NM_153230
163131	ZNF780B	NM_001005851
170712	COX7B2	NM_130902
196446	C12orf28	NM_182530
196527	TMEM16F	NM_001025356
200162	SPAG17	NM_206996
202374	STK32A	NM_145001
219771	CCNY	NM_145012 NM_181698
220032	GDPD4	NM_182833
220107	DLEU7	NM_198989
221143	N6AMT2	NM_174928
222234	MGC35361	NM_147194
246269	LACE1	NM_145315
253559	CADM2	NM_153184
253782	LASS6	NM_203463
254251	LCORL	NM_153686
256643	CXorf23	NM_198279
266695	DKFZp686A1627	NR_002801
280664	WFDC10B	NM_172006 NM_172131
283208	P4HA3	NM_182904
283455	KSR2	NM_173598
283755	LOC283755	NM_001024682
284525	SLC9A11	NM_178527

285216	LOC285216	-
285282	RABL3	NM_173825
285362	SUMF1	NM_182760
286046	XKR6	NM_173683
286554	BCORL2	NR_002923
337876	CHSY3	NM_175856
339416	ANKRD45	NM_198493
339479	FAM5C	NM_199051
340811	AKR1CL1	NM_001007536
341019	DCDC1	NM_181807
347365	ITIH5L	NM_198510
348158	ACSM2B	NM_182617
359791	LOC359791	-
374618	TEX9	NM_198524
389170	LEKR1	NM_001004316
389634	LOC389634	NM_001012988
399694	SHC4	NM_203349
401967	NBPF17P	-
404734	ANKHD1-EIF4EBP3	NM_020690
441116	FLJ16171	NM_001004348
441644	LOC441644	-
497258	BDNFOS	NR_002832
503497	MS4A13	NM_001012417
642484	FLJ45743	NM_001039759
643199	LOC643199	-
645272	LOC645272	-
645840	TR2IT1	NM_001039783
650666	LOC650666	-

Table 1.3 Genes with antisense L1s (832 genes).

NCBI gene ID	NCBI gene name	mRNA accession
22	ABCB7	NM_004299
245	ALOX12P2	NR_002710
329	BIRC2	NM_001166
351	APP	NM_000484 NM_201413 NM_201414
367	AR	NM_000044 NM_001011645
540	ATP7B	NM_000053 NM_001005918
577	BAI3	NM_001704
644	BLVRA	NM_000712
658	BMPR1B	NM_001203
729	C6	NM_000065
735	C9	NM_001737
753	C18orf1	NM_001003674 NM_001003675 NM_004338 NM_181481 NM_181482 NM_181483
840	CASP7	NM_001227 NM_033338 NM_033339 NM_033340
953	ENTPD1	NM_001776
987	LRBA	NM_006726
1006	CDH8	NM_001796
1010	CDH12	NM_004061
1016	CDH18	NM_004934
1047	CLGN	NM_004362
1062	CENPE	NM_001813
1121	CHM	NM_000390
1129	CHRM2	NM_000739 NM_001006626 NM_001006627 NM_001006628 NM_001006629 [illegible]
1130	LYST	NM_000081 NM_001005736
1131	CHRM3	NM_000740
1136	CHRNA3	NM_000743
1139	CHRNA7	NM_000746
1176	AP3S1	NM_001002924 NM_001284
1272	CNTN1	NM_001843 NM_175038
1286	COL4A4	NM_000092
1287	COL4A5	NM_000495 NM_033380 NM_033381
1295	COL8A1	NM_001850 NM_020351
1362	CPD	NM_001304
1496	CTNNA2	NM_004389
1550	CYP2A7P1	-
1557	CYP2C19	NM_000769
1559	CYP2C9	NM_000771
1612	DAPK1	NM_004938
1617	DAZ1	NM_004081
1620	DBC1	NM_014618
1629	DBT	NM_001918
1644	DDC	NM_000790
1770	DNAH9	NM_001372 NM_004662
1780	DYNC1I1	NM_004411
1806	DPYD	NM_000110
1828	DSG1	NM_001942
1837	DTNA	NM_001390 NM_001391 NM_001392 NM_032975 NM_032978 NM_032979 NM_032980 [illegible]
1910	EDNRB	NM_000115 NM_003991
1950	EGF	NM_001963

2042	EPHA3	NM_005233 NM_182644
2047	EPHB1	NM_004441
2060	EPS15	NM_001981
2066	ERBB4	NM_005235
2121	EVC	NM_153717
2176	FANCC	NM_000136
2182	ACSL4	NM_004458 NM_022977
2222	FDFT1	NM_004462
2259	FGF14	NM_004115 NM_175929
2312	FLG	NM_002016
2334	AFF2	NM_002025
2444	FRK	NM_002031
2530	FUT8	NM_004480 NM_178154 NM_178155 NM_178156 NM_178157
2585	GALK2	NM_001001556 NM_002044
2643	GCH1	NM_000161 NM_001024024 NM_001024070 NM_001024071
2719	GPC3	NM_004484
2742	GLRA2	NM_002063
2743	GLRB	NM_000824
2768	GNA12	NM_007353
2805	GOT1	NM_002079
2894	GRID1	NM_017551
2898	GRIK2	NM_021956 NM_175768
2900	GRIK4	NM_014619
2932	GSK3B	NM_002093
3064	HTT	NM_002111
3075	CFH	NM_000186 NM_001014975
3360	HTR4	NM_000870 NM_001040169 NM_001040171 NM_001040172 NM_001040173 [illegible]
3376	IARS	NM_002161 NM_013417
3382	ICA1	NM_004968 NM_022307
3426	CFI	NM_000204
3600	IL15	NM_000585 NM_172174
3617	IMPG1	NM_001563
3673	ITGA2	NM_002203
3680	ITGA9	NM_002207
3684	ITGAM	NM_000632
3688	ITGB1	NM_002211 NM_033666 NM_033667 NM_033668 NM_033669 NM_133376
3694	ITGB6	NM_000888
3709	ITPR2	NM_002223
3760	KCNJ3	NM_002239
3776	KCNK2	NM_001017424 NM_001017425 NM_014217
3784	KCNQ1	NM_000218 NM_181797 NM_181798
3790	KCNS3	NM_002252
3953	LEPR	NM_001003679 NM_001003680 NM_002303
3998	LMAN1	NM_005570
4008	LMO7	NM_005358
4018	LPA	NM_005577
4026	LPP	NM_005578
4063	LY9	NM_001033667 NM_002348
4065	LY75	NM_002349
4128	MAOA	NM_000240
4158	MC2R	NM_000529
4163	MCC	NM_002387

4179	CD46	NM_002389 NM_153826 NM_172350 NM_172351 NM_172352 NM_172353 NM_172354 [illegible]
4233	MET	NM_000245
4253	CTAGE5	NM_005930 NM_203354 NM_203355 NM_203356 NM_203357
4255	MGMT	NM_002412
4281	MID1	NM_000381 NM_033290 NM_033291
4285	MIPEP	NM_005932
4311	MME	NM_000902 NM_007287 NM_007288 NM_007289
4325	MMP16	NM_005941 NM_022564
4331	MNAT1	NM_002431
4332	MNDA	NM_002432
4361	MRE11A	NM_005590 NM_005591
4437	MSH3	NM_002439
4646	MYO6	NM_004999
4659	PPP1R12A	NM_002480
4724	NDUFS4	NM_002495
4734	NEDD4	NM_006154 NM_198400
4756	NEO1	NM_002499
4810	NHS	NM_198270
4848	CNOT2	NM_014515
4867	NPHP1	NM_000272 NM_207181
4940	OAS3	NM_006187
4983	OPHN1	NM_002547
5033	P4HA1	NM_000917 NM_001017962
5053	PAH	NM_000277
5058	PAK1	NM_002576
5101	PCDH9	NM_020403 NM_203487
5128	PCTK2	NM_002595
5136	PDE1A	NM_001003683 NM_005019
5137	PDE1C	NM_005020
5142	PDE4B	NM_001037339 NM_001037340 NM_001037341 NM_002600
5144	PDE4D	NM_006203
5151	PDE8A	NM_002605 NM_173454 NM_173455 NM_173456 NM_173457
5205	ATP8B1	NM_005603
5212	VIT	NM_053276
5241	PGR	NM_000926
5255	PHKA1	NM_002637
5257	PHKB	NM_000293 NM_001031835
5337	PLD1	NM_002662
5340	PLG	NM_000301
5446	PON3	NM_000940
5475	PPEF1	NM_006240 NM_152224 NM_152226
5521	PPP2R2B	NM_004576 NM_181674 NM_181675 NM_181676 NM_181677 NM_181678
5550	PREP	NM_002726
5567	PRKACB	NM_002731 NM_182948 NM_207578
5570	PKIB	NM_032471 NM_181794 NM_181795
5577	PRKAR2B	NM_002736
5578	PRKCA	NM_002737
5586	PKN2	NM_006256
5587	PRKD1	NM_002742
5588	PRKCQ	NM_006257
5593	PRKG2	NM_006259
5611	DNAJC3	NM_006260

5618	PRLR	NM_000949
5627	PROS1	NM_000313
5649	RELN	NM_005045 NM_173054
5728	PTEN	NM_000314
5747	PTK2	NM_005607 NM_153831
5783	PTPN13	NM_006264 NM_080683 NM_080684 NM_080685
5796	PTPRK	NM_002844
5801	PTPRR	NM_002849 NM_130846
5825	ABCD3	NM_002858
5858	PZP	NM_002864
5991	RFX3	NM_002919 NM_134428
6091	ROBO1	NM_002941 NM_133631
6092	ROBO2	NM_002942
6095	RORA	NM_002943 NM_134260 NM_134261 NM_134262
6196	RPS6KA2	NM_001006932 NM_021135
6252	RTN1	NM_021136 NM_206852 NM_206857
6344	SCTR	NM_002980
6399	TRAPPC2	NM_001011658 NM_014563
6444	SGCD	NM_000337 NM_172244
6456	SH3GL2	NM_003026
6457	SH3GL3	NM_003027
6480	ST6GAL1	NM_003032 NM_173216 NM_173217
6565	SLC15A2	NM_021082
6579	SLCO1A2	NM_005075 NM_021094 NM_134431
6586	SLIT3	NM_003062
6683	SPAST	NM_014946 NM_199436
6764	ST5	NM_005418 NM_139157 NM_213618
6769	STAC	NM_003149
6775	STAT4	NM_003151
6854	SYN2	NM_003178 NM_133625
6885	MAP3K7	NM_003188 NM_145331 NM_145332 NM_145333
6905	TBCE	NM_003193
7007	TECTA	NM_005422
7035	TFPI	NM_001032281 NM_006287
7068	THRB	NM_000461
7092	TLL1	NM_012464
7107	GPR137B	NM_003272
7111	TMOD1	NM_003275
7164	TPD52L1	NM_001003395 NM_001003396 NM_001003397 NM_003287
7260	TSSC1	NM_003310
7367	UGT2B17	NM_001077
7402	UTRN	NM_007124
7403	UTX	NM_021140
7405	UVRAG	NM_003369
7424	VEGFC	NM_005429
7757	ZNF208	NM_007153
7762	ZNF215	NM_013250
7768	ZNF225	NM_013362
7770	ZNF227	NM_182490
7851	MALL	NM_005434
7881	KCNAB1	NM_003471 NM_172159 NM_172160
7913	DEK	NM_003472

7957	EPM2A	NM_001018041 NM_005670
7991	TUSC3	NM_006765 NM_178234
8001	GLRA3	NM_006529
8287	USP9Y	NM_004654
8411	EEA1	NM_003566
8452	CUL3	NM_003590
8460	TPST1	NM_003596
8464	SUPT3H	NM_003599 NM_181356
8540	AGPS	NM_003659
8546	AP3B1	NM_003664
8573	CASK	NM_003688
8621	CDC2L5	NM_003718 NM_031267
8654	PDE5A	NM_001083 NM_033430 NM_033437
8732	RNGTT	NM_003800
8756	ADAM7	NM_003817
8801	SUCLG2	NM_003848
8805	TRIM24	NM_003852 NM_015905
8808	IL1RL2	NM_003854
8821	INPP4B	NM_003866
8832	CD84	NM_003874
8854	ALDH1A2	NM_003888 NM_170696 NM_170697
8936	WASF1	NM_001024934 NM_001024935 NM_001024936 NM_003931
9037	SEMA5A	NM_003966
9061	PAPSS1	NM_005443
9079	LDB2	NM_001290
9081	PRY	NM_004676
9162	DGKI	NM_004717
9213	XPR1	NM_004736
9223	MAGI1	NM_001033057 NM_004742 NM_015520
9364	RAB28	NM_001017979 NM_004249
9372	ZFYVE9	NM_004799 NM_007323 NM_007324
9378	NRXN1	NM_004801 NM_138735
9425	CDYL	NM_004824 NM_170751 NM_170752
9457	FHL5	NM_020482
9462	RASAL2	NM_004841 NM_170692
9465	AKAP7	NM_004842 NM_016377 NM_138633
9474	ATG5	NM_004849
9522	SCAMP1	NM_004866 NM_052822
9562	MINPP1	NM_004897
9568	GABBR2	NM_005458
9576	SPAG6	NM_012443 NM_172242
9586	CREB5	NM_001011666 NM_004904 NM_182898 NM_182899
9627	SNCAIP	NM_005460
9628	RGS6	NM_004296
9657	IQCB1	NM_001023570 NM_001023571
9659	PDE4DIP	NM_001002810 NM_001002811 NM_001002812 NM_014644 NM_022359
9692	KIAA0391	NM_014672
9722	NOS1AP	NM_014697
9730	VPRBP	NM_014703
9732	DOCK4	NM_014705
9734	HDAC9	NM_014707 NM_058176 NM_058177 NM_178423 NM_178425
9749	PHACTR2	NM_014721

9760	TOX	NM_014729
9843	HEPH	NM_014799 NM_138737
9873	FCHSD2	NM_014824
9899	SV2B	NM_014848
9910	RABGAP1L	NM_001035230 NM_014857
9934	P2RY14	NM_014879
9958	USP15	NM_006313
10000	AKT3	NM_005465 NM_181690
10010	TANK	NM_004180 NM_133484
10056	FARSB	NM_005687
10060	ABCC9	NM_005691 NM_020297 NM_020298
10085	EDIL3	NM_005711
10090	UST	NM_005715
10144	FAM13A1	NM_001015045 NM_014883
10165	SLC25A13	NM_014251
10240	MRPS31	NM_005830
10257	ABCC4	NM_005845
10283	SDCCAG10	NM_005869
10308	ZNF267	NM_003414
10314	LANCL1	NM_006055
10345	TRDN	NM_006073
10352	WARS2	NM_015836 NM_201263
10367	CBARA1	NM_006077
10392	NOD1	NM_006092
10406	WFDC2	NM_006103 NM_080734 NM_080735 NM_080736
10418	SPON1	NM_006108
10464	PIBF1	NM_006346
10466	COG5	NM_006348 NM_181733
10495	ENOX2	NM_006375 NM_182314
10497	UNC13B	NM_006377
10529	NEBL	NM_006393 NM_213569
10563	CXCL13	NM_006419
10564	ARFGEF2	NM_006420
10599	SLCO1B1	NM_006446
10643	IGF2BP3	NM_006547
10651	MTX2	NM_001006635 NM_006554
10667	FARS2	NM_006567
10718	NRG3	NM_001010848
10721	POLQ	NM_199420
10752	CHL1	NM_006614
10846	PDE10A	NM_006661
10873	ME3	NM_001014811 NM_006680
10874	NMU	NM_006681
10877	CFHR4	NM_006684
10878	CFHR3	NM_021023
10888	GPR83	NM_016540
10941	UGT2A1	NM_006798
11027	LILRA2	NM_006866
11036	GTF2A1L	NM_006872 NM_172196
11055	ZPBP	NM_007009
11107	PRDM5	NM_018699
11122	PTPRT	NM_007050 NM_133170

11128	POLR3A	NM_007055
11136	SLC7A9	NM_014270
11146	GLMN	NM_053274
11169	WDHD1	NM_001008396 NM_007086
11174	ADAMTS6	NM_197941
11227	GALNT5	NM_014568
11235	PDCD10	NM_007217 NM_145859 NM_145860
11275	KLHL2	NM_007246
11281	POU6F2	NM_007252
22796	COG2	NM_007357
22797	TFEC	NM_001018058 NM_012252
22829	NLGN4Y	NM_014893
22871	NLGN1	NM_014932
22882	ZHX2	NM_014943
22891	ZNF365	NM_014951 NM_199450 NM_199451 NM_199452
22901	ARSG	NM_014960
22920	KIFAP3	NM_014970
22955	SCMH1	NM_001031694 NM_012236
22986	SORCS3	NM_014978
22987	SV2C	NM_014979
22990	PCNX	NM_014982
23026	MYO16	NM_015011
23047	PDS5B	NM_015032
23057	NMNAT2	NM_015039 NM_170706
23071	TXNDC4	NM_015051
23077	MYCBP2	NM_015057
23081	JMJD2C	NM_015061
23090	ZNF423	NM_015069
23092	ARHGAP26	NM_015071
23105	FSTL4	NM_015082
23122	CLASP2	NM_015097
23136	EPB41L3	NM_012307
23161	SNX13	NM_015132
23215	BAT2D1	NM_015172
23229	ARHGEF9	NM_015185
23236	PLCB1	NM_015192 NM_182734
23261	CAMTA1	NM_015215
23273	KIAA0367	NM_015225
23275	POFUT2	NM_015227 NM_133634 NM_133635
23312	DMXL2	NM_015263
23345	SYNE1	NM_015293 NM_033071 NM_133650 NM_182961
23414	ZFPM2	NM_012082
23530	NNT	NM_012343 NM_182977
23553	HYAL4	NM_012269
23586	DDX58	NM_014314
23601	CLEC5A	NM_013252
23635	SSBP2	NM_012446
23639	LRRC6	NM_012472
23783	ADPRTL4	-
24137	KIF4A	NM_012310
24145	PANX1	NM_015368
25771	TBC1D22A	NM_014346

25827	FBXL2	NM_012157
25914	RTTN	NM_173630
25925	ZNF521	NM_015461
26009	ZZZ3	NM_015534
26033	ATRNL1	NM_207303
26034	PIP3-E	NM_015553
26040	SETBP1	NM_015559
26054	SENP6	NM_015571
26059	ERC2	NM_015576
26090	ABHD12	NM_015600
26137	ZBTB20	NM_015642
26166	RGS22	NM_015668
26228	STAP1	NM_012108
26235	FBXL4	NM_012160
26290	GALNT8	NM_017417
26575	RGS17	NM_012419
27067	STAU2	NM_014393
27068	PPA2	NM_001034191 NM_006903 NM_176866 NM_176867 NM_176869
27185	DISC1	NM_001012957 NM_001012958 NM_001012959 NM_018662
27194	SEDLP4	-
27241	BBS9	NM_001033604 NM_001033605 NM_014451 NM_198428
27242	TNFRSF21	NM_014452
27258	LSM3	NM_014463
27291	C10orf28	NM_014472
27332	ZNF638	NM_001014972 NM_014497
27333	GOLIM4	NM_014498
28957	MRPS28	NM_014018
28965	SLC27A6	NM_001017372 NM_014031
28998	MRPL13	NM_014078
29906	ST8SIA5	NM_013305
29967	LRP12	NM_013437
29969	MDFIC	NM_199072
29994	BAZ2B	NM_013450
30010	NXPH1	NM_152745
50507	NOX4	NM_016931
50814	NSDHL	NM_015922
50940	PDE11A	NM_016953
51057	LOC51057	NM_015910
51071	DERA	NM_015954
51086	TNNI3K	NM_015978
51164	DCTN4	NM_016221
51196	PLCE1	NM_016341
51281	ANKMY1	NM_016552 NM_017844
51302	CYP39A1	NM_016593
51306	C5orf5	NM_016603
51319	RSRC1	NM_016625
51334	PRR16	NM_016644
51366	UBR5	NM_015902
51390	AIG1	NM_016108
51430	C1orf9	NM_014283 NM_016227
51454	GULP1	NM_016315
51473	DCDC2	NM_016356

51501	C11orf73	NM_016401
51542	VPS54	NM_001005739 NM_016516
51594	NAG	NM_015909
51776	ZAK	NM_016653 NM_133646
53358	SHC3	NM_016848
53904	MYO3A	NM_017433
54431	DNAJC10	NM_018981
54462	KIAA1128	NM_018999
54504	CPVL	NM_019029 NM_031311
54514	DDX4	NM_024415
54520	CCDC93	NM_019044
54532	USP53	NM_019050
54768	HYDIN	NM_017558
54809	SAMD9	NM_017654
54823	C1orf26	NM_017673
54827	FAM55D	NM_017678
54830	NUP62CL	NM_017681
54832	VPS13C	NM_001018088 NM_017684 NM_018080 NM_020821
54839	LRRC49	NM_017691
54875	CNTLN	NM_017738
54885	TBC1D8B	NM_017752 NM_198881
54914	KIAA1797	NM_017794
54967	CXorf48	NM_001031705 NM_017863
54970	TTC12	NM_017868
55010	C12orf48	NM_017915
55013	CCDC109B	NM_017918
55023	PHIP	NM_017934
55034	MOCOS	NM_017947
55041	PLEKHB2	NM_001031706 NM_017958
55064	C9orf68	NM_001039395
55075	UACA	NM_001008224 NM_018003
55120	FANCL	NM_018062
55125	CEP192	NM_032142
55129	TMEM16K	NM_018075
55236	UBA6	NM_018227
55248	TMEM206	NM_018252
55255	WDR41	NM_018268
55334	SLC39A9	NM_018375
55553	SOX6	NM_017508 NM_033326
55576	STAB2	NM_017564
55617	TASP1	NM_017714
55686	MREG	NM_018000
55757	UGCGL2	NM_020121
55779	WDR52	NM_018338
55788	LMBRD1	NM_018368
55789	DEPDC1B	NM_018369
55840	EAF2	NM_018456
55869	HDAC8	NM_018486
55871	CBWD1	NM_018491
55914	ERBB2IP	NM_001006600 NM_018695
56163	RNF17	NM_031277
56164	STK31	NM_031414 NM_032944

56169	MLZE	NM_031415
56171	DNAH7	NM_018897
56341	PRMT8	NM_019854
56477	CCL28	NM_148672
56479	KCNQ5	NM_019842
56852	RAD18	NM_020165
56884	FSTL5	NM_020116
56981	PRDM11	NM_020229
56987	BBX	NM_020235
57054	DAZ3	NM_020364
57055	DAZ2	NM_001005785 NM_001005786 NM_020363
57062	DDX24	NM_020414
57097	PARP11	NM_020367
57102	C12orf4	NM_020374
57113	TRPC7	NM_020389
57135	DAZ4	NM_001005375 NM_020420
57187	THOC2	NM_020449
57282	SLC4A10	NM_022058
57337	SENP7	NM_020654
57406	ABHD6	NM_020676
57478	USP31	NM_020718
57484	RNF150	NM_020724
57512	GPR158	NM_020752
57526	PCDH19	NM_020766
57531	HACE1	NM_020771
57537	SORCS2	NM_020777
57544	TXNDC16	NM_020784
57554	LRRC7	NM_020794
57560	IFT80	NM_020800
57578	KIAA1409	NM_020818
57579	FAM135A	NM_020819
57589	KIAA1432	NM_020829
57620	STIM2	NM_020860
57623	ZFAT	NM_001029939 NM_020863
57626	KLHL1	NM_020866
57628	DPP10	NM_001004360 NM_020868
57639	CCDC146	NM_020879
57653	KIAA1529	NM_020893
57669	EPB41L5	NM_020909
57689	LRRC4C	NM_020929
57706	DENND1A	NM_020946 NM_024820
57821	C1orf114	NM_021179
58499	ZNF462	NM_021224
59277	NTN4	NM_021229
59350	RXFP1	NM_021634
60412	EXOC4	NM_001037126 NM_021807
60468	BACH2	NM_021813
60492	CCDC90B	NM_021825
63917	GALNT11	NM_022087
64084	CLSTN2	NM_022131
64087	MCCC2	NM_022132
64092	SAMSN1	NM_022136

64097	EPB41L4A	NM_022140
64116	SLC39A8	NM_022154
64168	NECAB1	NM_022351
64327	LMBR1	NM_022458
64393	ZMAT3	NM_022470 NM_152240
64754	SMYD3	NM_022743
64762	FAM59A	NM_022751
64799	IQCH	NM_001031715 NM_022784
64901	RANBP17	NM_022897
64902	AGXT2	NM_031900
64969	MRPS5	NM_031902
65217	PCDH15	NM_033056
65975	STK33	NM_030906
66037	BOLL	NM_033030 NM_197970
79071	ELOVL6	NM_024090
79172	CENPO	NM_024322
79175	ZNF343	NM_024325
79582	SPAG16	NM_001025436 NM_024532
79587	CARS2	NM_024537
79589	RNF128	NM_024539 NM_194463
79634	SCRN3	NM_024583
79657	RPAP3	NM_024604
79674	VEPH1	NM_024621
79698	ZMAT4	NM_024645
79710	MORC4	NM_024657
79722	ANKRD55	NM_001039935 NM_024669
79740	ZBBX	NM_024687
79768	C15orf29	NM_024713
79772	MCTP1	NM_001002796 NM_024717
79783	C7orf10	NM_024728
79799	UGT2A3	NM_024743
79807	GSTCD	NM_001031720 NM_024751
79815	NPAL2	NM_024759
79823	C2orf34	NM_024766
79937	CNTNAP3	NM_033655
79953	C20orf39	NM_024893
79974	C7orf58	NM_024913
80055	PGAP1	NM_024989
80070	ADAMTS20	NM_025003 NM_175851
80071	CCDC15	NM_025004
80146	UXS1	NM_025076
80157	FLJ21511	NM_025087
80224	NUBPL	NM_025152
80258	EFHC2	NM_025184
80321	CEP70	NM_024491
80821	DDHD1	NM_030637
80856	KIAA1715	NM_030650
81494	CFHR5	NM_030787
81533	ITFG1	NM_030790
81578	COL21A1	NM_030820
81608	FIP1L1	NM_030917
81792	ADAMTS12	NM_030955

81931	ZNF93	NM_031218
83468	GLT8D2	NM_031302
83478	ARHGAP24	NM_001025616 NM_031305
83698	CALN1	NM_001017440 NM_031468
83699	SH3BGRL2	NM_031469
83734	ATG10	NM_031482
83851	SYT16	NM_031914
83872	HMCN1	NM_031935
83938	C10orf11	NM_032024
83989	C5orf21	NM_032042
84034	EMILIN2	NM_032048
84056	KATNAL1	NM_001014380 NM_032116
84062	DTNBP1	NM_032122 NM_183040 NM_183041
84068	SLC10A7	NM_001029998 NM_001030316 NM_032128
84146	ZNF644	NM_016620 NM_032186 NM_201269
84187	TMEM164	NM_032227
84239	ATP13A4	NM_032279
84530	KIAA1853	NM_194286
84570	COL25A1	NM_032518 NM_198721
84620	ST6GAL2	NM_032528
84679	SLC9A7	NM_032591
84708	LNX1	NM_032622
84791	C1orf97	NM_032705
84871	AGBL4	NM_032785
84873	GPR128	NM_032787
84900	RNFT2	NM_032814
84946	LTV1	NM_032860
84953	MICALCL	NM_032867
84955	NUDCD1	NM_032869
84978	FRMD5	NM_032892
85413	SLC22A16	NM_033125
85417	CCNB3	NM_033031 NM_033670
89846	FGD3	NM_033086
90025	UBE2CBP	NM_198920
91050	CCDC149	NM_173463
91147	TMEM67	NM_153704
91431	LOC91431	NM_138698
91526	ANKRD44	NM_153697
91687	CENPL	NM_033319
92369	SPSB4	NM_080862
92454	PRR8	NM_053043
93035	PKHD1L1	NM_177531
93492	TPTE2	NM_130785 NM_199254
93627	MGC16169	NM_033115
93664	CADPS2	NM_001009571 NM_017954
112609	C6orf117	NM_138409
114327	EFHC1	NM_018100
114784	CSMD2	NM_052896
114792	KLHL32	NM_052904
114805	GALNT13	NM_052917
114836	SLAMF6	NM_052931
114908	TMEM123	NM_052932

115111	SLC26A7	NM_052832 NM_134266
115286	SLC25A26	NM_173471
115350	FCRL1	NM_052938
115825	WDFY2	NM_052950
115827	RAB3C	NM_138453
116496	FAM129A	NM_052966
117177	RAB3IP	NM_001024647 NM_022456 NM_175623 NM_175624 NM_175625
117245	HRASLS5	NM_054108
117583	PARD3B	NM_057177 NM_152526 NM_205863
118429	ANTXR2	NM_058172
118491	TTC18	NM_145170
118611	C10orf90	NM_001004298
120400	FAM55A	NM_152315
120406	FAM55B	NM_182495
121601	TMEM16D	NM_178826
122046	C13orf26	NM_152325
123355	LRRC28	NM_144598
123591	C15orf27	NM_152335
124149	FLJ43980	NM_001004299
125228	C18orf19	NM_152352
126204	NLRP13	NM_176810
126859	C1orf125	NM_144696 NM_182766
128153	SPATA17	NM_138796
129563	DIS3L2	NM_152383
129642	MBOAT2	NM_138799
130271	PLEKHH2	NM_172069
130540	ALS2CR12	NM_139163
130574	LYPD6	NM_194317
130940	CCDC148	NM_138803
131034	CPNE4	NM_130808
131544	DKFZp667G2110	NM_153605
131566	DCBLD2	NM_080927
132612	ADAD1	NM_139243
132671	SPATA18	NM_145263
132884	EVC2	NM_147127
132949	AASDH	NM_181806
133015	C4orf28	NM_145048
133121	ENPP6	NM_153343
133558	FLJ40243	NM_173489
133690	CAPSL	NM_144647
134359	C5orf37	NM_152408
135152	B3GAT2	NM_080742
137492	VPS37A	NM_152415
137868	SGCZ	NM_139167
138412	LOC138412	-
138639	PTPDC1	NM_152422 NM_177995
139221	MUM1L1	NM_152423
139322	APOOL	NM_198450
139411	PTCHD1	NM_173495
140469	MYO3B	NM_138995
140609	NEK7	NM_133494
143425	SYT9	NM_175733

143884	CWF19L2	NM_152434
144402	CPNE8	NM_153634
144577	FLJ32549	NM_152440
145389	SLC38A6	NM_153811
145407	C14orf37	NM_001001872
145508	C14orf145	NM_152446
145581	LRFN5	NM_152447
145773	FAM81A	NM_152450
146057	TTBK2	NM_173500
148418	SAMD13	NM_001010971
148534	TMEM56	NM_152487
148641	SLC35F3	NM_173508
148823	C1orf150	NM_145278
148867	SLC30A7	NM_133496
149233	IL23R	NM_144701
149297	FAM78B	NM_001017961
150159	NHEDC1	NM_139173
150465	TTL	NM_153712
150472	CBWD2	NM_172003
151246	SGOL2	NM_152524
151393	FAM82A	NM_144713
152028	FNDC6	NM_144717
152185	CCDC52	NM_144718
152330	CNTN4	NM_175607 NM_175612 NM_175613
153218	SPINK5L3	NM_001040129
154661	RUNDC3B	NM_138290
154664	ABCA13	NM_152701
154743	FLJ31818	NM_152556
157807	RLBP1L1	NM_173519
160140	C11orf65	NM_152587
160335	TMTC2	NM_152588
160492	IFLTD1	NM_152590
160728	SLC5A8	NM_145913
160777	CCDC60	NM_178499
161357	MDGA2	NM_182830
163081	ZNF567	NM_152603
163486	DENND1B	NM_144977
163589	TDRD5	NM_173533
166336	PRICKLE2	NM_198859
167359	MGC42105	NM_153361
168090	C6orf118	NM_144980
168667	BMPER	NM_133468
168975	CNBD1	NM_173538
169044	COL22A1	NM_152888
171019	ADAMTS19	NM_133638
196074	METT5D1	NM_152636
196296	DCDC5	NM_198462
196792	FAM24B	NM_152644
200420	ALMS1P	NM_145300
202333	CMYA5	NM_153610
203102	ADAM32	NM_145004
203427	SLC25A43	NM_145305

203447	NRK	NM_198465
204219	LASS3	NM_178842
204801	NLRP11	NM_145007
206938	C9orf94	NM_001040272
219578	ZNF804B	NM_181646
220115	LOC220115	NR_002793
220416	RP11-139H14.4	NM_001024609
220869	CBWD5	NM_001024916
221061	C10orf38	NM_001010924
221074	SLC39A12	NM_152725
221078	NSUN6	NM_182543
221264	C6orf199	NM_145025
221294	NT5DC1	NM_152729
221302	ZUFSP	NM_145062
221458	KIF6	NM_145027
221895	JAZF1	NM_175061
246126	CYorf15A	NM_001005852
253260	RICTOR	NM_152756
253430	IPMK	NM_152230
253582	C6orf191	NM_001010876
253769	WDR27	NM_182552
253827	MSRB3	NM_001031679 NM_198080
254065	BRWD3	NM_153252
254827	NAALADL2	NM_207015
256435	ST6GALNAC3	NM_152996
256691	MAMDC2	NM_153267
256764	WDR72	NM_182758
257019	FRMD3	NM_174938
257044	C1orf101	NM_173807
257068	PLCXD2	NM_153268
257194	NEGR1	NM_173808
282809	WDR51B	NM_172240
283209	PGM2L1	NM_173582
283316	CD163L1	NM_174941
283417	DPY19L2	NM_173812
283777	FLJ39743	NM_182562
285154	C2orf58	NM_173652
285195	SLC9A9	NM_173653
285331	CCDC66	NM_001012506
285386	TPRG1	NM_198485
285596	FAM153A	NM_173663
285600	C5orf36	NM_173665
285754	FLJ37396	NM_001039527
286053	NSMCE2	NM_173685
286205	C9orf126	NM_173690
286239	LOC286239	-
286451	YIPF6	NM_173834
286464	CXorf59	NM_173695
317761	C14orf39	NM_174978
327658	HDHD1BP	-
338645	LUZP2	NM_001009909
339500	ZNF678	NM_178549

339883	C3orf35	NM_178339 NM_178342
340419	RSPO2	NM_178565
340441	A26A1	NM_001002920 NM_001005365
340481	ZDHHC21	NM_178566
341640	FREM2	NM_207361
343450	KCNT2	NM_198503
344387	CDKL4	NM_001009565
344758	GPR149	NM_001038705
345757	TMEM157	NM_198507
347404	LANCL3	NM_198511
347613	PARP4P	-
347732	CATSPER3	NM_178019
348808	LOC348808	NR_002811
348825	TPRXL	NR_002223
349152	DPY19L2P2	NM_182634
353299	RGSL1	NM_181572
360021	PPP1R12BP	-
374467	C12orf63	NM_198520
374654	KIF7	NM_198525
374864	C18orf34	NM_198995
374868	ATP9B	NM_198531
374992	SEC63D1	NM_198550
375519	GJB7	NM_198568
378955	RBMY2JP	-
386617	KCTD8	NM_198353
386695	OFDYP11	-
387694	SH2D4B	NM_207372
387700	SLC16A12	NM_213606
388646	GBP7	NM_207398
388649	C1orf146	NM_001012425
388650	FAM69A	NM_001006605
389668	XKR9	NM_001011720
389840	MAP3K15	NM_001001671
401145	MGC48628	NM_207491
401191	FLJ46010	NM_001001703
401252	LOC401252	NM_001013681
401541	CENPP	NM_001012267
401612	MCART6	NM_001012755
404672	GTF2H5	NM_207118
404744	AAA1	NM_207283 NM_207284 NM_207285 NM_207286 NM_207287 NM_207288 NM_207289 [illegible]
414753	LOC414753	-
440867	FLJ16124	NM_001004345
441024	MTHFD2L	NM_001004346
442862	PRY2	NM_001002758
445571	CBWD3	NM_201453
554236	DPY19L2P1	NR_002833
619279	ZNF704	NM_001033723
642406	LOC642406	-
643707	LOC643707	-
643789	LOC643789	-
644335	LOC644335	-
644780	LOC644780	-

648283	LOC648283	-
649024	LOC649024	-
653510	LOC653510	-
654463	FER1L6	NM_001039112

Table 1.4 Genes with both sense and antisense L1s (286 genes).

NCBI gene ID	NCBI gene name	mRNA accession
105	ADARB2	NM_018702
132	ADK	NM_001123 NM_006721
320	APBA1	NM_001163
627	BDNF	NM_001709 NM_170731 NM_170732 NM_170733 NM_170734 NM_170735
1193	CLIC2	NM_001289
1288	COL4A6	NM_001847 NM_033641
1301	COL11A1	NM_001854 NM_080629 NM_080630
1310	COL19A1	NM_001858
1574	CYP3A	-
1600	DAB1	NM_021080
1607	DGKB	NM_004080 NM_145695
1630	DCC	NM_005215
1633	DCK	NM_000788
1730	DIAPH2	NM_006729 NM_007309
1740	DLG2	NM_001364
1756	DMD	NM_000109 NM_004006 NM_004007 NM_004009 NM_004010 NM_004011 NM_004012 [illegible]
1795	DOCK3	NM_004947
1826	DSCAM	NM_001389 NM_206887
1896	EDA	NM_001005609 NM_001005610 NM_001005611 NM_001005612 NM_001005613 [illegible]
2162	F13A1	NM_000129
2201	FBN2	NM_001999
2218	FKTN	NM_006731
2262	GPC5	NM_004466
2272	FHIT	NM_002012
2556	GABRA3	NM_000808
2560	GABRB1	NM_000812
2690	GHR	NM_000163
2762	GMDS	NM_001500
2863	GPR39	NM_001508
2893	GRIA4	NM_000829
2895	GRID2	NM_001510
2915	GRM5	NM_000842
2917	GRM7	NM_000844 NM_181874 NM_181875
2918	GRM8	NM_000845
3358	HTR2C	NM_000868
3535	IGL@	-
3756	KCNH1	NM_002238 NM_172362
3908	LAMA2	NM_000426
3973	LHCGR	NM_000233
4036	LRP2	NM_004525
4045	LSAMP	NM_002338
4139	MARK1	NM_018650
4199	ME1	NM_002395
4438	MSH4	NM_002440
4507	MTAP	NM_002451
4684	NCAM1	NM_000615 NM_181351
4745	NELL1	NM_006157
4750	NEK1	NM_012224

4753	NELL2	NM_006159
4978	OPCML	NM_001012393 NM_002545
5067	CNTN3	NM_020872
5140	PDE3B	NM_000922
5218	PFTK1	NM_012395
5251	PHEX	NM_000444
5288	PIK3C2G	NM_004570
5314	PKHD1	NM_138694 NM_170724
5558	PRIM2	NM_000947
5592	PRKG1	NM_006258
5602	MAPK10	NM_002753 NM_138980 NM_138981 NM_138982
5797	PTPRM	NM_002845
5890	RAD51L1	NM_002877 NM_133509 NM_133510
5927	JARID1A	NM_005056
6098	ROS1	NM_002944
6262	RYR2	NM_001035
6451	SH3BGRL	NM_003022
6641	SNTB1	NM_021021
6788	STK3	NM_006281
6845	VAMP7	NM_005638
6847	SYCP1	NM_003176
6870	TACR3	NM_001059
6935	ZEB1	NM_030751
7224	TRPC5	NM_012471
7225	TRPC6	NM_004621
7325	UBE2E2	NM_152653
7518	XRCC4	NM_003401 NM_022406 NM_022550
7813	EVI5	NM_005665
7840	ALMS1	NM_015120
8499	PPFIA2	NM_003625
8633	UNC5C	NM_003728
9228	DLGAP2	NM_004745
9312	KCNB2	NM_004770
9348	NDST3	NM_004784
9358	ITGBL1	NM_004791
9369	NRXN3	NM_004796 NM_138970
9472	AKAP6	NM_004274
9630	GNA14	NM_004297
9699	RIMS2	NM_014677
9758	FRMPD4	NM_014728
9779	TBC1D5	NM_014744
9844	ELMO1	NM_001039459 NM_014800 NM_130442
9863	MAGI2	NM_012301
10082	GPC6	NM_005708
10178	ODZ1	NM_014253
10225	CD96	NM_005816 NM_198196
10243	GPHN	NM_001024218 NM_020806
10274	STAG1	NM_005862
10371	SEMA3A	NM_006080
10404	PGCP	NM_016134
10451	VAV3	NM_006113
10886	NPFFR2	NM_004885 NM_053036

11064	CEP110	NM_007018
11214	AKAP13	NM_006738 NM_007200 NM_144767
22862	FNDC3A	NM_014923
22866	CNKSR2	NM_014927
22999	RIMS1	NM_014989
23007	PLCH1	NM_014996
23179	RGL1	NM_015149
23194	FBXL7	NM_012304
23245	ASTN2	NM_014010 NM_198186 NM_198187 NM_198188
23253	ANKRD12	NM_015208
23256	SCFD1	NM_016106 NM_182835
23281	KIAA0774	NM_001033602 NM_015233
23284	LPHN3	NM_015236
23301	EHBP1	NM_015252
25834	MGAT4C	NM_013244
25924	MYRIP	NM_015460
26002	MOXD1	NM_001031699
26047	CNTNAP2	NM_014141
26052	DNM3	NM_015569
26280	IL1RAPL2	NM_017416
26960	NBEA	NM_015678
27123	DKK2	NM_014421
27130	INVS	NM_014425 NM_183245
27152	INTU	NM_015693
27303	RBMS3	NM_001003792 NM_001003793 NM_014483
27328	PCDH11X	NM_014522 NM_032967 NM_032968 NM_032969
29119	CTNNA3	NM_013266
29953	TRHDE	NM_013381
29970	SCHIP1	NM_014575
49855	SCAPER	NM_020843
50859	SPOCK3	NM_001040159 NM_016950
51134	CCDC41	NM_016122
51167	CYB5R4	NM_016230
51397	COMMD10	NM_016144
51761	ATP8A2	NM_016529
53344	CHIC1	NM_001039840
53353	LRP1B	NM_018557
53616	ADAM22	NM_004194 NM_016351 NM_021721 NM_021722 NM_021723
53942	CNTN5	NM_014361 NM_175566
54212	SNTG1	NM_018967
54221	SNTG2	NM_018968
54558	SPATA6	NM_019073
54765	TRIM44	NM_017583
54808	DYM	NM_017653
54828	BCAS3	NM_017679
54886	RP11-35N6.1	NM_017753 NM_207299
55061	SUSD4	NM_001037175 NM_017982
55068	ENOX1	NM_017993
55100	WDR70	NM_018034
55130	ARMC4	NM_018076
55217	TMLHE	NM_018196
55277	FGGY	NM_018291

55297	CCDC91	NM_018318
55328	C10orf59	NM_001031709 NM_018363
55331	PHCA	NM_018367
55351	STK32B	NM_018401
55607	PPP1R9A	NM_017650
55613	MTMR8	NM_017677
55703	POLR3B	NM_018082
55799	CACNA2D3	NM_018398
55843	ARHGAP15	NM_018460
55906	KIAA1166	NM_018684
56001	NXF2	NM_017809 NM_022053
56159	TEX11	NM_001003811 NM_031276
56899	ANKS1B	NM_020140 NM_152788 NM_181670
56934	CA10	NM_020178
56990	CDC42SE2	NM_001038702 NM_020240
57047	PLSCR2	NM_020359
57161	PELI2	NM_021255
57536	KIAA1328	NM_020776
60495	HPSE2	NM_021828
60682	SMAP1	NM_021940
63982	TMEM16C	NM_031418
64326	RFWD2	NM_001001740 NM_022457
64478	CSMD1	NM_033225
64839	FBXL17	NM_022824
64864	RFXDC2	NM_022841
65084	TMEM135	NM_022918
66000	TMEM108	NM_023943
79741	C10orf68	NM_024688
79858	NEK11	NM_024800 NM_145910
79895	ATP8B4	NM_024837
79908	BTNL8	NM_024850
80144	FRAS1	NM_025074
80309	SPHKAP	NM_030623
80333	KCNIP4	NM_001035003 NM_001035004 NM_025221 NM_147181 NM_147182 NM_147183
80705	TSGA10	NM_025244 NM_182911
81567	TXNDC5	NM_022085 NM_030810
81846	SBF2	NM_030962
81849	ST6GALNAC5	NM_030965
83259	PCDH11Y	NM_032971 NM_032972 NM_032973
83696	NIBP	NM_031466
83894	TTC29	NM_031956
83943	IMMP2L	NM_032549
84059	GPR98	NM_032119
84083	ZRANB3	NM_032143
84216	TMEM117	NM_032256
91133	L3MBTL4	NM_173464
91752	ZNF804A	NM_194250
92675	DTD1	NM_080820
92949	ADAMTSL1	NM_052866 NM_139238 NM_139264
114134	SLC2A13	NM_052885
114299	PALM2	NM_001037293 NM_053016
114788	CSMD3	NM_052900 NM_198123 NM_198124

116328	C8orf34	NM_052958
117154	DACH2	NM_053281
117531	TMC1	NM_138691
121256	TMEM132D	NM_133448
128553	TSHZ2	NM_173485
128954	GAB4	NM_001037814
129684	CNTNAP5	NM_130773 NM_138996
130399	ACVR1C	NM_145259
131096	KCNH8	NM_144633
132320	SCLT1	NM_144643
133482	SLCO6A1	NM_173488
135138	PACRG	NM_152410
138046	RALYL	NM_173848
139324	HDX	NM_144657
140733	MACROD2	NM_001033086 NM_001033087 NM_080676
143279	HECTD2	NM_173497 NM_182765
145173	B3GALTL	NM_194318
145282	MIPOL1	NM_138731
150596	FLJ32955	NM_153041
151126	ZNF385B	NM_152520
151531	UPP2	NM_173355
151647	FAM19A4	NM_001005527 NM_182522
151790	WDR49	NM_178824
152002	C3orf21	NM_152531
152404	IGSF11	NM_001015887 NM_152538
152579	SCFD2	NM_152540
152940	FLJ25371	NM_152543
154215	NKAIN2	NM_001040214
157376	C8orf78	NM_182525
157680	VPS13B	NM_015243 NM_017890 NM_152564 NM_181661
158038	LINGO2	NM_152570
158584	FAAH2	NM_174912
161725	OTUD7A	NM_130901
166378	SPATA5	NM_145207
170691	ADAMTS17	NM_139057
196951	C15orf33	NM_152647
200150	PLD5	NM_152666
200844	C3orf67	NM_198463
202559	KHDRBS2	NM_152688
203238	C9orf93	NM_173550
204962	SLC44A5	NM_152697
221016	CCDC7	NM_001026383 NM_145023
221322	C6orf170	NM_152730
222235	FBXL13	NM_145032
223075	CCDC129	NM_194300
246119	TTTY10	NR_001542
255119	C4orf22	NM_152770
255631	COL24A1	NM_152890
255928	SYT14	NM_153262
259239	WFDC11	NM_147197
260425	MAGI3	NM_020965 NM_152900
266722	HS6ST3	NM_153456

283461	C12orf40	NM_001031748
284521	OR2L13	NM_175911
285335	SLC9A10	NM_183061
285555	C4orf37	NM_174952
286183	NKAIN3	NM_173688
338811	FAM19A2	NM_178539
338821	LST-3TM12	NM_001009562
340267	COL28A1	NM_001037763
340533	KIAA2022	NM_001008537
340595	ZCCHC16	NM_001004308
345557	PLCXD3	NM_001005473
348980	HCN1	NM_021072
349565	NMNAT3	NM_178177
374470	C12orf42	NM_198521
375484	C5orf25	NM_198567
375612	LHFPL3	NM_199000
387601	SLC22A25	NM_199352
388815	C21orf34	NM_001005732 NM_001005733 NM_001005734
389400	GFRAL	NM_207410
392232	LOC392232	-
392636	TMEM195	NM_001004320
401013	FLJ34870	NM_207481
401337	FLJ45974	NM_001001707
401398	LOC401398	NM_001023566
401474	SAMD12	NM_207506
407738	FAM19A1	NM_213609
441732	LOC441732	-
442117	GALNT17	NM_001034845
445815	PALM2-AKAP2	NM_007203 NM_147150
644094	LOC644094	-
