# Supplementary material for: Hypomethylation of Intragenic LINE-1 Represses Transcription in Cancer Cells through AGO2
Source: PLoS One. 2011 Mar 15;6(3):e17934. doi: 10.1371/journal.pone.0017934 (PMC3057998; doi:10.1371/journal.pone.0017934)
Supplement: Table S2 — Analysis of L1 characteristics. (PDF) [file pone.0017934.s004.pdf]

Table 2.1 The 2x2 contingency tables for chi-square analysis of categorical L1 characteristics. The number of observed and expected L1 sequences containing tested L1 characteristic were shown. For the characteristics following with "*", the values refer to the number of genes harboring L1 sequence with tested characteristic (for intragenic, sense, and antisense L1s) and the number of L1 sequences containing tested characteristic (for intergenic L1s).

Experiment : intragenics L1s vs intergenic L1s

Total number of intragenic L1s = 2546
Total number of intergenic L1s = 9355
p is one-tailed probability

chromosome location				
	intragenic L1s		intergenic L1s	
Case	Observed	Expected	Observed	Expected
chromosome1 *	126	94.305	578	609.695
Other	1321	1352.695	8777	8745.305
Odd Ratio = 1.45	Sum chi = 13.15	p = 0.000286432329		
chromosome1	203	167.081	578	613.919
Other	2343	2378.919	8777	8741.081
Odd Ratio = 1.32	Sum chi = 10.51	p = 0.001184999997		
chromosome2 *	122	114.265	731	738.735
Other	1325	1332.735	8624	8616.265
Odd Ratio = 1.09	Sum chi = 0.656	p = 0.417823626144		
chromosome2	201	199.384	731	732.616
Other	2345	2346.616	8624	8622.384
Odd Ratio = 1.01	Sum chi = 0.018	p = 0.893060196422		
chromosome3 *	115	108.505	695	701.495
Other	1332	1338.495	8660	8653.505
Odd Ratio = 1.08	Sum chi = 0.485	p = 0.486019706325		
chromosome3	230	197.887	695	727.113
Other	2316	2348.113	8660	8627.887
Odd Ratio = 1.24	Sum chi = 7.188	p = 0.007337699773		
chromosome4 *	107	124.178	820	802.822
Other	1340	1322.822	8535	8552.178
Odd Ratio = 0.83	Sum chi = 3.001	p = 0.083193857246		
chromosome4	224	223.345	820	820.655
Other	2322	2322.655	8535	8534.345
Odd Ratio = 1.00	Sum chi = 0.002	p = 0.958697529250		
chromosome5 *	96	113.193	749	731.807
Other	1351	1333.807	8606	8623.193
Odd Ratio = 0.82	Sum chi = 3.271	p = 0.070496024952		
chromosome5	171	196.817	749	723.183
Other	2375	2349.183	8606	8631.817
Odd Ratio = 0.83	Sum chi = 4.669	p = 0.030710148389		
chromosome6 *	101	95.779	614	619.221
Other	1346	1351.221	8741	8735.779
Odd Ratio = 1.07	Sum chi = 0.351	p = 0.553031999456		
chromosome6	164	166.439	614	611.561
Other	2382	2379.561	8741	8743.439
Odd Ratio = 0.98	Sum chi = 0.048	p = 0.825446764061		
chromosome7 *	87	68.318	423	441.682
Other	1360	1378.682	8932	8913.318
Odd Ratio = 1.35	Sum chi = 6.191	p = 0.012838026681		
chromosome7	152	123.011	423	451.989
Other	2394	2422.989	8932	8903.011

Experiment : sense L1s vs intergenic L1s

Total number of sense L1s = 829
Total number of intergenic L1s = 9355
p is one-tailed probability

chromosome location				
	sense L1s		intergenic L1s	
Case	Observed	Expected	Observed	Expected
chromosome1 *	50	38.915	578	589.085
Other	568	579.085	8777	8765.915
Odd Ratio = 1.34	Sum chi = 3.592	p = 0.058057076083		
chromosome1	62	52.097	578	587.903
Other	767	776.903	8777	8767.097
Odd Ratio = 1.23	Sum chi = 2.186	p = 0.139227751046		
chromosome2 *	51	48.458	731	733.542
Other	567	569.542	8624	8621.458
Odd Ratio = 1.06	Sum chi = 0.154	p = 0.694555978395		
chromosome2	63	64.633	731	729.367
Other	766	764.367	8624	8625.633
Odd Ratio = 0.97	Sum chi = 0.048	p = 0.825281963037		
chromosome3 *	49	46.104	695	697.896
Other	569	571.896	8660	8657.104
Odd Ratio = 1.07	Sum chi = 0.209	p = 0.647073739917		
chromosome3	70	62.273	695	702.727
Other	759	766.727	8660	8652.273
Odd Ratio = 1.15	Sum chi = 1.128	p = 0.288069679351		
chromosome4 *	45	53.602	820	811.398
Other	573	564.398	8535	8543.602
Odd Ratio = 0.82	Sum chi = 1.611	p = 0.204308780004		
chromosome4	71	72.529	820	818.471
Other	758	756.471	8535	8536.529
Odd Ratio = 0.97	Sum chi = 0.038	p = 0.844498786757		
chromosome5 *	36	48.644	749	736.356
Other	582	569.356	8606	8618.644
Odd Ratio = 0.71	Sum chi = 3.803	p = 0.051155347992		
chromosome5	48	64.878	749	732.122
Other	781	764.122	8606	8622.878
Odd Ratio = 0.71	Sum chi = 5.185	p = 0.022776112675		
chromosome6 *	38	40.403	614	611.597
Other	580	577.597	8741	8743.403
Odd Ratio = 0.93	Sum chi = 0.162	p = 0.686429211288		
chromosome6	46	53.725	614	606.275
Other	783	775.275	8741	8748.725
Odd Ratio = 0.84	Sum chi = 1.293	p = 0.255472423162		
chromosome7 *	36	28.443	423	430.557
Other	582	589.557	8932	8924.443
Odd Ratio = 1.31	Sum chi = 2.243	p = 0.134157884651		
chromosome7	48	38.34	423	432.66
Other	781	790.66	8932	8922.34

Experiment : antisense L1s vs intergenic L1s

Total number of antisense L1s = 1729
Total number of intergenic L1s = 9355
p is one-tailed probability

chromosome location				
	antisense L1s		intergenic L1s	
Case	Observed	Expected	Observed	Expected
chromosome1 *	99	72.212	578	604.788
Other	1018	1044.788	8777	8750.212
Odd Ratio = 1.48	Sum chi = 11.8922	p = 0.000563679949		
chromosome1	142	112.313	578	607.687
Other	1587	1616.687	8777	8747.313
Odd Ratio = 1.36	Sum chi = 9.94297	p = 0.001614643701		
chromosome2 *	89	87.466	731	732.534
Other	1028	1029.534	8624	8622.466
Odd Ratio = 1.02	Sum chi = 0.03269	p = 0.856519939615		
chromosome2	139	135.712	731	734.288
Other	1590	1593.288	8624	8620.712
Odd Ratio = 1.03	Sum chi = 0.10243	p = 0.748928555048		
chromosome3 *	93	84.052	695	703.948
Other	1024	1032.948	8660	8651.052
Odd Ratio = 1.13	Sum chi = 1.15300	p = 0.282921167770		
chromosome3	161	133.528	695	722.472
Other	1568	1595.472	8660	8632.528
Odd Ratio = 1.28	Sum chi = 7.25717	p = 0.007061814289		
chromosome4 *	86	96.639	820	809.361
Other	1031	1020.361	8535	8545.639
Odd Ratio = 0.87	Sum chi = 1.43523	p = 0.230912288369		
chromosome4	153	151.779	820	821.221
Other	1576	1577.221	8535	8533.779
Odd Ratio = 1.01	Sum chi = 0.01276	p = 0.910060418448		
chromosome5 *	74	87.786	749	735.214
Other	1043	1029.214	8606	8619.786
Odd Ratio = 0.82	Sum chi = 2.63003	p = 0.104859083492		
chromosome5	125	136.336	749	737.664
Other	1604	1592.664	8606	8617.336
Odd Ratio = 0.90	Sum chi = 1.21232	p = 0.270872795008		
chromosome6 *	82	74.239	614	621.761
Other	1035	1042.761	8741	8733.239
Odd Ratio = 1.13	Sum chi = 0.97284	p = 0.323971400290		
chromosome6	119	114.341	614	618.659
Other	1610	1614.659	8741	8736.341
Odd Ratio = 1.05	Sum chi = 0.24083	p = 0.623600688348		
chromosome7 *	70	52.586	423	440.414
Other	1047	1064.414	8932	8914.586
Odd Ratio = 1.41	Sum chi = 6.77412	p = 0.009248889625		
chromosome7	106	82.519	423	446.481
Other	1623	1646.481	8932	8908.519

Experiment : sense L1s vs antisense L1s

Total number of sense L1s = 829
Total number of antisense L1s = 1729
p is one-tailed probability

chromosome location				
	sense L1s		antisense L1s	
Case	Observed	Expected	Observed	Expected
chromosome1 *	50	53.073	99	95.927
Other	568	564.927	1018	1021.073
Odd Ratio = 0.91	Sum chi = 0.30	p = 0.582396118829		
chromosome1	62	66.113	142	137.887
Other	767	762.887	1587	1591.113
Odd Ratio = 0.90	Sum chi = 0.41	p = 0.521316571259		
chromosome2 *	51	49.867	89	90.133
Other	567	568.133	1028	1026.867
Odd Ratio = 1.04	Sum chi = 0.04	p = 0.834860677040		
chromosome2	63	65.464	139	136.536
Other	766	763.536	1590	1592.464
Odd Ratio = 0.94	Sum chi = 0.14	p = 0.699469626728		
chromosome3 *	49	50.58	93	91.42
Other	569	567.42	1024	1025.58
Odd Ratio = 0.95	Sum chi = 0.08	p = 0.772638380440		
chromosome3	70	74.863	161	156.137
Other	759	754.137	1568	1572.863
Odd Ratio = 0.90	Sum chi = 0.51	p = 0.473539477529		
chromosome4 *	45	46.662	86	84.338
Other	573	571.338	1031	1032.662
Odd Ratio = 0.94	Sum chi = 0.09	p = 0.752527404493		
chromosome4	71	72.594	153	151.406
Other	758	756.406	1576	1577.594
Odd Ratio = 0.96	Sum chi = 0.05	p = 0.811680652991		
chromosome5 *	36	39.182	74	70.818
Other	582	578.818	1043	1046.182
Odd Ratio = 0.87	Sum chi = 0.42	p = 0.512755447283		
chromosome5	48	56.066	125	116.934
Other	781	772.934	1604	1612.066
Odd Ratio = 0.79	Sum chi = 1.84	p = 0.174790349248		
chromosome6 *	38	42.744	82	77.256
Other	580	575.256	1035	1039.744
Odd Ratio = 0.83	Sum chi = 0.87	p = 0.348633954167		
chromosome6	46	53.473	119	111.527
Other	783	775.527	1610	1617.473
Odd Ratio = 0.79	Sum chi = 1.65	p = 0.198710814557		
chromosome7 *	36	37.757	70	68.243
Other	582	580.243	1047	1048.757
Odd Ratio = 0.93	Sum chi = 0.13	p = 0.713073721619		
chromosome7	48	49.909	106	104.091
Other	781	779.091	1623	1624.909

Odd Ratio = 1.34	Sum chi = 9.132	p = 0.002511359240		
chromosome8 *	71	79.57	523	514.43
Other	1376	1367.43	8832	8840.57
Odd Ratio = 0.87	Sum chi = 1.127	p = 0.288228727843		
chromosome8	137	141.195	523	518.805
Other	2409	2404.805	8832	8836.195
Odd Ratio = 0.96	Sum chi = 0.167	p = 0.682025405928		
chromosome9 *	64	54.118	340	349.882
Other	1383	1392.882	9015	9005.118
Odd Ratio = 1.23	Sum chi = 2.164	p = 0.141250025833		
chromosome9	103	94.772	340	348.228
Other	2443	2451.228	9015	9006.772
Odd Ratio = 1.12	Sum chi = 0.943	p = 0.331260233187		
chromosome10 *	61	51.305	322	331.695
Other	1386	1395.695	9033	9023.305
Odd Ratio = 1.23	Sum chi = 2.192	p = 0.138640887126		
chromosome10	132	97.125	322	356.875
Other	2414	2448.875	9033	8998.125
Odd Ratio = 1.53	Sum chi = 16.56	p = 0.000047068736		
chromosome11 *	74	76.221	495	492.779
Other	1373	1370.779	8860	8862.221
Odd Ratio = 0.96	Sum chi = 0.078	p = 0.778783469187		
chromosome11	130	133.707	495	491.293
Other	2416	2412.293	8860	8863.707
Odd Ratio = 0.96	Sum chi = 0.138	p = 0.710265298937		
chromosome12 *	75	65.907	417	426.093
Other	1372	1381.093	8938	8928.907
Odd Ratio = 1.17	Sum chi = 1.517	p = 0.217949304702		
chromosome12	120	114.881	417	422.119
Other	2426	2431.119	8938	8932.881
Odd Ratio = 1.06	Sum chi = 0.303	p = 0.581475436324		
chromosome13 *	46	38.847	244	251.153
Other	1401	1408.153	9111	9103.847
Odd Ratio = 1.23	Sum chi = 1.562	p = 0.211288841086		
chromosome13	76	68.458	244	251.542
Other	2470	2477.542	9111	9103.458
Odd Ratio = 1.15	Sum chi = 1.086	p = 0.297313364246		
chromosome14 *	37	43.268	286	279.732
Other	1410	1403.732	9069	9075.268
Odd Ratio = 0.83	Sum chi = 1.080	p = 0.298523502512		
chromosome14	68	75.732	286	278.268
Other	2478	2470.268	9069	9076.732
Odd Ratio = 0.87	Sum chi = 1.034	p = 0.308989878347		
chromosome15 *	52	31.882	186	206.118
Other	1395	1415.118	9169	9148.882
Odd Ratio = 1.84	Sum chi = 14.98	p = 0.000108128728		
chromosome15	76	56.05	186	205.95
Other	2470	2489.95	9169	9149.05
Odd Ratio = 1.52	Sum chi = 9.236	p = 0.002372210455		

Odd Ratio = 1.30	Sum chi = 2.7777	p = 0.095580664609		
chromosome8 *	34	34.516	523	522.484
Other	584	583.484	8832	8832.516
Odd Ratio = 0.98	Sum chi = 0.0087	p = 0.925672865648		
chromosome8	47	46.399	523	523.601
Other	782	782.601	8832	8831.399
Odd Ratio = 1.01	Sum chi = 0.0089	p = 0.924547914580		
chromosome9 *	26	22.68	340	343.32
Other	592	595.32	9015	9011.68
Odd Ratio = 1.16	Sum chi = 0.5378	p = 0.463334461224		
chromosome9	37	30.689	340	346.311
Other	792	798.311	9015	9008.689
Odd Ratio = 1.24	Sum chi = 1.4673	p = 0.225768011526		
chromosome10 *	24	21.441	322	324.559
Other	594	596.559	9033	9030.441
Odd Ratio = 1.13	Sum chi = 0.3373	p = 0.561343371579		
chromosome10	36	29.142	322	328.858
Other	793	799.858	9033	9026.142
Odd Ratio = 1.27	Sum chi = 1.8209	p = 0.177202434074		
chromosome11 *	37	32.967	495	499.033
Other	581	585.033	8860	8855.967
Odd Ratio = 1.14	Sum chi = 0.5557	p = 0.455989878045		
chromosome11	53	44.608	495	503.392
Other	776	784.392	8860	8851.608
Odd Ratio = 1.22	Sum chi = 1.8162	p = 0.177763859500		
chromosome12 *	35	28.009	417	423.991
Other	583	589.991	8938	8931.009
Odd Ratio = 1.29	Sum chi = 1.9483	p = 0.162760953302		
chromosome12	42	37.364	417	421.636
Other	787	791.636	8938	8933.364
Odd Ratio = 1.14	Sum chi = 0.6558	p = 0.418023508446		
chromosome13 *	22	16.483	244	249.517
Other	596	601.517	9111	9105.483
Odd Ratio = 1.38	Sum chi = 2.0222	p = 0.155008528207		
chromosome13	27	22.06	244	248.94
Other	802	806.94	9111	9106.06
Odd Ratio = 1.26	Sum chi = 1.2371	p = 0.266012945077		
chromosome14 *	14	18.59	286	281.41
Other	604	599.41	9069	9073.59
Odd Ratio = 0.73	Sum chi = 1.2457	p = 0.264369145185		
chromosome14	22	25.072	286	282.928
Other	807	803.928	9069	9072.072
Odd Ratio = 0.86	Sum chi = 0.4225	p = 0.515689267447		
chromosome15 *	26	13.137	186	198.863
Other	592	604.863	9169	9156.137
Odd Ratio = 2.17	Sum chi = 13.718	p = 0.000212393903		
chromosome15	32	17.746	186	200.254
Other	797	811.254	9169	9154.746
Odd Ratio = 1.98	Sum chi = 12.737	p = 0.000358462212		

Odd Ratio = 1.38	Sum chi = 8.31320	p = 0.003935779296		
chromosome8 *	54	61.546	523	515.454
Other	1063	1055.454	8832	8839.546
Odd Ratio = 0.86	Sum chi = 1.09603	p = 0.295136973305		
chromosome8	91	95.778	523	518.222
Other	1638	1633.222	8832	8836.778
Odd Ratio = 0.94	Sum chi = 0.29900	p = 0.584509905241		
chromosome9 *	51	41.706	340	349.294
Other	1066	1075.294	9015	9005.706
Odd Ratio = 1.27	Sum chi = 2.40824	p = 0.120697417406		
chromosome9	67	63.488	340	343.512
Other	1662	1665.512	9015	9011.488
Odd Ratio = 1.07	Sum chi = 0.23893	p = 0.624979740585		
chromosome10 *	48	39.466	322	330.534
Other	1069	1077.534	9033	9024.466
Odd Ratio = 1.26	Sum chi = 2.14125	p = 0.143384628376		
chromosome10	96	65.204	322	352.796
Other	1633	1663.796	9033	9002.204
Odd Ratio = 1.65	Sum chi = 17.9085	p = 0.000023178845		
chromosome11 *	54	58.559	495	490.441
Other	1063	1058.441	8860	8864.559
Odd Ratio = 0.91	Sum chi = 0.41934	p = 0.517263257498		
chromosome11	79	89.539	495	484.461
Other	1650	1639.461	8860	8870.539
Odd Ratio = 0.86	Sum chi = 1.54989	p = 0.213150471450		
chromosome12 *	57	50.559	417	423.441
Other	1060	1066.441	8938	8931.559
Odd Ratio = 1.15	Sum chi = 0.96195	p = 0.326695456896		
chromosome12	78	77.215	417	417.785
Other	1651	1651.785	8938	8937.215
Odd Ratio = 1.01	Sum chi = 0.00988	p = 0.920787625507		
chromosome13 *	34	29.653	244	248.347
Other	1083	1087.347	9111	9106.653
Odd Ratio = 1.17	Sum chi = 0.73280	p = 0.391976856190		
chromosome13	49	45.705	244	247.295
Other	1680	1683.295	9111	9107.705
Odd Ratio = 1.09	Sum chi = 0.28904	p = 0.590832863562		
chromosome14 *	30	33.706	286	282.294
Other	1087	1083.294	9069	9072.706
Odd Ratio = 0.88	Sum chi = 0.47038	p = 0.492809369474		
chromosome14	46	51.789	286	280.211
Other	1683	1677.211	9069	9074.789
Odd Ratio = 0.87	Sum chi = 0.79033	p = 0.373997727109		
chromosome15 *	34	23.466	186	196.534
Other	1083	1093.534	9169	9158.466
Odd Ratio = 1.55	Sum chi = 5.40649	p = 0.020062013848		
chromosome15	44	35.878	186	194.122
Other	1685	1693.122	9169	9160.878
Odd Ratio = 1.29	Sum chi = 2.22472	p = 0.135816994436		

Odd Ratio = 0.94	Sum chi = 0.114	p = 0.734641486199		
chromosome8 *	34	31.345	54	56.655
Other	584	586.655	1063	1060.345
Odd Ratio = 1.15	Sum chi = 0.367	p = 0.544151346506		
chromosome8	47	44.723	91	93.277
Other	782	784.277	1638	1635.723
Odd Ratio = 1.08	Sum chi = 0.181	p = 0.670293602075		
chromosome9 *	26	27.427	51	49.573
Other	592	590.573	1066	1067.427
Odd Ratio = 0.92	Sum chi = 0.120	p = 0.728283553068		
chromosome9	37	33.704	67	70.296
Other	792	795.296	1662	1658.704
Odd Ratio = 1.16	Sum chi = 0.496	p = 0.480850627169		
chromosome10 *	24	25.646	48	46.354
Other	594	592.354	1069	1070.646
Odd Ratio = 0.90	Sum chi = 0.171	p = 0.679031568365		
chromosome10	36	42.779	96	89.221
Other	793	786.221	1633	1639.779
Odd Ratio = 0.77	Sum chi = 1.675	p = 0.195502870781		
chromosome11 *	37	32.414	54	58.586
Other	581	585.586	1063	1058.414
Odd Ratio = 1.25	Sum chi = 1.063	p = 0.302376053282		
chromosome11	53	42.779	79	89.221
Other	776	786.221	1650	1639.779
Odd Ratio = 1.43	Sum chi = 3.809	p = 0.050954968981		
chromosome12 *	35	32.77	57	59.23
Other	583	585.23	1060	1057.77
Odd Ratio = 1.12	Sum chi = 0.248	p = 0.617848663877		
chromosome12	42	38.89	78	81.11
Other	787	790.11	1651	1647.89
Odd Ratio = 1.13	Sum chi = 0.386	p = 0.534344415750		
chromosome13 *	22	19.947	34	36.053
Other	596	598.053	1083	1080.947
Odd Ratio = 1.18	Sum chi = 0.339	p = 0.560313443960		
chromosome13	27	24.63	49	51.37
Other	802	804.37	1680	1677.63
Odd Ratio = 1.15	Sum chi = 0.347	p = 0.555434925740		
chromosome14 *	14	15.673	30	28.327
Other	604	602.327	1087	1088.673
Odd Ratio = 0.84	Sum chi = 0.284	p = 0.593777822846		
chromosome14	22	22.038	46	45.962
Other	807	806.962	1683	1683.038
Odd Ratio = 1.00	Sum chi = 0.000	p = 0.992136323450		
chromosome15 *	26	21.372	34	38.628
Other	592	596.628	1083	1078.372
Odd Ratio = 1.40	Sum chi = 1.612	p = 0.204128684314		
chromosome15	32	24.63	44	51.37
Other	797	804.37	1685	1677.63
Odd Ratio = 1.54	Sum chi = 3.362	p = 0.066700438911		

chromosome16 *	14	18.486	124	119.514
Other	1433	1428.514	9231	9235.486
Odd Ratio = 0.73	Sum chi = 1.273	p = 0.259152429793		
chromosome16	20	30.806	124	113.194
Other	2526	2515.194	9231	9241.806
Odd Ratio = 0.59	Sum chi = 4.881	p = 0.027149924960		
chromosome17 *	17	10.984	65	71.016
Other	1430	1436.016	9290	9283.984
Odd Ratio = 1.70	Sum chi = 3.833	p = 0.050251904047		
chromosome17	22	18.612	65	68.388
Other	2524	2527.388	9290	9286.612
Odd Ratio = 1.25	Sum chi = 0.790	p = 0.374001666166		
chromosome18 *	30	28.667	184	185.333
Other	1417	1418.333	9171	9169.667
Odd Ratio = 1.06	Sum chi = 0.073	p = 0.786949174465		
chromosome18	51	50.274	184	184.726
Other	2495	2495.726	9171	9170.274
Odd Ratio = 1.02	Sum chi = 0.013	p = 0.907132097278		
chromosome19 *	12	9.913	62	64.087
Other	1435	1437.087	9293	9290.913
Odd Ratio = 1.25	Sum chi = 0.510	p = 0.474726896927		
chromosome19	12	15.831	62	58.169
Other	2534	2530.169	9293	9296.831
Odd Ratio = 0.71	Sum chi = 1.186	p = 0.275989973931		
chromosome20 *	21	16.343	101	105.657
Other	1426	1430.657	9254	9249.343
Odd Ratio = 1.35	Sum chi = 1.550	p = 0.213133769327		
chromosome20	29	27.811	101	102.189
Other	2517	2518.189	9254	9252.811
Odd Ratio = 1.06	Sum chi = 0.065	p = 0.798201910783		
chromosome21 *	6	8.305	56	53.695
Other	1441	1438.695	9299	9301.305
Odd Ratio = 0.69	Sum chi = 0.743	p = 0.388660318388		
chromosome21	10	14.119	56	51.881
Other	2536	2531.881	9299	9303.119
Odd Ratio = 0.65	Sum chi = 1.537	p = 0.214986685028		
chromosome22 *	4	4.287	28	27.713
Other	1443	1442.713	9327	9327.287
Odd Ratio = 0.92	Sum chi = 0.012	p = 0.911685020465 with Yates' chi-square test		
chromosome22	7	7.488	28	27.512
Other	2539	2538.512	9327	9327.488
Odd Ratio = 0.92	Sum chi = 0.040	p = 0.840477326560		
chromosomeX *	86	158.739	1099	1026.261
Other	1361	1288.261	8256	8328.739
Odd Ratio = 0.47	Sum chi = 43.22	p = 4.86995E-11		
chromosomeX	176	272.763	1099	1002.237
Other	2370	2273.237	8256	8352.763
Odd Ratio = 0.56	Sum chi = 48.90	p = 2.68176E-12		
chromosomeY *	23	31.614	213	204.386
Other	1424	1415.386	9142	9150.614

chromosome16 *	5	7.994	124	121.006
Other	613	610.006	9231	9233.994
Odd Ratio = 0.61	Sum chi = 1.2109	p = 0.271144962338		
chromosome16	5	10.501	124	118.499
Other	824	818.499	9231	9236.501
Odd Ratio = 0.45	Sum chi = 3.1772	p = 0.074670767496		
chromosome17 *	12	4.771	65	72.229
Other	606	613.229	9290	9282.771
Odd Ratio = 2.83	Sum chi = 10.193	p = 0.001409183403 with Yates' chi-square test		
chromosome17	13	6.349	65	71.651
Other	816	822.651	9290	9283.349
Odd Ratio = 2.28	Sum chi = 7.6420	p = 0.005702394594		
chromosome18 *	12	12.146	184	183.854
Other	606	605.854	9171	9171.146
Odd Ratio = 0.99	Sum chi = 0.0018	p = 0.965251564495		
chromosome18	18	16.443	184	185.557
Other	811	812.557	9171	9169.443
Odd Ratio = 1.11	Sum chi = 0.1636	p = 0.685779406063		
chromosome19 *	2	3.966	62	60.034
Other	616	614.034	9293	9294.966
Odd Ratio = 0.49	Sum chi = 0.5813	p = 0.445777830383 with Yates' chi-square test		
chromosome19	2	5.21	62	58.79
Other	827	823.79	9293	9296.21
Odd Ratio = 0.36	Sum chi = 2.1663	p = 0.141057188200		
chromosome20 *	13	7.064	101	106.936
Other	605	610.936	9254	9248.064
Odd Ratio = 1.97	Sum chi = 5.3784	p = 0.020387257889		
chromosome20	15	9.443	101	106.557
Other	814	819.557	9254	9248.443
Odd Ratio = 1.69	Sum chi = 3.6015	p = 0.057725466818		
chromosome21 *	3	3.656	56	55.344
Other	615	614.344	9299	9299.656
Odd Ratio = 0.81	Sum chi = 0.0071	p = 0.932637439473 with Yates' chi-square test		
chromosome21	3	4.803	56	54.197
Other	826	824.197	9299	9300.803
Odd Ratio = 0.60	Sum chi = 0.3869	p = 0.533924109490 with Yates' chi-square test		
chromosome22 *	2	1.859	28	28.141
Other	616	616.141	9327	9326.859
Odd Ratio = 1.08	Sum chi = 0.0741	p = 0.785404146978 with Yates' chi-square test		
chromosome22	3	2.523	28	28.477
Other	826	826.477	9327	9326.523
Odd Ratio = 1.21	Sum chi = 0.0002	p = 0.987683063383 with Yates' chi-square test		
chromosomeX *	40	70.581	1099	1068.419
Other	578	547.419	8256	8286.581
Odd Ratio = 0.52	Sum chi = 15.946	p = 0.000065163826		
chromosomeX	59	94.264	1099	1063.736
Other	770	734.736	8256	8291.264
Odd Ratio = 0.58	Sum chi = 16.203	p = 0.000056887861		
chromosomeY *	6	13.571	213	205.429
Other	612	604.429	9142	9149.571

chromosome16 *	9	14.186	124	118.814
Other	1108	1102.814	9231	9236.186
Odd Ratio = 0.60	Sum chi = 2.14985	p = 0.142582937839		
chromosome16	15	21.683	124	117.317
Other	1714	1707.317	9231	9237.683
Odd Ratio = 0.65	Sum chi = 2.47128	p = 0.115942786877		
chromosome17 *	7	7.68	65	64.32
Other	1110	1109.32	9290	9290.68
Odd Ratio = 0.90	Sum chi = 0.06784	p = 0.794498616655		
chromosome17	9	11.543	65	62.457
Other	1720	1717.457	9290	9292.543
Odd Ratio = 0.75	Sum chi = 0.66838	p = 0.413614080729		
chromosome18 *	24	22.186	184	185.814
Other	1093	1094.814	9171	9169.186
Odd Ratio = 1.09	Sum chi = 0.16931	p = 0.680721773485		
chromosome18	33	33.85	184	183.15
Other	1696	1695.15	9171	9171.85
Odd Ratio = 0.97	Sum chi = 0.02579	p = 0.872409728227		
chromosome19 *	10	7.68	62	64.32
Other	1107	1109.32	9293	9290.68
Odd Ratio = 1.35	Sum chi = 0.79001	p = 0.374095010401		
chromosome19	10	11.231	62	60.769
Other	1719	1717.769	9293	9294.231
Odd Ratio = 0.87	Sum chi = 0.16098	p = 0.688247304470		
chromosome20 *	12	12.053	101	100.947
Other	1105	1104.947	9254	9254.053
Odd Ratio = 1.00	Sum chi = 0.00026	p = 0.986996980097		
chromosome20	14	17.939	101	97.061
Other	1715	1711.061	9254	9257.939
Odd Ratio = 0.75	Sum chi = 1.03547	p = 0.308875758719		
chromosome21 *	5	6.507	56	54.493
Other	1112	1110.493	9299	9300.507
Odd Ratio = 0.75	Sum chi = 0.39278	p = 0.530837053555		
chromosome21	7	9.827	56	53.173
Other	1722	1719.173	9299	9301.827
Odd Ratio = 0.68	Sum chi = 0.96931	p = 0.324850230972		
chromosome22 *	4	3.413	28	28.587
Other	1113	1113.587	9327	9326.413
Odd Ratio = 1.20	Sum chi = 0.00247	p = 0.960336775216 with Yates' chi-square test		
chromosome22	4	4.992	28	27.008
Other	1725	1724.008	9327	9327.992
Odd Ratio = 0.77	Sum chi = 0.05755	p = 0.810407912290 with Yates' chi-square test		
chromosomeX *	72	124.905	1099	1046.095
Other	1045	992.095	8256	8308.905
Odd Ratio = 0.52	Sum chi = 28.2424	p = 0.000000106877		
chromosomeX	117	189.685	1099	1026.315
Other	1612	1539.315	8256	8328.685
Odd Ratio = 0.55	Sum chi = 37.0657	p = 1.14213E-9		
chromosomeY *	19	24.746	213	207.254
Other	1098	1092.254	9142	9147.746

chromosome16 *	5	4.987	9	9.013
Other	613	613.013	1108	1107.987
Odd Ratio = 1.00	Sum chi = 0.074	p = 0.785040879183 with Yates' chi-square test		
chromosome16	5	6.482	15	13.518
Other	824	822.518	1714	1715.482
Odd Ratio = 0.69	Sum chi = 0.505	p = 0.477303026286		
chromosome17 *	12	6.768	7	12.232
Other	606	611.232	1110	1104.768
Odd Ratio = 3.14	Sum chi = 6.352	p = 0.011719413446		
chromosome17	13	7.13	9	14.87
Other	816	821.87	1720	1714.13
Odd Ratio = 3.04	Sum chi = 7.212	p = 0.007239640524		
chromosome18 *	12	12.823	24	23.177
Other	606	605.177	1093	1093.823
Odd Ratio = 0.90	Sum chi = 0.083	p = 0.772218866964		
chromosome18	18	16.528	33	34.472
Other	811	812.472	1696	1694.528
Odd Ratio = 1.14	Sum chi = 0.197	p = 0.656454334517		
chromosome19 *	2	4.274	10	7.726
Other	616	613.726	1107	1109.274
Odd Ratio = 0.36	Sum chi = 1.152	p = 0.283121368523 with Yates' chi-square test		
chromosome19	2	3.889	10	8.111
Other	827	825.111	1719	1720.889
Odd Ratio = 0.42	Sum chi = 0.737	p = 0.390495760910 with Yates' chi-square test		
chromosome20 *	13	8.905	12	16.095
Other	605	609.095	1105	1100.905
Odd Ratio = 1.98	Sum chi = 2.967	p = 0.084931821478		
chromosome20	15	9.398	14	19.602
Other	814	819.602	1715	1709.398
Odd Ratio = 2.26	Sum chi = 4.996	p = 0.025403686188		
chromosome21 *	3	2.85	5	5.15
Other	615	615.15	1112	1111.85
Odd Ratio = 1.08	Sum chi = 0.066	p = 0.795879529511 with Yates' chi-square test		
chromosome21	3	3.241	7	6.759
Other	826	825.759	1722	1722.241
Odd Ratio = 0.89	Sum chi = 0.030	p = 0.860714643363 with Yates' chi-square test		
chromosome22 *	2	2.137	4	3.863
Other	616	615.863	1113	1113.137
Odd Ratio = 0.90	Sum chi = 0.096	p = 0.756675011886 with Yates' chi-square test		
chromosome22	3	2.269	4	4.731
Other	826	826.731	1725	1724.269
Odd Ratio = 1.57	Sum chi = 0.035	p = 0.851541967406 with Yates' chi-square test		
chromosomeX *	40	39.894	72	72.106
Other	578	578.106	1045	1044.894
Odd Ratio = 1.00	Sum chi = 0.000	p = 0.982738257014		
chromosomeX	59	57.038	117	118.962
Other	770	771.962	1612	1610.038
Odd Ratio = 1.06	Sum chi = 0.107	p = 0.743365208145		
chromosomeY *	6	8.905	19	16.095
Other	612	609.095	1098	1100.905

Odd Ratio = 0.69	Sum chi = 2.770	p = 0.096013677221		
chromosomeY	32	52.413	213	192.587
Other	2514	2493.587	9142	9162.413
Odd Ratio = 0.55	Sum chi = 10.32	p = 0.001311279307		

ORF1 conservation

	intragenic L1s		intergenic L1s	
Case	Observed	Expected	Observed	Expected
not conserved *	7	9.265	62	59.735
Other	1444	1441.735	9293	9295.265
Odd Ratio = 0.73	Sum chi = 0.643	p = 0.422344983983		
not conserved	7	14.761	62	54.239
Other	2539	2531.239	9293	9300.761
Odd Ratio = 0.41	Sum chi = 5.221	p = 0.022307385948		
conserved *	1444	1441.735	9293	9295.265
Other	7	9.265	62	59.735
Odd Ratio = 1.38	Sum chi = 0.643	p = 0.422344983983		
conserved	2539	2531.239	9293	9300.761
Other	7	14.761	62	54.239
Odd Ratio = 2.42	Sum chi = 5.221	p = 0.022307385948		

ORF2 conservation

	intragenic L1s		intergenic L1s	
Case	Observed	Expected	Observed	Expected
not conserved *	0	0.134	1	0.866
Other	1446	1445.866	9354	9354.134
Odd Ratio = 0.00	Sum chi = 1.156	p = 0.282267035515 with Yates' chi-square test		
not conserved	0	0.214	1	0.786
Other	2546	2545.786	9354	9354.214
Odd Ratio = 0.00	Sum chi = 0.486	p = 0.485413296139 with Yates' chi-square test		
conserved *	1446	1445.866	9354	9354.134
Other	0	0.134	1	0.866
Odd Ratio = Error	Sum chi = 1.156	p = 0.282267035515 with Yates' chi-square test		
conserved	2546	2545.786	9354	9354.214
Other	0	0.214	1	0.786
Odd Ratio = Error	Sum chi = 0.486	p = 0.485413296139 with Yates' chi-square test		

ORF StartStop

	intragenic L1s		intergenic L1s	
Case	Observed	Expected	Observed	Expected
ORF1 cons *	298	272.491	1129	1154.509
Other	1910	1935.509	8226	8200.491
Odd Ratio = 1.14	Sum chi = 3.367	p = 0.066510287040		
ORF1 cons	300	305.708	1129	1123.292
Other	2246	2240.292	8226	8231.708
Odd Ratio = 0.97	Sum chi = 0.154	p = 0.694650602321		
ORF2 cons *	483	448.742	1867	1901.258
Other	1725	1759.258	7488	7453.742
Odd Ratio = 1.12	Sum chi = 4.057	p = 0.043982011819		
ORF2 cons	492	504.665	1867	1854.335
Other	2054	2041.335	7488	7500.665
Odd Ratio = 0.96	Sum chi = 0.504	p = 0.477627839806		
cons *	816	843.825	3603	3575.175
Other	1392	1364.175	5752	5779.825
Odd Ratio = 0.94	Sum chi = 1.835	p = 0.175465005079		

Odd Ratio = 0.42	Sum chi = 4.6036	p = 0.031903115331		
chromosomeY	7	17.908	213	202.092
Other	822	811.092	9142	9152.908
Odd Ratio = 0.37	Sum chi = 7.3931	p = 0.006547291318		

ORF1 conservation

	sense L1s		intergenic L1s	
Case	Observed	Expected	Observed	Expected
not conserved *	5	4.164	62	62.836
Other	615	615.836	9293	9292.164
Odd Ratio = 1.22	Sum chi = 0.0290	p = 0.864708127411 with Yates' chi-square test		
not conserved	5	5.454	62	61.546
Other	824	823.546	9293	9293.454
Odd Ratio = 0.91	Sum chi = 0.0414	p = 0.838760271008		
conserved *	615	615.836	9293	9292.164
Other	5	4.164	62	62.836
Odd Ratio = 0.82	Sum chi = 0.0290	p = 0.864708127411 with Yates' chi-square test		
conserved	824	823.546	9293	9293.454
Other	5	5.454	62	61.546
Odd Ratio = 1.10	Sum chi = 0.0414	p = 0.838760271008		

ORF2 conservation

	sense L1s		intergenic L1s	
Case	Observed	Expected	Observed	Expected
not conserved *	0	0.062	1	0.938
Other	618	617.938	9354	9354.062
Odd Ratio = 0.00	Sum chi = 3.3012	p = 0.069227969861 with Yates' chi-square test		
not conserved	0	0.081	1	0.919
Other	829	828.919	9354	9354.081
Odd Ratio = 0.00	Sum chi = 2.3435	p = 0.125802237027 with Yates' chi-square test		
conserved *	618	617.938	9354	9354.062
Other	0	0.062	1	0.938
Odd Ratio = Error	Sum chi = 3.3012	p = 0.069227969861 with Yates' chi-square test		
conserved	829	828.919	9354	9354.081
Other	0	0.081	1	0.919
Odd Ratio = Error	Sum chi = 2.3435	p = 0.125802237027 with Yates' chi-square test		

ORF StartStop

	sense L1s		intergenic L1s	
Case	Observed	Expected	Observed	Expected
ORF1 cons *	99	93.164	1129	1134.836
Other	669	674.836	8226	8220.164
Odd Ratio = 1.08	Sum chi = 0.4501	p = 0.502273225524		
ORF1 cons	99	99.962	1129	1128.038
Other	730	729.038	8226	8226.962
Odd Ratio = 0.99	Sum chi = 0.0114	p = 0.914755978746		
ORF2 cons *	161	153.858	1867	1874.142
Other	607	614.142	7488	7480.858
Odd Ratio = 1.06	Sum chi = 0.4486	p = 0.502988383388		
ORF2 cons	164	165.328	1867	1865.672
Other	665	663.672	7488	7489.328
Odd Ratio = 0.99	Sum chi = 0.0145	p = 0.904145885607		
cons *	311	296.943	3603	3617.057
Other	457	471.057	5752	5737.943
Odd Ratio = 1.09	Sum chi = 1.1740	p = 0.278575584492		

Odd Ratio = 0.74	Sum chi = 1.52753	p = 0.216482674658		
chromosomeY	25	37.126	213	200.874
Other	1704	1691.874	9142	9154.126
Odd Ratio = 0.63	Sum chi = 4.79537	p = 0.028536234482		

ORF1 conservation

	antisense L1s		intergenic L1s	
Case	Observed	Expected	Observed	Expected
not conserved *	2	6.838	62	57.162
Other	1117	1112.162	9293	9297.838
Odd Ratio = 0.27	Sum chi = 3.85545	p = 0.049584509798		
not conserved	2	9.983	62	54.017
Other	1727	1719.017	9293	9300.983
Odd Ratio = 0.17	Sum chi = 7.60790	p = 0.005811306132		
conserved *	1117	1112.162	9293	9297.838
Other	2	6.838	62	57.162
Odd Ratio = 3.73	Sum chi = 3.85545	p = 0.049584509798		
conserved	1727	1719.017	9293	9300.983
Other	2	9.983	62	54.017
Odd Ratio = 5.76	Sum chi = 7.60790	p = 0.005811306132		

ORF2 conservation

	antisense L1s		intergenic L1s	
Case	Observed	Expected	Observed	Expected
not conserved *	0	0.107	1	0.893
Other	1117	1116.893	9354	9354.107
Odd Ratio = 0.00	Sum chi = 1.62378	p = 0.202565054933 with Yates' chi-square test		
not conserved	0	0.156	1	0.844
Other	1729	1728.844	9354	9354.156
Odd Ratio = 0.00	Sum chi = 0.89894	p = 0.343064259359 with Yates' chi-square test		
conserved *	1117	1116.893	9354	9354.107
Other	0	0.107	1	0.893
Odd Ratio = Error	Sum chi = 1.62378	p = 0.202565054933 with Yates' chi-square test		
conserved	1729	1728.844	9354	9354.156
Other	0	0.156	1	0.844
Odd Ratio = Error	Sum chi = 0.89894	p = 0.343064259359 with Yates' chi-square test		

ORF StartStop

	antisense L1s		intergenic L1s	
Case	Observed	Expected	Observed	Expected
ORF1 cons *	200	188.69	1129	1140.31
Other	1348	1359.31	8226	8214.69
Odd Ratio = 1.08	Sum chi = 0.89969	p = 0.342863472300		
ORF1 cons	202	207.624	1129	1123.376
Other	1527	1521.376	8226	8231.624
Odd Ratio = 0.96	Sum chi = 0.20509	p = 0.650641401812		
ORF2 cons *	325	311.219	1867	1880.781
Other	1223	1236.781	7488	7474.219
Odd Ratio = 1.07	Sum chi = 0.89023	p = 0.345414174961		
ORF2 cons	329	342.555	1867	1853.445
Other	1400	1386.445	7488	7501.555
Odd Ratio = 0.94	Sum chi = 0.79257	p = 0.373323527200		
cons *	608	597.875	3603	3613.125
Other	940	950.125	5752	5741.875
Odd Ratio = 1.03	Sum chi = 0.32560	p = 0.568256016975		

Odd Ratio = 0.57	Sum chi = 1.493	p = 0.221686147881		
chromosomeY	7	10.371	25	21.629
Other	822	818.629	1704	1707.371
Odd Ratio = 0.58	Sum chi = 1.641	p = 0.200149393105		

ORF1 conservation

	sense L1s		antisense L1s	
Case	Observed	Expected	Observed	Expected
not conserved *	5	2.496	2	4.504
Other	615	617.504	1117	1114.496
Odd Ratio = 4.54	Sum chi = 2.511	p = 0.113006338593 with Yates' chi-square test		
not conserved	5	2.269	2	4.731
Other	824	826.731	1727	1724.269
Odd Ratio = 5.24	Sum chi = 3.256	p = 0.071154041547 with Yates' chi-square test		
conserved *	615	617.504	1117	1114.496
Other	5	2.496	2	4.504
Odd Ratio = 0.22	Sum chi = 2.511	p = 0.113006338593 with Yates' chi-square test		
conserved	824	826.731	1727	1724.269
Other	5	2.269	2	4.731
Odd Ratio = 0.19	Sum chi = 3.256	p = 0.071154041547 with Yates' chi-square test		

ORF2 conservation

	sense L1s		antisense L1s	
Case	Observed	Expected	Observed	Expected
not conserved *	618	618	1117	1117
Other	0	0	0	0
Odd Ratio = Error	Sum chi = 3.256	p = 0.071154041547 with Yates' chi-square test		
conserved	829	829	1729	1729
Other	0	0	0	0
Odd Ratio = Error	Sum chi = 3.256	p = Error with Yates' chi-square test		

ORF StartStop

	sense L1s		antisense L1s	
Case	Observed	Expected	Observed	Expected
ORF1 cons *	99	99.15	200	199.85
Other	669	668.85	1348	1348.15
Odd Ratio = 1.00	Sum chi = 0.000	p = 0.984220049489		
ORF1 cons	99	97.548	202	203.452
Other	730	731.452	1527	1525.548
Odd Ratio = 1.03	Sum chi = 0.036	p = 0.849069713099		
ORF2 cons *	161	161.161	325	324.839
Other	607	606.839	1223	1223.161
Odd Ratio = 1.00	Sum chi = 0.000	p = 0.986109413877		
ORF2 cons	164	159.772	329	333.228
Other	665	669.228	1400	1395.772
Odd Ratio = 1.05	Sum chi = 0.205	p = 0.650683658836		
cons *	311	304.746	608	614.254
Other	457	463.254	940	933.746
Odd Ratio = 1.05	Sum chi = 0.318	p = 0.572615672015		

cons	1143	1015.319	3603	3730.681
Other	1403	1530.681	5752	5624.319
Odd Ratio = 1.30	Sum chi = 33.97	p = 0.000000005556		
mut *	611	642.942	2756	2724.058
Other	1597	1565.058	6599	6630.942
Odd Ratio = 0.92	Sum chi = 2.767	p = 0.096213642361		
mut	611	720.308	2756	2646.692
Other	1935	1825.692	6599	6708.308
Odd Ratio = 0.76	Sum chi = 29.42	p = 0.000000057969		
Ta SSVs				
	intragenic L1s		intergenic L1s	
Case	Observed	Expected	Observed	Expected
AAGA *	124	118.486	511	516.514
Other	2022	2027.514	8844	8838.486
Odd Ratio = 1.06	Sum chi = 0.333	p = 0.563383933080		
AAGA	130	137.13	511	503.87
Other	2416	2408.87	8844	8851.13
Odd Ratio = 0.93	Sum chi = 0.498	p = 0.480167638680		
ACAG *	54	38.998	155	170.002
Other	2092	2107.002	9200	9184.998
Odd Ratio = 1.53	Sum chi = 7.226	p = 0.007183745561		
ACAG	59	45.781	155	168.219
Other	2487	2500.219	9200	9186.781
Odd Ratio = 1.41	Sum chi = 4.944	p = 0.026176855178		
ACGA *	19	13.621	54	59.379
Other	2127	2132.379	9301	9295.621
Odd Ratio = 1.54	Sum chi = 2.627	p = 0.105002892231		
ACGA	21	16.045	54	58.955
Other	2525	2529.955	9301	9296.045
Odd Ratio = 1.43	Sum chi = 1.959	p = 0.161608394724		
ACGG *	30	25.003	104	108.997
Other	2116	2120.997	9251	9246.003
Odd Ratio = 1.26	Sum chi = 1.242	p = 0.265079473722		
ACGG	33	29.309	104	107.691
Other	2513	2516.691	9251	9247.309
Odd Ratio = 1.17	Sum chi = 0.598	p = 0.439212186796		
GAGA *	883	893.218	3904	3893.782
Other	1263	1252.782	5451	5461.218
Odd Ratio = 0.98	Sum chi = 0.246	p = 0.619785557156		
GAGA	1228	1097.897	3904	4034.103
Other	1318	1448.103	5451	5320.897
Odd Ratio = 1.30	Sum chi = 34.48	p = 0.000000004302		
GAGG *	15	13.621	58	59.379
Other	2131	2132.379	9297	9295.621
Odd Ratio = 1.13	Sum chi = 0.172	p = 0.677751757085		
GAGG	15	15.617	58	57.383
Other	2531	2530.383	9297	9297.617
Odd Ratio = 0.95	Sum chi = 0.031	p = 0.859788611447		
GCGA *	6	6.158	27	26.842
Other	2140	2139.842	9328	9328.158

cons	369	323.33	3603	3648.67
Other	460	505.67	5752	5706.33
Odd Ratio = 1.28	Sum chi = 11.512	p = 0.000691127312		
mut *	197	224.035	2756	2728.965
Other	571	543.965	6599	6626.035
Odd Ratio = 0.83	Sum chi = 4.9840	p = 0.025581496447		
mut	197	240.381	2756	2712.619
Other	632	588.619	6599	6642.381
Odd Ratio = 0.75	Sum chi = 12.002	p = 0.000531164349		
Ta SSVs				
	sense L1s		intergenic L1s	
Case	Observed	Expected	Observed	Expected
AAGA *	37	41.174	511	506.826
Other	723	718.826	8844	8848.174
Odd Ratio = 0.89	Sum chi = 0.4838	p = 0.486693242173		
AAGA	38	44.69	511	504.31
Other	791	784.31	8844	8850.69
Odd Ratio = 0.83	Sum chi = 1.1522	p = 0.283071259992		
ACAG *	21	13.224	155	162.776
Other	739	746.776	9200	9192.224
Odd Ratio = 1.69	Sum chi = 5.0315	p = 0.024888945041		
ACAG	23	14.49	155	163.51
Other	806	814.51	9200	9191.49
Odd Ratio = 1.69	Sum chi = 5.5383	p = 0.018604553182		
ACGA *	8	4.658	54	57.342
Other	752	755.342	9301	9297.658
Odd Ratio = 1.83	Sum chi = 1.8856	p = 0.169688891774 with Yates' chi-square test		
ACGA	8	5.047	54	56.953
Other	821	823.953	9301	9298.047
Odd Ratio = 1.68	Sum chi = 1.8925	p = 0.168916022384		
ACGG *	7	8.34	104	102.66
Other	753	751.66	9251	9252.34
Odd Ratio = 0.83	Sum chi = 0.2354	p = 0.627546645296		
ACGG	7	9.036	104	101.964
Other	822	819.964	9251	9253.036
Odd Ratio = 0.76	Sum chi = 0.5047	p = 0.477419183108		
GAGA *	335	318.501	3904	3920.499
Other	425	441.499	5451	5434.501
Odd Ratio = 1.10	Sum chi = 1.5907	p = 0.207221118547		
GAGA	397	350.111	3904	3950.889
Other	432	478.889	5451	5404.111
Odd Ratio = 1.28	Sum chi = 11.834	p = 0.000581577126		
GAGG *	3	4.583	58	56.417
Other	757	755.417	9297	9298.583
Odd Ratio = 0.64	Sum chi = 0.2785	p = 0.597670223725 with Yates' chi-square test		
GAGG	3	4.966	58	56.034
Other	826	824.034	9297	9298.966
Odd Ratio = 0.58	Sum chi = 0.4737	p = 0.491286763438 with Yates' chi-square test		
GCGA *	1	2.104	27	25.896
Other	759	757.896	9328	9329.104

cons	783	684.175	3603	3701.825
Other	946	1044.825	5752	5653.175
Odd Ratio = 1.32	Sum chi = 27.9880	p = 0.000000121921		
mut *	415	450.216	2756	2720.784
Other	1133	1097.784	6599	6634.216
Odd Ratio = 0.88	Sum chi = 4.52711	p = 0.033361734269		
mut	415	494.646	2756	2676.354
Other	1314	1234.354	6599	6678.646
Odd Ratio = 0.76	Sum chi = 21.2835	p = 0.000003960773		
Ta SSVs				
	antisense L1s		intergenic L1s	
Case	Observed	Expected	Observed	Expected
AAGA *	90	84.002	511	516.998
Other	1430	1435.998	8844	8838.002
Odd Ratio = 1.09	Sum chi = 0.52701	p = 0.467864714127		
AAGA	93	94.218	511	509.782
Other	1636	1634.782	8844	8845.218
Odd Ratio = 0.98	Sum chi = 0.01974	p = 0.888260898650		
ACAG *	33	26.277	155	161.723
Other	1487	1493.723	9200	9193.277
Odd Ratio = 1.32	Sum chi = 2.03489	p = 0.153725183030		
ACAG	36	29.794	155	161.206
Other	1693	1699.206	9200	9193.794
Odd Ratio = 1.26	Sum chi = 1.55834	p = 0.211907323567		
ACGA *	11	9.085	54	55.915
Other	1509	1510.915	9301	9299.085
Odd Ratio = 1.26	Sum chi = 0.47203	p = 0.492053131192		
ACGA	13	10.451	54	56.549
Other	1716	1718.549	9301	9298.451
Odd Ratio = 1.30	Sum chi = 0.74084	p = 0.389391211610		
ACGG *	23	17.751	104	109.249
Other	1497	1502.249	9251	9245.751
Odd Ratio = 1.37	Sum chi = 1.82580	p = 0.176623780117		
ACGG	26	20.279	104	109.721
Other	1703	1708.721	9251	9245.279
Odd Ratio = 1.36	Sum chi = 1.93513	p = 0.164197417150		
GAGA *	655	637.212	3904	3921.788
Other	865	882.788	5451	5433.212
Odd Ratio = 1.06	Sum chi = 0.99390	p = 0.318789652095		
GAGA	840	740.019	3904	4003.981
Other	889	988.981	5451	5351.019
Odd Ratio = 1.32	Sum chi = 27.9799	p = 0.000000122428		
GAGG *	12	9.784	58	60.216
Other	1508	1510.216	9297	9294.784
Odd Ratio = 1.28	Sum chi = 0.58729	p = 0.443468685867		
GAGG	12	10.919	58	59.081
Other	1717	1718.081	9297	9295.919
Odd Ratio = 1.12	Sum chi = 0.12752	p = 0.721015855842		
GCGA *	5	4.473	27	27.527
Other	1515	1515.527	9328	9327.473

cons	369	373.342	783	778.658
Other	460	455.658	946	950.342
Odd Ratio = 0.97	Sum chi = 0.135	p = 0.712388596957		
mut *	197	202.943	415	409.057
Other	571	565.057	1133	1138.943
Odd Ratio = 0.94	Sum chi = 0.353	p = 0.551916380998		
mut	197	198.338	415	413.662
Other	632	630.662	1314	1315.338
Odd Ratio = 0.99	Sum chi = 0.017	p = 0.894614758292		
Ta SSVs				
	sense L1s		antisense L1s	
Case	Observed	Expected	Observed	Expected
AAGA *	37	42.333	90	84.667
Other	723	717.667	1430	1435.333
Odd Ratio = 0.81	Sum chi = 1.067	p = 0.301550233750		
AAGA	38	42.455	93	88.545
Other	791	786.545	1636	1640.455
Odd Ratio = 0.85	Sum chi = 0.728	p = 0.393255959844		
ACAG *	21	18	33	36
Other	739	742	1487	1484
Odd Ratio = 1.28	Sum chi = 0.768	p = 0.380776311582		
ACAG	23	19.121	36	39.879
Other	806	809.879	1693	1689.121
Odd Ratio = 1.34	Sum chi = 1.191	p = 0.274958292440		
ACGA *	8	6.333	11	12.667
Other	752	753.667	1509	1507.333
Odd Ratio = 1.46	Sum chi = 0.663	p = 0.415353997497		
ACGA	8	6.806	13	14.194
Other	821	822.194	1716	1714.806
Odd Ratio = 1.29	Sum chi = 0.312	p = 0.576069446375		
ACGG *	7	10	23	20
Other	753	750	1497	1500
Odd Ratio = 0.61	Sum chi = 1.368	p = 0.242155808838		
ACGG	7	10.695	26	22.305
Other	822	818.305	1703	1706.695
Odd Ratio = 0.56	Sum chi = 1.913	p = 0.166622461367		
GAGA *	335	330	655	660
Other	425	430	865	860
Odd Ratio = 1.04	Sum chi = 0.200	p = 0.654039112946		
GAGA	397	400.889	840	836.111
Other	432	428.111	889	892.889
Odd Ratio = 0.97	Sum chi = 0.108	p = 0.742364338125		
GAGG *	3	5	12	10
Other	757	755	1508	1510
Odd Ratio = 0.50	Sum chi = 1.207	p = 0.271739090716		
GAGG	3	4.861	12	10.139
Other	826	824.139	1717	1718.861
Odd Ratio = 0.52	Sum chi = 0.567	p = 0.451355629218 with Yates' chi-square test		
GCGA *	1	2	5	4
Other	759	758	1515	1516

Odd Ratio = 0.97	Sum chi = 0.004	p = 0.943795789549		
GCGA	6	7.06	27	25.94
Other	2540	2538.94	9328	9329.06
Odd Ratio = 0.82	Sum chi = 0.202	p = 0.652361873739		
Unclassified *	1015	1036.894	4542	4520.106
Other	1131	1109.106	4813	4834.894
Odd Ratio = 0.95	Sum chi = 1.099	p = 0.294329577912		
Unclassified	1054	1197.161	4542	4398.839
Other	1492	1348.839	4813	4956.161
Odd Ratio = 0.75	Sum chi = 41.10	p = 1.43976E-10		

Ta0/Ta1 SSVs

	intragenic L1s		intergenic L1s	
Case	Observed	Expected	Observed	Expected
L1PA5 *	837	758.461	3505	3583.539
Other	1143	1221.539	5850	5771.461
Odd Ratio = 1.22	Sum chi = 15.97	p = 0.000064274755		
L1PA5	1088	982.588	3505	3610.412
Other	1458	1563.412	5850	5744.588
Odd Ratio = 1.25	Sum chi = 23.42	p = 0.000001296874		
Ta-0/L1PA2 *	520	495.044	2314	2338.956
Other	1460	1484.956	7041	7016.044
Odd Ratio = 1.08	Sum chi = 2.032	p = 0.153959285199		
Ta-0/L1PA2	668	637.944	2314	2344.056
Other	1878	1908.056	7041	7010.944
Odd Ratio = 1.08	Sum chi = 2.403	p = 0.121046348036		
Ta-1 *	68	54.85	246	259.15
Other	1912	1925.15	9109	9095.85
Odd Ratio = 1.32	Sum chi = 3.929	p = 0.047459622976		
Ta-1	71	67.816	246	249.184
Other	2475	2478.184	9109	9105.816
Odd Ratio = 1.06	Sum chi = 0.195	p = 0.658509384442		
non canonical *	555	671.645	3290	3173.355
Other	1425	1308.355	6065	6181.645
Odd Ratio = 0.72	Sum chi = 37.14	p = 1.09606E-9		
non canonical	719	857.652	3290	3151.348
Other	1827	1688.348	6065	6203.652
Odd Ratio = 0.73	Sum chi = 43.00	p = 5.47189E-11		

Ta1-nd/d

	intragenic L1s		intergenic L1s	
Case	Observed	Expected	Observed	Expected
Ta1-d *	221	219.931	1091	1092.069
Other	1663	1664.069	8264	8262.931
Odd Ratio = 1.01	Sum chi = 0.007	p = 0.933021753997		
Ta1-d	238	284.315	1091	1044.685
Other	2308	2261.685	8264	8310.315
Odd Ratio = 0.78	Sum chi = 10.80	p = 0.001012448074		
Ta1-nd *	906	867.488	4269	4307.512
Other	978	1016.512	5086	5047.488
Odd Ratio = 1.10	Sum chi = 3.806	p = 0.051040579775		
Ta1-nd	1273	1185.609	4269	4356.391
Other	1273	1360.391	5086	4998.609

Odd Ratio = 0.46	Sum chi = 0.1878	p = 0.664674330904 with Yates' chi-square test		
GCGA	1	2.279	27	25.721
Other	828	826.721	9328	9329.279
Odd Ratio = 0.42	Sum chi = 0.2908	p = 0.589687655196 with Yates' chi-square test		
Unclassified *	348	367.415	4542	4522.585
Other	412	392.585	4813	4832.415
Odd Ratio = 0.90	Sum chi = 2.1473	p = 0.142813893818		
Unclassified	352	398.382	4542	4495.618
Other	477	430.618	4813	4859.382
Odd Ratio = 0.78	Sum chi = 11.317	p = 0.000767882228		

Ta0/Ta1 SSVs

	sense L1s		intergenic L1s	
Case	Observed	Expected	Observed	Expected
L1PA5 *	298	275.279	3505	3527.721
Other	432	454.721	5850	5827.279
Odd Ratio = 1.15	Sum chi = 3.2455	p = 0.071618137835		
L1PA5	342	313.154	3505	3533.846
Other	487	515.846	5850	5821.154
Odd Ratio = 1.17	Sum chi = 4.6485	p = 0.031080417928		
Ta-0/L1PA2 *	197	181.758	2314	2329.242
Other	533	548.242	7041	7025.758
Odd Ratio = 1.12	Sum chi = 1.8347	p = 0.175571088094		
Ta-0/L1PA2	219	206.192	2314	2326.808
Other	610	622.808	7041	7028.192
Odd Ratio = 1.09	Sum chi = 1.1528	p = 0.282948464796		
Ta-1 *	22	19.399	246	248.601
Other	708	710.601	9109	9106.399
Odd Ratio = 1.15	Sum chi = 0.3861	p = 0.534313060115		
Ta-1	22	21.816	246	246.184
Other	807	807.184	9109	9108.816
Odd Ratio = 1.01	Sum chi = 0.0017	p = 0.966736234468		
non canonical *	213	253.564	3290	3249.436
Other	517	476.436	6065	6105.564
Odd Ratio = 0.76	Sum chi = 10.718	p = 0.001060635641		
non canonical	246	287.838	3290	3248.162
Other	583	541.162	6065	6106.838
Odd Ratio = 0.78	Sum chi = 10.141	p = 0.001449738340		

Ta1-nd/d

	sense L1s		intergenic L1s	
Case	Observed	Expected	Observed	Expected
Ta1-d *	88	84.147	1091	1094.853
Other	631	634.853	8264	8260.147
Odd Ratio = 1.06	Sum chi = 0.2151	p = 0.642784277370		
Ta1-d	90	96.136	1091	1084.864
Other	739	732.864	8264	8270.136
Odd Ratio = 0.92	Sum chi = 0.4822	p = 0.487395984024		
Ta1-nd *	357	330.166	4269	4295.834
Other	362	388.834	5086	5059.166
Odd Ratio = 1.17	Sum chi = 4.3426	p = 0.037168827844		
Ta1-nd	420	381.695	4269	4307.305
Other	409	447.305	5086	5047.695

Odd Ratio = 1.14	Sum chi = 0.00019	p = 0.988856178782 with Yates' chi-square test		
GCGA	5	4.992	27	27.008
Other	1724	1724.008	9328	9327.992
Odd Ratio = 1.00	Sum chi = 0.05755	p = 0.810407912290 with Yates' chi-square test		
Unclassified *	691	731.417	4542	4501.583
Other	829	788.583	4813	4853.417
Odd Ratio = 0.88	Sum chi = 5.00431	p = 0.025284148264		
Unclassified	704	818.327	4542	4427.673
Other	1025	910.673	4813	4927.327
Odd Ratio = 0.73	Sum chi = 35.9297	p = 0.000000002054		

Ta0/Ta1 SSVs

	antisense L1s		intergenic L1s	
Case	Observed	Expected	Observed	Expected
L1PA5 *	619	550.783	3505	3573.217
Other	823	891.217	5850	5781.783
Odd Ratio = 1.26	Sum chi = 15.7776	p = 0.000071240693		
L1PA5	753	664.208	3505	3593.792
Other	976	1064.792	5850	5761.208
Odd Ratio = 1.29	Sum chi = 22.8363	p = 0.000001763921		
Ta-0/L1PA2 *	374	358.997	2314	2329.003
Other	1068	1083.003	7041	7025.997
Odd Ratio = 1.07	Sum chi = 0.96345	p = 0.326318168484		
Ta-0/L1PA2	452	431.47	2314	2334.53
Other	1277	1297.53	7041	7020.47
Odd Ratio = 1.08	Sum chi = 1.54225	p = 0.214282780847		
Ta-1 *	46	38.998	246	253.002
Other	1396	1403.002	9109	9101.998
Odd Ratio = 1.22	Sum chi = 1.49119	p = 0.222030442083		
Ta-1	49	46.017	246	248.983
Other	1680	1682.983	9109	9106.017
Odd Ratio = 1.08	Sum chi = 0.23533	p = 0.627595926232		
non canonical *	403	493.221	3290	3199.779
Other	1039	948.779	6065	6155.221
Odd Ratio = 0.72	Sum chi = 28.9489	p = 0.000000074184		
non canonical	475	587.305	3290	3177.695
Other	1254	1141.695	6065	6177.305
Odd Ratio = 0.70	Sum chi = 38.5327	p = 5.38433E-10		

Ta1-nd/d

	antisense L1s		intergenic L1s	
Case	Observed	Expected	Observed	Expected
Ta1-d *	142	158.304	1091	1074.696
Other	1236	1219.696	8264	8280.304
Odd Ratio = 0.87	Sum chi = 2.17649	p = 0.140133144035		
Ta1-d	149	193.428	1091	1046.572
Other	1580	1535.572	8264	8308.428
Odd Ratio = 0.71	Sum chi = 13.6137	p = 0.000224532937		
Ta1-nd *	660	632.83	4269	4296.17
Other	718	745.17	5086	5058.83
Odd Ratio = 1.10	Sum chi = 2.47497	p = 0.115671392839		
Ta1-nd	859	799.92	4269	4328.08
Other	870	929.08	5086	5026.92

Odd Ratio = 0.40	Sum chi = 0.187	p = 0.664590858793 with Yates' chi-square test		
GCGA	1	1.944	5	4.056
Other	828	827.056	1724	1724.944
Odd Ratio = 0.42	Sum chi = 0.150	p = 0.697891525419 with Yates' chi-square test		
Unclassified *	348	346.333	691	692.667
Other	412	413.667	829	827.333
Odd Ratio = 1.01	Sum chi = 0.022	p = 0.881812569424		
Unclassified	352	342.23	704	713.77
Other	477	486.77	1025	1015.23
Odd Ratio = 1.07	Sum chi = 0.702	p = 0.401851258162		

Ta0/Ta1 SSVs

	sense L1s		antisense L1s	
Case	Observed	Expected	Observed	Expected
L1PA5 *	298	308.2	619	608.8
Other	432	421.8	823	833.2
Odd Ratio = 0.92	Sum chi = 0.879	p = 0.348212629277		
L1PA5	342	354.869	753	740.131
Other	487	474.131	976	988.869
Odd Ratio = 0.91	Sum chi = 1.207	p = 0.271883715102		
Ta-0/L1PA2 *	197	191.911	374	379.089
Other	533	538.089	1068	1062.911
Odd Ratio = 1.06	Sum chi = 0.275	p = 0.599472010401		
Ta-0/L1PA2	219	217.459	452	453.541
Other	610	611.541	1277	1275.459
Odd Ratio = 1.01	Sum chi = 0.021	p = 0.882317657133		
Ta-1 *	22	22.855	46	45.145
Other	708	707.145	1396	1396.855
Odd Ratio = 0.94	Sum chi = 0.049	p = 0.823622751673		
Ta-1	22	23.01	49	47.99
Other	807	805.99	1680	1681.01
Odd Ratio = 0.93	Sum chi = 0.067	p = 0.795113229690		
non canonical *	213	207.035	403	408.965
Other	517	522.965	1039	1033.035
Odd Ratio = 1.06	Sum chi = 0.361	p = 0.547759639871		
non canonical	246	233.663	475	487.337
Other	583	595.337	1254	1241.663
Odd Ratio = 1.11	Sum chi = 1.342	p = 0.246681274817		

Ta1-nd/d

	sense L1s		antisense L1s	
Case	Observed	Expected	Observed	Expected
Ta1-d *	88	78.86	142	151.14
Other	631	640.14	1236	1226.86
Odd Ratio = 1.21	Sum chi = 1.810	p = 0.178441959857		
Ta1-d	90	77.455	149	161.545
Other	739	751.545	1580	1567.455
Odd Ratio = 1.29	Sum chi = 3.315	p = 0.068624436456		
Ta1-nd *	357	348.7	660	668.3
Other	362	370.3	718	709.7
Odd Ratio = 1.07	Sum chi = 0.583	p = 0.444821714917		
Ta1-nd	420	414.5	859	864.5
Other	409	414.5	870	864.5

Odd Ratio = 1.19	Sum chi = 15.33	p = 0.000089960061		
non canonical *	757	796.58	3995	3955.42
Other	1127	1087.42	5360	5399.58
Odd Ratio = 0.90	Sum chi = 4.093	p = 0.043047142702		
non canonical	1035	1076.076	3995	3953.924
Other	1511	1469.924	5360	5401.076
Odd Ratio = 0.92	Sum chi = 3.454	p = 0.063064487453		

L1M/L1PA Discrimination

	intragenic L1s		intergenic L1s	
Case	Observed	Expected	Observed	Expected
Mammalian L1M *	158	137.694	796	816.306
Other	1420	1440.306	8559	8538.694
Odd Ratio = 1.20	Sum chi = 3.834	p = 0.050219020803		
Mammalian L1M	173	207.3	796	761.7
Other	2373	2338.7	8559	8593.3
Odd Ratio = 0.78	Sum chi = 7.859	p = 0.005054877059		
Primate L1PA *	1379	1402.923	8341	8317.077
Other	199	175.077	1014	1037.923
Odd Ratio = 0.84	Sum chi = 4.297	p = 0.038176210396		
Primate L1PA	2331	2283.078	8341	8388.922
Other	215	262.922	1014	966.078
Odd Ratio = 1.32	Sum chi = 12.39	p = 0.000431323636		

PolyA Signal

	intragenic L1s		intergenic L1s	
Case	Observed	Expected	Observed	Expected
cons *	646	555.424	2855	2945.576
Other	1118	1208.576	6500	6409.424
Odd Ratio = 1.32	Sum chi = 25.62	p = 0.000000415098		
cons	826	787.482	2855	2893.518
Other	1720	1758.518	6500	6461.482
Odd Ratio = 1.09	Sum chi = 3.470	p = 0.062490292510		
mut *	1118	1208.576	6500	6409.424
Other	646	555.424	2855	2945.576
Odd Ratio = 0.76	Sum chi = 25.62	p = 0.000000415098		
mut	1720	1758.518	6500	6461.482
Other	826	787.482	2855	2893.518
Odd Ratio = 0.91	Sum chi = 3.470	p = 0.062490292510		

Runx3 Site

	intragenic L1s		intergenic L1s	
Case	Observed	Expected	Observed	Expected
cons *	468	369.828	1931	2029.172
Other	1237	1335.172	7424	7325.828
Odd Ratio = 1.45	Sum chi = 39.34	p = 3.55379E-10		
cons	588	538.894	1931	1980.106
Other	1958	2007.106	7424	7374.894
Odd Ratio = 1.15	Sum chi = 7.221	p = 0.007205453807		
mut *	1231	1330.239	7398	7298.761
Other	474	374.761	1957	2056.239
Odd Ratio = 0.69	Sum chi = 39.82	p = 2.78262E-10		
mut	1952	2000.26	7398	7349.74
Other	594	545.74	1957	2005.26
Odd Ratio = 0.87	Sum chi = 6.910	p = 0.008569107812		

Odd Ratio = 1.22	Sum chi = 7.7557	p = 0.005354286125		
non canonical *	274	304.686	3995	3964.314
Other	445	414.314	5360	5390.686
Odd Ratio = 0.83	Sum chi = 5.7756	p = 0.016250153583		
non canonical	319	351.169	3995	3962.831
Other	510	477.831	5360	5392.169
Odd Ratio = 0.84	Sum chi = 5.5656	p = 0.018316176428		

L1M/L1PA Discrimination

	sense L1s		intergenic L1s	
Case	Observed	Expected	Observed	Expected
Mammalian L1M *	57	55.417	796	797.583
Other	593	594.583	8559	8557.417
Odd Ratio = 1.03	Sum chi = 0.0528	p = 0.818179123431		
Mammalian L1M	58	69.517	796	784.483
Other	771	759.483	8559	8570.517
Odd Ratio = 0.81	Sum chi = 2.2674	p = 0.132119608326		
Primate L1PA *	578	579.445	8341	8339.555
Other	72	70.555	1014	1015.445
Odd Ratio = 0.98	Sum chi = 0.0355	p = 0.850513999615		
Primate L1PA	756	740.516	8341	8356.484
Other	73	88.484	1014	998.516
Odd Ratio = 1.26	Sum chi = 3.3022	p = 0.069186274740		

PolyA Signal

	sense L1s		intergenic L1s	
Case	Observed	Expected	Observed	Expected
cons *	226	212.208	2855	2868.792
Other	466	479.792	6500	6486.208
Odd Ratio = 1.10	Sum chi = 1.3885	p = 0.238656470193		
cons	255	253.161	2855	2856.839
Other	574	575.839	6500	6498.161
Odd Ratio = 1.01	Sum chi = 0.0209	p = 0.884943383074		
mut *	466	479.792	6500	6486.208
Other	226	212.208	2855	2868.792
Odd Ratio = 0.91	Sum chi = 1.3885	p = 0.238656470193		
mut	574	575.839	6500	6498.161
Other	255	253.161	2855	2856.839
Odd Ratio = 0.99	Sum chi = 0.0209	p = 0.884943383074		

Runx3 Site

	sense L1s		intergenic L1s	
Case	Observed	Expected	Observed	Expected
cons *	178	142.128	1931	1966.872
Other	498	533.872	7424	7388.128
Odd Ratio = 1.37	Sum chi = 12.292	p = 0.000454737829		
cons	195	173.061	1931	1952.939
Other	634	655.939	7424	7402.061
Odd Ratio = 1.18	Sum chi = 3.8264	p = 0.050449434147		
mut *	496	531.985	7398	7362.015
Other	180	144.015	1957	1992.985
Odd Ratio = 0.73	Sum chi = 12.251	p = 0.000464883173		
mut	632	653.66	7398	7376.34
Other	197	175.34	1957	1978.66
Odd Ratio = 0.85	Sum chi = 3.6940	p = 0.054607930118		

Odd Ratio = 1.18	Sum chi = 9.62123	p = 0.001923404840		
non canonical *	576	586.866	3995	3984.134
Other	802	791.134	5360	5370.866
Odd Ratio = 0.96	Sum chi = 0.40208	p = 0.526015444002		
non canonical	721	735.652	3995	3980.348
Other	1008	993.348	5360	5374.652
Odd Ratio = 0.96	Sum chi = 0.60180	p = 0.437891673496		

L1M/L1PA Discrimination

	antisense L1s		intergenic L1s	
Case	Observed	Expected	Observed	Expected
Mammalian L1M *	109	102.281	796	802.719
Other	1083	1089.719	8559	8552.281
Odd Ratio = 1.08	Sum chi = 0.54429	p = 0.460659245451		
Mammalian L1M	116	142.263	796	769.737
Other	1613	1586.737	8559	8585.263
Odd Ratio = 0.77	Sum chi = 6.25969	p = 0.012351575103		
Primate L1PA *	1056	1062.029	8341	8334.971
Other	136	129.971	1014	1020.029
Odd Ratio = 0.94	Sum chi = 0.35393	p = 0.551892524438		
Primate L1PA	1586	1548.519	8341	8378.481
Other	143	180.481	1014	976.519
Odd Ratio = 1.35	Sum chi = 10.2973	p = 0.001332221145		

PolyA Signal

	antisense L1s		intergenic L1s	
Case	Observed	Expected	Observed	Expected
cons *	484	412.33	2855	2926.67
Other	834	905.67	6500	6428.33
Odd Ratio = 1.32	Sum chi = 20.6829	p = 0.000005419162		
cons	575	535.048	2855	2894.952
Other	1154	1193.952	6500	6460.048
Odd Ratio = 1.13	Sum chi = 5.11857	p = 0.023671022896		
mut *	834	905.67	6500	6428.33
Other	484	412.33	2855	2926.67
Odd Ratio = 0.76	Sum chi = 20.6829	p = 0.000005419162		
mut	1154	1193.952	6500	6460.048
Other	575	535.048	2855	2894.952
Odd Ratio = 0.88	Sum chi = 5.11857	p = 0.023671022896		

Runx3 Site

	antisense L1s		intergenic L1s	
Case	Observed	Expected	Observed	Expected
cons *	333	272.114	1931	1991.886
Other	945	1005.886	7424	7363.114
Odd Ratio = 1.35	Sum chi = 19.6730	p = 0.000009188558		
cons	395	362.834	1931	1963.166
Other	1334	1366.166	7424	7391.834
Odd Ratio = 1.14	Sum chi = 4.27587	p = 0.038656944183		
mut *	941	1002.28	7398	7336.72
Other	337	275.72	1957	2018.28
Odd Ratio = 0.74	Sum chi = 19.7388	p = 0.000008877835		
mut	1330	1361.486	7398	7366.514
Other	399	367.514	1957	1988.486
Odd Ratio = 0.88	Sum chi = 4.05880	p = 0.043941471578		

Odd Ratio = 1.04	Sum chi = 0.215	p = 0.642149740988		
non canonical *	274	291.44	576	558.56
Other	445	427.56	802	819.44
Odd Ratio = 0.86	Sum chi = 2.670	p = 0.102208192861		
non canonical	319	337.045	721	702.955
Other	510	491.955	1008	1026.045
Odd Ratio = 0.87	Sum chi = 2.408	p = 0.120680848699		

L1M/L1PA Discrimination

	sense L1s		antisense L1s	
Case	Observed	Expected	Observed	Expected
Mammalian L1M *	57	58.578	109	107.422
Other	593	591.422	1083	1084.578
Odd Ratio = 0.96	Sum chi = 0.072	p = 0.788214398358		
Mammalian L1M	58	56.39	116	117.61
Other	771	772.61	1613	1611.39
Odd Ratio = 1.05	Sum chi = 0.072	p = 0.787078768795		
Primate L1PA *	578	576.602	1056	1057.398
Other	72	73.398	136	134.602
Odd Ratio = 1.03	Sum chi = 0.046	p = 0.829419710961		
Primate L1PA	756	758.998	1586	1583.002
Other	73	70.002	143	145.998
Odd Ratio = 0.93	Sum chi = 0.207	p = 0.648702665147		

PolyA Signal

	sense L1s		antisense L1s	
Case	Observed	Expected	Observed	Expected
cons *	226	244.438	484	465.562
Other	466	447.562	834	852.438
Odd Ratio = 0.84	Sum chi = 3.279	p = 0.070158029143		
cons	255	268.987	575	561.013
Other	574	560.013	1154	1167.987
Odd Ratio = 0.89	Sum chi = 1.592	p = 0.206901134452		
mut *	466	447.562	834	852.438
Other	226	244.438	484	465.562
Odd Ratio = 1.20	Sum chi = 3.279	p = 0.070158029143		
mut	574	560.013	1154	1167.987
Other	255	268.987	575	561.013
Odd Ratio = 1.12	Sum chi = 1.592	p = 0.206901134452		

Runx3 Site

	sense L1s		antisense L1s	
Case	Observed	Expected	Observed	Expected
cons *	178	176.784	333	334.216
Other	498	499.216	945	943.784
Odd Ratio = 1.01	Sum chi = 0.017	p = 0.895307771458		
cons	195	191.208	395	398.792
Other	634	637.792	1334	1330.208
Odd Ratio = 1.04	Sum chi = 0.144	p = 0.703734197582		
mut *	496	497.14	941	939.86
Other	180	178.86	337	338.14
Odd Ratio = 0.99	Sum chi = 0.015	p = 0.902160925664		
mut	632	635.848	1330	1326.152
Other	197	193.152	399	402.848
Odd Ratio = 0.96	Sum chi = 0.147	p = 0.700614526427		

mut 100T/C *	4	1.696	7	9.304
Other	1701	1703.304	9348	9345.696
Odd Ratio = 3.14	Sum chi = 2.271	p = 0.131744733120 with Yates' chi-square test		
mut 100T/C	4	2.353	7	8.647
Other	2542	2543.647	9348	9346.353
Odd Ratio = 2.10	Sum chi = 0.711	p = 0.398925653570 with Yates' chi-square test		
mut 84G/A *	2	3.237	19	17.763
Other	1703	1701.763	9336	9337.237
Odd Ratio = 0.58	Sum chi = 0.198	p = 0.655591260460 with Yates' chi-square test		
mut 84G/A	2	4.493	19	16.507
Other	2544	2541.507	9336	9338.493
Odd Ratio = 0.39	Sum chi = 1.126	p = 0.288575502907 with Yates' chi-square test		
Runx3 ASP				
	intragenic L1s		intergenic L1s	
Case	Observed	Expected	Observed	Expected
cons *	557	452.566	2340	2444.434
Other	1175	1279.434	7015	6910.566
Odd Ratio = 1.42	Sum chi = 38.66	p = 5.03603E-10		
cons	721	654.845	2340	2406.155
Other	1825	1891.155	7015	6948.845
Odd Ratio = 1.18	Sum chi = 11.44	p = 0.000716390404		
mut *	1175	1279.434	7015	6910.566
Other	557	452.566	2340	2444.434
Odd Ratio = 0.70	Sum chi = 38.66	p = 5.03603E-10		
mut	1825	1891.155	7015	6948.845
Other	721	654.845	2340	2406.155
Odd Ratio = 0.84	Sum chi = 11.44	p = 0.000716390404		
SRY Site 1				
	intragenic L1s		intergenic L1s	
Case	Observed	Expected	Observed	Expected
cons *	1036	991.589	5167	5211.411
Other	744	788.411	4188	4143.589
Odd Ratio = 1.13	Sum chi = 5.345	p = 0.020778615510		
cons	1523	1431.202	5167	5258.798
Other	1023	1114.798	4188	4096.202
Odd Ratio = 1.21	Sum chi = 17.10	p = 0.000035339976		
mut *	744	788.411	4188	4143.589
Other	1036	991.589	5167	5211.411
Odd Ratio = 0.89	Sum chi = 5.345	p = 0.020778615510		
mut	1023	1114.798	4188	4096.202
Other	1523	1431.202	5167	5258.798
Odd Ratio = 0.83	Sum chi = 17.10	p = 0.000035339976		
SRY Site 2				
	intragenic L1s		intergenic L1s	
Case	Observed	Expected	Observed	Expected
cons *	699	639.247	3298	3357.753
Other	1082	1141.753	6057	5997.247
Odd Ratio = 1.19	Sum chi = 10.37	p = 0.001280034717		
cons	957	910.279	3298	3344.721
Other	1589	1635.721	6057	6010.279
Odd Ratio = 1.11	Sum chi = 4.748	p = 0.029327066041		

mut 100T/C *	2	0.607	7	8.393
Other	674	675.393	9348	9346.607
Odd Ratio = 3.96	Sum chi = 1.4125	p = 0.234627428743 with Yates' chi-square test		
mut 100T/C	2	0.733	7	8.267
Other	827	828.267	9348	9346.733
Odd Ratio = 3.23	Sum chi = 0.8757	p = 0.349356739516 with Yates' chi-square test		
mut 84G/A *	0	1.28	19	17.72
Other	676	674.72	9336	9337.28
Odd Ratio = 0.00	Sum chi = 0.5110	p = 0.474698394414 with Yates' chi-square test		
mut 84G/A	0	1.547	19	17.453
Other	829	827.453	9336	9337.547
Odd Ratio = 0.00	Sum chi = 0.7724	p = 0.379448321865 with Yates' chi-square test		
Runx3 ASP				
	sense L1s		intergenic L1s	
Case	Observed	Expected	Observed	Expected
cons *	202	171.544	2340	2370.456
Other	475	505.456	7015	6984.544
Odd Ratio = 1.27	Sum chi = 7.7661	p = 0.005323458561		
cons	223	208.634	2340	2354.366
Other	606	620.366	7015	7000.634
Odd Ratio = 1.10	Sum chi = 1.4390	p = 0.230292084741		
mut *	475	505.456	7015	6984.544
Other	202	171.544	2340	2370.456
Odd Ratio = 0.78	Sum chi = 7.7661	p = 0.005323458561		
mut	606	620.366	7015	7000.634
Other	223	208.634	2340	2354.366
Odd Ratio = 0.91	Sum chi = 1.4390	p = 0.230292084741		
SRY Site 1				
	sense L1s		intergenic L1s	
Case	Observed	Expected	Observed	Expected
cons *	405	383.779	5167	5188.221
Other	287	308.221	4188	4166.779
Odd Ratio = 1.14	Sum chi = 2.8294	p = 0.092550216132		
cons	489	460.411	5167	5195.589
Other	340	368.589	4188	4159.411
Odd Ratio = 1.17	Sum chi = 4.3465	p = 0.037084187235		
mut *	287	308.221	4188	4166.779
Other	405	383.779	5167	5188.221
Odd Ratio = 0.87	Sum chi = 2.8294	p = 0.092550216132		
mut	340	368.589	4188	4159.411
Other	489	460.411	5167	5195.589
Odd Ratio = 0.86	Sum chi = 4.3465	p = 0.037084187235		
SRY Site 2				
	sense L1s		intergenic L1s	
Case	Observed	Expected	Observed	Expected
cons *	262	244.87	3298	3315.13
Other	429	446.13	6057	6039.87
Odd Ratio = 1.12	Sum chi = 1.9932	p = 0.157999570920		
cons	298	292.722	3298	3303.278
Other	531	536.278	6057	6051.722
Odd Ratio = 1.03	Sum chi = 0.1601	p = 0.689037295474		

mut 100T/C *	2	1.082	7	7.918
Other	1276	1276.918	9348	9347.082
Odd Ratio = 2.09	Sum chi = 0.18398	p = 0.667970693594 with Yates' chi-square test		
mut 100T/C	2	1.404	7	7.596
Other	1727	1727.596	9348	9347.404
Odd Ratio = 1.55	Sum chi = 0.00779	p = 0.929634255901 with Yates' chi-square test		
mut 84G/A *	2	2.524	19	18.476
Other	1276	1275.476	9336	9336.524
Odd Ratio = 0.77	Sum chi = 0.00026	p = 0.987122118486 with Yates' chi-square test		
mut 84G/A	2	3.276	19	17.724
Other	1727	1725.724	9336	9337.276
Odd Ratio = 0.57	Sum chi = 0.21810	p = 0.640489237371 with Yates' chi-square test		
Runx3 ASP				
	antisense L1s		intergenic L1s	
Case	Observed	Expected	Observed	Expected
cons *	409	334.268	2340	2414.732
Other	886	960.732	7015	6940.268
Odd Ratio = 1.38	Sum chi = 25.6383	p = 0.000000411977		
cons	500	443.013	2340	2396.987
Other	1229	1285.987	7015	6958.013
Odd Ratio = 1.22	Sum chi = 11.6772	p = 0.000632690011		
mut *	886	960.732	7015	6940.268
Other	409	334.268	2340	2414.732
Odd Ratio = 0.72	Sum chi = 25.6383	p = 0.000000411977		
mut	1229	1285.987	7015	6958.013
Other	500	443.013	2340	2396.987
Odd Ratio = 0.82	Sum chi = 11.6772	p = 0.000632690011		
SRY Site 1				
	antisense L1s		intergenic L1s	
Case	Observed	Expected	Observed	Expected
cons *	774	734.625	5167	5206.375
Other	546	585.375	4188	4148.625
Odd Ratio = 1.15	Sum chi = 5.43054	p = 0.019787542382		
cons	1043	968.702	5167	5241.298
Other	686	760.298	4188	4113.702
Odd Ratio = 1.23	Sum chi = 15.3543	p = 0.000089115794		
mut *	546	585.375	4188	4148.625
Other	774	734.625	5167	5206.375
Odd Ratio = 0.87	Sum chi = 5.43054	p = 0.019787542382		
mut	686	760.298	4188	4113.702
Other	1043	968.702	5167	5241.298
Odd Ratio = 0.81	Sum chi = 15.3543	p = 0.000089115794		
SRY Site 2				
	antisense L1s		intergenic L1s	
Case	Observed	Expected	Observed	Expected
cons *	515	474.305	3298	3338.695
Other	814	854.695	6057	6016.305
Odd Ratio = 1.16	Sum chi = 6.20045	p = 0.012771757327		
cons	663	617.879	3298	3343.121
Other	1066	1111.121	6057	6011.879
Odd Ratio = 1.14	Sum chi = 6.07496	p = 0.013711120473		

mut 100T/C *	2	1.384	2	2.616
Other	674	674.616	1276	1275.384
Odd Ratio = 1.89	Sum chi = 0.014	p = 0.902711463059 with Yates' chi-square test		
mut 100T/C	2	1.296	2	2.704
Other	827	827.704	1727	1726.296
Odd Ratio = 2.09	Sum chi = 0.047	p = 0.827618189896 with Yates' chi-square test		
mut 84G/A *	0	0.692	2	1.308
Other	676	675.308	1276	1276.692
Odd Ratio = 0.00	Sum chi = 0.081	p = 0.775314600190 with Yates' chi-square test		
mut 84G/A	0	0.648	2	1.352
Other	829	828.352	1727	1727.648
Odd Ratio = 0.00	Sum chi = 0.050	p = 0.822808957039 with Yates' chi-square test		
Runx3 ASP				
	sense L1s		antisense L1s	
Case	Observed	Expected	Observed	Expected
cons *	202	209.76	409	401.24
Other	475	467.24	886	893.76
Odd Ratio = 0.92	Sum chi = 0.633	p = 0.426097998979		
cons	223	234.311	500	488.689
Other	606	594.689	1229	1240.311
Odd Ratio = 0.90	Sum chi = 1.126	p = 0.288616171315		
mut *	475	467.24	886	893.76
Other	202	209.76	409	401.24
Odd Ratio = 1.09	Sum chi = 0.633	p = 0.426097998979		
mut	606	594.689	1229	1240.311
Other	223	234.311	500	488.689
Odd Ratio = 1.11	Sum chi = 1.126	p = 0.288616171315		
SRY Site 1				
	sense L1s		antisense L1s	
Case	Observed	Expected	Observed	Expected
cons *	405	405.501	774	773.499
Other	287	286.499	546	546.501
Odd Ratio = 1.00	Sum chi = 0.002	p = 0.961925847586		
cons	489	496.493	1043	1035.507
Other	340	332.507	686	693.493
Odd Ratio = 0.95	Sum chi = 0.417	p = 0.518403216884		
mut *	287	286.499	546	546.501
Other	405	405.501	774	773.499
Odd Ratio = 1.00	Sum chi = 0.002	p = 0.961925847586		
mut	340	332.507	686	693.493
Other	489	496.493	1043	1035.507
Odd Ratio = 1.06	Sum chi = 0.417	p = 0.518403216884		
SRY Site 2				
	sense L1s		antisense L1s	
Case	Observed	Expected	Observed	Expected
cons *	262	265.796	515	511.204
Other	429	425.204	814	817.796
Odd Ratio = 0.97	Sum chi = 0.133	p = 0.714445259880		
cons	298	311.442	663	649.558
Other	531	517.558	1066	1079.442
Odd Ratio = 0.90	Sum chi = 1.374	p = 0.240977378440		

mut *	1082	1141.753	6057	5997.247
Other	699	639.247	3298	3357.753
Odd Ratio = 0.84	Sum chi = 10.37	p = 0.001280034717		
mut	1589	1635.721	6057	6010.279
Other	957	910.279	3298	3344.721
Odd Ratio = 0.90	Sum chi = 4.748	p = 0.029327066041		
YY1 BoxA+BoxA				
	intragenic L1s		intergenic L1s	
Case	Observed	Expected	Observed	Expected
cons *	425	305.102	1567	1686.898
Other	1267	1386.898	7788	7668.102
Odd Ratio = 1.67	Sum chi = 67.87	p = 1.73869E-16		
cons	508	443.908	1567	1631.092
Other	2038	2102.092	7788	7723.908
Odd Ratio = 1.24	Sum chi = 14.25	p = 0.000159377393		
mut *	1267	1386.898	7788	7668.102
Other	425	305.102	1567	1686.898
Odd Ratio = 0.60	Sum chi = 67.87	p = 1.73869E-16		
mut	2038	2102.092	7788	7723.908
Other	508	443.908	1567	1631.092
Odd Ratio = 0.81	Sum chi = 14.25	p = 0.000159377393		
TF nkx-2.5				
	intragenic L1s		intergenic L1s	
Case	Observed	Expected	Observed	Expected
cons *	1063	997.249	5208	5273.751
Other	706	771.751	4147	4081.249
Odd Ratio = 1.20	Sum chi = 11.81	p = 0.000587264333		
cons	1587	1453.665	5208	5341.335
Other	959	1092.335	4147	4013.665
Odd Ratio = 1.32	Sum chi = 36.26	p = 1.72399E-9		
mut *	706	771.751	4147	4081.249
Other	1063	997.249	5208	5273.751
Odd Ratio = 0.83	Sum chi = 11.81	p = 0.000587264333		
mut	959	1092.335	4147	4013.665
Other	1587	1453.665	5208	5341.335
Odd Ratio = 0.76	Sum chi = 36.26	p = 1.72399E-9		
TF nkx-2.5B				
	intragenic L1s		intergenic L1s	
Case	Observed	Expected	Observed	Expected
cons *	183	115.576	627	694.424
Other	1374	1441.424	8728	8660.576
Odd Ratio = 1.85	Sum chi = 49.55	p = 1.92615E-12		
cons	210	179.061	627	657.939
Other	2336	2366.939	8728	8697.061
Odd Ratio = 1.25	Sum chi = 7.315	p = 0.006837131981		
mut *	1374	1441.424	8728	8660.576
Other	183	115.576	627	694.424
Odd Ratio = 0.54	Sum chi = 49.55	p = 1.92615E-12		
mut	2336	2366.939	8728	8697.061
Other	210	179.061	627	657.939
Odd Ratio = 0.80	Sum chi = 7.315	p = 0.006837131981		
REKG235				

mut *	429	446.13	6057	6039.87
Other	262	244.87	3298	3315.13
Odd Ratio = 0.89	Sum chi = 1.993	p = 0.157999570920		
mut	531	536.278	6057	6051.722
Other	298	292.722	3298	3303.278
Odd Ratio = 0.97	Sum chi = 0.160	p = 0.689037295474		
YY1 BoxA+BoxA				
	sense L1s		intergenic L1s	
Case	Observed	Expected	Observed	Expected
cons *	144	113.076	1567	1597.924
Other	518	548.924	7788	7757.076
Odd Ratio = 1.38	Sum chi = 10.92	p = 0.000950815911		
cons	156	140.256	1567	1582.744
Other	673	688.744	7788	7772.256
Odd Ratio = 1.15	Sum chi = 2.315	p = 0.128074376751		
mut *	518	548.924	7788	7757.076
Other	144	113.076	1567	1597.924
Odd Ratio = 0.72	Sum chi = 10.92	p = 0.000950815911		
mut	673	688.744	7788	7772.256
Other	156	140.256	1567	1582.744
Odd Ratio = 0.87	Sum chi = 2.315	p = 0.128074376751		
TF nkx-2.5				
	sense L1s		intergenic L1s	
Case	Observed	Expected	Observed	Expected
cons *	423	386.798	5208	5244.202
Other	267	303.202	4147	4110.798
Odd Ratio = 1.26	Sum chi = 8.279	p = 0.004009923259		
cons	513	465.702	5208	5255.298
Other	316	363.298	4147	4099.702
Odd Ratio = 1.29	Sum chi = 11.93	p = 0.000551530725		
mut *	267	303.202	4147	4110.798
Other	423	386.798	5208	5244.202
Odd Ratio = 0.79	Sum chi = 8.279	p = 0.004009923259		
mut	316	363.298	4147	4099.702
Other	513	465.702	5208	5255.298
Odd Ratio = 0.77	Sum chi = 11.93	p = 0.000551530725		
TF nkx-2.5B				
	sense L1s		intergenic L1s	
Case	Observed	Expected	Observed	Expected
cons *	66	44.569	627	648.431
Other	577	598.431	8728	8706.569
Odd Ratio = 1.59	Sum chi = 11.83	p = 0.000581625205		
cons	69	56.656	627	639.344
Other	760	772.344	8728	8715.656
Odd Ratio = 1.26	Sum chi = 3.142	p = 0.076271956978		
mut *	577	598.431	8728	8706.569
Other	66	44.569	627	648.431
Odd Ratio = 0.63	Sum chi = 11.83	p = 0.000581625205		
mut	760	772.344	8728	8715.656
Other	69	56.656	627	639.344
Odd Ratio = 0.79	Sum chi = 3.142	p = 0.076271956978		
REKG235				

mut *	814	854.695	6057	6016.305
Other	515	474.305	3298	3338.695
Odd Ratio = 0.86	Sum chi = 6.20045	p = 0.012771757327		
mut	1066	1111.121	6057	6011.879
Other	663	617.879	3298	3343.121
Odd Ratio = 0.88	Sum chi = 6.07496	p = 0.013711120473		
YY1 BoxA+BoxA				
	antisense L1s		intergenic L1s	
Case	Observed	Expected	Observed	Expected
cons *	314	226.081	1567	1654.919
Other	964	1051.919	7788	7700.081
Odd Ratio = 1.62	Sum chi = 47.2131	p = 6.36705E-12		
cons	353	299.502	1567	1620.498
Other	1376	1429.498	7788	7734.502
Odd Ratio = 1.28	Sum chi = 13.6942	p = 0.000215105875		
mut *	964	1051.919	7788	7700.081
Other	314	226.081	1567	1654.919
Odd Ratio = 0.62	Sum chi = 47.2131	p = 6.36705E-12		
mut	1376	1429.498	7788	7734.502
Other	353	299.502	1567	1620.498
Odd Ratio = 0.78	Sum chi = 13.6942	p = 0.000215105875		
TF nkx-2.5				
	antisense L1s		intergenic L1s	
Case	Observed	Expected	Observed	Expected
cons *	801	745.499	5208	5263.501
Other	524	579.501	4147	4091.499
Odd Ratio = 1.22	Sum chi = 10.7857	p = 0.001022850589		
cons	1082	981.181	5208	5308.819
Other	647	747.819	4147	4046.181
Odd Ratio = 1.33	Sum chi = 28.3783	p = 0.000000099619		
mut *	524	579.501	4147	4091.499
Other	801	745.499	5208	5263.501
Odd Ratio = 0.82	Sum chi = 10.7857	p = 0.001022850589		
mut	647	747.819	4147	4046.181
Other	1082	981.181	5208	5308.819
Odd Ratio = 0.75	Sum chi = 28.3783	p = 0.000000099619		
TF nkx-2.5B				
	antisense L1s		intergenic L1s	
Case	Observed	Expected	Observed	Expected
cons *	124	84.054	627	666.946
Other	1055	1094.946	8728	8688.054
Odd Ratio = 1.64	Sum chi = 23.0169	p = 0.000001605732		
cons	143	120.113	627	649.887
Other	1586	1608.887	8728	8705.113
Odd Ratio = 1.26	Sum chi = 5.55289	p = 0.018450182380		
mut *	1055	1094.946	8728	8688.054
Other	124	84.054	627	666.946
Odd Ratio = 0.61	Sum chi = 23.0169	p = 0.000001605732		
mut	1586	1608.887	8728	8705.113
Other	143	120.113	627	649.887
Odd Ratio = 0.80	Sum chi = 5.55289	p = 0.018450182380		
REKG235				

mut *	429	425.204	814	817.796
Other	262	265.796	515	511.204
Odd Ratio = 1.04	Sum chi = 0.13	p = 0.714445259880		
mut	531	517.558	1066	1079.442
Other	298	311.442	663	649.558
Odd Ratio = 1.11	Sum chi = 1.37	p = 0.240977378440		
YY1 BoxA+BoxA				
	sense L1s		antisense L1s	
Case	Observed	Expected	Observed	Expected
cons *	144	156.287	314	301.713
Other	518	505.713	964	976.287
Odd Ratio = 0.85	Sum chi = 1.91	p = 0.165922591012		
cons	156	164.957	353	344.043
Other	673	664.043	1376	1384.957
Odd Ratio = 0.90	Sum chi = 0.89	p = 0.343219180713		
mut *	518	505.713	964	976.287
Other	144	156.287	314	301.713
Odd Ratio = 1.17	Sum chi = 1.91	p = 0.165922591012		
mut	673	664.043	1376	1384.957
Other	156	164.957	353	344.043
Odd Ratio = 1.11	Sum chi = 0.89	p = 0.343219180713		
TF nkx-2.5				
	sense L1s		antisense L1s	
Case	Observed	Expected	Observed	Expected
cons *	423	419.136	801	804.864
Other	267	270.864	524	520.136
Odd Ratio = 1.04	Sum chi = 0.13	p = 0.710312361853		
cons	513	516.91	1082	1078.09
Other	316	312.09	647	650.91
Odd Ratio = 0.97	Sum chi = 0.11	p = 0.733179782516		
mut *	267	270.864	524	520.136
Other	423	419.136	801	804.864
Odd Ratio = 0.96	Sum chi = 0.13	p = 0.710312361853		
mut	316	312.09	647	650.91
Other	513	516.91	1082	1078.09
Odd Ratio = 1.03	Sum chi = 0.11	p = 0.733179782516		
TF nkx-2.5B				
	sense L1s		antisense L1s	
Case	Observed	Expected	Observed	Expected
cons *	66	67.053	124	122.947
Other	577	575.947	1055	1056.053
Odd Ratio = 0.97	Sum chi = 0.02	p = 0.865907760477		
cons	69	68.705	143	143.295
Other	760	760.295	1586	1585.705
Odd Ratio = 1.01	Sum chi = 0.00	p = 0.963974721979		
mut *	577	575.947	1055	1056.053
Other	66	67.053	124	122.947
Odd Ratio = 1.03	Sum chi = 0.02	p = 0.865907760477		
mut	760	760.295	1586	1585.705
Other	69	68.705	143	143.295
Odd Ratio = 0.99	Sum chi = 0.00	p = 0.963974721979		
REKG235				

	intragenic L1s		intergenic L1s	
Case	Observed	Expected	Observed	Expected
cons *	1111	1069.725	5635	5676.275
Other	652	693.275	3720	3678.725
Odd Ratio = 1.12	Sum chi = 4.813	p = 0.028241414574		
cons	1676	1564.054	5635	5746.946
Other	870	981.946	3720	3608.054
Odd Ratio = 1.27	Sum chi = 26.42	p = 0.000000273518		
mut *	645	682.334	3658	3620.666
Other	1118	1080.666	5697	5734.334
Odd Ratio = 0.90	Sum chi = 3.960	p = 0.046578638142		
mut	863	967.185	3658	3553.815
Other	1683	1578.815	5697	5801.185
Odd Ratio = 0.80	Sum chi = 23.02	p = 0.000001600542		

ARR260

	intragenic L1s		intergenic L1s	
Case	Observed	Expected	Observed	Expected
cons *	1255	1223.127	6775	6806.873
Other	426	457.873	2580	2548.127
Odd Ratio = 1.12	Sum chi = 3.597	p = 0.057877178553		
cons	2021	1881.742	6775	6914.258
Other	525	664.258	2580	2440.742
Odd Ratio = 1.47	Sum chi = 50.25	p = 1.35329E-12		
mut *	419	447.363	2518	2489.637
Other	1262	1233.637	6837	6865.363
Odd Ratio = 0.90	Sum chi = 2.890	p = 0.089097152494		
mut	518	649.496	2518	2386.504
Other	2028	1896.504	6837	6968.496
Odd Ratio = 0.69	Sum chi = 45.46	p = 1.55243E-11		

YPAKLS282

	intragenic L1s		intergenic L1s	
Case	Observed	Expected	Observed	Expected
cons *	1068	987.902	5162	5242.098
Other	695	775.098	4193	4112.902
Odd Ratio = 1.25	Sum chi = 17.55	p = 0.000027905332		
cons	1583	1442.969	5162	5302.031
Other	963	1103.031	4193	4052.969
Odd Ratio = 1.34	Sum chi = 39.90	p = 2.66915E-10		
mut *	688	764.157	4131	4054.843
Other	1075	998.843	5224	5300.157
Odd Ratio = 0.81	Sum chi = 15.92	p = 0.000066036527		
mut	956	1088.27	4131	3998.73
Other	1590	1457.73	5224	5356.27
Odd Ratio = 0.76	Sum chi = 35.71	p = 0.000000002312		

N14

	intragenic L1s		intergenic L1s	
Case	Observed	Expected	Observed	Expected
cons *	1390	1419.856	8501	8471.144
Other	178	148.144	854	883.856
Odd Ratio = 0.78	Sum chi = 7.758	p = 0.005345825237		
cons	2339	2319.019	8501	8520.981
Other	207	226.981	854	834.019
Odd Ratio = 1.14	Sum chi = 2.456	p = 0.117022504760		

	sense L1s		intergenic L1s	
Case	Observed	Expected	Observed	Expected
cons *	446	418.837	5635	5662.163
Other	246	273.163	3720	3692.837
Odd Ratio = 1.20	Sum chi = 4.792	p = 0.028577397484		
cons	546	503.147	5635	5677.853
Other	283	325.853	3720	3677.147
Odd Ratio = 1.27	Sum chi = 10.10	p = 0.001476087330		
mut *	241	268.549	3658	3630.451
Other	451	423.451	5697	5724.549
Odd Ratio = 0.83	Sum chi = 4.959	p = 0.025941871666		
mut	278	320.399	3658	3615.601
Other	551	508.601	5697	5739.399
Odd Ratio = 0.79	Sum chi = 9.955	p = 0.001603494413		

ARR260

	sense L1s		intergenic L1s	
Case	Observed	Expected	Observed	Expected
cons *	516	491.348	6775	6799.652
Other	160	184.652	2580	2555.348
Odd Ratio = 1.23	Sum chi = 4.855	p = 0.027565252516		
cons	650	604.411	6775	6820.589
Other	179	224.589	2580	2534.411
Odd Ratio = 1.38	Sum chi = 13.81	p = 0.000201474166		
mut *	155	180.136	2518	2492.864
Other	521	495.864	6837	6862.136
Odd Ratio = 0.81	Sum chi = 5.127	p = 0.023552321605		
mut	174	219.135	2518	2472.865
Other	655	609.865	6837	6882.135
Odd Ratio = 0.72	Sum chi = 13.75	p = 0.000208107225		

YPAKLS282

	sense L1s		intergenic L1s	
Case	Observed	Expected	Observed	Expected
cons *	436	383.493	5162	5214.507
Other	252	304.507	4193	4140.493
Odd Ratio = 1.41	Sum chi = 17.43	p = 0.000029693200		
cons	531	463.423	5162	5229.577
Other	298	365.577	4193	4125.423
Odd Ratio = 1.45	Sum chi = 24.32	p = 0.000000813506		
mut *	247	299.917	4131	4078.083
Other	441	388.083	5224	5276.917
Odd Ratio = 0.71	Sum chi = 17.76	p = 0.000024938778		
mut	293	360.123	4131	4063.877
Other	536	468.877	5224	5291.123
Odd Ratio = 0.69	Sum chi = 24.08	p = 0.000000924106		

N14

	sense L1s		intergenic L1s	
Case	Observed	Expected	Observed	Expected
cons *	590	589.77	8501	8501.23
Other	59	59.23	854	853.77
Odd Ratio = 1.00	Sum chi = 0.001	p = 0.974136816260		
cons	763	754.11	8501	8509.89
Other	66	74.89	854	845.11
Odd Ratio = 1.16	Sum chi = 1.262	p = 0.261098845071		

	antisense L1s		intergenic L1s	
Case	Observed	Expected	Observed	Expected
cons *	830	791.447	5635	5673.553
Other	475	513.553	3720	3681.447
Odd Ratio = 1.15	Sum chi = 5.43792	p = 0.019704062662		
cons	1139	1056.68	5635	5717.32
Other	590	672.32	3720	3637.68
Odd Ratio = 1.27	Sum chi = 19.5404	p = 0.000009849208		
mut *	473	505.718	3658	3625.282
Other	832	799.282	5697	5729.718
Odd Ratio = 0.89	Sum chi = 3.93814	p = 0.047202793471		
mut	588	662.336	3658	3583.664
Other	1141	1066.664	5697	5771.336
Odd Ratio = 0.80	Sum chi = 16.0229	p = 0.000062579971		

ARR260

	antisense L1s		intergenic L1s	
Case	Observed	Expected	Observed	Expected
cons *	953	914.746	6775	6813.254
Other	303	341.254	2580	2541.746
Odd Ratio = 1.20	Sum chi = 6.67853	p = 0.009758070046		
cons	1382	1272.415	6775	6884.585
Other	347	456.585	2580	2470.415
Odd Ratio = 1.52	Sum chi = 42.3444	p = 7.65346E-11		
mut *	301	333.679	2518	2485.321
Other	955	922.321	6837	6869.679
Odd Ratio = 0.86	Sum chi = 4.94332	p = 0.026191580660		
mut	345	446.601	2518	2416.399
Other	1384	1282.399	6837	6938.601
Odd Ratio = 0.68	Sum chi = 36.9234	p = 1.22861E-9		

YPAKLS282

	antisense L1s		intergenic L1s	
Case	Observed	Expected	Observed	Expected
cons *	785	732.436	5162	5214.564
Other	529	581.564	4193	4140.436
Odd Ratio = 1.21	Sum chi = 9.72047	p = 0.001822263507		
cons	1061	970.73	5162	5252.27
Other	668	758.27	4193	4102.73
Odd Ratio = 1.29	Sum chi = 22.6785	p = 0.000001914767		
mut *	527	573.682	4131	4084.318
Other	787	740.318	5224	5270.682
Odd Ratio = 0.85	Sum chi = 7.68922	p = 0.005555152702		
mut	666	748.287	4131	4048.713
Other	1063	980.713	5224	5306.287
Odd Ratio = 0.79	Sum chi = 18.9016	p = 0.000013763534		

N14

	antisense L1s		intergenic L1s	
Case	Observed	Expected	Observed	Expected
cons *	1063	1083.316	8501	8480.684
Other	132	111.684	854	874.316
Odd Ratio = 0.81	Sum chi = 4.59716	p = 0.032024850329		
cons	1587	1573.633	8501	8514.367
Other	142	155.367	854	840.633
Odd Ratio = 1.12	Sum chi = 1.49703	p = 0.221128217459		

	sense L1s		antisense L1s	
Case	Observed	Expected	Observed	Expected
cons *	446	442.159	830	833.841
Other	246	249.841	475	471.159
Odd Ratio = 1.04	Sum chi = 0.14	p = 0.706887751293		
cons	546	546.077	1139	1138.923
Other	283	282.923	590	590.077
Odd Ratio = 1.00	Sum chi = 0.00	p = 0.994525161492		
mut *	241	247.415	473	466.585
Other	451	444.585	832	838.415
Odd Ratio = 0.94	Sum chi = 0.39	p = 0.529063894805		
mut	278	280.654	588	585.346
Other	551	548.346	1141	1143.654
Odd Ratio = 0.98	Sum chi = 0.05	p = 0.812683116597		

ARR260

	sense L1s		antisense L1s	
Case	Observed	Expected	Observed	Expected
cons *	516	513.998	953	955.002
Other	160	162.002	303	300.998
Odd Ratio = 1.03	Sum chi = 0.05	p = 0.822968907143		
cons	650	658.533	1382	1373.467
Other	179	170.467	347	355.533
Odd Ratio = 0.91	Sum chi = 0.79	p = 0.372426096417		
mut *	155	159.553	301	296.447
Other	521	516.447	955	959.553
Odd Ratio = 0.94	Sum chi = 0.26	p = 0.609042725962		
mut	174	168.198	345	350.802
Other	655	660.802	1384	1378.198
Odd Ratio = 1.07	Sum chi = 0.37	p = 0.542218486607		

YPAKLS282

	sense L1s		antisense L1s	
Case	Observed	Expected	Observed	Expected
cons *	436	419.604	785	801.396
Other	252	268.396	529	512.604
Odd Ratio = 1.17	Sum chi = 2.50	p = 0.113697964026		
cons	531	515.937	1061	1076.063
Other	298	313.063	668	652.937
Odd Ratio = 1.12	Sum chi = 1.72	p = 0.189336088816		
mut *	247	265.99	527	508.01
Other	441	422.01	787	805.99
Odd Ratio = 0.84	Sum chi = 3.36	p = 0.066490872410		
mut	293	310.794	666	648.206
Other	536	518.206	1063	1080.794
Odd Ratio = 0.87	Sum chi = 2.41	p = 0.120470853632		

N14

	sense L1s		antisense L1s	
Case	Observed	Expected	Observed	Expected
cons *	590	581.777	1063	1071.223
Other	59	67.223	132	123.777
Odd Ratio = 1.24	Sum chi = 1.73	p = 0.188224600119		
cons	763	761.591	1587	1588.409
Other	66	67.409	142	140.591
Odd Ratio = 1.03	Sum chi = 0.04	p = 0.827609632605		

mut *	178	148	853	883
Other	1390	1420	8502	8472
Odd Ratio = 1.28	Sum chi = 7.840	p = 0.005109800633		
mut	207	226.767	853	833.233
Other	2339	2319.233	8502	8521.767
Odd Ratio = 0.88	Sum chi = 2.406	p = 0.120836305038		

E43

	intragenic L1s		intergenic L1s	
Case	Observed	Expected	Observed	Expected
cons *	1368	1401.402	8352	8318.598
Other	208	174.598	1003	1036.402
Odd Ratio = 0.79	Sum chi = 8.396	p = 0.003759304757		
cons	2313	2281.581	8352	8383.419
Other	233	264.419	1003	971.581
Odd Ratio = 1.19	Sum chi = 5.299	p = 0.021326854777		
mut *	208	174.454	1002	1035.546
Other	1368	1401.546	8353	8319.454
Odd Ratio = 1.27	Sum chi = 8.475	p = 0.003599936169		
mut	233	264.206	1002	970.794
Other	2313	2281.794	8353	8384.206
Odd Ratio = 0.84	Sum chi = 5.231	p = 0.022178859814		

Y115

	intragenic L1s		intergenic L1s	
Case	Observed	Expected	Observed	Expected
cons *	1361	1396.654	8279	8243.346
Other	224	188.346	1076	1111.654
Odd Ratio = 0.79	Sum chi = 8.957	p = 0.002763329892		
cons	2285	2259.973	8279	8304.027
Other	261	286.027	1076	1050.973
Odd Ratio = 1.14	Sum chi = 3.138	p = 0.076474843622		
mut *	224	188.201	1075	1110.799
Other	1361	1396.799	8280	8244.201
Odd Ratio = 1.27	Sum chi = 9.036	p = 0.002646478747		
mut	261	285.813	1075	1050.187
Other	2285	2260.187	8280	8304.813
Odd Ratio = 0.88	Sum chi = 3.086	p = 0.078926785168		

D145

	intragenic L1s		intergenic L1s	
Case	Observed	Expected	Observed	Expected
cons *	1339	1355.243	7975	7958.757
Other	254	237.757	1380	1396.243
Odd Ratio = 0.91	Sum chi = 1.526	p = 0.216638396345		
cons	2261	2189.804	7975	8046.196
Other	285	356.196	1380	1308.804
Odd Ratio = 1.37	Sum chi = 21.04	p = 0.000004478187		
mut *	254	237.611	1379	1395.389
Other	1339	1355.389	7976	7959.611
Odd Ratio = 1.10	Sum chi = 1.554	p = 0.212434611763		
mut	285	355.982	1379	1308.018
Other	2261	2190.018	7976	8046.982
Odd Ratio = 0.73	Sum chi = 20.93	p = 0.000004757175		

mut *	59	59.165	853	852.835
Other	590	589.835	8502	8502.165
Odd Ratio = 1.00	Sum chi = 0.000	p = 0.981420829669		
mut	66	74.809	853	844.191
Other	763	754.191	8502	8510.809
Odd Ratio = 0.86	Sum chi = 1.241	p = 0.265256398009		

E43

	sense L1s		intergenic L1s	
Case	Observed	Expected	Observed	Expected
cons *	588	579.974	8352	8360.026
Other	61	69.026	1003	994.974
Odd Ratio = 1.16	Sum chi = 1.116	p = 0.290622318452		
cons	764	742.062	8352	8373.938
Other	65	86.938	1003	981.062
Odd Ratio = 1.41	Sum chi = 6.732	p = 0.009468735920		
mut *	61	68.961	1002	994.039
Other	588	580.039	8353	8360.961
Odd Ratio = 0.86	Sum chi = 1.099	p = 0.294339270936		
mut	65	86.856	1002	980.144
Other	764	742.144	8353	8374.856
Odd Ratio = 0.71	Sum chi = 6.687	p = 0.009707124983		

Y115

	sense L1s		intergenic L1s	
Case	Observed	Expected	Observed	Expected
cons *	566	572.984	8279	8272.016
Other	82	75.016	1076	1082.984
Odd Ratio = 0.90	Sum chi = 0.786	p = 0.375221918811		
cons	739	734.085	8279	8283.915
Other	90	94.915	1076	1071.085
Odd Ratio = 1.07	Sum chi = 0.312	p = 0.575913395130		
mut *	82	74.951	1075	1082.049
Other	566	573.049	8280	8272.951
Odd Ratio = 1.12	Sum chi = 0.801	p = 0.370629253517		
mut	90	94.834	1075	1070.166
Other	739	734.166	8280	8284.834
Odd Ratio = 0.94	Sum chi = 0.302	p = 0.582110070259		

D145

	sense L1s		intergenic L1s	
Case	Observed	Expected	Observed	Expected
cons *	569	555.881	7975	7988.119
Other	82	95.119	1380	1366.881
Odd Ratio = 1.20	Sum chi = 2.266	p = 0.132197109884		
cons	740	709.42	7975	8005.58
Other	89	119.58	1380	1349.42
Odd Ratio = 1.44	Sum chi = 9.948	p = 0.001610201042		
mut *	82	95.054	1379	1365.946
Other	569	555.946	7976	7989.054
Odd Ratio = 0.83	Sum chi = 2.245	p = 0.134015642751		
mut	89	119.498	1379	1348.502
Other	740	709.502	7976	8006.498
Odd Ratio = 0.70	Sum chi = 9.900	p = 0.001652105443		

mut *	132	111.571	853	873.429
Other	1063	1083.429	8502	8481.571
Odd Ratio = 1.24	Sum chi = 4.65280	p = 0.031002846392		
mut	142	155.211	853	839.789
Other	1587	1573.789	8502	8515.211
Odd Ratio = 0.89	Sum chi = 1.46362	p = 0.226354738586		

E43

	antisense L1s		intergenic L1s	
Case	Observed	Expected	Observed	Expected
cons *	1043	1068.119	8352	8326.881
Other	157	131.881	1003	1028.119
Odd Ratio = 0.80	Sum chi = 6.06474	p = 0.013790665063		
cons	1559	1546.023	8352	8364.977
Other	170	182.977	1003	990.023
Odd Ratio = 1.10	Sum chi = 1.21950	p = 0.269457877656		
mut *	157	131.767	1002	1027.233
Other	1043	1068.233	8353	8327.767
Odd Ratio = 1.25	Sum chi = 6.12439	p = 0.013332885226		
mut	170	182.821	1002	989.179
Other	1559	1546.179	8353	8365.821
Odd Ratio = 0.91	Sum chi = 1.19125	p = 0.275075921084		

Y115

	antisense L1s		intergenic L1s	
Case	Observed	Expected	Observed	Expected
cons *	1044	1060.716	8279	8262.284
Other	157	140.284	1076	1092.716
Odd Ratio = 0.86	Sum chi = 2.54496	p = 0.110646512537		
cons	1557	1534.324	8279	8301.676
Other	172	194.676	1076	1053.324
Odd Ratio = 1.18	Sum chi = 3.52664	p = 0.060389891991		
mut *	157	140.17	1075	1091.83
Other	1044	1060.83	8280	8263.17
Odd Ratio = 1.16	Sum chi = 2.58154	p = 0.108116402629		
mut	172	194.52	1075	1052.48
Other	1557	1534.48	8280	8302.52
Odd Ratio = 0.85	Sum chi = 3.48072	p = 0.062087455824		

D145

	antisense L1s		intergenic L1s	
Case	Observed	Expected	Observed	Expected
cons *	1014	1020.451	7975	7968.549
Other	184	177.549	1380	1386.451
Odd Ratio = 0.95	Sum chi = 0.31043	p = 0.577415296974		
cons	1533	1483.159	7975	8024.841
Other	196	245.841	1380	1330.159
Odd Ratio = 1.35	Sum chi = 13.9566	p = 0.000187069531		
mut *	184	177.435	1379	1385.565
Other	1014	1020.565	7976	7969.435
Odd Ratio = 1.05	Sum chi = 0.32162	p = 0.570633802424		
mut	196	245.685	1379	1329.315
Other	1533	1483.315	7976	8025.685
Odd Ratio = 0.74	Sum chi = 13.8768	p = 0.000195190765		

mut *	59	67.223	132	123.777
Other	590	581.777	1063	1071.223
Odd Ratio = 0.81	Sum chi = 1.73	p = 0.188224600119		
mut	66	67.409	142	140.591
Other	763	761.591	1587	1588.409
Odd Ratio = 0.97	Sum chi = 0.04	p = 0.827609632605		

E43

	sense L1s		antisense L1s	
Case	Observed	Expected	Observed	Expected
cons *	588	572.482	1043	1058.518
Other	61	76.518	157	141.482
Odd Ratio = 1.45	Sum chi = 5.49	p = 0.019045445075		
cons	764	752.841	1559	1570.159
Other	65	76.159	170	158.841
Odd Ratio = 1.28	Sum chi = 2.66	p = 0.102658220161		
mut *	61	76.518	157	141.482
Other	588	572.482	1043	1058.518
Odd Ratio = 0.69	Sum chi = 5.49	p = 0.019045445075		
mut	65	76.159	170	158.841
Other	764	752.841	1559	1570.159
Odd Ratio = 0.78	Sum chi = 2.66	p = 0.102658220161		

Y115

	sense L1s		antisense L1s	
Case	Observed	Expected	Observed	Expected
cons *	566	564.24	1044	1045.76
Other	82	83.76	157	155.24
Odd Ratio = 1.04	Sum chi = 0.06	p = 0.798189596370		
cons	739	744.091	1557	1551.909
Other	90	84.909	172	177.091
Odd Ratio = 0.91	Sum chi = 0.50	p = 0.478151854185		
mut *	82	83.76	157	155.24
Other	566	564.24	1044	1045.76
Odd Ratio = 0.96	Sum chi = 0.06	p = 0.798189596370		
mut	90	84.909	172	177.091
Other	739	744.091	1557	1551.909
Odd Ratio = 1.10	Sum chi = 0.50	p = 0.478151854185		

D145

	sense L1s		antisense L1s	
Case	Observed	Expected	Observed	Expected
cons *	569	557.346	1014	1025.654
Other	82	93.654	184	172.346
Odd Ratio = 1.26	Sum chi = 2.61	p = 0.105906232466		
cons	740	736.637	1533	1536.363
Other	89	92.363	196	192.637
Odd Ratio = 1.06	Sum chi = 0.20	p = 0.651595117530		
mut *	82	93.654	184	172.346
Other	569	557.346	1014	1025.654
Odd Ratio = 0.79	Sum chi = 2.61	p = 0.105906232466		
mut	89	92.363	196	192.637
Other	740	736.637	1533	1536.363
Odd Ratio = 0.94	Sum chi = 0.20	p = 0.651595117530		

N147				
	intragenic L1s		intergenic L1s	
Case	Observed	Expected	Observed	Expected
cons *	1380	1396.743	8382	8365.257
Other	182	165.257	973	989.743
Odd Ratio = 0.88	Sum chi = 2.213	p = 0.136783336427		
cons	2344	2294.63	8382	8431.37
Other	202	251.37	973	923.63
Odd Ratio = 1.35	Sum chi = 13.68	p = 0.000216001354		
mut *	182	165.114	972	988.886
Other	1380	1396.886	8383	8366.114
Odd Ratio = 1.14	Sum chi = 2.253	p = 0.133312904372		
mut	202	251.156	972	922.844
Other	2344	2294.844	8383	8432.156
Odd Ratio = 0.74	Sum chi = 13.57	p = 0.000228798818		

N147				
	sense L1s		intergenic L1s	
Case	Observed	Expected	Observed	Expected
cons *	575	578.564	8382	8378.436
Other	71	67.436	973	976.564
Odd Ratio = 0.94	Sum chi = 0.2248	p = 0.635346814847		
cons	755	743.772	8382	8393.228
Other	74	85.228	973	961.772
Odd Ratio = 1.18	Sum chi = 1.7948	p = 0.180340792432		
mut *	71	67.371	972	975.629
Other	575	578.629	8383	8379.371
Odd Ratio = 1.06	Sum chi = 0.2333	p = 0.629086565349		
mut	74	85.147	972	960.853
Other	755	743.853	8383	8394.147
Odd Ratio = 0.85	Sum chi = 1.7703	p = 0.183334958796		

N147				
	antisense L1s		intergenic L1s	
Case	Observed	Expected	Observed	Expected
cons *	1058	1054.964	8382	8385.036
Other	119	122.036	973	969.964
Odd Ratio = 1.03	Sum chi = 0.09487	p = 0.758070783492		
cons	1600	1557.098	8382	8424.902
Other	129	171.902	973	930.098
Odd Ratio = 1.44	Sum chi = 14.0863	p = 0.000174601558		
mut *	119	121.924	972	969.076
Other	1058	1055.076	8383	8385.924
Odd Ratio = 0.97	Sum chi = 0.08808	p = 0.766620957062		
mut	129	171.746	972	929.254
Other	1600	1557.254	8383	8425.746
Odd Ratio = 0.70	Sum chi = 13.9954	p = 0.000183253568		

N147				
	sense L1s		antisense L1s	
Case	Observed	Expected	Observed	Expected
cons *	575	578.671	1058	1054.329
Other	71	67.329	119	122.671
Odd Ratio = 0.91	Sum chi = 0.346	p = 0.556293738121		
cons	755	763.211	1600	1591.789
Other	74	65.789	129	137.211
Odd Ratio = 0.82	Sum chi = 1.647	p = 0.199359544282		
mut *	71	67.329	119	122.671
Other	575	578.671	1058	1054.329
Odd Ratio = 1.10	Sum chi = 0.346	p = 0.556293738121		
mut	74	65.789	129	137.211
Other	755	763.211	1600	1591.789
Odd Ratio = 1.22	Sum chi = 1.647	p = 0.199359544282		

T192				
	intragenic L1s		intergenic L1s	
Case	Observed	Expected	Observed	Expected
cons *	1351	1375.787	8254	8229.213
Other	213	188.213	1101	1125.787
Odd Ratio = 0.85	Sum chi = 4.331	p = 0.037415333646		
cons	2301	2258.048	8254	8296.952
Other	245	287.952	1101	1058.048
Odd Ratio = 1.25	Sum chi = 9.189	p = 0.002433552083		
mut *	213	188.07	1100	1124.93
Other	1351	1375.93	8255	8230.07
Odd Ratio = 1.18	Sum chi = 4.384	p = 0.036267332409		
mut	245	287.738	1100	1057.262
Other	2301	2258.262	8255	8297.738
Odd Ratio = 0.80	Sum chi = 9.104	p = 0.002549844931		

T192				
	sense L1s		intergenic L1s	
Case	Observed	Expected	Observed	Expected
cons *	569	571.559	8254	8251.441
Other	79	76.441	1101	1103.559
Odd Ratio = 0.96	Sum chi = 0.1038	p = 0.747261771315		
cons	744	732.457	8254	8265.543
Other	85	96.543	1101	1089.457
Odd Ratio = 1.17	Sum chi = 1.7004	p = 0.192228960195		
mut *	79	76.376	1100	1102.624
Other	569	571.624	8255	8252.376
Odd Ratio = 1.04	Sum chi = 0.1092	p = 0.740998506787		
mut	85	96.462	1100	1088.538
Other	744	732.538	8255	8266.462
Odd Ratio = 0.86	Sum chi = 1.6777	p = 0.195219587657		

T192				
	antisense L1s		intergenic L1s	
Case	Observed	Expected	Observed	Expected
cons *	1038	1044.689	8254	8247.311
Other	147	140.311	1101	1107.689
Odd Ratio = 0.94	Sum chi = 0.40750	p = 0.523238605317		
cons	1567	1531.984	8254	8289.016
Other	162	197.016	1101	1065.984
Odd Ratio = 1.29	Sum chi = 8.32201	p = 0.003916744802		
mut *	147	140.199	1100	1106.801
Other	1038	1044.801	8255	8248.199
Odd Ratio = 1.06	Sum chi = 0.42161	p = 0.516133929682		
mut	162	196.86	1100	1065.14
Other	1567	1532.14	8255	8289.86
Odd Ratio = 0.78	Sum chi = 8.25372	p = 0.004066836756		

T192				
	sense L1s		antisense L1s	
Case	Observed	Expected	Observed	Expected
cons *	569	568.105	1038	1038.895
Other	79	79.895	147	146.105
Odd Ratio = 1.02	Sum chi = 0.017	p = 0.894162051194		
cons	744	748.952	1567	1562.048
Other	85	80.048	162	166.952
Odd Ratio = 0.90	Sum chi = 0.501	p = 0.478775596148		
mut *	79	79.895	147	146.105
Other	569	568.105	1038	1038.895
Odd Ratio = 0.98	Sum chi = 0.017	p = 0.894162051194		
mut	85	80.048	162	166.952
Other	744	748.952	1567	1562.048
Odd Ratio = 1.11	Sum chi = 0.501	p = 0.478775596148		

D205				
	intragenic L1s		intergenic L1s	
Case	Observed	Expected	Observed	Expected
cons *	1340	1342.265	7821	7818.735
Other	266	263.735	1534	1536.265
Odd Ratio = 0.99	Sum chi = 0.027	p = 0.868840613555		
cons	2242	2152.794	7821	7910.206
Other	304	393.206	1534	1444.794
Odd Ratio = 1.45	Sum chi = 30.44	p = 0.000000034334		
mut *	266	263.589	1533	1535.411
Other	1340	1342.411	7822	7819.589
Odd Ratio = 1.01	Sum chi = 0.030	p = 0.860409449137		
mut	304	392.992	1533	1444.008
Other	2242	2153.008	7822	7910.992
Odd Ratio = 0.69	Sum chi = 30.31	p = 0.000000036741		

D205				
	sense L1s		intergenic L1s	
Case	Observed	Expected	Observed	Expected
cons *	553	547.949	7821	7826.051
Other	102	107.051	1534	1528.949
Odd Ratio = 1.06	Sum chi = 0.3048	p = 0.580874892434		
cons	716	694.931	7821	7842.069
Other	113	134.069	1534	1512.931
Odd Ratio = 1.24	Sum chi = 4.2999	p = 0.038113529828		
mut *	102	106.986	1533	1528.014
Other	553	548.014	7822	7826.986
Odd Ratio = 0.94	Sum chi = 0.2971	p = 0.585692438619		
mut	113	133.988	1533	1512.012
Other	716	695.012	7822	7842.988
Odd Ratio = 0.81	Sum chi = 4.2688	p = 0.038816533485		

D205				
	antisense L1s		intergenic L1s	
Case	Observed	Expected	Observed	Expected
cons *	1031	1010.1	7821	7841.9
Other	174	194.9	1534	1513.1
Odd Ratio = 1.16	Sum chi = 3.01792	p = 0.082348758173		
cons	1537	1459.76	7821	7898.24
Other	192	269.24	1534	1456.76
Odd Ratio = 1.57	Sum chi = 31.0963	p = 0.000000024649		
mut *	174	194.786	1533	1512.214
Other	1031	1010.214	7822	7842.786
Odd Ratio = 0.86	Sum chi = 2.98647	p = 0.083962945295		
mut	192	269.084	1533	1455.916
Other	1537	1459.916	7822	7899.084
Odd Ratio = 0.64	Sum chi = 30.9855	p = 0.000000026087		

D205				
	sense L1s		antisense L1s	
Case	Observed	Expected	Observed	Expected
cons *	553	557.806	1031	1026.194
Other	102	97.194	174	178.806
Odd Ratio = 0.91	Sum chi = 0.430	p = 0.511587094689		
cons	716	730.155	1537	1522.845
Other	113	98.845	192	206.155
Odd Ratio = 0.79	Sum chi = 3.405	p = 0.064997282128		
mut *	102	97.194	174	178.806
Other	553	557.806	1031	1026.194
Odd Ratio = 1.09	Sum chi = 0.430	p = 0.511587094689		
mut	113	98.845	192	206.155
Other	716	730.155	1537	1522.845
Odd Ratio = 1.26	Sum chi = 3.405	p = 0.064997282128		

SDH228				
	intragenic L1s		intergenic L1s	
Case	Observed	Expected	Observed	Expected
cons *	1231	1225.765	6752	6757.235
Other	466	471.235	2603	2597.765
Odd Ratio = 1.02	Sum chi = 0.095	p = 0.757752221259		
cons	1963	1864.414	6752	6850.586
Other	583	681.586	2603	2504.414

SDH228				
	sense L1s		intergenic L1s	
Case	Observed	Expected	Observed	Expected
cons *	491	482.722	6752	6760.278
Other	177	185.278	2603	2594.722
Odd Ratio = 1.07	Sum chi = 0.5483	p = 0.458998843176		
cons	631	600.992	6752	6782.008
Other	198	228.008	2603	2572.992

SDH228				
	antisense L1s		intergenic L1s	
Case	Observed	Expected	Observed	Expected
cons *	946	922.051	6752	6775.949
Other	327	350.949	2603	2579.051
Odd Ratio = 1.12	Sum chi = 2.56345	p = 0.109359461196		
cons	1341	1262.432	6752	6830.568
Other	388	466.568	2603	2524.432

SDH228				
	sense L1s		antisense L1s	
Case	Observed	Expected	Observed	Expected
cons *	491	494.547	946	942.453
Other	177	173.453	327	330.547
Odd Ratio = 0.96	Sum chi = 0.149	p = 0.699112479027		
cons	631	639.088	1341	1332.912
Other	198	189.912	388	396.088

Odd Ratio = 1.30	Sum chi = 24.77	p = 0.000000645454		
mut *	466	471.082	2602	2596.918
Other	1231	1225.918	6753	6758.082
Odd Ratio = 0.98	Sum chi = 0.089	p = 0.764621232372		
mut	583	681.372	2602	2503.628
Other	1963	1864.628	6753	6851.372
Odd Ratio = 0.77	Sum chi = 24.66	p = 0.000000680686		
R363				
	intragenic L1s		intergenic L1s	
Case	Observed	Expected	Observed	Expected
cons *	1212	1203.504	6507	6515.496
Other	516	524.496	2848	2839.504
Odd Ratio = 1.03	Sum chi = 0.234	p = 0.628491899589		
cons	1915	1801.732	6507	6620.268
Other	631	744.268	2848	2734.732
Odd Ratio = 1.33	Sum chi = 30.98	p = 0.000000026054		
mut *	454	476.475	2602	2579.525
Other	1274	1251.525	6753	6775.475
Odd Ratio = 0.92	Sum chi = 1.734	p = 0.187894393196		
mut	566	677.735	2602	2490.265
Other	1980	1868.265	6753	6864.735
Odd Ratio = 0.74	Sum chi = 31.93	p = 0.000000016016		
mut (R/G) *	62	47.866	245	259.134
Other	1666	1680.134	9110	9095.866
Odd Ratio = 1.38	Sum chi = 5.085	p = 0.024126559042		
mut (R/G)	65	66.319	245	243.681
Other	2481	2479.681	9110	9111.319
Odd Ratio = 0.97	Sum chi = 0.034	p = 0.853165900079		
FADD700				
	intragenic L1s		intergenic L1s	
Case	Observed	Expected	Observed	Expected
cons *	1127	1106.621	6009	6029.379
Other	590	610.379	3346	3325.621
Odd Ratio = 1.06	Sum chi = 1.249	p = 0.263666036521		
cons	1751	1660.109	6009	6099.891
Other	795	885.891	3346	3255.109
Odd Ratio = 1.23	Sum chi = 18.19	p = 0.000019954393		
mut *	590	610.224	3345	3324.776
Other	1127	1106.776	6010	6030.224
Odd Ratio = 0.94	Sum chi = 1.230	p = 0.267289821388		
mut	795	885.677	3345	3254.323
Other	1751	1660.323	6010	6100.677
Odd Ratio = 0.82	Sum chi = 18.11	p = 0.000020848856		
HMKK1091				
	intragenic L1s		intergenic L1s	
Case	Observed	Expected	Observed	Expected
cons *	1073	995.002	5172	5249.998
Other	700	777.998	4183	4105.002
Odd Ratio = 1.24	Sum chi = 16.57	p = 0.000046772857		
cons	1575	1443.397	5172	5303.603
Other	971	1102.603	4183	4051.397
Odd Ratio = 1.31	Sum chi = 35.24	p = 0.000000002954		

Odd Ratio = 1.23	Sum chi = 5.930	p = 0.014883214201		
mut *	177	185.211	2602	2593.789
Other	491	482.789	6753	6761.211
Odd Ratio = 0.94	Sum chi = 0.539	p = 0.462573418957		
mut	198	227.926	2602	2572.074
Other	631	601.074	6753	6782.926
Odd Ratio = 0.81	Sum chi = 5.899	p = 0.015145897981		
R363				
	sense L1s		intergenic L1s	
Case	Observed	Expected	Observed	Expected
cons *	496	473.892	6507	6529.108
Other	183	205.108	2848	2825.892
Odd Ratio = 1.19	Sum chi = 3.662	p = 0.055666508531		
cons	626	580.642	6507	6552.358
Other	203	248.358	2848	2802.642
Odd Ratio = 1.35	Sum chi = 12.87	p = 0.000332973819		
mut *	159	186.837	2602	2574.163
Other	520	492.163	6753	6780.837
Odd Ratio = 0.79	Sum chi = 6.137	p = 0.013237461661		
mut	177	226.217	2602	2552.783
Other	652	602.783	6753	6802.217
Odd Ratio = 0.70	Sum chi = 16.03	p = 0.000062304296		
mut (R/G) *	24	18.203	245	250.797
Other	655	660.797	9110	9104.203
Odd Ratio = 1.36	Sum chi = 2.034	p = 0.153763901448		
mut (R/G)	26	22.06	245	248.94
Other	803	806.94	9110	9106.06
Odd Ratio = 1.20	Sum chi = 0.787	p = 0.375008237716		
FADD700				
	sense L1s		intergenic L1s	
Case	Observed	Expected	Observed	Expected
cons *	456	437.486	6009	6027.514
Other	223	241.514	3346	3327.486
Odd Ratio = 1.14	Sum chi = 2.362	p = 0.124274154469		
cons	561	534.812	6009	6035.188
Other	268	294.188	3346	3319.812
Odd Ratio = 1.17	Sum chi = 3.933	p = 0.047329796035		
mut *	223	241.446	3345	3326.554
Other	456	437.554	6010	6028.446
Odd Ratio = 0.88	Sum chi = 2.345	p = 0.125631979026		
mut	268	294.106	3345	3318.894
Other	561	534.894	6010	6036.106
Odd Ratio = 0.86	Sum chi = 3.909	p = 0.048008284733		
HMKK1091				
	sense L1s		intergenic L1s	
Case	Observed	Expected	Observed	Expected
cons *	417	383.913	5172	5205.087
Other	273	306.087	4183	4149.913
Odd Ratio = 1.24	Sum chi = 6.902	p = 0.008609384686		
cons	502	461.876	5172	5212.124
Other	327	367.124	4183	4142.876
Odd Ratio = 1.24	Sum chi = 8.568	p = 0.003420551027		

Odd Ratio = 1.33	Sum chi = 21.4691	p = 0.000003595224		
mut *	327	350.83	2602	2578.17
Other	946	922.17	6753	6776.83
Odd Ratio = 0.90	Sum chi = 2.53841	p = 0.111106405640		
mut	388	466.412	2602	2523.588
Other	1341	1262.588	6753	6831.412
Odd Ratio = 0.75	Sum chi = 21.3885	p = 0.000003749698		
R363				
	antisense L1s		intergenic L1s	
Case	Observed	Expected	Observed	Expected
cons *	918	903.464	6507	6521.536
Other	378	392.536	2848	2833.464
Odd Ratio = 1.06	Sum chi = 0.87907	p = 0.348456405070		
cons	1299	1217.663	6507	6588.337
Other	430	511.337	2848	2766.663
Odd Ratio = 1.32	Sum chi = 21.7666	p = 0.000003078665		
mut *	339	357.857	2602	2583.143
Other	957	938.143	6753	6771.857
Odd Ratio = 0.92	Sum chi = 1.56287	p = 0.211245474638		
mut	391	466.88	2602	2526.12
Other	1338	1262.12	6753	6828.88
Odd Ratio = 0.76	Sum chi = 20.0168	p = 0.000007676111		
mut (R/G) *	39	34.557	245	249.443
Other	1257	1261.443	9110	9105.557
Odd Ratio = 1.15	Sum chi = 0.66826	p = 0.413655719691		
mut (R/G)	39	44.301	245	239.699
Other	1690	1684.699	9110	9115.301
Odd Ratio = 0.86	Sum chi = 0.77139	p = 0.379784531753		
FADD700				
	antisense L1s		intergenic L1s	
Case	Observed	Expected	Observed	Expected
cons *	858	832.735	6009	6034.265
Other	433	458.265	3346	3320.735
Odd Ratio = 1.10	Sum chi = 2.45744	p = 0.116969077688		
cons	1198	1124.224	6009	6082.776
Other	531	604.776	3346	3272.224
Odd Ratio = 1.26	Sum chi = 16.3993	p = 0.000051303482		
mut *	433	458.144	3345	3319.856
Other	858	832.856	6010	6035.144
Odd Ratio = 0.91	Sum chi = 2.43419	p = 0.118714540149		
mut	531	604.62	3345	3271.38
Other	1198	1124.38	6010	6083.62
Odd Ratio = 0.80	Sum chi = 16.3320	p = 0.000053158720		
HMKK1091				
	antisense L1s		intergenic L1s	
Case	Observed	Expected	Observed	Expected
cons *	807	735.394	5172	5243.606
Other	505	576.606	4183	4111.394
Odd Ratio = 1.29	Sum chi = 18.0897	p = 0.000021074192		
cons	1082	975.565	5172	5278.435
Other	647	753.435	4183	4076.565
Odd Ratio = 1.35	Sum chi = 31.5727	p = 0.000000019309		

Odd Ratio = 0.92	Sum chi = 0.66	p = 0.416171701958		
mut *	177	173.453	327	330.547
Other	491	494.547	946	942.453
Odd Ratio = 1.04	Sum chi = 0.14	p = 0.699112479027		
mut	198	189.912	388	396.088
Other	631	639.088	1341	1332.912
Odd Ratio = 1.08	Sum chi = 0.66	p = 0.416171701958		
R363				
	sense L1s		antisense L1s	
Case	Observed	Expected	Observed	Expected
cons *	496	486.13	918	927.87
Other	183	192.87	378	368.13
Odd Ratio = 1.12	Sum chi = 1.07	p = 0.299777885668		
cons	626	623.857	1299	1301.143
Other	203	205.143	430	427.857
Odd Ratio = 1.02	Sum chi = 0.04	p = 0.833796981945		
mut *	159	171.211	339	326.789
Other	520	507.789	957	969.211
Odd Ratio = 0.86	Sum chi = 1.77	p = 0.182799417579		
mut	177	184.078	391	383.922
Other	652	644.922	1338	1345.078
Odd Ratio = 0.93	Sum chi = 0.51	p = 0.471867065121		
mut (R/G) *	24	21.659	39	41.341
Other	655	657.341	1257	1254.659
Odd Ratio = 1.18	Sum chi = 0.39	p = 0.528015370285		
mut (R/G)	26	21.065	39	43.935
Other	803	807.935	1690	1685.065
Odd Ratio = 1.40	Sum chi = 1.75	p = 0.185267903859		
FADD700				
	sense L1s		antisense L1s	
Case	Observed	Expected	Observed	Expected
cons *	456	452.896	858	861.104
Other	223	226.104	433	429.896
Odd Ratio = 1.03	Sum chi = 0.09	p = 0.754900529309		
cons	561	570.059	1198	1188.941
Other	268	258.941	531	540.059
Odd Ratio = 0.93	Sum chi = 0.68	p = 0.408942817818		
mut *	223	226.104	433	429.896
Other	456	452.896	858	861.104
Odd Ratio = 0.97	Sum chi = 0.09	p = 0.754900529309		
mut	268	258.941	531	540.059
Other	561	570.059	1198	1188.941
Odd Ratio = 1.08	Sum chi = 0.68	p = 0.408942817818		
HMKK1091				
	sense L1s		antisense L1s	
Case	Observed	Expected	Observed	Expected
cons *	417	421.858	807	802.142
Other	273	268.142	505	509.858
Odd Ratio = 0.96	Sum chi = 0.21	p = 0.639284566801		
cons	502	513.345	1082	1070.655
Other	327	315.655	647	658.345
Odd Ratio = 0.92	Sum chi = 0.97	p = 0.323644090648		

mut *	700	777.838	4182	4104.162
Other	1073	995.162	5173	5250.838
Odd Ratio = 0.81	Sum chi = 16.50	p = 0.000048452616		
mut	971	1102.39	4182	4050.61
Other	1575	1443.61	5173	5304.39
Odd Ratio = 0.76	Sum chi = 35.13	p = 0.000000003124		
SSS1096				
	intragenic L1s		intergenic L1s	
Case	Observed	Expected	Observed	Expected
cons *	974	876.609	4541	4638.391
Other	794	891.391	4814	4716.609
Odd Ratio = 1.30	Sum chi = 25.51	p = 0.000000438787		
cons	1401	1271.182	4541	4670.818
Other	1145	1274.818	4814	4684.182
Odd Ratio = 1.30	Sum chi = 33.68	p = 0.000000006454		
mut *	794	891.232	4813	4715.768
Other	974	876.768	4542	4639.232
Odd Ratio = 0.77	Sum chi = 25.43	p = 0.000000458146		
mut	1145	1274.604	4813	4683.396
Other	1401	1271.396	4542	4671.604
Odd Ratio = 0.77	Sum chi = 33.57	p = 0.000000006836		
I1220				
	intragenic L1s		intergenic L1s	
Case	Observed	Expected	Observed	Expected
cons *	1204	1210.782	6596	6589.218
Other	515	508.218	2759	2765.782
Odd Ratio = 0.98	Sum chi = 0.152	p = 0.696533726457		
cons	1911	1819.916	6596	6687.084
Other	635	726.084	2759	2667.916
Odd Ratio = 1.26	Sum chi = 20.33	p = 0.000006499999		
mut *	489	488.504	2658	2658.496
Other	1230	1230.496	6697	6696.504
Odd Ratio = 1.00	Sum chi = 0.000	p = 0.976976094163		
mut	609	698.915	2658	2568.085
Other	1937	1847.085	6697	6786.915
Odd Ratio = 0.79	Sum chi = 20.28	p = 0.000006676464		
mut (I/M) *	26	19.559	100	106.441
Other	1693	1699.441	9255	9248.559
Odd Ratio = 1.42	Sum chi = 2.539	p = 0.110998795609		
mut (I/M)	26	26.955	100	99.045
Other	2520	2519.045	9255	9255.955
Odd Ratio = 0.95	Sum chi = 0.043	p = 0.834715107262		
S1259				
	intragenic L1s		intergenic L1s	
Case	Observed	Expected	Observed	Expected
cons *	1292	1312.01	7405	7384.99
Other	370	349.99	1950	1970.01
Odd Ratio = 0.92	Sum chi = 1.706	p = 0.191417104893		
cons	2115	2036.629	7405	7483.371
Other	431	509.371	1950	1871.629
Odd Ratio = 1.29	Sum chi = 19.17	p = 0.000011918695		

mut *	273	306.018	4182	4148.982
Other	417	383.982	5173	5206.018
Odd Ratio = 0.81	Sum chi = 6.873	p = 0.008746853033		
mut	327	367.043	4182	4141.957
Other	502	461.957	5173	5213.043
Odd Ratio = 0.81	Sum chi = 8.534	p = 0.003485694521		
SSS1096				
	sense L1s		intergenic L1s	
Case	Observed	Expected	Observed	Expected
cons *	366	338.431	4541	4568.569
Other	327	354.569	4814	4786.431
Odd Ratio = 1.19	Sum chi = 4.714	p = 0.029906216346		
cons	434	404.976	4541	4570.024
Other	395	424.024	4814	4784.976
Odd Ratio = 1.16	Sum chi = 4.427	p = 0.035371225124		
mut *	327	354.5	4813	4785.5
Other	366	338.5	4542	4569.5
Odd Ratio = 0.84	Sum chi = 4.691	p = 0.030319719885		
mut	395	423.943	4813	4784.057
Other	434	405.057	4542	4570.943
Odd Ratio = 0.86	Sum chi = 4.402	p = 0.035889939823		
I1220				
	sense L1s		intergenic L1s	
Case	Observed	Expected	Observed	Expected
cons *	491	478.259	6596	6608.741
Other	186	198.741	2759	2746.259
Odd Ratio = 1.10	Sum chi = 1.239	p = 0.265506434524		
cons	620	587.398	6596	6628.602
Other	209	241.602	2759	2726.398
Odd Ratio = 1.24	Sum chi = 6.758	p = 0.009327991329		
mut *	177	191.317	2658	2643.683
Other	500	485.683	6697	6711.317
Odd Ratio = 0.89	Sum chi = 1.601	p = 0.205680370199		
mut	200	232.647	2658	2625.353
Other	629	596.353	6697	6729.647
Odd Ratio = 0.80	Sum chi = 6.933	p = 0.008461511072		
mut (I/M) *	9	7.356	100	101.644
Other	668	669.644	9255	9253.356
Odd Ratio = 1.25	Sum chi = 0.398	p = 0.527883045667		
mut (I/M)	9	8.873	100	100.127
Other	820	820.127	9255	9254.873
Odd Ratio = 1.02	Sum chi = 0.002	p = 0.964281850098		
S1259				
	sense L1s		intergenic L1s	
Case	Observed	Expected	Observed	Expected
cons *	535	529.914	7405	7410.086
Other	134	139.086	1950	1944.914
Odd Ratio = 1.05	Sum chi = 0.251	p = 0.615974297143		
cons	687	658.707	7405	7433.293
Other	142	170.293	1950	1921.707
Odd Ratio = 1.27	Sum chi = 6.440	p = 0.011155653128		

mut *	505	576.483	4182	4110.517
Other	807	735.517	5173	5244.483
Odd Ratio = 0.77	Sum chi = 18.0284	p = 0.000021763300		
mut	647	753.279	4182	4075.721
Other	1082	975.721	5173	5279.279
Odd Ratio = 0.74	Sum chi = 31.4817	p = 0.000000020232		
SSS1096				
	antisense L1s		intergenic L1s	
Case	Observed	Expected	Observed	Expected
cons *	733	646.947	4541	4627.053
Other	575	661.053	4814	4727.947
Odd Ratio = 1.35	Sum chi = 25.8151	p = 0.000000375921		
cons	974	860.288	4541	4654.712
Other	755	868.712	4814	4700.288
Odd Ratio = 1.37	Sum chi = 35.4436	p = 0.000000002675		
mut *	575	660.931	4813	4727.069
Other	733	647.069	4542	4627.931
Odd Ratio = 0.74	Sum chi = 25.7414	p = 0.000000390547		
mut	755	868.556	4813	4699.444
Other	974	860.444	4542	4655.556
Odd Ratio = 0.73	Sum chi = 35.3464	p = 0.000000002810		
I1220				
	antisense L1s		intergenic L1s	
Case	Observed	Expected	Observed	Expected
cons *	913	909.348	6596	6599.652
Other	376	379.652	2759	2755.348
Odd Ratio = 1.02	Sum chi = 0.05665	p = 0.811862812222		
cons	1300	1231.702	6596	6664.298
Other	429	497.298	2759	2690.702
Odd Ratio = 1.27	Sum chi = 15.6006	p = 0.000078227870		
mut *	359	365.362	2658	2651.638
Other	930	923.638	6697	6703.362
Odd Ratio = 0.97	Sum chi = 0.17590	p = 0.674917590519		
mut	412	478.891	2658	2591.109
Other	1317	1250.109	6697	6763.891
Odd Ratio = 0.79	Sum chi = 15.3109	p = 0.000091187980		
mut (I/M) *	17	14.169	100	102.831
Other	1272	1274.831	9255	9252.169
Odd Ratio = 1.24	Sum chi = 0.65081	p = 0.419820103316		
mut (I/M)	17	18.251	100	98.749
Other	1712	1710.749	9255	9256.251
Odd Ratio = 0.92	Sum chi = 0.10266	p = 0.748654288290		
S1259				
	antisense L1s		intergenic L1s	
Case	Observed	Expected	Observed	Expected
cons *	987	989.854	7405	7402.146
Other	264	261.146	1950	1952.854
Odd Ratio = 0.98	Sum chi = 0.04469	p = 0.832570920850		
cons	1438	1379.425	7405	7463.575
Other	291	349.575	1950	1891.425
Odd Ratio = 1.30	Sum chi = 14.5758	p = 0.000134629153		

mut *	273	268.142	505	509.858
Other	417	421.858	807	802.142
Odd Ratio = 1.05	Sum chi = 0.21	p = 0.639284566801		
mut	327	315.655	647	658.345
Other	502	513.345	1082	1070.655
Odd Ratio = 1.09	Sum chi = 0.97	p = 0.323644090648		
SSS1096				
	sense L1s		antisense L1s	
Case	Observed	Expected	Observed	Expected
cons *	366	380.613	733	718.387
Other	327	312.387	575	589.613
Odd Ratio = 0.88	Sum chi = 1.90	p = 0.167621794489		
cons	434	456.306	974	951.694
Other	395	372.694	755	777.306
Odd Ratio = 0.85	Sum chi = 3.58	p = 0.058180416390		
mut *	327	312.387	575	589.613
Other	366	380.613	733	718.387
Odd Ratio = 1.14	Sum chi = 1.90	p = 0.167621794489		
mut	395	372.694	755	777.306
Other	434	456.306	974	951.694
Odd Ratio = 1.17	Sum chi = 3.58	p = 0.058180416390		
I1220				
	sense L1s		antisense L1s	
Case	Observed	Expected	Observed	Expected
cons *	491	483.473	913	920.527
Other	186	193.527	376	368.473
Odd Ratio = 1.09	Sum chi = 0.62	p = 0.429107879390		
cons	620	622.236	1300	1297.764
Other	209	206.764	429	431.236
Odd Ratio = 0.98	Sum chi = 0.04	p = 0.827173110605		
mut *	177	184.574	359	351.426
Other	500	492.426	930	937.574
Odd Ratio = 0.92	Sum chi = 0.65	p = 0.419514761652		
mut	200	198.338	412	413.662
Other	629	630.662	1317	1315.338
Odd Ratio = 1.02	Sum chi = 0.02	p = 0.869261276313		
mut (I/M) *	9	8.953	17	17.047
Other	668	668.047	1272	1271.953
Odd Ratio = 1.01	Sum chi = 0.00	p = 0.984487422992		
mut (I/M)	9	8.426	17	17.574
Other	820	820.574	1712	1711.426
Odd Ratio = 1.11	Sum chi = 0.05	p = 0.809009874287		
S1259				
	sense L1s		antisense L1s	
Case	Observed	Expected	Observed	Expected
cons *	535	530.322	987	991.678
Other	134	138.678	264	259.322
Odd Ratio = 1.07	Sum chi = 0.30	p = 0.580430829434		
cons	687	688.673	1438	1436.327
Other	142	140.327	291	292.673
Odd Ratio = 0.98	Sum chi = 0.03	p = 0.850524506894		

mut *	355	340.486	1902	1916.514
Other	1307	1321.514	7453	7438.486
Odd Ratio = 1.06	Sum chi = 0.916 p = 0.338438592656			
mut	416	495.893	1902	1822.107
Other	2130	2050.107	7453	7532.893
Odd Ratio = 0.77	Sum chi = 20.33 p = 0.000006497946			
mut (S/L) *	15	9.353	47	52.647
Other	1647	1652.647	9308	9302.353
Odd Ratio = 1.80	Sum chi = 4.037 p = 0.044498153916			
mut (S/L)	15	13.264	47	48.736
Other	2531	2532.736	9308	9306.264
Odd Ratio = 1.17	Sum chi = 0.290 p = 0.589808159475			

CPG Islands

	intragenic L1s		intergenic L1s	
Case	Observed	Expected	Observed	Expected
0 Islands *	1305	1408.031	8239	8135.969
Other	314	210.969	1116	1219.031
Odd Ratio = 0.56	Sum chi = 67.86 p = 1.74657E-16			
0 Islands	2167	2226.172	8239	8179.828
Other	379	319.828	1116	1175.172
Odd Ratio = 0.77	Sum chi = 15.92 p = 0.000065800408			
1 Islands *	311	207.871	1098	1201.129
Other	1308	1411.129	8257	8153.871
Odd Ratio = 1.79	Sum chi = 68.86 p = 1.05648E-16			
1 Islands	376	315.335	1098	1158.665
Other	2170	2230.665	8257	8196.335
Odd Ratio = 1.30	Sum chi = 16.94 p = 0.000038460378			
2 Islands *	3	3.098	18	17.902
Other	1616	1615.902	9337	9337.098
Odd Ratio = 0.96	Sum chi = 0.061 p = 0.804510551904 with Yates' chi-square test			
2 Islands	3	4.493	18	16.507
Other	2543	2541.507	9337	9338.493
Odd Ratio = 0.61	Sum chi = 0.279 p = 0.597051351574 with Yates' chi-square test			

find TSDs

	intragenic L1s		intergenic L1s	
Case	Observed	Expected	Observed	Expected
no tsd *	714	736.496	3935	3912.504
Other	1047	1024.504	5420	5442.496
Odd Ratio = 0.94	Sum chi = 1.403 p = 0.236151172980			
no tsd	997	1055.111	3935	3876.889
Other	1549	1490.889	5420	5478.111
Odd Ratio = 0.89	Sum chi = 6.952 p = 0.008368324561			
have tsds *	1047	1024.504	5420	5442.496
Other	714	736.496	3935	3912.504
Odd Ratio = 1.06	Sum chi = 1.403 p = 0.236151172980			
have tsds	1549	1490.889	5420	5478.111
Other	997	1055.111	3935	3876.889
Odd Ratio = 1.13	Sum chi = 6.952 p = 0.008368324561			

mut *	130	135.615	1902	1896.385
Other	539	533.385	7453	7458.615
Odd Ratio = 0.95	Sum chi = 0.312 p = 0.576161724913			
mut	138	166.06	1902	1873.94
Other	691	662.94	7453	7481.06
Odd Ratio = 0.78	Sum chi = 6.454 p = 0.011065620977			
mut (S/L) *	4	3.404	47	47.596
Other	665	665.596	9308	9307.404
Odd Ratio = 1.19	Sum chi = 0.002 p = 0.956814024361 with Yates' chi-square test			
mut (S/L)	4	4.152	47	46.848
Other	825	824.848	9308	9308.152
Odd Ratio = 0.96	Sum chi = 0.032 p = 0.858015710579 with Yates' chi-square test			

CPG Islands

	sense L1s		intergenic L1s	
Case	Observed	Expected	Observed	Expected
0 Islands *	538	575.139	8239	8201.861
Other	118	80.861	1116	1153.139
Odd Ratio = 0.62	Sum chi = 20.81 p = 0.000005045888			
0 Islands	704	727.98	8239	8215.02
Other	125	101.02	1116	1139.98
Odd Ratio = 0.76	Sum chi = 7.056 p = 0.007897375673			
1 Islands *	118	79.682	1098	1136.318
Other	538	576.318	8257	8218.682
Odd Ratio = 1.65	Sum chi = 22.44 p = 0.000002162132			
1 Islands	125	99.555	1098	1123.445
Other	704	729.445	8257	8231.555
Odd Ratio = 1.34	Sum chi = 8.046 p = 0.004560304660			
2 Islands *	0	1.18	18	16.82
Other	656	654.82	9337	9338.18
Odd Ratio = 0.00	Sum chi = 0.419 p = 0.517106282301 with Yates' chi-square test			
2 Islands	0	1.465	18	16.535
Other	829	827.535	9337	9338.465
Odd Ratio = 0.00	Sum chi = 0.693 p = 0.404999145612 with Yates' chi-square test			

find TSDs

	sense L1s		intergenic L1s	
Case	Observed	Expected	Observed	Expected
no tsd *	284	289.807	3935	3929.193
Other	406	400.193	5420	5425.807
Odd Ratio = 0.96	Sum chi = 0.215 p = 0.642561605459			
no tsd	322	346.529	3935	3910.471
Other	507	482.471	5420	5444.529
Odd Ratio = 0.87	Sum chi = 3.247 p = 0.071521198001			
have tsds *	406	400.193	5420	5425.807
Other	284	289.807	3935	3929.193
Odd Ratio = 1.04	Sum chi = 0.215 p = 0.642561605459			
have tsds	507	482.471	5420	5444.529
Other	322	346.529	3935	3910.471
Odd Ratio = 1.14	Sum chi = 3.247 p = 0.071521198001			

mut *	253	254.187	1902	1900.813
Other	998	996.813	7453	7454.187
Odd Ratio = 0.99	Sum chi = 0.00788 p = 0.929247871081			
mut	280	340.372	1902	1841.628
Other	1449	1388.628	7453	7513.372
Odd Ratio = 0.76	Sum chi = 15.7969 p = 0.000070516629			
mut (S/L) *	11	6.841	47	51.159
Other	1240	1244.159	9308	9303.841
Odd Ratio = 1.76	Sum chi = 2.88195 p = 0.089577227575			
mut (S/L)	11	9.047	47	48.953
Other	1718	1719.953	9308	9306.047
Odd Ratio = 1.27	Sum chi = 0.50188 p = 0.478671864034			

CPG Islands

	antisense L1s		intergenic L1s	
Case	Observed	Expected	Observed	Expected
0 Islands *	999	1061.889	8239	8176.111
Other	216	153.111	1116	1178.889
Odd Ratio = 0.63	Sum chi = 33.3946 p = 0.000000007498			
0 Islands	1475	1515.293	8239	8198.707
Other	254	213.707	1116	1156.293
Odd Ratio = 0.79	Sum chi = 10.2704 p = 0.001351800979			
1 Islands *	213	150.697	1098	1160.303
Other	1002	1064.303	8257	8194.697
Odd Ratio = 1.60	Sum chi = 33.2245 p = 0.000000008193			
1 Islands	251	210.431	1098	1138.569
Other	1478	1518.569	8257	8216.431
Odd Ratio = 1.28	Sum chi = 10.5507 p = 0.001161397809			
2 Islands *	3	2.414	18	18.586
Other	1212	1212.586	9337	9336.414
Odd Ratio = 1.28	Sum chi = 0.00347 p = 0.952984391631 with Yates' chi-square test			
2 Islands	3	3.276	18	17.724
Other	1726	1725.724	9337	9337.276
Odd Ratio = 0.90	Sum chi = 0.01821 p = 0.892642496384 with Yates' chi-square test			

find TSDs

	antisense L1s		intergenic L1s	
Case	Observed	Expected	Observed	Expected
no tsd *	521	550.634	3935	3905.366
Other	798	768.366	5420	5449.634
Odd Ratio = 0.90	Sum chi = 3.12369 p = 0.077161653389			
no tsd	679	719.741	3935	3894.259
Other	1050	1009.259	5420	5460.741
Odd Ratio = 0.89	Sum chi = 4.68086 p = 0.030500406196			
have tsds *	798	768.366	5420	5449.634
Other	521	550.634	3935	3905.366
Odd Ratio = 1.11	Sum chi = 3.12369 p = 0.077161653389			
have tsds	1050	1009.259	5420	5460.741
Other	679	719.741	3935	3894.259
Odd Ratio = 1.12	Sum chi = 4.68086 p = 0.030500406196			

mut *	130	133.452	253	249.548
Other	539	535.548	998	1001.452
Odd Ratio = 0.95	Sum chi = 0.171 p = 0.679091015525			
mut	138	135.466	280	282.534
Other	691	693.534	1449	1446.466
Odd Ratio = 1.03	Sum chi = 0.08 p = 0.772177898250			
mut (S/L) *	4	5.227	11	9.773
Other	665	663.773	1240	1241.227
Odd Ratio = 0.68	Sum chi = 0.44 p = 0.504594688215			
mut (S/L)	4	4.861	11	10.139
Other	825	824.139	1718	1718.861
Odd Ratio = 0.76	Sum chi = 0.03 p = 0.841589071608 with Yates' chi-square test			

CPG Islands

	sense L1s		antisense L1s	
Case	Observed	Expected	Observed	Expected
0 Islands *	538	538.895	999	998.105
Other	118	117.105	216	216.895
Odd Ratio = 0.99	Sum chi = 0.01 p = 0.909872885326			
0 Islands	704	706.173	1475	1472.827
Other	125	122.827	254	256.173
Odd Ratio = 0.97	Sum chi = 0.06 p = 0.796083774715			
1 Islands *	118	116.053	213	214.947
Other	538	539.947	1002	1000.053
Odd Ratio = 1.03	Sum chi = 0.061 p = 0.804791344558			
1 Islands	125	121.855	251	254.145
Other	704	707.145	1478	1474.855
Odd Ratio = 1.05	Sum chi = 0.14 p = 0.707465847731			
2 Islands *	0	1.052	3	1.948
Other	656	654.948	1212	1213.052
Odd Ratio = 0.00	Sum chi = 0.44 p = 0.503975138918 with Yates' chi-square test			
2 Islands	0	0.972	3	2.028
Other	829	828.028	1726	1726.972
Odd Ratio = 0.00	Sum chi = 0.33 p = 0.559967571468 with Yates' chi-square test			

find TSDs

	sense L1s		antisense L1s	
Case	Observed	Expected	Observed	Expected
no tsd *	284	276.481	521	528.519
Other	406	413.519	798	790.481
Odd Ratio = 1.07	Sum chi = 0.51 p = 0.470964612046			
no tsd	322	324.405	679	676.595
Other	507	504.595	1050	1052.405
Odd Ratio = 0.98	Sum chi = 0.04 p = 0.835065190192			
have tsds *	406	413.519	798	790.481
Other	284	276.481	521	528.519
Odd Ratio = 0.93	Sum chi = 0.51 p = 0.470964612046			
have tsds	507	504.595	1050	1052.405
Other	322	324.405	679	676.595
Odd Ratio = 1.02	Sum chi = 0.04 p = 0.835065190192			

Table 2.2 Homoscedastic t-test of non-categorical L1 characteristics. The total number of L1 sequences containing tested L1 characteristic (N) including the mean, SD, minimum, and maximum values of such characteristic were shown for compared L1 groups.

Experiment : intragenics L1s vs intergenic L1s

Samp1 is intragenic L1s (Total 2546 L1s)
Samp2 is intergenic L1s (Total 9535 L1s)

p is two-tailed probability

line1length

Independent samples T-test.

Name	N	Mean	SD	Min	Max
Samp1	2546	7401.919	549.36	6001.0	9328.0
Samp2	9355	7368.684	601.074	6001.0	10570.0

Test statistic = 2.51832 p = 0.011804552279

ORF1 gaps

Independent samples T-test.

Name	N	Mean	SD	Min	Max
Samp1	2546	2.501	5.068	0.0	99.0
Samp2	9355	2.781	5.107	0.0	100.0

Test statistic = -2.4547 p = 0.014114268400

ORF1 frameshifts

Independent samples T-test.

Name	N	Mean	SD	Min	Max
Samp1	2546	1.672	1.587	0.0	8.0
Samp2	9355	1.855	1.662	0.0	9.0

Test statistic = -4.95428 p = 0.000000735956

ORF1 stops

Independent samples T-test.

Name	N	Mean	SD	Min	Max
Samp1	2546	3.321	3.223	0.0	25.0
Samp2	9355	3.896	3.478	0.0	28.0

Test statistic = -7.50756 p = 6.45331060225E-14

ORF2 gaps

Independent samples T-test.

Name	N	Mean	SD	Min	Max
Samp1	2546	12.407	25.55	0.0	443.0
Samp2	9354	14.306	29.875	0.0	527.0

Test statistic = -2.92812 p = 0.003416629707

ORF2 frameshifts

Independent samples T-test.

Name	N	Mean	SD	Min	Max
Samp1	2546	8.184	6.337	0.0	31.0
Samp2	9354	8.951	6.672	0.0	34.0

Test statistic = -5.2018 p = 0.000000200651

ORF2 stops

Experiment : sense L1s vs intergenic L1s

Samp1 is sense L1s (Total 829 L1s)
Samp2 is intergenic L1s (Total 9535 L1s)

p is two-tailed probability

line1length

Independent samples T-test.

Name	N	Mean	SD	Min	Max
Samp1	829	7392.657	570.269	6001.0	9178.0
Samp2	9355	7368.684	601.074	6001.0	10570.0

Test statistic = 1.10514 p = 0.269126476848

ORF1 gaps

Independent samples T-test.

Name	N	Mean	SD	Min	Max
Samp1	829	2.637	5.774	0.0	99.0
Samp2	9355	2.781	5.107	0.0	100.0

Test statistic = -0.76751 p = 0.442797059622

ORF1 frameshifts

Independent samples T-test.

Name	N	Mean	SD	Min	Max
Samp1	829	1.738	1.597	0.0	7.0
Samp2	9355	1.855	1.662	0.0	9.0

Test statistic = -1.9404 p = 0.052358408845

ORF1 stops

Independent samples T-test.

Name	N	Mean	SD	Min	Max
Samp1	829	3.329	3.333	0.0	25.0
Samp2	9355	3.896	3.478	0.0	28.0

Test statistic = -4.51186 p = 0.000006498155

ORF2 gaps

Independent samples T-test.

Name	N	Mean	SD	Min	Max
Samp1	829	13.815	28.538	0.0	443.0
Samp2	9354	14.306	29.875	0.0	527.0

Test statistic = -0.45452 p = 0.649466302868

ORF2 frameshifts

Independent samples T-test.

Name	N	Mean	SD	Min	Max
Samp1	829	8.607	6.402	0.0	29.0
Samp2	9354	8.951	6.672	0.0	34.0

Test statistic = -1.43034 p = 0.152649916820

ORF2 stops

Experiment : antisense L1s vs intergenic L1s

Samp1 is antisense L1s (Total 1729 L1s)
Samp2 is intergenic L1s (Total 9535 L1s)

p is two-tailed probability

line1length

Independent samples T-test.

Name	N	Mean	SD	Min	Max
Samp1	1729	7407.511	538.857	6002.0	9328.0
Samp2	9355	7368.684	601.074	6001.0	10570.0

Test statistic = 2.5063 p = 0.012214494184

ORF1 gaps

Independent samples T-test.

Name	N	Mean	SD	Min	Max
Samp1	1729	2.431	4.679	0.0	66.0
Samp2	9355	2.781	5.107	0.0	100.0

Test statistic = -2.64455 p = 0.008191461195

ORF1 frameshifts

Independent samples T-test.

Name	N	Mean	SD	Min	Max
Samp1	1729	1.642	1.58	0.0	8.0
Samp2	9355	1.855	1.662	0.0	9.0

Test statistic = -4.92713 p = 0.000000846520

ORF1 stops

Independent samples T-test.

Name	N	Mean	SD	Min	Max
Samp1	1729	3.312	3.181	0.0	24.0
Samp2	9355	3.896	3.478	0.0	28.0

Test statistic = -6.49491 p = 0.000000000087

ORF2 gaps

Independent samples T-test.

Name	N	Mean	SD	Min	Max
Samp1	1729	11.805	24.064	0.0	291.0
Samp2	9354	14.306	29.875	0.0	527.0

Test statistic = -3.28959 p = 0.001006451726

ORF2 frameshifts

Independent samples T-test.

Name	N	Mean	SD	Min	Max
Samp1	1729	7.98	6.288	0.0	31.0
Samp2	9354	8.951	6.672	0.0	34.0

Test statistic = -5.61265 p = 0.000000020405

ORF2 stops

Experiment : sense L1s vs antisense L1s

Samp1 is sense L1s (Total 829 L1s)
Samp2 is antisense L1s (Total 1729 L1s)

p is two-tailed probability

line1length

Independent samples T-test.

Name	N	Mean	SD	Min	Max
Samp1	829	7392.657	570.269	6001.0	9178.0
Samp2	1729	7407.511	538.857	6002.0	9328.0

Test statistic = -0.64019 p = 0.522105418719

ORF1 gaps

Independent samples T-test.

Name	N	Mean	SD	Min	Max
Samp1	829	2.637	5.774	0.0	99.0
Samp2	1729	2.431	4.679	0.0	66.0

Test statistic = 0.96116 p = 0.336564770103

ORF1 frameshifts

Independent samples T-test.

Name	N	Mean	SD	Min	Max
Samp1	829	1.738	1.597	0.0	7.0
Samp2	1729	1.642	1.58	0.0	8.0

Test statistic = 1.43674 p = 0.150914237144

ORF1 stops

Independent samples T-test.

Name	N	Mean	SD	Min	Max
Samp1	829	3.329	3.333	0.0	25.0
Samp2	1729	3.312	3.181	0.0	24.0

Test statistic = 0.12449 p = 0.900940945327

ORF2 gaps

Independent samples T-test.

Name	N	Mean	SD	Min	Max
Samp1	829	13.815	28.538	0.0	443.0
Samp2	1729	11.805	24.064	0.0	291.0

Test statistic = 1.8595 p = 0.063071552434

ORF2 frameshifts

Independent samples T-test.

Name	N	Mean	SD	Min	Max
Samp1	829	8.607	6.402	0.0	29.0
Samp2	1729	7.98	6.288	0.0	31.0

Test statistic = 2.34665 p = 0.019019165723

ORF2 stops

Independent samples T-test.

Name	N	Mean	SD	Min	Max
Samp1	2546	8.551	9.129	0.0	72.0
Samp2	9354	10.439	10.161	0.0	72.0

Test statistic = -8.48709 p = 2.36895678182E-17

find TSDs

Independent samples T-test.

Name	N	Mean	SD	Min	Max
Samp1	2546	14.231	32.826	0.0	325.0
Samp2	9355	12.503	32.742	0.0	496.0

Test statistic = 2.36057 p = 0.018263014321

G-C Content

Independent samples T-test.

Name	N	Mean	SD	Min	Max
Samp1	2546	39.955	1.998	30.05	45.06
Samp2	9355	39.509	2.18	28.53	46.92

Test statistic = 9.31291 p = 1.46085796928E-20

ORF1 %A

Independent samples T-test.

Name	N	Mean	SD	Min	Max
Samp1	2546	0.411	0.016	0.357236	0.495074
Samp2	9355	0.414	0.018	0.332792	0.509248

Test statistic = -7.81779 p = 5.82783361453E-15

ORF1 %T

Independent samples T-test.

Name	N	Mean	SD	Min	Max
Samp1	2546	0.187	0.015	0.163617	0.270982
Samp2	9355	0.191	0.018	0.154131	0.288446

Test statistic = -9.66258 p = 5.2369963219E-22

ORF2 %A

Independent samples T-test.

Name	N	Mean	SD	Min	Max
Samp1	2546	0.417	0.012	0.373984	0.481356
Samp2	9355	0.419	0.014	0.305574	0.483788

Test statistic = -8.20577 p = 2.5264128446E-16

ORF2 %T

Independent samples T-test.

Name	N	Mean	SD	Min	Max
Samp1	2546	0.209	0.01	0.190687	0.282009
Samp2	9355	0.211	0.011	0.190024	0.286972

Test statistic = -10.86897 p = 2.17864E-27

ORF1&2 %A

Independent samples T-test.

Name	N	Mean	SD	Min	Max
Samp1	2539	0.416	0.013	0.38057294631	0.470935654336

Independent samples T-test.

Name	N	Mean	SD	Min	Max
Samp1	829	8.931	9.495	0.0	57.0
Samp2	9354	10.439	10.161	0.0	72.0

Test statistic = -4.11606 p = 0.000038843796

find TSDs

Independent samples T-test.

Name	N	Mean	SD	Min	Max
Samp1	829	13.268	31.093	0.0	325.0
Samp2	9355	12.503	32.742	0.0	496.0

Test statistic = 0.64741 p = 0.517382984009

G-C Content

Independent samples T-test.

Name	N	Mean	SD	Min	Max
Samp1	829	39.922	2.024	30.48	44.24
Samp2	9355	39.509	2.18	28.53	46.92

Test statistic = 5.25651 p = 0.000000149790

ORF1 %A

Independent samples T-test.

Name	N	Mean	SD	Min	Max
Samp1	829	0.409	0.016	0.357236	0.477716
Samp2	9355	0.414	0.018	0.332792	0.509248

Test statistic = -7.15066 p = 9.22947860676E-13

ORF1 %T

Independent samples T-test.

Name	N	Mean	SD	Min	Max
Samp1	829	0.188	0.016	0.16568	0.262452
Samp2	9355	0.191	0.018	0.154131	0.288446

Test statistic = -3.65486 p = 0.000258613465

ORF2 %A

Independent samples T-test.

Name	N	Mean	SD	Min	Max
Samp1	829	0.415	0.011	0.375286	0.465581
Samp2	9355	0.419	0.014	0.305574	0.483788

Test statistic = -8.65376 p = 5.73543475768E-18

ORF2 %T

Independent samples T-test.

Name	N	Mean	SD	Min	Max
Samp1	829	0.21	0.01	0.190687	0.275814
Samp2	9355	0.211	0.011	0.190024	0.286972

Test statistic = -3.0338 p = 0.002421004540

ORF1&2 %A

Independent samples T-test.

Name	N	Mean	SD	Min	Max
Samp1	824	0.414	0.012	0.386503067485	0.460413855151

Independent samples T-test.

Name	N	Mean	SD	Min	Max
Samp1	1729	8.358	8.928	0.0	72.0
Samp2	9354	10.439	10.161	0.0	72.0

Test statistic = -7.96647 p = 1.7925416267E-15

find TSDs

Independent samples T-test.

Name	N	Mean	SD	Min	Max
Samp1	1729	14.748	33.7	0.0	292.0
Samp2	9355	12.503	32.742	0.0	496.0

Test statistic = 2.60804 p = 0.009118315005

G-C Content

Independent samples T-test.

Name	N	Mean	SD	Min	Max
Samp1	1729	39.977	1.985	30.05	45.06
Samp2	9355	39.509	2.18	28.53	46.92

Test statistic = 8.31149 p = 1.05525977744E-16

ORF1 %A

Independent samples T-test.

Name	N	Mean	SD	Min	Max
Samp1	1729	0.411	0.017	0.371705	0.495074
Samp2	9355	0.414	0.018	0.332792	0.509248

Test statistic = -5.14518 p = 0.000000271831

ORF1 %T

Independent samples T-test.

Name	N	Mean	SD	Min	Max
Samp1	1729	0.186	0.015	0.163617	0.270982
Samp2	9355	0.191	0.018	0.154131	0.288446

Test statistic = -9.75585 p = 2.1427337084E-22

ORF2 %A

Independent samples T-test.

Name	N	Mean	SD	Min	Max
Samp1	1729	0.418	0.013	0.373984	0.481356
Samp2	9355	0.419	0.014	0.305574	0.483788

Test statistic = -4.57415 p = 0.000004833502

ORF2 %T

Independent samples T-test.

Name	N	Mean	SD	Min	Max
Samp1	1729	0.208	0.009	0.192041	0.282009
Samp2	9355	0.211	0.011	0.190024	0.286972

Test statistic = -11.70575 p = 1.8E-31

ORF1&2 %A

Independent samples T-test.

Name	N	Mean	SD	Min	Max
Samp1	1727	0.417	0.013	0.38057294631	0.470935654336

Independent samples T-test.

Name	N	Mean	SD	Min	Max
Samp1	829	8.931	9.495	0.0	57.0
Samp2	1729	8.358	8.928	0.0	72.0

Test statistic = 1.4886 p = 0.136716027088

find TSDs

Independent samples T-test.

Name	N	Mean	SD	Min	Max
Samp1	829	13.268	31.093	0.0	325.0
Samp2	1729	14.748	33.7	0.0	292.0

Test statistic = -1.066 p = 0.286522948391

G-C Content

Independent samples T-test.

Name	N	Mean	SD	Min	Max
Samp1	829	39.922	2.024	30.48	44.24
Samp2	1729	39.977	1.985	30.05	45.06

Test statistic = -0.652 p = 0.514458571725

ORF1 %A

Independent samples T-test.

Name	N	Mean	SD	Min	Max
Samp1	829	0.409	0.016	0.357236	0.477716
Samp2	1729	0.411	0.017	0.371705	0.495074

Test statistic = -3.14829 p = 0.001661242642

ORF1 %T

Independent samples T-test.

Name	N	Mean	SD	Min	Max
Samp1	829	0.188	0.016	0.16568	0.262452
Samp2	1729	0.186	0.015	0.163617	0.270982

Test statistic = 3.18202 p = 0.001480060988

ORF2 %A

Independent samples T-test.

Name	N	Mean	SD	Min	Max
Samp1	829	0.415	0.011	0.375286	0.465581
Samp2	1729	0.418	0.013	0.373984	0.481356

Test statistic = -4.96522 p = 0.000000731454

ORF2 %T

Independent samples T-test.

Name	N	Mean	SD	Min	Max
Samp1	829	0.21	0.01	0.190687	0.275814
Samp2	1729	0.208	0.009	0.192041	0.282009

Test statistic = 5.06197 p = 0.000000444460

ORF1&2 %A

Independent samples T-test.

Name	N	Mean	SD	Min	Max
Samp1	824	0.414	0.012	0.386503067485	0.460413855151

Name	N	Mean	SD	Min	Max
Samp2	9292	0.419	0.014	0.381032665964	0.478818998716

Test statistic = -8.30598 p = 1.09752741953E-16

ORF1&2 %T

Independent samples T-test.

Name	N	Mean	SD	Min	Max
Samp1	2539	0.204	0.011	0.190520663087	0.276936776492
Samp2	9292	0.206	0.012	0.185328185328	0.275983973604

Test statistic = -10.42991 p = 2.3320387E-25

ORF1 CAI

Independent samples T-test.

Name	N	Mean	SD	Min	Max
Samp1	2546	0.661	0.022	0.558	0.714
Samp2	9355	0.658	0.024	0.549	0.766

Test statistic = 5.30686 p = 0.000000113531

ORF2 CAI

Independent samples T-test.

Name	N	Mean	SD	Min	Max
Samp1	2546	0.632	0.013	0.564	0.667
Samp2	9355	0.63	0.013	0.571	0.669

Test statistic = 6.57908 p = 0.000000000049

IntactnessScore

Independent samples T-test.

Name	N	Mean	SD	Min	Max
Samp1	2546	16.125	4.973	2.0	24.0
Samp2	9355	15.166	5.184	0.0	24.0

Test statistic = 8.34984 p = 7.58960682118E-17

Name	N	Mean	SD	Min	Max
Samp2	9292	0.419	0.014	0.381032665964	0.478818998716

Test statistic = -8.83919 p = 1.1246598688E-18

ORF1&2 %T

Independent samples T-test.

Name	N	Mean	SD	Min	Max
Samp1	824	0.205	0.011	0.190520663087	0.267793780004
Samp2	9292	0.206	0.012	0.185328185328	0.275983973604

Test statistic = -3.27304 p = 0.001067561434

ORF1 CAI

Independent samples T-test.

Name	N	Mean	SD	Min	Max
Samp1	829	0.66	0.023	0.565	0.713
Samp2	9355	0.658	0.024	0.549	0.766

Test statistic = 2.49378 p = 0.012654671928

ORF2 CAI

Independent samples T-test.

Name	N	Mean	SD	Min	Max
Samp1	829	0.632	0.013	0.564	0.663
Samp2	9355	0.63	0.013	0.571	0.669

Test statistic = 4.41348 p = 0.000010276886

IntactnessScore

Independent samples T-test.

Name	N	Mean	SD	Min	Max
Samp1	829	15.992	5.003	2.0	24.0
Samp2	9355	15.166	5.184	0.0	24.0

Test statistic = 4.40894 p = 0.000010494499

Name	N	Mean	SD	Min	Max
Samp2	9292	0.419	0.014	0.381032665964	0.478818998716

Test statistic = -4.61236 p = 0.000004026098

ORF1&2 %T

Independent samples T-test.

Name	N	Mean	SD	Min	Max
Samp1	1727	0.203	0.01	0.190683229814	0.276936776492
Samp2	9292	0.206	0.012	0.185328185328	0.275983973604

Test statistic = -10.99449 p = 5.6762E-28

ORF1 CAI

Independent samples T-test.

Name	N	Mean	SD	Min	Max
Samp1	1729	0.661	0.022	0.558	0.714
Samp2	9355	0.658	0.024	0.549	0.766

Test statistic = 5.03548 p = 0.000000484147

ORF2 CAI

Independent samples T-test.

Name	N	Mean	SD	Min	Max
Samp1	1729	0.632	0.012	0.572	0.667
Samp2	9355	0.63	0.013	0.571	0.669

Test statistic = 5.41858 p = 0.000000061332

IntactnessScore

Independent samples T-test.

Name	N	Mean	SD	Min	Max
Samp1	1729	16.193	4.954	2.0	24.0
Samp2	9355	15.166	5.184	0.0	24.0

Test statistic = 7.61958 p = 2.75249626371E-14

Name	N	Mean	SD	Min	Max
Samp2	1727	0.417	0.013	0.38057294631	0.470935654336

Test statistic = -5.04474 p = 0.000000486081

ORF1&2 %T

Independent samples T-test.

Name	N	Mean	SD	Min	Max
Samp1	824	0.205	0.011	0.190520663087	0.267793780004
Samp2	1727	0.203	0.01	0.190683229814	0.276936776492

Test statistic = 4.2864 p = 0.000018833760

ORF1 CAI

Independent samples T-test.

Name	N	Mean	SD	Min	Max
Samp1	829	0.66	0.023	0.565	0.713
Samp2	1729	0.661	0.022	0.558	0.714

Test statistic = -1.01912 p = 0.308242251641

ORF2 CAI

Independent samples T-test.

Name	N	Mean	SD	Min	Max
Samp1	829	0.632	0.013	0.564	0.663
Samp2	1729	0.632	0.012	0.572	0.667

Test statistic = 0.48312 p = 0.629050941629

IntactnessScore

Independent samples T-test.

Name	N	Mean	SD	Min	Max
Samp1	829	15.992	5.003	2.0	24.0
Samp2	1729	16.193	4.954	2.0	24.0

Test statistic = -0.95753 p = 0.338391666837
