# Supplementary material for: Hypomethylation of Intragenic LINE-1 Represses Transcription in Cancer Cells through AGO2
Source: PLoS One. 2011 Mar 15;6(3):e17934. doi: 10.1371/journal.pone.0017934 (PMC3057998; doi:10.1371/journal.pone.0017934)
Supplement: Table S3 — GSE records, GSM samples, type of t-test, and 2×2 contingency tables of chi-square tests corresponding to the expression analysis of genes possessing internal L1s. (PDF) [file pone.0017934.s005.pdf]

Table 3.1 shows the experiment GSE6631 head and neck squamous cell carcinoma vs normal oral epithelium. A gene either possesses LINE-1 (denoted by L1) or does not possess LINE-1 (denoted by "No L1"). The up/down regulation of a gene (denoted by "Up" and "Down") is determined by paired t-test (p-value threshold is set at 0.01). The entries in the 2x2 tables show the resulting number of genes. The p-values of 2x2 tables are obtained from Chi-square distribution. The 22 tests and 22 controls in the t-test are shown below.

	Up (p < 0.01)	Not up
L1	64	589
No L1	1094	7201

P-value:	1.54E-02		
Odd ratio:	0.72	Lower 95% CI:	0.55
		Upper 95% CI:	0.93

	Down (p < 0.01)	Not down
L1	70	583
No L1	821	7474

P-value:	5.43E-01		
Odd ratio:	1.09	Lower 95% CI:	0.84
		Upper 95% CI:	1.42

Test	Control
GSM153814 Cancer 1	GSM153813 Normal mucosa 1
GSM153816 Cancer 2	GSM153815 Normal mucosa 2
GSM153818 Cancer 3	GSM153817 Normal mucosa 3
GSM153820 Cancer 4	GSM153819 Normal mucosa 4
GSM153822 Cancer 5	GSM153821 Normal mucosa 5
GSM153824 Cancer 6	GSM153823 Normal mucosa 6
GSM153826 Cancer 7	GSM153825 Normal mucosa 7
GSM153828 Cancer 8	GSM153827 Normal mucosa 8
GSM153830 Cancer 9	GSM153829 Normal mucosa 9
GSM153832 Cancer 10	GSM153831 Normal mucosa 10
GSM153834 Cancer 11	GSM153833 Normal mucosa 11
GSM153836 Cancer 12	GSM153835 Normal mucosa 12
GSM153838 Cancer 13	GSM153837 Normal mucosa 13
GSM153840 Cancer 14	GSM153839 Normal mucosa 14
GSM153842 Cancer 15	GSM153841 Normal mucosa 15
GSM153844 Cancer 16	GSM153843 Normal mucosa 16
GSM153846 Cancer 17	GSM153845 Normal mucosa 17
GSM153848 Cancer 18	GSM153847 Normal mucosa 18
GSM153850 Cancer 19	GSM153849 Normal mucosa 19
GSM153852 Cancer 20	GSM153851 Normal mucosa 20
GSM153854 Cancer 21	GSM153853 Normal mucosa 21
GSM153856 Cancer 22	GSM153855 Normal mucosa 22

Table 3.2 shows the experiment GSE9750 cervical cancer cells vs cervical cancer epithelium. A gene either possesses LINE-1 (denoted by L1) or does not possess LINE-1 (denoted by "No L1"). The up/down regulation of a gene (denoted by "Up" and "Down") is determined by unpaired t-test (p-value threshold is set at 0.01). The entries in the 2x2 tables show the resulting number of genes. The p-values of 2x2 tables are obtained from Chi-square distribution. The 9 tests and 6 controls in the t-test are shown below.

	Up ($p < 0.01$)	Not up
L1	39	880
No L1	1152	10987

P-value:	1.39E-07
Odd ratio:	0.42
Lower 95% CI:	0.30
Upper 95% CI:	0.59

	Down ($p < 0.01$)	Not down
L1	246	673
No L1	2041	10098

P-value:	2.74E-14
Odd ratio:	1.81
Lower 95% CI:	1.55
Upper 95% CI:	2.11

Test	Control
GSM246087 Cervical cancer cell line, C4-I	GSM246488 Normal cervix epithelium_03-3505
GSM246088 Cervical cancer cell line, CaSki	GSM246489 Normal cervix epithelium_03-4216
GSM246089 Cervical cancer cell line, C-33A	GSM246490 Normal cervix epithelium_03-4508
GSM246090 Cervical cancer cell line, HT-3	GSM246491 Normal cervix epithelium_03-4986
GSM246119 Cervical cancer cell line, SiHa	GSM247162 Normal cervix epithelium_03-5419
GSM246120 Cervical cancer cell line, SW756	GSM247163 Normal cervix epithelium_03-5438
GSM246121 Cervical cancer cell line, MS751	
GSM246122 Cervical cancer cell line, ME-180	
GSM246123 Cervical cancer cell line, HeLa	

Table 3.3 shows the experiment GSE5816 lung adrenocarcinoma vs human bronchial epithelium. A gene either possesses LINE-1 (denoted by L1) or does not possess LINE-1 (denoted by "No L1"). The up/down regulation of a gene (denoted by "Up" and "Down") is determined by unpaired t-test (p-value threshold is set at 0.01). The entries in the 2x2 tables show the resulting number of genes. The p-values of 2x2 tables are obtained from Chi-square distribution. The 8 tests and 6 controls in the t-test are shown below.

	Up (p < 0.01)	Not up
L1	14	1326
No L1	176	18858

P-value:	7.68E-01		
Odd ratio:	1.13	Lower 95% CI:	0.65
		Upper 95% CI:	1.96

	Down (p < 0.01)	Not down
L1	|A| = 110	|B| = 1230
No L1	|C| = 1097	|D| = 17937

P-value:	3.12E-04		
Odd ratio:	1.46	Lower 95% CI:	1.19
		Upper 95% CI:	1.79

Test	Control
GSM134899 H2347 DMSO Control	GSM134920 HBEC4 Control treatment group (DMSO).
GSM134904 H1993 Control treatment group (DMSO)	GSM134924 HBEC2 Control treatment group (DMSO)
GSM134909 H1299 Control group treatment	GSM134931 HBEC2-Rep2 Control treatment group (DMSO).
GSM154492 H157 Control group treatment.	GSM134933 HBEC3-Rep2 Control treatment group (DMSO)
GSM134927 H460 Control treatment group (DMSO)	GSM134936 HBEC4-Rep2 Control treatment group (DMSO)
GSM134938 A549 Control treatment group (DMSO).	GSM154498 HBEC2 Control group treatment
GSM154495 H1819 Control group treatment	
GSM155191 H526 Control treatment group	

Table 3.4 shows the experiment GSE14811 liver cancer vs normal liver. A gene either possesses LINE-1 (denoted by L1) or does not possess LINE-1 (denoted by "No L1"). The up/down regulation of a gene (denoted by "Up" and "Down") is determined by paired t-test (p-value threshold is set at 0.01). The entries in the 2x2 tables show the resulting number of genes. The p-values of 2x2 tables are obtained from Chi-square distribution. The 56 tests and 56 controls in the t-test are shown below.

	Up (p < 0.01)	Not up
L1	35	255
No L1	988	4501

P-value:	1.24E-02
Odd ratio:	0.63
Lower 95% CI:	0.44
Upper 95% CI:	0.90

	Down (p < 0.01)	Not down
L1	56	234
No L1	973	4516

P-value:	5.43E-01
Odd ratio:	1.11
Lower 95% CI:	0.82
Upper 95% CI:	1.50

Test	Control
GSM370747 HCC 2	GSM370691 Adjacent Tissue 2
GSM370748 HCC 3	GSM370692 Adjacent Tissue 3
GSM370749 HCC 5	GSM370693 Adjacent Tissue 5
GSM370750 HCC 10	GSM370694 Adjacent Tissue 10
GSM370751 HCC 13	GSM370695 Adjacent Tissue 13
GSM370752 HCC 14	GSM370696 Adjacent Tissue 14
GSM370753 HCC 15	GSM370697 Adjacent Tissue 15
GSM370754 HCC 20	GSM370698 Adjacent Tissue 20
GSM370755 HCC 22	GSM370699 Adjacent Tissue 22
GSM370756 HCC 25	GSM370700 Adjacent Tissue 25
GSM370757 HCC 26	GSM370701 Adjacent Tissue 26
GSM370758 HCC 27	GSM370702 Adjacent Tissue 27
GSM370759 HCC 28	GSM370703 Adjacent Tissue 28
GSM370760 HCC 31	GSM370704 Adjacent Tissue 31
GSM370761 HCC 33	GSM370705 Adjacent Tissue 33
GSM370762 HCC 35	GSM370706 Adjacent Tissue 35
GSM370763 HCC 36	GSM370707 Adjacent Tissue 36
GSM370764 HCC 37	GSM370708 Adjacent Tissue 37
GSM370765 HCC 38	GSM370709 Adjacent Tissue 38
GSM370766 HCC 39	GSM370710 Adjacent Tissue 39
GSM370767 HCC 41	GSM370711 Adjacent Tissue 41
GSM370768 HCC 43	GSM370712 Adjacent Tissue 43
GSM370769 HCC 44	GSM370713 Adjacent Tissue 44
GSM370770 HCC 45	GSM370714 Adjacent Tissue 45
GSM370771 HCC 46	GSM370715 Adjacent Tissue 46
GSM370772 HCC 50	GSM370716 Adjacent Tissue 50
GSM370773 HCC 54	GSM370717 Adjacent Tissue 54
GSM370774 HCC 55	GSM370718 Adjacent Tissue 55
GSM370775 HCC 59	GSM370719 Adjacent Tissue 59
GSM370776 HCC 61	GSM370720 Adjacent Tissue 61
GSM370777 HCC 62	GSM370721 Adjacent Tissue 62
GSM370778 HCC 63	GSM370722 Adjacent Tissue 63
GSM370779 HCC 70	GSM370723 Adjacent Tissue 70
GSM370780 HCC 71	GSM370724 Adjacent Tissue 71
GSM370781 HCC 72	GSM370725 Adjacent Tissue 72
GSM370782 HCC 75	GSM370726 Adjacent Tissue 75
GSM370783 HCC 77	GSM370727 Adjacent Tissue 77
GSM370784 HCC 78	GSM370728 Adjacent Tissue 78
GSM370785 HCC 79	GSM370729 Adjacent Tissue 79
GSM370786 HCC 81	GSM370730 Adjacent Tissue 81
GSM370787 HCC 83	GSM370731 Adjacent Tissue 83
GSM370788 HCC 87	GSM370732 Adjacent Tissue 87
GSM370789 HCC 89	GSM370733 Adjacent Tissue 89
GSM370790 HCC 91	GSM370734 Adjacent Tissue 91
GSM370791 HCC 92	GSM370735 Adjacent Tissue 92

GSM370792 HCC 93	GSM370736 Adjacent Tissue 93
GSM370793 HCC 94	GSM370737 Adjacent Tissue 94
GSM370794 HCC 95	GSM370738 Adjacent Tissue 95
GSM370795 HCC 97	GSM370739 Adjacent Tissue 97
GSM370796 HCC 98	GSM370740 Adjacent Tissue 98
GSM370797 HCC 106	GSM370741 Adjacent Tissue 106
GSM370797 HCC 106	GSM370742 Adjacent Tissue 113
GSM370799 HCC 118	GSM370743 Adjacent Tissue 118
GSM370800 HCC 124	GSM370744 Adjacent Tissue 124
GSM370801 HCC 127	GSM370745 Adjacent Tissue 127
GSM370802 HCC 132	GSM370746 Adjacent Tissue 132

Table 3.5 shows the experiment GSE1299 Breast Cancer cells vs Normal Breast Epithelium. A gene either possesses LINE-1 (denoted by L1) or does not possess LINE-1 (denoted by "No L1"). The up/down regulation of a gene (denoted by "Up" and "Down") is determined by unpaired t-test (p-value threshold is set at 0.01). The entries in the 2x2 tables show the resulting number of genes. The p-values of 2x2 tables are obtained from Chi-square distribution. The 4 tests and 2 controls in the t-test are shown below.

	Up (p < 0.01)	Not up
L1	31	888
No L1	844	11295

P-value:	3.85E-05
Odd ratio:	0.47
Lower 95% CI:	0.32
Upper 95% CI:	0.67

	Down (p < 0.01)	Not down
L1	80	839
No L1	698	11441

P-value:	3.48E-04
Odd ratio:	1.56
Lower 95% CI:	1.23
Upper 95% CI:	1.99

**Test**	**Control**
GSM21248 Breast Cancer cells MDA-MB-436 replicate 1 133B	GSM21252 Normal Breast Epithelium Control replicate 1 133B
GSM21249 Breast Cancer cells MDA-MB-436 replicate 2 133B	GSM21253 Normal Breast Epithelium Control replicate 2 133B
GSM21250 Breast Cancer cells HCC1954 replicate 1 133B	
GSM21251 Breast Cancer cells HCC1954 replicate 2 133B	

Table 3.6 shows the experiment GSE5764 ductal and lobular breast cancer vs normal breast. A gene either possesses LINE-1 (denoted by L1) or does not possess LINE-1 (denoted by "No L1"). The up/down regulation of a gene (denoted by "Up" and "Down") is determined by paired t-test (p-value threshold is set at 0.01). The entries in the 2x2 tables show the resulting number of genes. The p-values of 2x2 tables are obtained from Chi-square distribution. The 10 tests and 10 controls in the t-test are shown below.

	Up ($p < 0.01$)	Not up
L1	12	1328
No L1	160	18874

P-value:	9.54E-01
Odd ratio:	1.07
Lower 95% CI:	0.59
Upper 95% CI:	1.92

	Down ($p < 0.01$)	Not down
L1	23	1317
No L1	169	18865

P-value:	3.88E-03
Odd ratio:	1.95
Lower 95% CI:	1.26
Upper 95% CI:	3.02

**Test**	**Control**
GSM134587 6ILC_tumor_lobular	GSM134586 6ILC_normal_lobular
GSM134591 9ILC_tumor_lobular	GSM134589 9ILC_normal_lobular
GSM134689 7ILC_tumor_lobular	GSM134688 7ILC_normal_lobular
GSM134692 8ILC_tumor_lobular	GSM134691 8ILC_normal_lobular
GSM134695 10ILC_tumor_lobular	GSM134694 10ILC_normal_lobular
GSM134698 1IDC_tumor_ductal	GSM134696 1IDC_normal_ductal
GSM134701 2IDC_tumor_ductal	GSM134699 2IDC_normal_ductal
GSM134704 3IDC_tumor ductal	GSM134702 3IDC_normal_ductal
GSM134707 4IDC_tumor ductal	GSM134705 4IDC_normal_ductal
GSM134710 5IDC_tumor ductal	GSM134708 5IDC_normal_ductal

Table 3.7 shows the experiment GSE3167 bladder carcinoma situ vs normal bladder epithelium. A gene either possesses LINE-1 (denoted by L1) or does not possess LINE-1 (denoted by "No L1"). The up/down regulation of a gene (denoted by "Up" and "Down") is determined by unpaired t-test (p-value threshold is set at 0.01). The entries in the 2x2 tables show the resulting number of genes. The p-values of 2x2 tables are obtained from Chi-square distribution. The 41 tests and 9 controls in the t-test are shown below.

	Up (p < 0.01)	Not up
L1	188	731
No L1	3687	8452

P-value:	2.84E-10
Odd ratio:	0.59
Lower 95% CI:	0.50
Upper 95% CI:	0.70

	Down (p < 0.01)	Not down
L1	382	537
No L1	3377	8762

P-value:	9.83E-19
Odd ratio:	1.85
Lower 95% CI:	1.61
Upper 95% CI:	2.12

**Test**	**Control**
GSM71028 320-7	GSM71019 Normal bladder 7-99
GSM71029 524-1	GSM71020 Normal bladder 9-99
GSM71030 602-8	GSM71021 Normal bladder 16-99
GSM71031 692-1	GSM71022 Normal bladder 18-99
GSM71032 763-1	GSM71023 Normal bladder 61-99
GSM71033 775-1	GSM71024 Normal bladder 81-99
GSM71034 797-1	GSM71025 Normal bladder 272-99
GSM71035 956-2	GSM71026 Normal bladder 277-99
GSM71036 989-1	GSM71027 Normal bladder 283-99
GSM71037 1015-1	
GSM71038 1024-1	
GSM71039 1041-1	
GSM71040 1044-1	
GSM71041 1055-1	
GSM71042 1060-1	
GSM71043 1062-2	
GSM71044 1066-1	
GSM71045 1070-1	
GSM71046 1093-1	
GSM71047 1109-1	
GSM71048 1124-1	
GSM71049 1146-1	
GSM71050 1154-1	
GSM71051 1167-1	
GSM71052 1178-1	
GSM71053 1182-1	
GSM71054 1215-1	
GSM71055 1216-1	
GSM71056 1264-1	
GSM71057 1271-1	
GSM71058 1276-1	
GSM71059 1303-1	
GSM71060 1321-1	
GSM71061 1330-1	
GSM71062 1337-1	
GSM71063 1345-2	
GSM71064 1350-1	
GSM71065 1354-1	
GSM71066 1385-1	
GSM71067 1482-1	
GSM71068 1625-1	

Table 3.8 shows the experiment GSE13911 microsatellite instable gastric cancer vs normal stomach epithelium. A gene either possesses LINE-1 (denoted by L1) or does not possess LINE-1 (denoted by "No L1"). The up/down regulation of a gene (denoted by "Up" and "Down") is determined by paired t-test (p-value threshold is set at 0.01). The entries in the 2x2 tables show the resulting number of genes. The p-values of 2x2 tables are obtained from Chi-square distribution. The 12 tests and 12 controls in the t-test are shown below.

	Up ($p < 0.01$)	Not up
L1	98	1242
No L1	2193	16841

P-value:	3.04E-06
Odd ratio:	0.61
Lower 95% CI:	0.49
Upper 95% CI:	0.75

	Down ($p < 0.01$)	Not down
L1	304	1036
No L1	2882	16152

P-value:	2.65E-13
Odd ratio:	1.64
Lower 95% CI:	1.44
Upper 95% CI:	1.88

Test	Control
GSM350411 sample105KHG - tumor	GSM350412 sample105MHG - normal
GSM350413 sample114KHG - tumor	GSM350414 sample114MHG - normal
GSM350416 sample117KHG - tumor	GSM350417 sample117MHG - normal
GSM350418 sample118KHG - tumor	GSM350419 sample118MHG - normal
GSM350420 sample123KHG - tumor	GSM350421 sample123MHG - normal
GSM350429 sample146KHG - tumor	GSM350430 sample146MHG - normal
GSM350434 sample154KHG - tumor	GSM350435 sample154MHG - normal
GSM350445 sample183KHG - tumor	GSM350446 sample183MHG - normal
GSM350447 sample186KHG - tumor	GSM350448 sample186MHG - normal
GSM350451 sample192KHG - tumor	GSM350452 sample192MHG - normal
GSM350457 sample202KHG - tumor	GSM350458 sample202MHG - normal
GSM350471 sample60KHG - tumor	GSM350472 sample60MHG - normal

Table 3.9 shows the experiment GSE6919 metastasis prostate cancer. A gene either possesses LINE-1 (denoted by L1) or does not possess LINE-1 (denoted by "No L1"). The up/down regulation of a gene (denoted by "Up" and "Down") is determined by unpaired t-test (p-value threshold is set at 0.01). The entries in the 2x2 tables show the resulting number of genes. The p-values of 2x2 tables are obtained from Chi-square distribution. The 25 tests and 18 controls in the t-test are shown below.

	Up (p < 0.01)	Not up
L1	109	544
No L1	1756	6536

P-value:	7.67E-03		
Odd ratio:	0.75	Lower 95% CI:	0.60
		Upper 95% CI:	0.92

	Down (p < 0.01)	Not down
L1	99	554
No L1	1177	7115

P-value:	5.34E-01		
Odd ratio:	1.08	Lower 95% CI:	0.86
		Upper 95% CI:	1.35

Test	Control
GSM152856 Metastatic prostate tumor samples in liver from patient FB561 FB561_5FU95Av2	GSM152804 Normal prostate tissue free of any pathological alteration from organ donor PD001 PD001U95Av2
GSM152857 Metastatic prostate tumor samples recurrent in prostate from patient FB561 FB561_4HU95Av2	GSM152805 Normal prostate tissue free of any pathological alteration from organ donor PD002 PD002U95Av2
GSM152858 Metastatic prostate tumor samples in liver from patient EB666 EB666_1G+1HU95Av2	GSM152806 Normal prostate tissue free of any pathological alteration from organ donor PD003 PD003U95Av2
GSM152859 Metastatic prostate tumor samples in kidney from patient EB667 EB667_2A+2B+2CU95Av2	GSM152807 Normal prostate tissue free of any pathological alteration from organ donor PD005 PD004U95Av2
GSM152860 Metastatic prostate tumor samples in adrenal gland from patient EB669 EB669_8BU95Av2	GSM152808 Normal prostate tissue free of any pathological alteration from organ donor PD006 PD005U95Av2
GSM152861 Metastatic prostate tumor samples in adrenal gland from patient EB669 EB669_8CU95Av2	GSM152809 Normal prostate tissue free of any pathological alteration from organ donor PD004 PD006U95Av2
GSM152862 Metastatic prostate tumor samples in retroperitoneal lymph node from patient EB667 EB667_6AU95Av2	GSM152810 Normal prostate tissue free of any pathological alteration from organ donor PD007 PD007U95Av2
GSM152863 Metastatic prostate tumor samples in left inguinal lymph node from patient EB667 EB667_5A+5BU95Av2	GSM152811 Normal prostate tissue free of any pathological alteration from organ donor PD008 PD008U95Av2
GSM152864 Metastatic prostate tumor samples in paratracheal lymph node from patient EB667 EB667_7AU95Av2	GSM152812 Normal prostate tissue free of any pathological alteration from organ donor PD009 PD009U95Av2
GSM152865 Metastatic prostate tumor samples in para tracheal lymph node from patient EB667 EB667_7B+7DU95Av2	GSM152813 Normal prostate tissue free of any pathological alteration from organ donor PD010 PD010U95Av2
GSM152866 Metastatic prostate tumor samples in lung from patient FB561 FB561_7BU95Av2	GSM152814 Normal prostate tissue free of any pathological alteration from organ donor PD011 PD011U95Av2
GSM152867 Metastatic prostate tumor samples in para tracheal lymph node from patient EB667 EB667_7CU95Av2	GSM152815 Normal prostate tissue free of any pathological alteration from organ donor PD012 PD012U95Av2
GSM152868 Metastatic prostate tumor samples in para tracheal lymph node from patient EB667 EB667_7EU95Av2	GSM152816 Normal prostate tissue free of any pathological alteration from organ donor PD013 PD013U95Av2
GSM152869 Metastatic prostate tumor samples in para tracheal lymph node from patient EB667 EB667_7FU95Av2	GSM152817 Normal prostate tissue free of any pathological alteration from organ donor PD014 PD014U95Av2
GSM152870 Metastatic prostate tumor samples in para aortic lymph node from patient EB669 EB669_4AU95Av2	GSM152818 Normal prostate tissue free of any pathological alteration from organ donor PD015 PD015U95Av2
GSM152871 Metastatic prostate tumor samples in liver from patient EB669 EB669_7A+7CU95Av2	GSM152819 Normal prostate tissue free of any pathological alteration from organ donor PD016 PD016U95Av2
GSM152872 Metastatic prostate tumor samples in liver from patient EB669 EB669_7B+7DU95Av2	GSM152820 Normal prostate tissue free of any pathological alteration from organ donor PD019 PD019U95Av2
GSM152873 Metastatic prostate tumor samples in para tracheal lymph node from patient FB561 FB561_6BU95Av2	GSM152821 Normal prostate tissue free of any pathological alteration from organ donor PD020 PD020U95Av2
GSM152874 Metastatic prostate tumor samples in para tracheal lymph node from patient FB561 FB561_6CU95Av2	
GSM152875 Metastatic prostate tumor samples in para tracheal lymph node from patient FB561 FB561_3BU95Av2	
GSM152876 Metastatic prostate tumor samples in retroperitoneal lymph node from patient FB561 FB561_2FU95Av2	
GSM152877 Metastatic prostate tumor samples in retroperitoneal lymph node from patient FB561 FB561_2GU95Av2	
GSM152878 Metastatic prostate tumor samples in para aortic lymph node from patient FB561 FB561_1BU95Av2	
GSM152879 Metastatic prostate tumor samples in para aortic lymph node from patient FB561 FB561_1CU95Av2	
GSM152880 Metastatic prostate tumor samples in liver from patient FB561 FB561_5BU95Av2	

Table 3.10 shows the experiment GSE9764 5-azadeoxycytidune treated vs untreated human mesenchymal stem cells. A gene either possesses LINE-1 (denoted by L1) or does not possess LINE-1 (denoted by "No L1"). The up/down regulation of a gene (denoted by "Up" and "Down") is determined by paired t-test (p-value threshold is set at 0.01). The entries in the 2x2 tables show the resulting number of genes. The p-values of 2x2 tables are obtained from Chi-square distribution. The 3 tests and 3 controls in the t-test are shown below.

	Up (p < 0.01)	Not up
L1	35	1305
No L1	417	18617

P-value:	3.60E-01		
Odd ratio:	1.20	Lower 95% CI:	0.84
		Upper 95% CI:	1.70

	Down (p < 0.01)	Not down
L1	38	1302
No L1	337	18697

P-value:	6.95E-03		
Odd ratio:	1.62	Lower 95% CI:	1.15
		Upper 95% CI:	2.28

Test	Control
GSM246358 5-Aza hMSCs replicate 1	GSM246361 hMSCs DMEM replicate 1
GSM246359 5-Aza hMSCs replicate 2	GSM246362 hMSCs DMEM replicate 2
GSM246360 5-Aza hMSCs replicate 3	GSM246363 hMSCs DMEM replicate 3

Table 3.11 shows the experiment GSE5816 hBEC high dose vs human bronchial epithelium (3). A gene either possesses LINE-1 (denoted by L1) or does not possess LINE-1 (denoted by "No L1"). The up/down regulation of a gene (denoted by "Up" and "Down") is determined by paired t-test (p-value threshold is set at 0.01). The entries in the 2x2 tables show the resulting number of genes. The p-values of 2x2 tables are obtained from Chi-square distribution. The 5 tests and 5 controls in the t-test are shown below.

	Up (p < 0.01)	Not up
L1	43	1297
No L1	768	18266

P-value:	1.55E-01
Odd ratio:	0.79
Lower 95% CI:	0.58
Upper 95% CI:	1.08

	Down (p < 0.01)	Not down
L1	|E| = 67	|F| = 1273
No L1	|G| = 636	|H| = 18398

P-value:	1.70E-03
Odd ratio:	1.52
Lower 95% CI:	1.18
Upper 95% CI:	1.97

Test	Control
GSM134942 HBEC4 High dose treatment group (1000 nM)	GSM134920 HBEC4 Control treatment group (DMSO)
GSM134947 HBEC2 High dose treatment group (1000 nM)	GSM134924 HBEC2 Control treatment group (DMSO)
GSM134950 HBEC2-Rep2 High dose treatment group (1000 nM)	GSM134931 HBEC2-Rep2 Control treatment group (DMSO)
GSM134951 HBEC3-Rep2 High dose treatment group (1000 nM)	GSM134933 HBEC3-Rep2 Control treatment group (DMSO)
GSM134949 HBEC4-Rep2 High dose treatment group (1000 nM)	GSM134936 HBEC4-Rep2 Control treatment group (DMSO)

Table 3.12 shows the experiment GSE4246 HEK293T Ago2sh. A gene either possesses LINE-1 (denoted by L1) or does not possess LINE-1 (denoted by "No L1"). The up/down regulation of a gene (denoted by "Up" and "Down") is determined by paired t-test (p-value threshold is set at 0.05). The entries in the 2x2 tables show the resulting number of genes. The p-values of 2x2 tables are obtained from Chi-square distribution. The 2 tests and 2 controls in the t-test are shown below.

	Up ($p < 0.05$)	Not up
L1	|A| = 125	|B| = 633
No L1	|C| = 1207	|D| = 8825

P-value:	3.98E-04		
Odd ratio:	1.44	Lower 95% CI:	1.18
		Upper 95% CI:	1.77

	Down ($p < 0.05$)	Not down
L1	57	701
No L1	1145	8887

P-value:	1.26E-03		
Odd ratio:	0.63	Lower 95% CI:	0.48
		Upper 95% CI:	0.83

**Test**	**Control**
GSM96818 Ago2 knock down replicate 1	GSM96816 shRNA ctrl replicate 1
GSM96819 Ago2 knock down replicate 2	GSM96817 shRNA ctrl replicate 2

Table 3.13 shows the experiment GSE14537 AGO2IP vs Control. A gene either possesses LINE-1 (denoted by L1) or does not possess LINE-1 (denoted by "No L1"). The increased/decreased mRNA of a gene (denoted by "Up" and "Down") is determined by paired t-test (p-value threshold is set at 0.05). The entries in the 2x2 tables show the resulting number of genes. The p-values of 2x2 tables are obtained from Chi-square distribution. The 2 tests and 2 controls in the t-test are shown below.

	Up (p < 0.05)	Not up
L1	|E| = 38	|F| = 1302
No L1	|G| = 833	|H| = 18201

P-value:	8.67E-03
Odd ratio:	0.64
Lower 95% CI:	0.46
Upper 95% CI:	0.89

	Down (p < 0.05)	Not down
L1	59	1281
No L1	835	18199

P-value:	9.67E-01
Odd ratio:	1.00
Lower 95% CI:	0.77
Upper 95% CI:	1.32

**Test (use the mean of 2 replicates)**	**Control**
GSM363764<br>MOCK transfection AGO2-IP RNA, biological replicate 1, technical replicate 1<br>GSM363765<br>MOCK transfection AGO2-IP RNA, biological replicate 1, technical replicate 2	GSM363763 MOCK transfection lysate RNA, biological replicate 1
GSM363767<br>MOCK transfection AGO2-IP RNA, biological replicate 2, technical replicate 1<br>GSM363768<br>MOCK transfection AGO2-IP RNA, biological replicate 2, technical replicate 2	GSM363766 MOCK transfection lysate RNA, biological replicate 2
