# Supplementary material for: Hypomethylation of Intragenic LINE-1 Represses Transcription in Cancer Cells through AGO2
Source: PLoS One. 2011 Mar 15;6(3):e17934. doi: 10.1371/journal.pone.0017934 (PMC3057998; doi:10.1371/journal.pone.0017934)
Supplement: Table S4 — The 2×2 contingency tables corresponding to CU-DREAM chi-square tests. (PDF) [file pone.0017934.s006.pdf]

Table 4.1 shows the experiment GSE5816 lung cancer vs. GSE5816 5-AZA treated normal brochial epithelial (hBECs). The variables A, B, C, D, E, F, G, H refer to the sets of genes in the supplementary Table 3.3 and Table 3.11. The "+" and "&" denote union and intersection operators respectively. |A| denotes the number of genes in set A. By means of set operations, entries in the 2x2 tables show the resulting number of genes. The p-values of 2x2 tables are obtained from Chi-square distribution.

<u>Table 4.1a</u> shows all genes.

<table>
<tr><td></td><td>GSE5816 lung cancers express at lower levels than hBECs at p<0.01</td><td>p ≥ 0.01</td><td>P-value:</td><td>2.67E-28</td><td></td><td></td></tr>
<tr><td>GSE5816 5-AZA treated hBECs express at lower levels than hBECs at p<0.01</td><td>|(A + C) & (E + G)| = 110</td><td>|(B + D) & (E + G)| = 593</td><td rowspan="2">Odd ratio:</td><td rowspan="2">3.14</td><td>Lower 95% CI:</td><td>2.54</td></tr>
<tr><td>p ≥ 0.01</td><td>|(A + C) & (F + H)| = 1097</td><td>|(B + D) & (F + H)| = 18574</td><td>Upper 95% CI:</td><td>3.88</td></tr>
</table>

<u>Table 4.1b</u> shows genes with intragenic L1s.

<table>
<tr><td></td><td>GSE5816 lung cancers express at lower levels than hBECs at p<0.01</td><td>p ≥ 0.01</td><td>P-value:</td><td>2.59E-04</td><td></td><td></td></tr>
<tr><td>GSE5816 5-AZA treated hBECs express at lower levels than hBECs at p<0.01</td><td>|I| = |A & E| = 14</td><td>|J| = |B & E| = 53</td><td rowspan="2">Odd ratio:</td><td rowspan="2">3.24</td><td>Lower 95% CI:</td><td>1.73</td></tr>
<tr><td>p ≥ 0.01</td><td>|K| = |A & F| = 96</td><td>|L| = |B & F| = 1177</td><td>Upper 95% CI:</td><td>6.05</td></tr>
</table>

<u>Table 4.1c</u> shows genes without intragenic L1s.

<table>
<tr><td></td><td>GSE5816 lung cancers express at lower levels than hBECs at p<0.01</td><td>p ≥ 0.01</td><td>P-value:</td><td>2.34E-24</td><td></td><td></td></tr>
<tr><td>GSE5816 5-AZA treated hBECs express at lower levels than hBECs at p<0.01</td><td>|M| = |C & G| = 96</td><td>|N| = |G & D| = 540</td><td rowspan="2">Odd ratio:</td><td rowspan="2">3.09</td><td>Lower 95% CI:</td><td>2.46</td></tr>
<tr><td>p ≥ 0.01</td><td>|O| = |C & H| = 1001</td><td>|P| = |D & H| = 17397</td><td>Upper 95% CI:</td><td>3.87</td></tr>
</table>

Table 4.2 shows the experiment GSE14537 AGO2IP bound mRNA vs GSE4246 AGO2sh increase expression. The variables A, B, C, D, E, F, G, H refer to the sets of genes in the supplementary Table 3.12 and Table 3.13. The "+" and "&" denote union and intersection operators respectively. |A| denotes the number of genes in set A. By means of set operations, entries in the 2x2 tables show the resulting number of genes. The p-values of 2x2 tables are obtained from Chi-square distribution.

Table 4.2a shows all genes.

	GSE4246 AGO2sh increase expression at p<0.05	p ≥ 0.05				
			P-value:	1.98E-03		
GSE14537 AGO2IP bound mRNA at p<0.05	|(A + C) & (E + G)| = 118	|(B + D) & (E + G)| = 1214	Odd ratio:	1.39	Lower 95% CI:	1.13
p ≥ 0.05	|(A + C) & (F + H)| = 618	|(B + D) & (F + H)| = 8840			Upper 95% CI:	1.71

Table 4.2b shows genes with intragenic L1s.

	GSE4246 AGO2sh increase expression at p<0.05	p ≥ 0.05				
			P-value:	8.99E-01		
GSE14537 AGO2IP bound mRNA at p<0.05	|A & E| = 6	|B & E| = 119	Odd ratio:	1.05	Lower 95% CI:	0.43
p ≥ 0.05	|A & F| = 29	|B & F| = 604			Upper 95% CI:	2.58

Table 4.2c shows genes without intragenic L1s.

	GSE4246 AGO2sh increase expression at p<0.05	p ≥ 0.05				
			P-value:	1.08E-03		
GSE14537 AGO2IP bound mRNA at p<0.05	|C & G| = 112	|G & D| = 1095	Odd ratio:	1.43	Lower 95% CI:	1.16
p ≥ 0.05	|C & H| = 589	|D & H| = 8236			Upper 95% CI:	1.77
