# Supplementary material for: Hypomethylation of Intragenic LINE-1 Represses Transcription in Cancer Cells through AGO2
Source: PLoS One. 2011 Mar 15;6(3):e17934. doi: 10.1371/journal.pone.0017934 (PMC3057998; doi:10.1371/journal.pone.0017934)
Supplement: Table S5 — The 2×2 contingency tables corresponding to genes possessing internal L1s which were down-regulated in both cancer and demethylated normal cells. (PDF) [file pone.0017934.s007.pdf]

Table 5 shows GSE5816 lung cancer and GSE5816 5-AZA treated normal brochial epithelial (hBECs) L1 vs. no L1. The variables I, J, K, L, M, N, O, P refer to the sets of genes in the supplementary Table 4.1b and Table 4.1c. The "+" denotes union operator. |I| denotes the number of genes in set I. By means of set operations, entries in the 2x2 tables show the resulting number of genes. The p-values of 2x2 tables are obtained from Chi-square distribution.

	Down and down	No down or no down
L1	|I| = 14	|J + K + L| = 1326
No L1	|M| = 96	|N + O + P| = 18938

P-value:	9.07E-03
Odd ratio:	2.08
Lower 95% CI:	1.14
Upper 95% CI:	3.75
