# Supplementary material for: Hypomethylation of Intragenic LINE-1 Represses Transcription in Cancer Cells through AGO2
Source: PLoS One. 2011 Mar 15;6(3):e17934. doi: 10.1371/journal.pone.0017934 (PMC3057998; doi:10.1371/journal.pone.0017934)
Supplement: Table S6 — List of GSE records and GSM samples, type of t-test, and 2×2 contingency tables of chi-square tests for the analysis of up-regulation in genes possessing internal L1s. The data were displayed for expression in demethylated lung cancer cells (Table S6.1 and S6.2), DICER1sh (Table S6.3 and S6.4), AGO1sh (Table S6.5), AGO3sh (Table S6.6), and AGO4sh (Table S6.7). (PDF) [file pone.0017934.s008.pdf]

Table 6.1 shows the experiment GSE5816 5-azadeoxycytidune treated vs untreated lung cancer cell. A gene either possesses LINE-1 (denoted by L1) or does not possess LINE-1 (denoted by "No L1"). The up/down regulation of a gene (denoted by "Up" and "Down") is determined by paired t-test (p-value threshold is set at 0.01). The entries in the 2x2 tables show the resulting number of genes. The p-values of 2x2 tables are obtained from Chi-square distribution. The 8 tests and 8 controls in the t-test are shown below.

	Up (p<0.01)	Not up
L1	30	1,312
No L1	482	18,550

P-value:	5.02E-01		
Odds Ratio:	0.88	Lower 95% CI:	0.61
		Upper 95% CI:	1.28

Test	Control
GSM134902 H2347 High dose 5-aza treatment group (1000 nM)	GSM134899 H2347 DMSO Control
GSM134908 H1993 High dose 5-aza treatment group (1000 nM)	GSM134904 H1993 Control treatment group (DMSO)
GSM134944 H1299 High dose treatment group (1000 nM)	GSM134909 H1299 Control group treatment
GSM134946 H460 High dose treatment group (1000 nM)	GSM134927 H460 Control treatment group (DMSO)
GSM134945 A549 High dose treatment group (1000 nM)	GSM134938 A549 Control treatment group (DMSO)
GSM154494 H157 High dose treatment group	GSM154492 H157 Control group treatment
GSM154497 H1819 High dose treatment group (1000 nM)	GSM154495 H1819 Control group treatment
GSM155193 H526 High dose treatment group (1000 nM)	GSM155191 H526 Control treatment group

Table 6.2 shows the experiment GSE5816 5-azadeoxycytidune treated vs untreated adenocarcinoma lung cancer cell. A gene either possesses LINE-1 (denoted by L1) or does not possess LINE-1 (denoted by "No L1"). The up/down regulation of a gene (denoted by "Up" and "Down") is determined by paired t-test (p-value threshold is set at 0.01). The entries in the 2x2 tables show the resulting number of genes. The p-values of 2x2 tables are obtained from Chi-square distribution. The 3 tests and 3 controls in the t-test are shown below.

	Up (p<0.01)	Not up
L1	7	1,335
No L1	183	18,849

P-value:	1.05E-01		
Odds Ratio:	0.54	Lower 95% CI:	0.25
		Upper 95% CI:	1.15

Test	Control
GSM134902 H2347 High dose 5-aza treatment group (1000 nM)	GSM134899 H2347 DMSO Control
GSM134945 A549 High dose treatment group (1000 nM)	GSM134938 A549 Control treatment group (DMSO)
GSM154497 H1819 High dose treatment group (1000 nM)	GSM154495 H1819 Control group treatment

Table 6.3 shows the experiment GSE4246 HEK293T DICER1sh 2 days. A gene either possesses LINE-1 (denoted by L1) or does not possess LINE-1 (denoted by "No L1"). The up/down regulation of a gene (denoted by "Up" and "Down") is determined by paired t-test (p-value threshold is set at 0.05). The entries in the 2x2 tables show the resulting number of genes. The p-values of 2x2 tables are obtained from Chi-square distribution. The 2 tests and 2 controls in the t-test are shown below.

	Up (p<0.05)	Not up
L1	62	689
No L1	778	9,258

P-value:	6.19E-01		
Odds Ratio:	1.07	Lower 95% CI:	0.82
		Upper 95% CI:	1.40

Test	Control
(GSM96842 + GSM96843) / 2<br>Dicer knockdown clone #2-2 replicate 1 2 days induced<br>Dicer knockdown clone #2-2 replicate 2 2 days induced	(GSM96840 + GSM96841) / 2<br>empty vector ctrl replicate 1 2 days induced<br>empty vector ctrl replicate 2 2 days induced
(GSM96844 + GSM96845) / 2<br>Dicer knockdown clone #2b2 replicate 1 2 days induced<br>Dicer knockdown clone #2b2 replicate 2 2 days induced	(GSM96840 + GSM96841) / 2<br>empty vector ctrl replicate 1 2 days induced<br>empty vector ctrl replicate 2 2 days induced

Table 6.4 shows the experiment GSE4246 HEK293T DICER1sh 6 days. A gene either possesses LINE-1 (denoted by L1) or does not possess LINE-1 (denoted by "No L1"). The up/down regulation of a gene (denoted by "Up" and "Down") is determined by paired t-test (p-value threshold is set at 0.05). The entries in the 2x2 tables show the resulting number of genes. The p-values of 2x2 tables are obtained from Chi-square distribution. The 2 tests and 2 controls in the t-test are shown below.

	Up (p<0.05)	Not up
L1	63	705
No L1	684	9,500

P-value:	1.15E-01		
Odds Ratio:	1.24	Lower 95% CI:	0.95
		Upper 95% CI:	1.62

Test	Control
(GSM96848 + GSM96849) / 2<br>Dicer knockdown clone #2-2 replicate 1 6 days induced<br>Dicer knockdown clone #2-2 replicate 2 6 days induced	(GSM96846 + GSM96847) / 2<br>empty vector ctrl replicate 1 6 days induced<br>empty vector ctrl replicate 2 6 days induced
(GSM96850 + GSM96851) / 2<br>Dicer knockdown clone #2b2 replicate 1 6 days induced<br>Dicer knockdown clone #2b2 replicate 2 6 days induced	(GSM96846 + GSM96847) / 2<br>empty vector ctrl replicate 1 6 days induced<br>empty vector ctrl replicate 2 6 days induced

Table 6.5 shows the experiment GSE4246 HEK293T AGO1sh. A gene either possesses LINE-1 (denoted by L1) or does not possess LINE-1 (denoted by "No L1"). The up/down regulation of a gene (denoted by "Up" and "Down") is determined by paired t-test (p-value threshold is set at 0.05). The entries in the 2x2 tables show the resulting number of genes. The p-values of 2x2 tables are obtained from Chi-square distribution. The 2 tests and 2 controls in the t-test are shown below.

	Up (p<0.05)	Not up
L1	78	595
No L1	1,007	8,281

P-value:	5.48E-01		
Odds Ratio:	1.08	Lower 95% CI:	0.84
		Upper 95% CI:	1.38

Test	Control
(GSM96822 + GSM96823) / 2<br>Ago1 knock down clone #1 replicate 1<br>Ago1 knock down clone #1 replicate 2	(GSM96832 + GSM96833) / 2<br>shRNA ctrl replicate 1 exp2<br>shRNA ctrl replicate 2 exp2
(GSM96824 + GSM96825) / 2<br>Ago1 knock down clone #2 replicate 1<br>Ago1 knock down clone #2 replicate 2	(GSM96832 + GSM96833) / 2<br>shRNA ctrl replicate 1 exp2<br>shRNA ctrl replicate 2 exp2

Table 6.6 shows the experiment GSE4246 HEK293T AGO3sh. A gene either possesses LINE-1 (denoted by L1) or does not possess LINE-1 (denoted by "No L1"). The up/down regulation of a gene (denoted by "Up" and "Down") is determined by paired t-test (p-value threshold is set at 0.05). The entries in the 2x2 tables show the resulting number of genes. The p-values of 2x2 tables are obtained from Chi-square distribution. The 2 tests and 2 controls in the t-test are shown below.

	Up (p<0.05)	Not up
L1	39	708
No L1	786	9,205

P-value:	8.81E-03		
Odds Ratio:	0.65	Lower 95% CI:	0.46
		Upper 95% CI:	0.90

Test	Control
GSM96820 Ago3 knock down replicate 1	GSM96816 shRNA ctrl replicate 1
GSM96821 Ago3 knock down replicate 2	GSM96817 shRNA ctrl replicate 2

Table 6.7 shows the experiment GSE4246 HEK293T AGO4sh. A gene either possesses LINE-1 (denoted by L1) or does not possess LINE-1 (denoted by "No L1"). The up/down regulation of a gene (denoted by "Up" and "Down") is determined by paired t-test (p-value threshold is set at 0.05). The entries in the 2x2 tables show the resulting number of genes. The p-values of 2x2 tables are obtained from Chi-square distribution. The 2 tests and 2 controls in the t-test are shown below.

	Up (p<0.05)	Not up
L1	54	650
No L1	634	9,066

P-value:	2.42E-01		
Odds Ratio:	1.19	Lower 95% CI:	0.89
		Upper 95% CI:	1.59

Test	Control
(GSM96826 + GSM96827) / 2)<br>Ago4 knock down clone #1 replicate 1<br>Ago4 knock down clone #1 replicate 2	(GSM96832 + GSM96833) / 2<br>shRNA ctrl replicate 1 exp2<br>shRNA ctrl replicate 2 exp2
(GSM96828 + GSM96829) / 2<br>Ago4 knock down clone #2 replicate 1<br>Ago4 knock down clone #2 replicate 2	(GSM96832 + GSM96833) / 2<br>shRNA ctrl replicate 1 exp2<br>shRNA ctrl replicate 2 exp2
