# Supplementary material for: Hypomethylation of Intragenic LINE-1 Represses Transcription in Cancer Cells through AGO2
Source: PLoS One. 2011 Mar 15;6(3):e17934. doi: 10.1371/journal.pone.0017934 (PMC3057998; doi:10.1371/journal.pone.0017934)
Supplement: Table S7 — Odds ratios, p values and 2×2 contingency tables of chi-square tests comparing between expression of genes possessing nearby intergenic L1s and the rest of genes in bladder carcinoma situ. The GSE and GSM records were listed in supporting table S3.7. (PDF) [file pone.0017934.s009.pdf]

Supporting file table S7: Odds ratios, p values and 2x2 contingency tables of chi square tests comparing between expression of genes possessing nearby intergenic L1s and the rest of genes in bladder carcinoma situ. The GSE and GSM records were listed in supporting table S3.7.

Location from gene	Direction of L1	Number of L1	Regulation	OR	95%CI	P value
1 kb 5′ or 3′	Sense or antisense	29	Down	1.30	0.61-2.80	0.498
1 kb 5′ or 3′	Antisense	14	Down	0.99	0.31-3.16	0.986
1 kb 5′ or 3′	Sense	16	Down	1.93	0.72-5.17	0.186
1 kb 5′	Sense or antisense	7	Down	1.86	0.42-8.30	0.411
1 kb 5′	Antisense	3	Down	1.24	0.11-13.65	0.862
1 kb 5′	Sense	5	Down	3.71	0.62-22.23	0.123
1 kb 3′	Sense or antisense	23	Down	1.32	0.56-3.12	0.525
1 kb 3′	Antisense	12	Down	1.24	0.37-4.11	0.728
1 kb 3′	Sense	12	Down	1.77	0.56-5.57	0.324
1 kb 5′ or 3′	Sense or antisense	29	Up	0.49	0.19-1.29	0.142
1 kb 5′ or 3′	Antisense	14	Up	0.65	0.18-2.32	0.499
1 kb 5′ or 3′	Sense	16	Up	0.34	0.08-1.49	0.132
1 kb 5′	Sense or antisense	7	Up	1.78	0.40-7.95	0.445
1 kb 5′	Antisense	3	Up	4.74	0.43-52.31	0.161
1 kb 5′	Sense	5	Up	0.59	0.07-5.30	0.636
1 kb 3′	Sense or antisense	23	Up	0.23	0.05-0.96	0.027*
1 kb 3′	Antisense	12	Up	0.22	0.03-1.67	0.105
1 kb 3′	Sense	12	Up	0.22	0.03-1.67	0.105
2 kb 5′ or 3′	Sense or antisense	72	Down	1.16	0.71-1.91	0.553
2 kb 5′ or 3′	Antisense	33	Down	1.24	0.60-2.55	0.564
2 kb 5′ or 3′	Sense	40	Down	1.19	0.61-2.31	0.603
2 kb 5′	Sense or antisense	23	Down	1.59	0.69-3.68	0.273
2 kb 5′	Antisense	7	Down	3.30	0.74-14.76	0.974
2 kb 5′	Sense	17	Down	1.35	0.50-3.65	0.553
2 kb 3′	Sense or antisense	50	Down	1.06	0.58-1.94	0.849
2 kb 3′	Antisense	27	Down	1.04	0.46-2.38	0.923
2 kb 3′	Sense	24	Down	1.24	0.53-2.89	0.622
2 kb 5′ or 3′	Sense or antisense	72	Up	0.62	0.35-1.10	0.099
2 kb 5′ or 3′	Antisense	33	Up	0.76	0.34-1.68	0.494
2 kb 5′ or 3′	Sense	40	Up	0.50	0.22-1.14	0.091
2 kb 5′	Sense or antisense	23	Up	0.66	0.24-1.77	0.404
2 kb 5′	Antisense	7	Up	0.95	0.18-4.89	0.949
2 kb 5′	Sense	17	Up	0.51	0.15-1.77	0.277
2 kb 3′	Sense or antisense	50	Up	0.59	0.30-1.18	0.133
2 kb 3′	Antisense	27	Up	0.68	0.27-1.68	0.396
2 kb 3′	Sense	24	Up	0.47	0.16-1.39	0.163

* Statistic significant
